# Effects of a short‐term cold exposure on circulating microRNAs and metabolic parameters in healthy adult subjects

**DOI:** 10.1111/jcmm.17121

**Published:** 2021-12-17

**Authors:** Marc Thibonnier, Sujoy Ghosh, Anne Blanchard

**Affiliations:** ^1^ AptamiR Therapeutics, Inc. Naples Florida USA; ^2^ Duke‐NUS Medical School Singapore City Singapore; ^3^ Pennington Biomedical Research Center Baton Rouge Louisiana USA; ^4^ Clinical Investigation Center Hôpital Européen Georges Pompidou Paris France

**Keywords:** circulating miRNAs, cold exposure, metabolic parameters, miRNAs/mRNAs co‐expression networks

## Abstract

This discovery study investigated in healthy subjects whether a short‐term cold exposure may alter circulating microRNAs and metabolic parameters and if co‐expression networks between these factors could be identified. This open randomized crossover (cold vs no cold exposure) study with blind end‐ point evaluation was conducted at 1 center with 10 healthy adult male volunteers. Wearing a cooling vest perfused at 14°C for 2 h reduced the local skin temperature without triggering shivering, increased norepinephrine and blood pressure while decreasing copeptin, C‐peptide and heart rate. Circulating microRNAs measured before and after wearing the cooling vest twice (4 time points) identified 196 mature microRNAs with excellent reproducibility over 72 h. Significant correlations of microRNA expression with copeptin, norepinephrine and C‐peptide were found. A co‐expression‐based microRNA‐microRNA network, as well as microRNA pairs displaying differential correlation as a function of temperature were also detected. This study demonstrates that circulating miRNAs are differentially expressed and coregulated upon cold exposure in humans, supporting their use as predictive and dynamic biomarkers of cardio‐metabolic disorders.

## 1 INTRODUCTION

MicroRNAs (miRNAs) play important roles in the pathogenesis of several metabolic disorders including obesity, type 2 diabetes, dyslipidaemia and metabolic dysfunction‐associated fatty liver disease (MAFLD).^1^, ^2^, ^3^, ^4^, ^5^, ^6^ Circulating miRNAs are associated with vesicles (exosomes, microparticles and apoptotic bodies), protein complexes (Ago2, NPM1) and lipoprotein complexes (HDL, LDL complexes).^7^, ^8^ Circulating miRNAs are regarded as promising noninvasive biomarkers for risk stratification, diagnosis and prognosis of various cardiometabolic diseases.^9^, ^10^, ^11^, ^12^ In addition, circulating miRNAs are mediators of intercellular signalling.^13^ It has been proposed that circulating miRNAs behave like hormones in intercellular communications.^14^ For instance, the adipose tissue is a major source of circulating miRNAs that can regulate gene expression in distant metabolic tissues and organs.^15^ miRNAs have also been identified as extensive regulators of adipocyte development, differentiation and biologic functions,^16^ often through their effects on glucose and lipid metabolism.^6^ This has raised the exciting prospect of using miRNAs as therapeutic targets in obesity and obesity‐associated metabolic dysfunction.^6^, ^17^, ^18^, ^19^ We recently provided evidence for this prospect by demonstrating that inhibition of miRNA‐22‐3p could lead to a potent treatment of fat accumulation, insulin resistance and related complex metabolic disorders like obesity, type 2 diabetes mellitus and nonalcoholic fatty liver disease.^20^, ^21^

Short‐term cold exposure activates oxidative metabolism in adipose tissues and increases total energy expenditure.^22^, ^23^, ^24^ While many aspects of cold exposure, especially those pertaining to brown fat activation, have been widely studied in animal models, there is less known about short‐term cold exposures in humans. Even less information exists on the molecular consequences of such exposures, and the association between molecular mediators and circulating metabolic effectors. In this study, we have investigated the effects of short‐term cold exposure of adult human subjects on changes in circulating miRNAs and their association with circulating metabolic effectors also regulated by such activation. Current advances in network biology indicate that molecular regulators such as mRNAs and miRNAs seldom function alone but rather make joint contributions through functional associations such as gene co‐expression networks. It is also becoming increasingly clear that differential correlation among molecular mediators (in addition to differential expression) is an equally important mediator of disease processes, such that changes in co‐expression, rather than differential expression, among molecular regulators become important.^25^ Based on these premises, we have characterized circulating ‘miRNA‐miRNA’ and ‘miRNA‐metabolite’ co‐expression networks among robustly expressed miRNAs, and also examined the extent of differential correlation among miRNAs as a function of cold exposure. To our knowledge, such a network‐based analysis of circulating miRNAs and their association with metabolic mediators in response to brief cold exposures in humans have not been reported.

## 2 MATERIAL AND METHODS

### 2.1 Study design

The primary objective of the study was to characterize the effect of short‐term cold exposure on the profile of miRNAs, metabolic and hormonal parameters released in the peripheral circulation of healthy adults. The secondary objective was to look for correlations between such miRNAs and metabolic or hormonal parameters. Safety measurements included adverse event assessment and physical examination, blood pressure (BP), heart rate (HR), electrocardiogram (ECG) and laboratory parameters (haematology, biochemistry, urinalysis, serologies and drugs screening), skin and core temperatures and visual analogue scale grading discomfort symptoms.

This was an open randomized crossover (cold vs. the absence of cold exposure) study with blind end‐point evaluation conducted at 1 centre in healthy adult male volunteers (Appendix 1). To alter body temperature, the participants put on an appropriately sized surgeon's cooling vest (Cool Flow Fitted Vest System^®^, POLAR Products, Figure S1). The use of the cooling vests provided for the participants involved in the study was strictly restricted to those participants and disinfected before each use. Following a predefined randomization code, the temperature of the water circulating inside the vest was set at 14°C (cold test) or kept at room temperature (RT, sham test) and monitored by a digital thermometer.

The protocol was approved before study initiation by the Agence Nationale de Sécurité du Médicament (ANSM) (Protocol I.D.: 2013‐A0110‐45) and the Comité de Protection des Personnes (CPP) Ile de France III (Protocol I.D.: 2013‐003033‐14). The study was performed in accordance with the ethical principles stated in the Declaration of Helsinki and French laws. All the subjects were fully informed of the risks and methodologies of the study. They all provided a signed and dated written consent to participate in the study.

The execution of the study is detailed in Appendix 1. A sufficient number (20) of subjects were included, so that a total of 10 subjects will complete the study.

To be included in the study, the subjects had to meet the following criteria:

1. **Inclusion criteria:** Healthy male, Caucasian, 18–35 years old, nonsmoker, stable BMI between 18 and 27 kg/m^2^, normal clinical examination including normal blood pressure, ECG and routine lab work, ability to tolerate a cooling vest set at 14°C for 2 h, no chronic pharmacologic treatment, signed and dated informed consent form.
2. **Exclusion criteria:** Intense physical activity, attempt to modify body weight by diet and/or therapies, inclusion into another clinical study within the last 3 months, alcohol use exceeding 21 drinks per week, alcohol consumption within 2 days prior to inclusion into the study, illicit drug use within the last 12 months, therapies resulting in potential dependence (e.g., sedatives and hypnotics), positive urine drug screen, blood donation during the prior 3 months or the intention to give blood during the next 3 months, blood transfusion within the last 12 months.

### 2.2 Laboratory parameters

The metabolic and hormonal parameters measured during the study are listed in Appendix 1. The assays were performed locally following manufacturers’ recommendations. The circulating miRNAs were extracted using the miRNeasy serum/plasma kit from Qiagen (miRNeasy Serum/Plasma Advanced Kit, Cat No./ID: 217204). miRNA sequencing was performed at the Genome Sequencing and Analysis Facility at the University of Texas at Austin on an Illumina HiSeq 2500 (Run SR50) system following the manufacturer's protocol. The results are expressed in reads per million mapped reads (rpm, linked to depth of sequencing) and not in reads per kilobase per million mapped reads (rpkm, linked to gene length) as the analysis was centred on miRNAs.

### 2.3 Statistical analyses

Based on our own experience and prior reports involving human subjects, we considered 10 completed subjects to provide adequate information for descriptive statistical analyses. Results in the text and data points in the figures are shown as the mean ± SEM. Statistical analysis used ANOVA and Student's *t*‐test, unless nonparametric tests were selected, based on data distribution (GraphPad Prism 8). The type I error of the statistical analyses was set at 5%. As the study is exploratory, no adjustment to control the type I error was used. Descriptive statistics are provided by period (cold vs sham exposure). The effect was investigated using an analysis of variance appropriate for crossover design. The study baseline is the last observation before the first period wearing the surgeon's cooling vest and the period baseline is the last observation before wearing the surgeon's cooling vest at each period.

### 2.4 Bioinformatic analysis of miRNA expression data

Data obtained from miRNA sequencing were analysed to generate normalized read counts for each sample.^26^ Pearson correlations between miRNA expression levels and blood analytes were estimated via the *psych* package in R (https://CRAN.R‐project.org/package=psych.). A miRNA‐miRNA and miRNA‐analyte co‐expression network was constructed based on graphical Gaussian models and shrinkage‐based estimations of partial correlation of miRNA and selected analyte expression profiles measured across 2 temperatures and 2 time points, via the *GeneNet* package in R (http://strimmerlab.org/software/genets/). The co‐expression network was visualized in Cytoscape (v.3.8, http://www.cytoscape.org/). Differential correlation analysis was conducted via the DiffCorr package in R (http://diffcorr.sourceforge.net/) to identify miRNA pairs that show statistically significant differences in correlation with expression between 14°C and RT.

## 3 RESULTS

### 3.1 Participants

Appendix 1 summarizes the baseline characteristics of the 10 subjects who completed the study. As outdoor temperature can rapidly affect nonshivering thermogenesis, we performed both tests (vest worn at RT and at 14°C) in each volunteer only 3 days apart. The study of the 10 volunteers was completed over the span of 4 weeks. Female were not included in the study as breast tissue may have affected the cooling effect of the vest and to avoid hormonal variations related to menstrual cycle.

### 3.2 Effects of cold exposure on various haemodynamic, metabolic and hormonal parameters

Based on the visual analogue scale of discomfort (Figure S2), wearing the cooling vest at RT produced no discomfort (score: 0 on a scale from 0 to 10) in 7 subjects and mild discomfort (score: 1) in 3 subjects. Wearing the cooling vest at 14°C for 2 h produced no discomfort in 4 subjects and mild discomfort (score 1 to 3) in 6 subjects. No shivering was reported nor observed.

**FIGURE 1:**
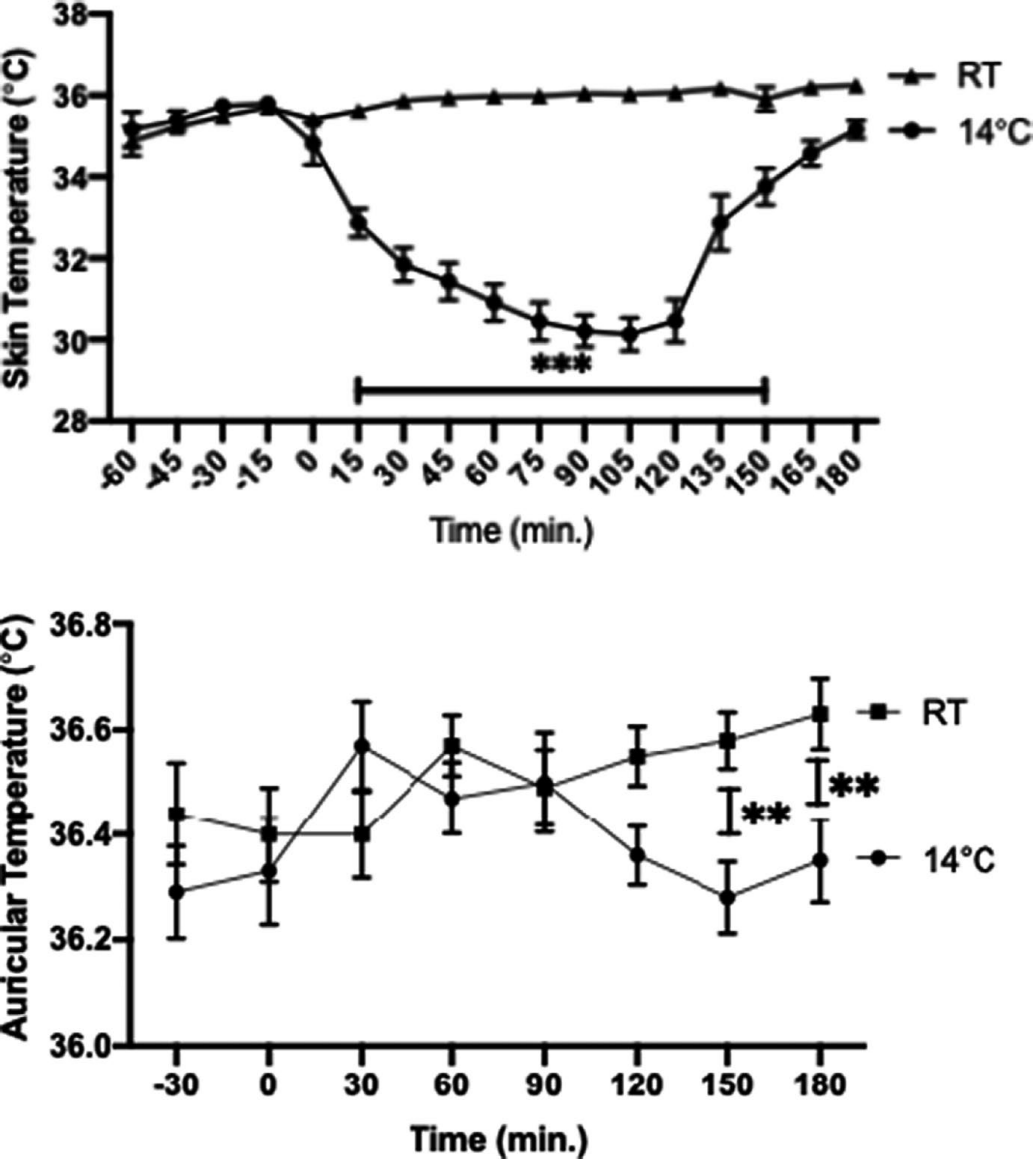
Skin and body temperature profile

Wearing the cooling vest at 14°C produced a significant decrease of local skin temperature from an average of 35.80°C at time point 0 min to an average nadir of 30.12°C at time point +105 min, followed by recovery within 1 h, whereas wearing the cooling vest at RT did not alter skin temperature (Figure 1). During the test at 14°C, body temperature trended downwards with time to a nadir of 36.28°C at time point 150 min (Figure 1). During the test at RT, the body temperature trended upwards with time from 36.44 to 36.63°C from time point −30 min to time point +180 min.

**FIGURE 2:**
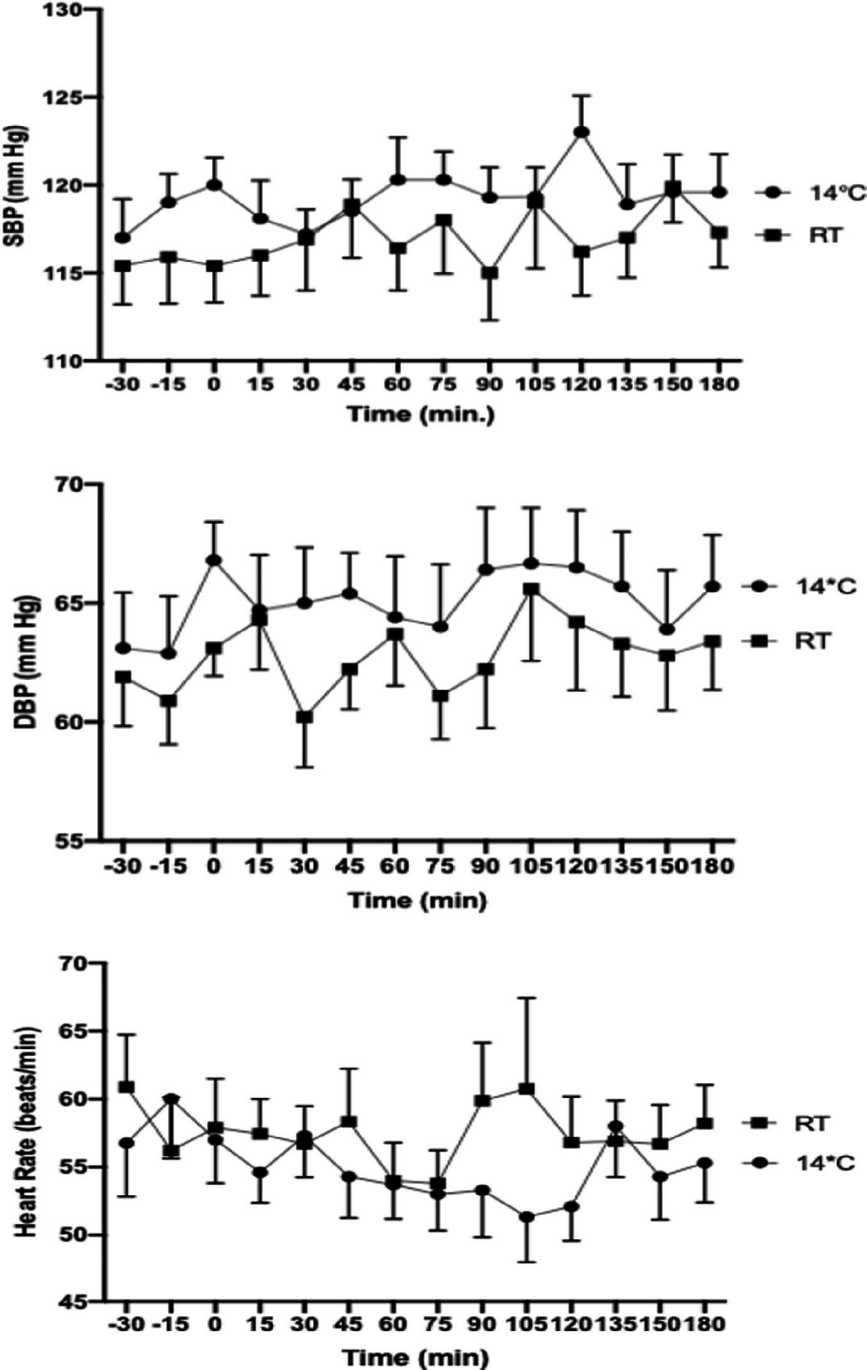
Blood pressure and heart rate profile

Both systolic (SBP) and diastolic (DBP) blood pressures were slightly higher during the test at 14°C than during the test at RT (Figure 2). Average SBP was 119.3 ± 0.40 mm Hg at 14°C and 116.9 ± 0.40 mm Hg at RT, *p* = 0.0009. SBP AUC over 100 mm Hg was 14% higher during the test at 14°C than during the test at RT (251.8 ± 15.38 vs. 220.9 ± 20.29, *p* = 0.0431). Average DBP was 65.08 ± 0.35 mm Hg at 14°C and 62.78 ± 0.39 mm Hg at RT, *p* ≤ 0.0001. DBP AUC over 55 mm Hg was 30% higher during the test at 14°C than during the test at RT (131.8 ± 18.59 vs. 101.3 ± 16.87, *p* = 0.0033). Heart rate was slightly slower during the test at 14°C than during the test at RT (Figure 2): Average HR was 55.07 ± 0.65 bpm at 14°C and 57.47 ± 0.57 mm Hg at RT, *p* = 0.0194. HR AUC over 50 bpm was 32% lower during the test at 14°C than during the test at RT (64.96 ± 25.19 vs. 94.98 ± 26.50, *p* = 0.0266).

**FIGURE 3:**
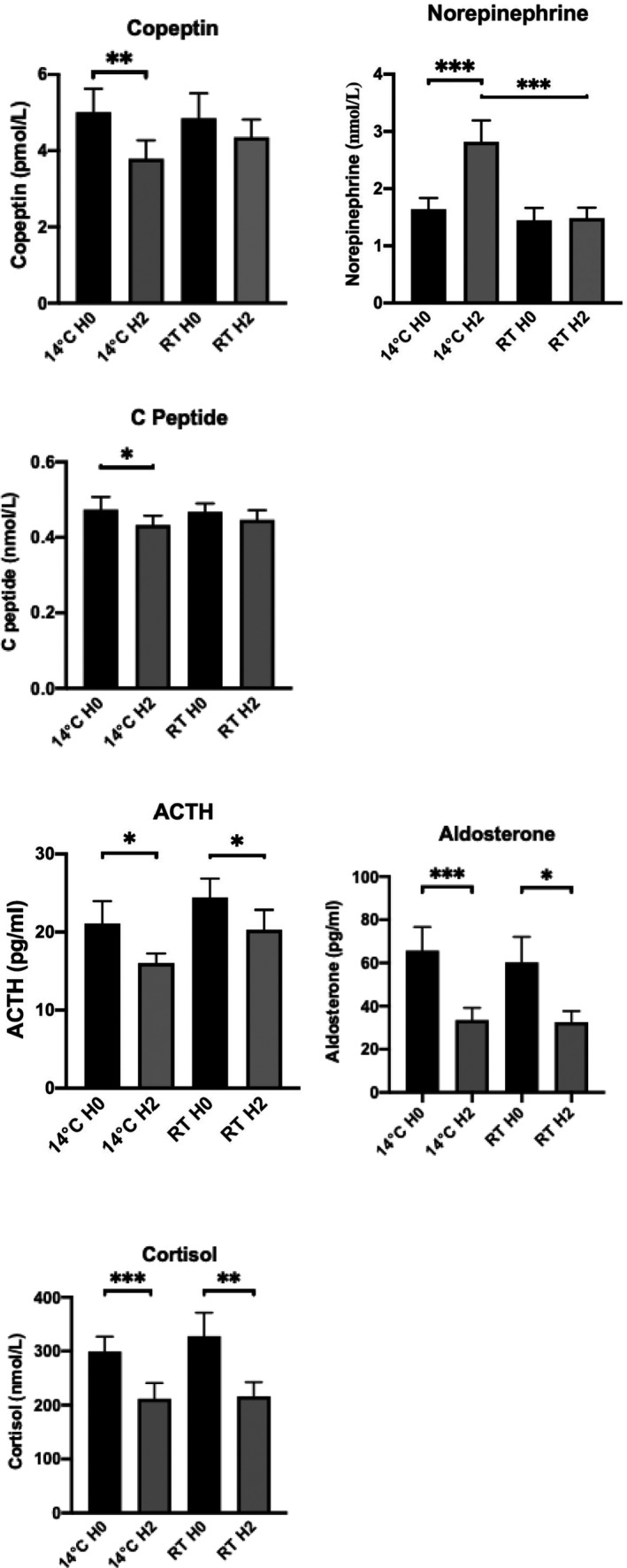
Relevant hormonal parameters

Three distinct patterns of circulating hormonal and metabolic variations were observed during cold exposure (Figure 3 and Table 1). First, there were metabolites that varied significantly only during cold exposure: increased norepinephrine as well as decreased copeptin and C‐peptide upon cold exposure. The second pattern involved metabolites (ACTH, aldosterone and cortisol) that trended downwards with time both at RT and during cold exposure, reflecting the hypothalamic‐pituitary‐adrenal (HPA) axis circadian rhythm. The third class included metabolites that did not show significant temporal variation either at RT or cold exposure, under the given conditions of the study (epinephrine, dopamine, renin, insulin, glucose, T3, T4, adiponectin, FGF21, lactic acid and pyruvic acid).

**TABLE 1:** Metabolic and hormonal parameters results (in alphabetic order)

Parameter	14°C H0	14°C H2	RT H0	RT H2
ACTH (pg/ml)	21.10 ± 2.82	16.00 ± 1.23**	24.40 ±2.42	20.30 ± 2.53**
Adiponectin (pg/ml)	3982 ± 466	3807 ± 410	3853 ± 449	3949 ± 448
LMW Adiponectin (pg/ml)	1317 ± 236	1272 ± 208	1242 ± 232	1361 ± 237
Aldosterone (pg/ml)	65.7 ± 10.95	33.5 ± 5.68***	60.3 ± 11.76	32.6 ± 5.08*
Copeptin (pmol/L)	5.01 ± 0.62	3.79 ± 0.49***	4.85 ± 0.66	4.35 ± 0.47
C‐peptide (nmol/L)	0.474 ± 0.03	0.433 ± 0.02**	0.468 ± 0.02	0.447 ± 0.03
Cortisol (nmol/L)	299.1 ± 27.83	211.7 ± 29.05***	327.6 ± 43.72	216.3 ± 26.22**
Dopamine (nmol/L)	0.318 ± 0.12	0.182 ± 0.05	0.182 ± 0.04	0.136 ± 027
Epinephrine (nmol/L)	0.227 ± 0.03	0.259 ± 0.04	0.295± 0.06	0.371 ± 0.05
FGF21 (pg/ml)	44.7 ± 9.77	42.4 ± 9.48	52.2 ± 11.62	45.9 ± 8.57
Glucose (mmol/L)	4.91 ± 0.06	4.86 ± 0.08	4.86 ± 0.01	4.92 ± 0.01
Insulin (mU/L)	3.27 ± 0.72	4.14 ± 0.61	3.40 ± 0.38	4.00 ± 0.26
Lactic Acid (mmol/L)	0.71 ± 0–14	0.76 ± 0.18	0.91 ± 0.20	0.91 ± 0.16
Norepinephrine (nmol/L)	1.64 ± 0.19	2.82 ± 0.37***, ^†††^	1.45 ± 0.22	1.48 ± 0.19
Pyruvic Acid (mmol/L)	74.75 ± 15.16	61.88 ± 12.65	86.30 ± 11.44	108.90 ± 19.34
Renin (mIU/L)	17.9 ± 2.05	14.9 ± 2.19	16.1 ± 2.31	14 ± 1.66
T3 (pmol/L)	4.73 ± 0.15	4.79 ± 0.17	4.94 ± 0.14	4.77 ± 015
T4 (pmol/L)	15.65 ± 0.64	15.22 ± 0.65	16.14 ± 1.00	16.13 ± 1.04
Triglycerides (mmol/L)	0.938 ± 0.20	0.916 ± 0.18	0.896 ±.013	0.853 ± 0.12

^*^ p<0.05, ^**^ p<0.01, ^***^ p<0.001.

^†††^ p<0.001.

### 3.3 Profile of circulating miRNAs at baseline and after cold exposure

The circulating miRNA identified before and after wearing the cooling vest twice are listed in Table S1. A total of 196 mature miRNAs were detected at ≥10 rpm. Across the 4 time points, 107–117 mature miRNAs were detected at ≥100 rpm and 38–41 were detected at ≥1000 rpm. The 10 most abundant miRNAs across the 4 time points were mir‐451a, mir‐26a‐5p, mir‐21‐5p, mir‐92a‐3p, mir‐126‐3p, mir‐22‐3p, let‐7g‐5p, mir‐486‐5p, let‐7i‐5p and mir‐30d‐5p. A total of 104 miRNAs detected at ≥100 rpm were shared between the 4 time points, suggesting an excellent reproducibility of the measurements. Over 80% of all measured miRNAs (365/442) displayed similar expression patterns across the time points (*t*‐test *p* > 0.05).

**FIGURE 4:**
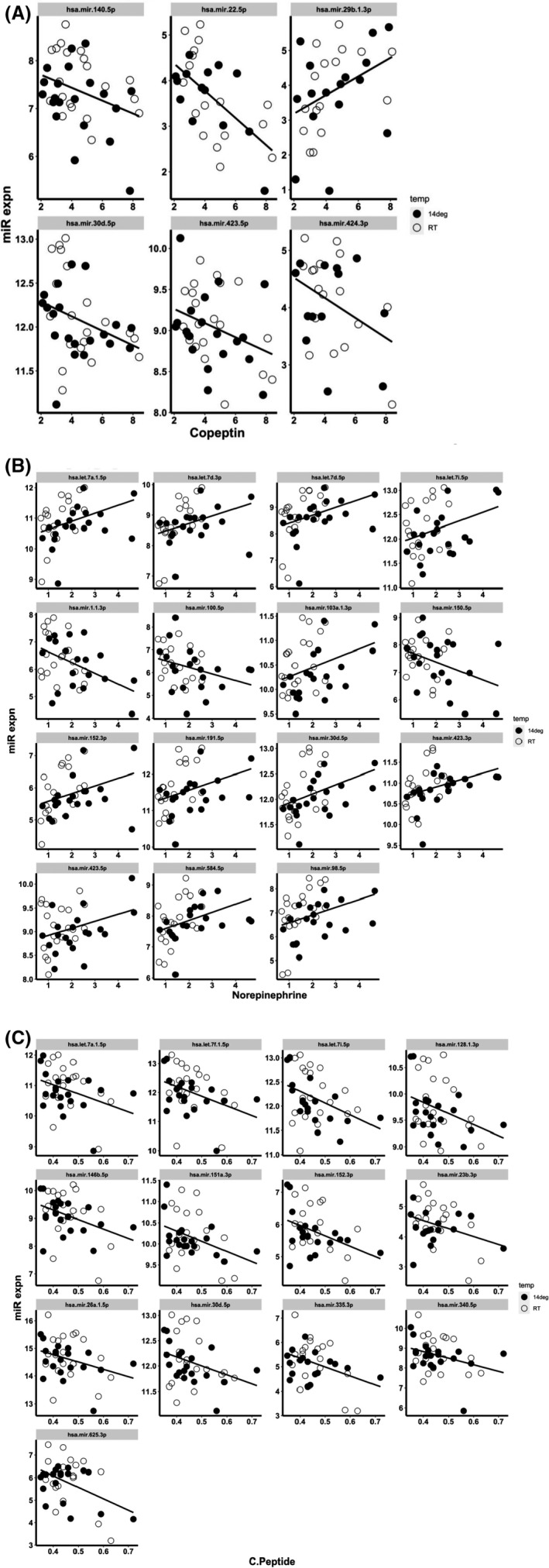
Correlation between circulating miRNAs and temperature‐sensitive blood metabolites. Correlation analysis was performed on miRNAs with nonzero expression values in all samples. Hormone/peptide levels are plotted on the *x*‐axis (pmol/L) and miRNA expression on the *y*‐axis (log2 scale). Open and closed circles represent data for RT and 14°C respectively. The correlation value and its significance are listed for each plot. Only correlations with *p* < 0.05 are shown. (A) Correlations between circulating miRNAs and copeptin levels, (B) correlations between circulating miRNAs and norepinephrine levels, (C) correlations between circulating miRNAs and C‐peptide levels

Correlations between circulating miRNAs and the metabolic/hormonal parameters which were significantly altered during the cold exposure (copeptin, norepinephrine and C‐peptide) are shown in Figure 4a–c. For copeptin, 5 miRNAs (miR‐140‐5p, miR‐22‐5p, miR‐30d‐5p, miR‐423‐5p and miR‐424‐3p) were negatively correlated and 1 miRNA (miR‐29b‐1‐3p) was positively correlated (Pearson correlation coefficient *p* ≤ 0.05, Figure 4a). For norepinephrine, 12 miRNAs (let‐7a‐1‐5p, let‐7d‐3p, let‐7d‐5p, let‐7i‐5p, miR‐103a‐3p, miR‐152‐3p, miR‐191‐5p, miR‐30d‐5p, miR‐423‐3p, miR‐423‐5p, miR‐584‐5p and miR‐98‐5p) were positively correlated and 3 miRNA (miR‐1–1‐3p, miR‐100‐5p and miR‐150‐5p) were negatively correlated (Pearson correlation coefficient *p* ≤ 0.05, Figure 4b). For C‐peptide, 13 miRNAs (let‐7a‐1‐5p, let‐7f‐1‐5p, let‐7i‐5p, miR‐128‐1‐3p, miR‐146b‐5p, miR‐151a‐3p, miR‐152‐3p miR‐23b‐3p, miR‐26a‐1‐5p, miR‐30d‐5p, miR‐335‐3p, miR‐340‐5p and miR‐625‐3p) were negatively correlated (Pearson correlation coefficient *p* ≤ 0.05, Figure 4c). Full details of the correlation analysis are provided in Table S2.

**FIGURE 5:**
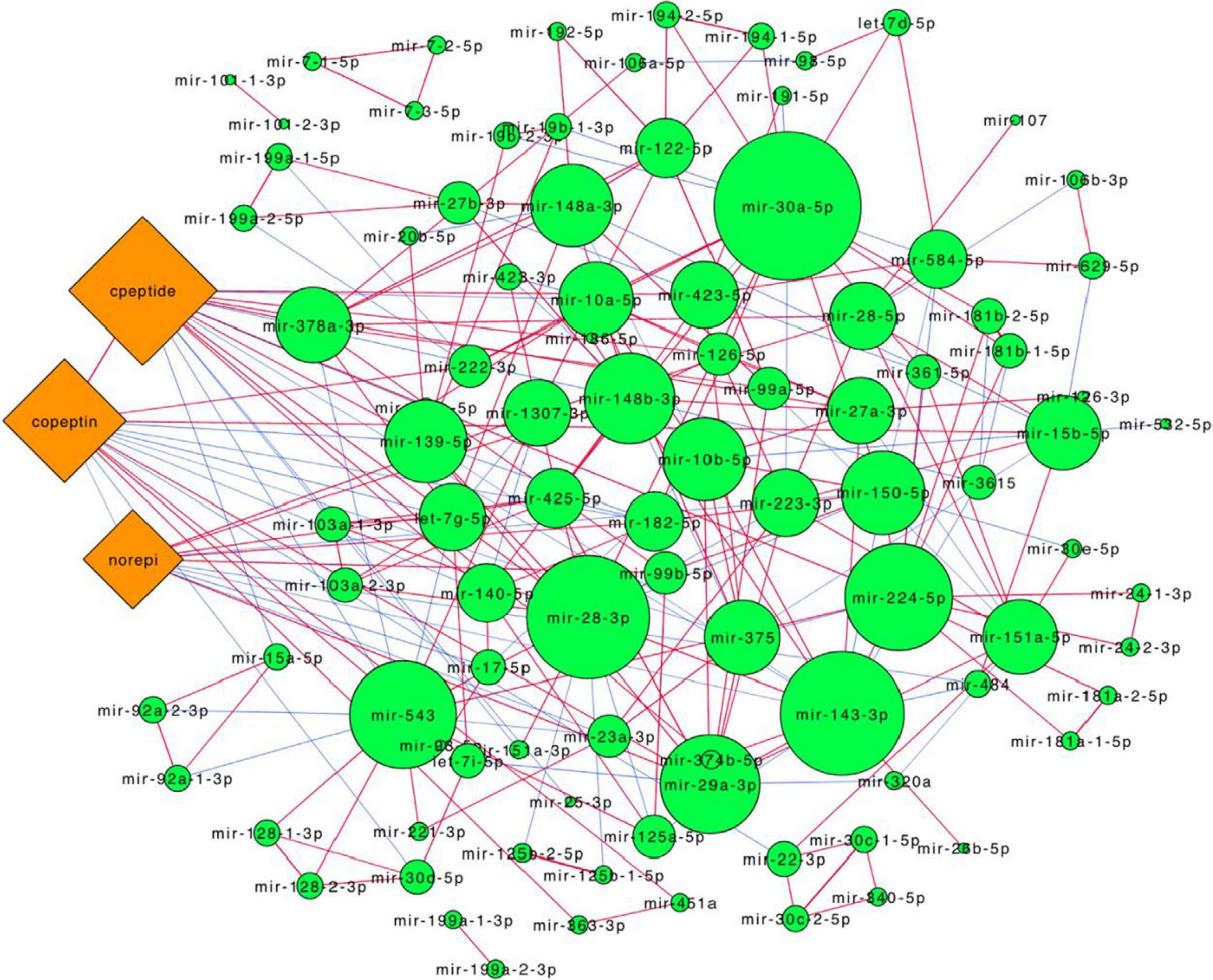
miRNA‐miRNA and miRNA‐metabolite correlation networks. Partial correlation based co‐expression networks were constructed from 118 miRNAs and 3 metabolite (copeptin, norepinephrine, C‐peptide) expression data. Network is restricted to nodes with a probability cut‐off of nonzero correlation >0.8. Nodes are shaped based on molecule type (miRNA, green circle; metabolite, orange diamond). Node size is directly proportional to node degree (density of connectivity to other nodes). Edges are colour coded based on the sign of partial correlation (red, positive; blue, negative). Network was generated in Cytoscape using the yFiles organic layout

We next investigated the relation among the expressed circulating miRNAs to infer a co‐expression network based on pairwise partial correlations among miRNAs. For this analysis, 118 miRNAs with nonzero expression values in all measurements were considered. Additionally, correlation of miRNA expression to copeptin, norepinephrine and C‐peptide were also considered during network generation. Figure 5 shows the miRNA‐miRNA and miRNA‐analyte networks based on nodes with a probability cut‐off for nonzero correlation >0.8. In the network, miRNAs are sized by their number of connections to other miRNAs/analytes. Some of the highly connected miRNAs include mir‐30a‐5p, mir‐148a‐3p, mir‐378a‐3p and mir‐143‐3p. The full output correlation analysis is provided in Table S3.

**FIGURE 6:**
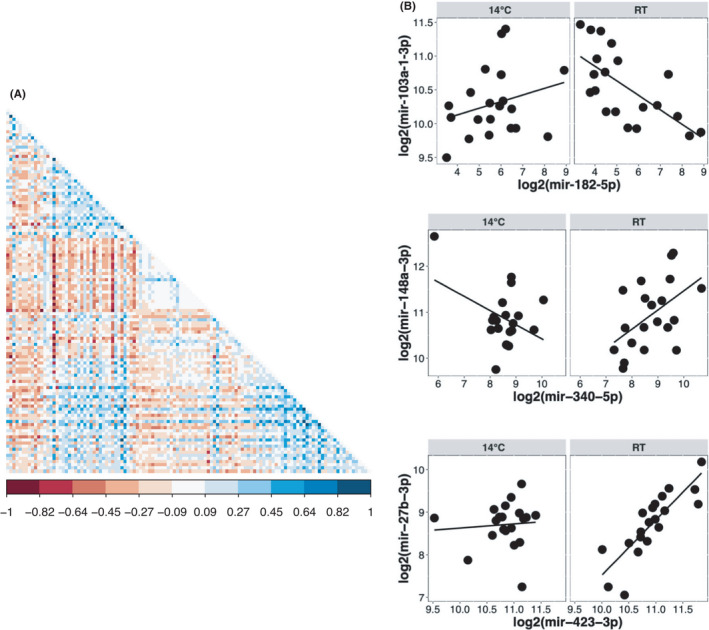
Differential correlation analysis of circulating miRNAs. Differential correlation analysis was conducted with 118 miRNAs to identify miRNA pairs that show significantly different correlations at 14°C compared to RT. (A) Hierarchical clustering of correlogram based on differences in correlation values at 14°C and RT. Each cell is the difference in correlation of a miRNA pair and is coloured according to the colour key shown at the bottom. (B) Examples of differential correlations among miRNA pairs. Correlations at 14°C and RT are shown side by side for miRNA pairs listed on the *x*‐ and *y*‐axis (in log 2 expression units)

An additional analysis was conducted to identify miRNA pairs that are differentially correlated with each other at 14°C compared to RT. The same set of 118 miRNAs with nonzero expression values at all measurements were used for this analysis. A total of 73 miRNA pairs were found to be significantly differentially correlated (*p* < 0.01) (full results provided in Table S4). The correlogram in Figure 6a shows a hierarchically clustered view of the differences in correlation values between miRNA pairs. Some specific examples of differential correlation are depicted in Figure 6b and include cases where the correlation signs are reversed between 14°C and RT (mir‐182‐5p/mir‐103a‐1‐3p, mir‐340‐5p/148a‐3p) or is weaker in one condition relative to the other (mir‐423‐3p/mir‐27b‐3p).

## 4 DISCUSSION

The communication between circulating miRNAs and target cells may lead to a series of effects on both physiological and pathological conditions.^27^ Cell‐secreted miRNAs facilitate the exchange of genetic information between cells and play an important role in intercellular communication.^28^ They are also implicated in physiological processes such as the regulation of immunity and angiogenesis or cellular migration, while they are also involved in various pathological conditions. Importantly, miRNA released from cells can be detected in various human body fluids including saliva, urine, blood, serum, plasma, seminal fluid and pleural effusion.^29^, ^30^ The expression profile of extracellular miRNAs in different bio‐fluids under different pathophysiological conditions displays specific patterns suggesting that such miRNAs are not passively released from the necrotic or injured cells, but rather selectively released from specific cells.^29^, ^30^ Circulating miRNAs exist in two distinct populations as vesicle‐associated and nonvesicle‐associated (e.g., as a ribonucleoprotein complex), respectively, with the majority existing in the nonvesicle‐associated form. In this study, we assayed total circulating miRNAs which reflects the abundances in the predominant vesicle‐free form.

Several recent studies identified circulating miRNAs as associated with markers of metabolic disorders including obesity, type 2 diabetes, NAFLD/NASH and liver fibrosis.^31^, ^32^, ^33^, ^34^, ^35^ Manning et al. reported a substantial dysregulation of 21 miRNAs that were associated with impaired glucose tolerance, senescence, cardiac hypertrophy, angiogenesis, inflammation and cell death in obese women.^36^ Ghavami et al. demonstrated a significant increase in circulating miR‐375 as well as a significant decrease in KLF5 mRNA expression after 6 weeks of insulin supplementation in diabetic patients.^37^ In another study employing RT‐PCR‐based analysis of the effects of diets on miRNA expression, Assman et al. reported 7 miRNAs (miR‐130a‐3p, miR‐142‐5p, miR‐144‐5p, miR‐15a‐5p, miR‐22‐3p, miR‐221‐3p and miR‐29c‐3p) as differentially expressed between responders and nonresponders to a low‐fat diet.^38^ Kurylowicz et al. showed that the expression of *SIRT1* in vascular adipose tissue of obese subjects was negatively correlated with the expression of miR‐22‐3p.^39^ Notably, we also recently observed that inhibition of microRNA‐22‐3p by complementary antagomirs in primary cultures of human subcutaneous adipocytes and in the diet‐induced obesity (DIO) mouse model resulted in increased lipid oxidation, mitochondrial activity and energy expenditure.^20^, ^21^

Short‐term cold exposure protocols have been used to explore the activation of thermogenesis in humans without triggering shivering and related metabolic responses due to increased muscular activity.^40^, ^41^, ^42^, ^43^ Our crossover protocol produced a maximum 5°C reduction in the thoracic skin temperature when the cooling vest was perfused at 14°C. The impact on core body temperature was a maximum decrease of −0.3°C without shivering. These findings are similar to those (−4.5 ± 0.3°C for skin temperature and −0.4 ± 0.1°C for core temperature) reported by Blondin et al. during subjects’ exposure to a suit cooled at 18°C for 180 min.^41^ The BP increase and HR decrease observed in our study were also similar to that reported by Cypess et al. who exposed healthy subjects to a surgeon's cooling vest cooled at 14°C for 120 min,^43^ presumably related to peripheral vasoconstriction and increase of central blood volume. Under similar conditions, we observed upon cold exposure a significant increase of norepinephrine reflecting the activation of the beta‐adrenergic signalling and peripheral sympathetic nervous system in responsive tissues such as brown fat.^44^ Our findings are in line with other studies that observed increased plasma norepinephrine, and increased norepinephrine turnover due to sympathetic activation of BAT following cold exposure.^45^, ^46^ The other circulating peptide significantly altered by cold exposure was copeptin, a surrogate biomarker of arginine‐vasopressin (AVP) secretion. AVP levels are most notably regulated through changes in plasma osmolality, as well as adaptations to physiological stress.^47^ In the absence of osmotic changes in the current study, the decrease in copeptin during cold exposure may suggest an alternative haemodynamic adjustment such as an increase in the central volume sensed by central baroreceptors. Notably, a decline of plasma vasopressin has also been reported during cold exposure in the context of normal hydration.^48^ As copeptin levels have also been associated with additional metabolic effects including lipid oxidation hyperinsulinaemia, metabolic syndrome and future type 2 diabetes, the observed changes in copeptin in our study might encompass additional physiologic processes besides haemodynamic control.^49^, ^50^ Finally, the observed changes in circulating insulin C‐peptide levels upon cold exposure is in line with existing literature linking insulin's requirement for cold‐induced thermogenesis, presumably due to alterations in insulin sensitivity following cold exposure.^51^ However, in a study involving human subjects exposed to thermoneutrality (22°C) or moderate cold (18°C) for 100 min, plasma C‐peptide was found to be unaltered, in contrast to our findings.^40^ The shorter exposure to a milder temperature reduction in that study may explain these different C‐peptide variations. As for the other effectors tested, wearing the cooling vest at 14°C did not alter the circadian rhythm of the pituitary‐adrenal axis components (ACTH, aldosterone and cortisol) which usually peak around 8:30 AM.^52^ Various hormonal and metabolic parameters (Adiponectin, LMW adiponectin, dopamine, epinephrine, FGF21, glucose, insulin, lactic acid, pyruvic acid, renin, T3, T4 and triglycerides) were also not altered by wearing the cooling vest at 14°C.

Several investigators have examined the association of circulating miRNAs to specific indices of BAT activation. For example, Saito et al. recently measured circulating miRNAs in male volunteers for 2 h to a room climatized at 19°C and placing their feet on a cloth‐wrapped ice block for 4 min every 5 min.^53^ They reported that circulating miR‐122‐5p was negatively correlated to brown adipose tissue activity measured by 18F‐FDG‐PET/CT at the end of the cold exposure period. Similarly, Pfeifer et al reported that serum concentrations of exosomal miR‐92a were inversely correlated with human BAT activity measured by 18F‐FDG PET/CT in 41 healthy individuals.^54^ To put these findings in context, both miR‐122‐5p and miR‐92a‐3p were highly expressed in our study, and miR‐122‐5p expression was further reduced during cold exposure with nominal significance (*p *< 0.05) (miR‐92a‐3p was not significantly altered). However, cold exposure‐induced circulating miRNAs are expected to also associate with other metabolic endpoints in addition to BAT activation. This possibility has been addressed in our study where the correlation of circulating miRNAs to cold‐induced changes in circulating hormones were compared. We further expanded the scope of examination beyond single miRNA‐metabolite correlations and investigated the co‐expression structure of circulating miRNAs and differences in such co‐expression networks as a function of cold exposure. These are the novel contributions from this study.

The number of circulating miRNAs found in our study is within the range reported by others in 12 body fluids.^55^, ^56^ The correlations between circulating miRNAs and the metabolic/hormonal parameters that were significantly altered during the cold exposure (copeptin, norepinephrine and C‐peptide) reveal that miR‐30d‐5p was correlated with all 3 parameters. Both positive and negative correlation values were observed, suggesting a complex interplay between circulating miRNA and analyte levels.

Expanding on the individual miRNA‐metabolite correlations, we generated a co‐expression network based on miRNA‐miRNA and miRNA‐metabolite partial correlation estimates. The network allowed us to further characterize the miRNAs based on their relationships with other miRNAs. For example, several miRNAs, such as mir‐30a‐5p, mir‐148a‐3p, mir‐378a‐3p, mir‐143‐3p, were found to be highly connected to multiple other miRNAs in the network (high degree), whereas other miRNAs, such as mir‐199a‐1‐3p and mir‐199a‐2‐3p, were only associated with each other and with no other miRNAs. Interestingly, the largest node belonged to miR‐30a‐5p, which has been reported to promote browning of adipocytes and insulin sensitivity.^57^, ^58^ Also, mir‐378‐3p, a regulator of energy and glucose homeostasis, was found to be one of the highly connected elements in the miRNA co‐expression network.^59^

While the co‐expression network provided a general view of miRNA‐miRNA associations, we also investigated if a subset of these associations is differentially regulated as a function of temperature. Of the significantly differentially correlated miRNAs included are mir‐148b‐3p and mir‐151a‐5p which are also some of the highly connected nodes in the miRNA co‐expression network. Although the functional relevance of these observations is currently unknown, they nevertheless point to a complex and dynamic interplay among circulating miRNA levels, possibly as a consequence or adaptation to a changing thermal stimulus.

Some limitations of the current study are now discussed. The small sample size limits the power to detect small differences in miRNA expression between the different time points, due to which we have not focused on differential miRNA expression in the study. Also, the limited timespan of the study may have prevented the development of greater differences in circulating miRNA levels. Nevertheless, this study provides a comprehensive inventory of circulating miRNAs in response to short‐term cold exposure in humans, and their association with important metabolic mediators, copeptin, C‐peptide and norepinephrine. Additionally, this study provides a network‐centric view of circulating miRNAs and also identifies miRNA pairs with altered co‐expression patterns upon cold exposure.

These results lay the foundation for further functional studies on the biological origins and consequences of the observed circulating miRNAs. For instance, circulating miRNAs are attractive and convenient biomarkers (‘liquid biopsies’) for various conditions. The miRs contained in exosomes (the exomiRs) are suitable candidates as noninvasive biomarkers and are critical factors involved in intercellular communications.^60^ Finally, circulating miRNAs are the targets of miRNA‐based therapeutic agents (such as miRNA mimetics, miRNA antagomirs and miRNA Sponges) currently in development for many pathologies.

## CONFLICT OF INTEREST

There is no conflict of interest to declare.

## AUTHOR CONTRIBUTIONS

**Marc Thibonnier:** Conceptualization (equal); Data curation (equal); Formal analysis (equal); Funding acquisition (equal); Methodology (equal); Project administration (equal); Supervision (equal); Writing – original draft (equal); Writing – review & editing (equal). **Sujoy Ghosh:** Data curation (equal); Formal analysis (equal); Software (equal); Validation (equal); Writing – original draft (equal); Writing – review & editing (equal). **Anne Blanchard:** Formal analysis (equal); Investigation (equal); Methodology (equal); Resources (equal); Supervision (equal); Writing – review & editing (equal).

## Supporting information

Figure S1–S2

Table S1

Table S2

Table S3

Table S4

## Data Availability

All data generated or analysed during this study are included in this published article or in the data repositories listed in References.

## APPENDIX 1 Study Execution

All subjects reported to the CIC for 1 screening visit, 2 one‐day in‐patient stays, and 1 follow‐up visit. All study assessments and procedures were timed related to the first CIC in‐patient stay. Subjects were permitted a window of ±2 days for each scheduled visit.

- During the initial screening visit (Day‐28 to day‐7) in the CIC:
  - The study protocol was fully explained to the subjects and their questions answered.
  - Signed and dated written informed consent forms were obtained.
  - A full history and physical examination were performed.
  - The subjects were asked to try on an appropriately sized Cooling Vest to verify that:
    1. The vest can be well adjusted to their morphology and
    2. The subjects feel comfortable wearing the vest
  - A licensed phlebotomist drew blood (amount of up to 30 ml) from an arm vein for routine and safety lab work.
  - A 12‐lead ECG was recorded.
  - A calendar of the actions to be taken during the next weeks as part of the study was given to the study participants.
  - For the upcoming weeks, the participants were asked to follow the same healthy lifestyle and maintain their weight. Involvement in strenuous physical activity was prohibited. Proper hydration was maintained.
  - The participants were asked to refrain from caffeine and alcohol intake for 48 h prior to reporting to the CIC the evening before Study Day 1 and Study Day 3.
- On Study Day 1:
  - The participants were admitted to the CIC in the evening (20h00).
  - A normocaloric meal was served at around 20h30
  - RT was maintained at 23°C throughout the stay in the CIC.
  - Fasting was initiated at midnight onwards.
- On Study Day 1 in the CIC:
  - Upon waking up at 07h00, the participants wore a standard hospital scrub suit.
  - A physical examination was performed.
  - Vital signs (blood pressure (BP), heart rate (HR), skin (thorax level) and core temperatures were recorded every 15 min from 08h00 to 12h00 in supine position.
  - Eighty (80) ml of venous blood was drawn before 09h00 for baseline miRNAs and metabolic/hormonal parameters measurements
  - Cutaneous and central temperature will be measured every 15 min from 08h00 to 12h00 with skin thermistors placed under the vest and electronic thermometers placed in the ear.
  - At 09h00, the participants put on a surgeon's cooling vest (Cool Flow Fitted Vest System^®^, POLAR Products, Figure S1).
  - Following a predefined randomization code (RANDI2, http://www.randi2.org/), the temperature of the water circulating inside the vest was set at 14°C (cold test) or kept at RT (sham cold) and monitored by a digital thermometer.
  - The participants rested comfortably in bed in a supine position and were exposed to a total of 120 min of cold (or sham cold) from 09h00 to 11h00.
  - At the end of the cold (or sham cold) exposure period (11h00), 80 ml of venous blood was drawn for cold phase (or sham cold) miRNAs and metabolic/hormonal parameter measurements.
  - By the end of the morning, the subjects were fed and discharged from the CIC. They were given the date and time of the second stay in the CIC and were reminded to continue the same healthy lifestyle and to avoid caffeine and alcohol intake.
- On Study Day 2:
  - The participants were admitted to the CIC in the evening (20h00).
  - A normocaloric meal was served at around 20h30
  - They were fasting from midnight onwards.
  - RT was maintained at 23ºC throughout the stay in the CIC.
- On Study Day 3 in the CIC:
  - The protocol applied on Study Day 1 in the CIC was repeated, but each subject was wearing the vest set at the temperature, which was not applied on Study Day 1.
  - By the end of the morning, which was the final visit:
    1. A full physical examination was completed.
    2. Assessment of adverse events was performed.
    3. The subjects were discharged from the CIC after being fed lunch.

Five venous blood draws totalling 350 ml of blood were collected over the duration of the study.

**Study Diagram:**

**Investigations schedule:**

**Table:**

Day number	−28 to −7	−6	−5	−4	−3	−2	−1	1	2	3
Informed Consent	X									
Selection/Inclusion criteria	X									
Medical History	X									
Vital Signs	X						X	X	X	X
Physical Examination	X							X		X
12‐lead ECG	X									
Laboratory Safety Panel*	X									
Urine screen for drugs abuse	X									
Baseline blood work	X									
Enrolment	X									
Admission to CIC							X		X	
Hormonal Blood Samples**								X		X
Central pulse pressure and pulse wave velocity								X		X
2‐h Cold or Sham Test								X		X
miRNA Blood Samples**								X		X
Discharge from CIC								X		X
Final visit										X
AE Reporting	X	X	X	X	X	X	X	X	X	X

*Including haematology (haemoglobin, haematocrit, erythrocytes, neutrophils, basophils, eosinophils lymphocytes, monocytes, leucocytes and platelets), blood biochemistry (sodium, potassium, calcium, chloride, uric acid, creatinine, fasting blood glucose, albumin, total protein, triglycerides, total cholesterol, total bilirubin, alkaline phosphatase, ASAT, ALAT, γGT), immunoserology (HIV, hepatitis B and hepatitis C) and urinalysis (specific gravity, pH, semi‐quantitative ‘dipstick’ evaluation of glucose, protein, ketones, leucocytes and blood).

**Performed right before and at the end of the 2‐h cold (or sham) exposure.

**Metabolic and hormonal parameters measured during the study:**

**Table:**

ACTH
Adiponectin
LMW Adiponectin
Aldosterone
Copeptin
C‐peptide
Cortisol
Dopamine
Epinephrine
FGF21
Glucose
Insulin
Lactic Acid
Norepinephrine
Pyruvic Acid
Renin
T3
T4
Triglycerides

**Baseline characteristics of the 10 subjects who completed the study:**

**Table:**

Baseline parameter	
Gender	All male
Age	26.1 ± 1.2 years
Race	All Caucasian
Height	179 ± 0.02 cm
Weight	73.95 ± 3.05 kg
Body Mass Index	23.1 ± 0.54 kg/m^2^
Systolic Blood Pressure	114.6 ± 1.46 mm Hg
Diastolic Blood Pressure	61.5 ± 1.23 mm Hg
Heart rate	58.9 ± 2.36 beats/min
