# Supplementary material for: Effects of a short‐term cold exposure on circulating microRNAs and metabolic parameters in healthy adult subjects
Source: J Cell Mol Med. 2021 Dec 17;26(2):548–62. doi: 10.1111/jcmm.17121 (PMC8743656; doi:10.1111/jcmm.17121)
Supplement: Supplementary file 1 — Figure S1–S2 [file JCMM-26-548-s001.pdf]

Supplemental Figure 1: Cooling vest used for the study

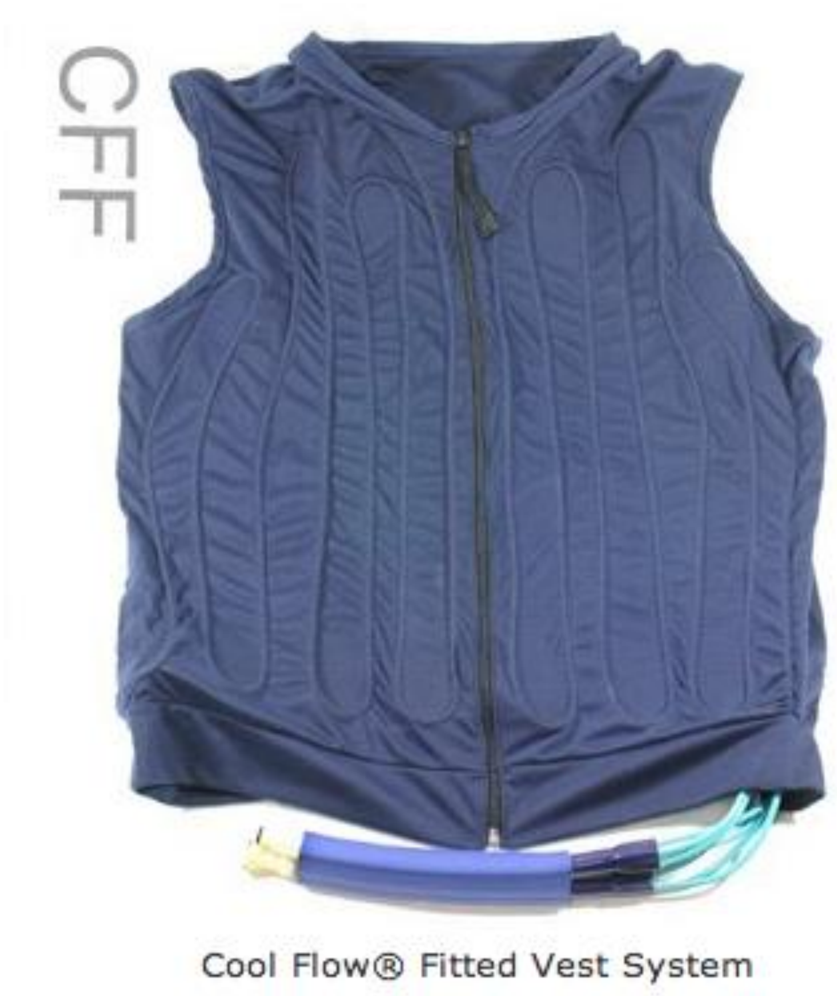

Supplemental Figure 2: Discomfort Visual Analog Scale used in the study

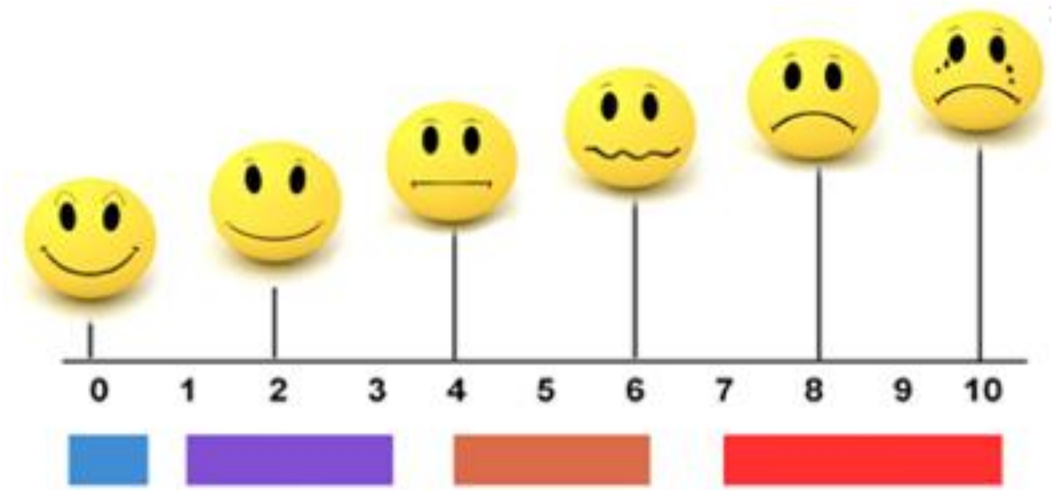

**None**	**Mild**	**Moderate**	**Severe**
