# Supplementary material for: Effects of a short‐term cold exposure on circulating microRNAs and metabolic parameters in healthy adult subjects
Source: J Cell Mol Med. 2021 Dec 17;26(2):548–62. doi: 10.1111/jcmm.17121 (PMC8743656; doi:10.1111/jcmm.17121)
Supplement: Supplementary file 2 — Table S1 [file JCMM-26-548-s005.docx]

**Supplemental Table 1: List of circulating mature miRNAs measured at the 4 different time points over 72 hours (before and after wearing the cooling vest twice)**

*Listing is in decreasing order of reads per million (rpm) for the top 100 miRNAs (Time point* 14ºC H0 was used as the reference*)*

|  | miRNA | **mean rpm**  **14ºC H0** | **mean rpm**  **14ºC H2** | **mean rpm**  **RT H0** | **mean rpm**  **RT H2** |
| --- | --- | --- | --- | --- | --- |
| 1 | mir-451a | **76,540** | **56,985** | **77,131** | **58,497** |
| 2 | mir-26a-2-5p | **23,695** | **27,257** | **26,492** | **34,979** |
| 3 | mir-26a-1-5p | **23,695** | **27,257** | **26,492** | **34,979** |
| 4 | mir-21-5p | **14,488** | **16,676** | **16,088** | **22,966** |
| 5 | mir-92a-2-3p | **9,934** | **11,364** | **10,513** | **8,086** |
| 6 | mir-92a-1-3p | **9,934** | **11,364** | **10,513** | **8,086** |
| 7 | mir-126-3p | **7,490** | **7,488** | **8,241** | **9,714** |
| 8 | mir-22-3p | **6,901** | **6,256** | **7,106** | **7,309** |
| 9 | mir-486-2-5p | **5,821** | **5,938** | **6,212** | **4,532** |
| 10 | mir-486-1-5p | **5,644** | **5,638** | **6,068** | **4,095** |
| 11 | let-7g-5p | **5,091** | **5,922** | **6,113** | **6,423** |
| 12 | mir-122-5p | **4,909** | **2,373** | **4,679** | **3,029** |
| 13 | let-7i-5p | **4,346** | **5,012** | **4,941** | **5,340** |
| 14 | mir-30d-5p | **4,118** | **4,502** | **4,712** | **4,859** |
| 15 | let-7f-2-5p | **4,014** | **4,475** | **4,562** | **5,675** |
| 16 | let-7f-1-5p | **4,014** | **4,475** | **4,562** | **5,675** |
| 17 | mir-146a-5p | **2,909** | **3,005** | **3,455** | **3,457** |
| 18 | mir-30e-5p | **2,866** | **2,895** | **3,034** | **3,184** |
| 19 | mir-191-5p | **2,834** | **3,044** | **3,609** | **3,420** |
| 20 | mir-26b-5p | **2,210** | **2,166** | **2,551** | **2,343** |
| 21 | mir-148a-3p | **2,139** | **2,011** | **1,993** | **2,464** |
| 22 | mir-20a-5p | **1,948** | **1,806** | **2,079** | **1,734** |
| 23 | mir-24-2-3p | **1,826** | **1,739** | **2,226** | **1,990** |
| 24 | mir-24-1-3p | **1,826** | **1,739** | **2,226** | **1,990** |
| 25 | let-7a-3-5p | **1,804** | **1,973** | **2,159** | **2,320** |
| 26 | let-7a-2-5p | **1,804** | **1,973** | **2,159** | **2,320** |
| 27 | let-7a-1-5p | **1,804** | **1,973** | **2,159** | **2,320** |
| 28 | let-7b-5p | **1,741** | **1,923** | **1,934** | **1,577** |
| 29 | mir-423-3p | **1,694** | **2,097** | **1,927** | **2,180** |
| 30 | mir-101-1-3p | **1,328** | **1,111** | **1,311** | **942** |
| 31 | mir-101-2-3p | **1,328** | **1,111** | **1,311** | **942** |
| 32 | mir-25-3p | **1,316** | **1,120** | **1,212** | **968** |
| 33 | mir-103a-2-3p | **1,258** | **1,413** | **1,598** | **1,607** |
| 34 | mir-103a-1-3p | **1,258** | **1,413** | **1,598** | **1,607** |
| 35 | mir-185-5p | **1,231** | **1,191** | **1,331** | **1,134** |
| 36 | mir-151a-3p | **1,160** | **1,169** | **1,307** | **1,282** |
| 37 | mir-93-5p | **1,034** | **1,031** | **1,181** | **944** |
| 38 | mir-199a-2-3p | **1,007** | **1,097** | **1,273** | **1,355** |
| 39 | mir-199a-1-3p | **1,007** | **1,097** | **1,273** | **1,355** |
| 40 | mir-199b-3p | **1,007** | **1,097** | **1,273** | **1,355** |
| 41 | mir-16-2-5p | **948** | **764** | **910** | **761** |
| 42 | mir-16-1-5p | **948** | **764** | **910** | **761** |
| 43 | mir-99a-5p | **919** | **625** | **896** | **827** |
| 44 | mir-27a-3p | **900** | **687** | **796** | **967** |
| 45 | mir-126-5p | **862** | **714** | **755** | **1,012** |
| 46 | mir-15a-5p | **810** | **830** | **832** | **655** |
| 47 | mir-128-1-3p | **794** | **899** | **912** | **999** |
| 48 | mir-128-2-3p | **794** | **899** | **912** | **999** |
| 49 | mir-221-3p | **756** | **763** | **937** | **837** |
| 50 | mir-320a | **666** | **654** | **796** | **616** |
| 51 | mir-17-5p | **599** | **545** | **678** | **613** |
| 52 | mir-146b-5p | **536** | **620** | **631** | **685** |
| 53 | mir-423-5p | **529** | **542** | **621** | **528** |
| 54 | mir-181a-2-5p | **487** | **494** | **543** | **633** |
| 55 | mir-181a-1-5p | **487** | **494** | **543** | **633** |
| 56 | mir-186-5p | **483** | **451** | **476** | **469** |
| 57 | mir-199a-1-5p | **480** | **491** | **394** | **758** |
| 58 | mir-199a-2-5p | **480** | **491** | **394** | **758** |
| 59 | mir-744-5p | **456** | **468** | **541** | **517** |
| 60 | mir-532-5p | **455** | **430** | **441** | **362** |
| 61 | mir-10b-5p | **453** | **338** | **400** | **478** |
| 62 | mir-27b-3p | **452** | **432** | **415** | **536** |
| 63 | mir-143-3p | **444** | **411** | **353** | **572** |
| 64 | mir-484 | **433** | **466** | **520** | **528** |
| 65 | mir-140-3p | **422** | **333** | **369** | **315** |
| 66 | let-7d-3p | **417** | **451** | **448** | **515** |
| 67 | mir-10a-5p | **406** | **339** | **375** | **453** |
| 68 | let-7d-5p | **378** | **455** | **494** | **500** |
| 69 | mir-340-5p | **375** | **471** | **424** | **605** |
| 70 | mir-192-5p | **328** | **211** | **314** | **240** |
| 71 | mir-144-3p | **317** | **443** | **589** | **313** |
| 72 | mir-222-3p | **303** | **233** | **298** | **262** |
| 73 | mir-99b-5p | **298** | **306** | **311** | **282** |
| 74 | mir-30c-1-5p | **292** | **351** | **328** | **469** |
| 75 | mir-30c-2-5p | **292** | **351** | **328** | **469** |
| 76 | mir-151a-5p | **277** | **65** | **70** | **485** |
| 77 | mir-106b-3p | **276** | **286** | **307** | **228** |
| 78 | mir-363-3p | **271** | **218** | **284** | **199** |
| 79 | mir-107 | **244** | **220** | **237** | **191** |
| 80 | mir-150-5p | **236** | **156** | **201** | **206** |
| 81 | mir-19b-2-3p | **229** | **179** | **237** | **179** |
| 82 | mir-19b-1-3p | **229** | **179** | **237** | **179** |
| 83 | mir-378a-3p | **222** | **177** | **220** | **213** |
| 84 | mir-584-5p | **218** | **253** | **291** | **233** |
| 85 | mir-23a-3p | **211** | **170** | **216** | **246** |
| 86 | mir-148b-3p | **207** | **237** | **214** | **281** |
| 87 | mir-328-3p | **202** | **261** | **263** | **271** |
| 88 | mir-1307-3p | **189** | **236** | **222** | **204** |
| 89 | mir-125a-5p | **189** | **169** | **206** | **170** |
| 90 | mir-106b-5p | **184** | **124** | **183** | **139** |
| 91 | mir-3615 | **169** | **145** | **160** | **120** |
| 92 | mir-7-3-5p | **166** | **144** | **166** | **122** |
| 93 | mir-7-1-5p | **166** | **144** | **166** | **122** |
| 94 | mir-7-2-5p | **166** | **144** | **166** | **122** |
| 95 | mir-361-5p | **160** | **153** | **167** | **169** |
| 96 | mir-20b-5p | **160** | **134** | **179** | **150** |
| 97 | mir-140-5p | **158** | **163** | **189** | **223** |
| 98 | mir-425-5p | **155** | **147** | **172** | **166** |
| 99 | mir-370-3p | **148** | **207** | **179** | **208** |
| 100 | mir-30a-5p | **136** | **136** | **125** | **164** |
