# Supplementary material for: Effects of a short‐term cold exposure on circulating microRNAs and metabolic parameters in healthy adult subjects
Source: J Cell Mol Med. 2021 Dec 17;26(2):548–62. doi: 10.1111/jcmm.17121 (PMC8743656; doi:10.1111/jcmm.17121)
Supplement: Supplementary file 3 — Table S2 [file JCMM-26-548-s002.pdf]

miRNA correlations to copeptin, C-peptide and norepinephrine. Column 1, miRNA name; col 2, correlation value; col 3, p-value of correlation; col 4, fdr estimate of correlation; col 5, analyte name; col 6, correlation method.

gene	r	p	fdr	phenotype	method
hsa.mir.369.5p	0.072067295	0.680773788	0.966407403	copeptin	pearson
hsa.mir.411.3p	0.01333307	0.943248323	0.97904585	copeptin	pearson
hsa.mir.29b.1.3p	0.390230549	0.027242512	0.893634813	copeptin	pearson
hsa.mir.29b.2.3p	0.332832924	0.067318665	0.893634813	copeptin	pearson
hsa.mir.941.1	0.331888249	0.073171311	0.893634813	copeptin	pearson
hsa.mir.654.5p	0.043690365	0.806189732	0.968587664	copeptin	pearson
hsa.mir.411.5p	0.103776356	0.55917882	0.935167048	copeptin	pearson
hsa.mir.181a.1.3p	-0.110908857	0.559580191	0.935167048	copeptin	pearson
hsa.mir.1307.5p	0.001779159	0.992159798	0.996476	copeptin	pearson
hsa.mir.493.3p	0.043887005	0.793609554	0.968587664	copeptin	pearson
hsa.mir.26b.3p	0.012006006	0.948889187	0.97904585	copeptin	pearson
hsa.mir.22.5p	-0.604527043	0.000315908	0.052756633	copeptin	pearson
hsa.mir.21.3p	-0.155724069	0.411234042	0.935167048	copeptin	pearson
hsa.mir.501.3p	0.13020548	0.477529579	0.935167048	copeptin	pearson
hsa.mir.454.3p	-0.007296719	0.967853404	0.97958496	copeptin	pearson
hsa.mir.486.1.3p	-0.029245509	0.861632661	0.97904585	copeptin	pearson
hsa.mir.323a.3p	0.13066519	0.491306738	0.935167048	copeptin	pearson
hsa.mir.144.5p	0.08833539	0.597930779	0.935167048	copeptin	pearson
hsa.mir.374a.5p	-0.256299064	0.125719416	0.893634813	copeptin	pearson
hsa.mir.148a.5p	0.051832929	0.770954867	0.968587664	copeptin	pearson
hsa.mir.495.3p	0.077806615	0.64242207	0.935411011	copeptin	pearson
hsa.mir.493.5p	-0.090684867	0.588188666	0.935167048	copeptin	pearson
hsa.mir.432.5p	-0.030651249	0.872260637	0.97904585	copeptin	pearson
hsa.mir.425.3p	-0.18698392	0.282124716	0.893634813	copeptin	pearson
hsa.mir.424.3p	-0.393925407	0.03124901	0.893634813	copeptin	pearson
hsa.mir.485.3p	0.226204807	0.178221905	0.893634813	copeptin	pearson
hsa.mir.369.3p	0.079525712	0.649746571	0.935411011	copeptin	pearson
hsa.mir.330.3p	-0.256800237	0.124955611	0.893634813	copeptin	pearson
hsa.mir.339.5p	-0.288432419	0.074958845	0.893634813	copeptin	pearson
hsa.mir.4433b.5p	-0.172201088	0.294517509	0.893634813	copeptin	pearson
hsa.mir.23b.3p	-0.106982178	0.528547595	0.935167048	copeptin	pearson
hsa.mir.1180.3p	0.019169298	0.910339431	0.97904585	copeptin	pearson
hsa.mir.654.3p	0.114370834	0.488129147	0.935167048	copeptin	pearson
hsa.mir.409.3p	0.17478365	0.287224906	0.893634813	copeptin	pearson
hsa.mir.181d.5p	-0.198251655	0.261031076	0.893634813	copeptin	pearson
hsa.mir.19a.3p	-0.048255863	0.789719107	0.968587664	copeptin	pearson
hsa.mir.421	0.020609642	0.909364442	0.97904585	copeptin	pearson
hsa.mir.339.3p	-0.114480768	0.506156446	0.935167048	copeptin	pearson
hsa.mir.342.3p	0.219627822	0.191516678	0.893634813	copeptin	pearson
hsa.mir.183.5p	-0.060300769	0.719124488	0.966407403	copeptin	pearson
hsa.mir.340.3p	-0.076813231	0.642089733	0.935411011	copeptin	pearson
hsa.mir.381.3p	0.126970203	0.441148281	0.935167048	copeptin	pearson
hsa.mir.370.3p	0.028314383	0.867880337	0.97904585	copeptin	pearson
hsa.mir.335.5p	-0.155214584	0.358983005	0.908335785	copeptin	pearson
hsa.mir.134.5p	0.135538066	0.423797782	0.935167048	copeptin	pearson
hsa.mir.494.3p	-0.021458986	0.910384026	0.97904585	copeptin	pearson
hsa.mir.125b.1.5p	-0.053316554	0.743862047	0.966407403	copeptin	pearson
hsa.mir.130a.3p	0.134113228	0.422120028	0.935167048	copeptin	pearson
hsa.let.7e.5p	-0.161227263	0.326819824	0.893634813	copeptin	pearson
hsa.mir.543	0.105693398	0.516281029	0.935167048	copeptin	pearson
hsa.mir.30b.5p	-0.233713187	0.157884298	0.893634813	copeptin	pearson

hsa.mir.625.3p	-0.183939537	0.268957183	0.893634813	copeptin	pearson
hsa.mir.335.3p	-0.251359253	0.133434932	0.893634813	copeptin	pearson
hsa.mir.197.3p	-0.096543996	0.564205519	0.935167048	copeptin	pearson
hsa.mir.130b.5p	-0.11545318	0.496218712	0.935167048	copeptin	pearson
hsa.mir.375	-0.229849342	0.153642643	0.893634813	copeptin	pearson
hsa.mir.18a.5p	-0.164688571	0.316401595	0.893634813	copeptin	pearson
hsa.mir.320b.1	-0.189543834	0.261182479	0.893634813	copeptin	pearson
hsa.mir.182.5p	-0.08702335	0.593378841	0.935167048	copeptin	pearson
hsa.mir.106a.5p	0.052620784	0.74709409	0.966407403	copeptin	pearson
hsa.mir.224.5p	0.013779081	0.932748697	0.97904585	copeptin	pearson
hsa.mir.127.3p	-0.068647696	0.699670868	0.966407403	copeptin	pearson
hsa.mir.142.3p	-0.063542308	0.700756425	0.966407403	copeptin	pearson
hsa.mir.379.5p	-0.082101816	0.634052403	0.935411011	copeptin	pearson
hsa.mir.151a.5p	-0.174045189	0.282788045	0.893634813	copeptin	pearson
hsa.mir.20b.5p	0.037182649	0.819813121	0.977919937	copeptin	pearson
hsa.mir.7.1.5p	-0.014860801	0.927482635	0.97904585	copeptin	pearson
hsa.let.7c.5p	-0.296820261	0.093464419	0.893634813	copeptin	pearson
hsa.mir.660.5p	0.040985992	0.804340417	0.968587664	copeptin	pearson
hsa.mir.194.1.5p	0.05150348	0.752293188	0.966407403	copeptin	pearson
hsa.mir.15b.5p	-0.077214395	0.635807044	0.935411011	copeptin	pearson
hsa.mir.28.5p	-0.093579168	0.565736458	0.935167048	copeptin	pearson
hsa.mir.142.5p	-0.017858804	0.912904272	0.97904585	copeptin	pearson
hsa.mir.28.3p	-0.112726658	0.488591447	0.935167048	copeptin	pearson
hsa.mir.223.5p	-0.034495991	0.851323055	0.97904585	copeptin	pearson
hsa.mir.100.5p	0.14132886	0.390780901	0.935167048	copeptin	pearson
hsa.mir.382.5p	-0.070148527	0.693419088	0.966407403	copeptin	pearson
hsa.mir.29a.3p	0.022893801	0.888486192	0.97904585	copeptin	pearson
hsa.mir.155.5p	-0.05255741	0.750665624	0.966407403	copeptin	pearson
hsa.mir.152.3p	-0.299105073	0.06435144	0.893634813	copeptin	pearson
hsa.mir.30e.3p	-0.20107608	0.219654326	0.893634813	copeptin	pearson
hsa.mir.106b.5p	0.129057529	0.439980614	0.935167048	copeptin	pearson
hsa.mir.1.1.3p	0.212400422	0.194234192	0.893634813	copeptin	pearson
hsa.mir.139.5p	-0.235793445	0.142990819	0.893634813	copeptin	pearson
hsa.mir.652.3p	-0.267873259	0.094691203	0.893634813	copeptin	pearson
hsa.mir.98.5p	-0.158584466	0.328383466	0.893634813	copeptin	pearson
hsa.mir.181b.1.5p	-0.095268772	0.558709816	0.935167048	copeptin	pearson
hsa.mir.629.5p	0.082521854	0.612695891	0.935411011	copeptin	pearson
hsa.mir.374b.5p	-0.263267668	0.100724298	0.893634813	copeptin	pearson
hsa.mir.144.3p	0.092009838	0.572299172	0.935167048	copeptin	pearson
hsa.mir.19b.1.3p	-0.015745149	0.923179679	0.97904585	copeptin	pearson
hsa.mir.223.3p	-0.097439321	0.549743235	0.935167048	copeptin	pearson
hsa.mir.363.3p	0.154461683	0.341273647	0.893634813	copeptin	pearson
hsa.mir.361.5p	-0.166675183	0.303981787	0.893634813	copeptin	pearson
hsa.mir.107	0.041878821	0.797504129	0.968587664	copeptin	pearson
hsa.mir.99b.5p	-0.006900671	0.966291281	0.97958496	copeptin	pearson
hsa.mir.125a.5p	0.022242264	0.8916407	0.97904585	copeptin	pearson
hsa.mir.3615	0.083181182	0.609849921	0.935411011	copeptin	pearson
hsa.mir.425.5p	-0.242480028	0.131670013	0.893634813	copeptin	pearson
hsa.mir.23a.3p	-0.096787998	0.5524267	0.935167048	copeptin	pearson
hsa.mir.192.5p	0.017414238	0.915064399	0.97904585	copeptin	pearson
hsa.mir.140.5p	-0.33612881	0.033956408	0.893634813	copeptin	pearson
hsa.mir.106b.3p	0.106725247	0.512170411	0.935167048	copeptin	pearson
hsa.mir.1307.3p	-0.166040131	0.305854155	0.893634813	copeptin	pearson
hsa.mir.150.5p	0.15825866	0.329390946	0.893634813	copeptin	pearson
hsa.mir.340.5p	-0.081176718	0.618519521	0.935411011	copeptin	pearson
hsa.mir.30a.5p	-0.029245447	0.857829809	0.97904585	copeptin	pearson

hsa.mir.378a.3p	0.130312512	0.422865147	0.935167048	copeptin	pearson
hsa.mir.584.5p	-0.206128792	0.201923955	0.893634813	copeptin	pearson
hsa.mir.744.5p	-0.152403964	0.347821933	0.893634813	copeptin	pearson
hsa.let.7d.5p	-0.112948097	0.487732321	0.935167048	copeptin	pearson
hsa.mir.221.3p	-0.156062726	0.336231417	0.893634813	copeptin	pearson
hsa.mir.140.3p	-0.01226112	0.944268552	0.97904585	copeptin	pearson
hsa.mir.10b.5p	0.158667337	0.328127513	0.893634813	copeptin	pearson
hsa.mir.10a.5p	-0.111196865	0.494547991	0.935167048	copeptin	pearson
hsa.mir.30c.1.5p	-0.198664801	0.219102557	0.893634813	copeptin	pearson
hsa.mir.199a.1.5p	-0.170948055	0.291574223	0.893634813	copeptin	pearson
hsa.mir.328.3p	-0.195801568	0.232242569	0.893634813	copeptin	pearson
hsa.mir.186.5p	-0.121008425	0.457000015	0.935167048	copeptin	pearson
hsa.mir.148b.3p	-0.154567492	0.340938992	0.893634813	copeptin	pearson
hsa.let.7d.3p	-0.189848142	0.240659952	0.893634813	copeptin	pearson
hsa.mir.146b.5p	-0.097724134	0.548571742	0.935167048	copeptin	pearson
hsa.mir.143.3p	0.153503648	0.344312923	0.893634813	copeptin	pearson
hsa.mir.199a.1.3p	-0.223777645	0.165109306	0.893634813	copeptin	pearson
hsa.mir.222.3p	0.01740854	0.915092093	0.97904585	copeptin	pearson
hsa.mir.27b.3p	-0.044957293	0.782964482	0.968587664	copeptin	pearson
hsa.mir.16.1.5p	0.044849312	0.783473278	0.968587664	copeptin	pearson
hsa.mir.532.5p	0.074999186	0.645556396	0.935411011	copeptin	pearson
hsa.mir.17.5p	-0.201391459	0.212714066	0.893634813	copeptin	pearson
hsa.mir.484	-0.236807969	0.141228538	0.893634813	copeptin	pearson
hsa.mir.15a.5p	0.096821843	0.552287108	0.935167048	copeptin	pearson
hsa.mir.181a.1.5p	-0.297179245	0.062568327	0.893634813	copeptin	pearson
hsa.mir.101.1.3p	0.021524275	0.895118848	0.97904585	copeptin	pearson
hsa.mir.99a.5p	0.052507464	0.747620895	0.966407403	copeptin	pearson
hsa.mir.25.3p	-0.000721211	0.996476	0.996476	copeptin	pearson
hsa.mir.93.5p	-0.140930158	0.385724238	0.935167048	copeptin	pearson
hsa.mir.122.5p	0.051825486	0.750793697	0.966407403	copeptin	pearson
hsa.mir.423.5p	-0.333685129	0.035359335	0.893634813	copeptin	pearson
hsa.mir.320a	-0.109966738	0.49936461	0.935167048	copeptin	pearson
hsa.mir.185.5p	-0.055579254	0.733381366	0.966407403	copeptin	pearson
hsa.mir.126.5p	-0.254723114	0.112690296	0.893634813	copeptin	pearson
hsa.mir.27a.3p	0.100370067	0.537745398	0.935167048	copeptin	pearson
hsa.mir.146a.5p	-0.195407245	0.226906762	0.893634813	copeptin	pearson
hsa.let.7a.1.5p	-0.163777473	0.312584834	0.893634813	copeptin	pearson
hsa.mir.24.1.3p	-0.182918489	0.258578446	0.893634813	copeptin	pearson
hsa.mir.128.1.3p	-0.190811051	0.238238258	0.893634813	copeptin	pearson
hsa.mir.26b.5p	-0.015546656	0.9241453	0.97904585	copeptin	pearson
hsa.mir.20a.5p	-0.085665174	0.599178887	0.935167048	copeptin	pearson
hsa.let.7b.5p	0.062246983	0.702779482	0.966407403	copeptin	pearson
hsa.mir.151a.3p	-0.202458414	0.210249723	0.893634813	copeptin	pearson
hsa.mir.103a.1.3p	-0.237650521	0.139777201	0.893634813	copeptin	pearson
hsa.mir.423.3p	-0.284631182	0.075058469	0.893634813	copeptin	pearson
hsa.mir.486.1.5p	0.106834873	0.511734654	0.935167048	copeptin	pearson
hsa.mir.148a.3p	-0.010293977	0.9497331	0.97904585	copeptin	pearson
hsa.let.7f.1.5p	-0.086957178	0.593660856	0.935167048	copeptin	pearson
hsa.mir.191.5p	-0.279054117	0.081201761	0.893634813	copeptin	pearson
hsa.mir.30e.5p	-0.193677323	0.231127604	0.893634813	copeptin	pearson
hsa.mir.486.2.5p	0.006954463	0.966028669	0.97958496	copeptin	pearson
hsa.mir.92a.1.3p	0.049117273	0.763432747	0.968587664	copeptin	pearson
hsa.let.7i.5p	-0.124260987	0.444901344	0.935167048	copeptin	pearson
hsa.mir.30d.5p	-0.324764528	0.040889128	0.893634813	copeptin	pearson
hsa.mir.22.3p	-0.22537538	0.162033712	0.893634813	copeptin	pearson
hsa.mir.126.3p	-0.114961646	0.479956296	0.935167048	copeptin	pearson

hsa.mir.21.5p	-0.058389572	0.720430464	0.966407403	copeptin	pearson
hsa.let.7g.5p	-0.265814994	0.097352124	0.893634813	copeptin	pearson
hsa.mir.451a	0.191993998	0.235285849	0.893634813	copeptin	pearson
hsa.mir.26a.1.5p	-0.14239545	0.380753475	0.935167048	copeptin	pearson
hsa.mir.369.5p	-0.008700254	0.960438794	0.979778435	cpeptide	pearson
hsa.mir.411.3p	-0.027548382	0.88304723	0.979778435	cpeptide	pearson
hsa.mir.29b.1.3p	0.154466707	0.398605645	0.977281128	cpeptide	pearson
hsa.mir.29b.2.3p	0.131674717	0.480132252	0.977281128	cpeptide	pearson
hsa.mir.941.1	-0.089152022	0.639433041	0.977281128	cpeptide	pearson
hsa.mir.654.5p	0.144670176	0.414317443	0.977281128	cpeptide	pearson
hsa.mir.411.5p	0.020471721	0.908511262	0.979778435	cpeptide	pearson
hsa.mir.181a.1.3p	0.164116758	0.386157774	0.977281128	cpeptide	pearson
hsa.mir.1307.5p	0.106585328	0.554947234	0.977281128	cpeptide	pearson
hsa.mir.493.3p	0.080157722	0.632372566	0.977281128	cpeptide	pearson
hsa.mir.26b.3p	0.095442194	0.609539664	0.977281128	cpeptide	pearson
hsa.mir.22.5p	0.039419739	0.833244623	0.979778435	cpeptide	pearson
hsa.mir.21.3p	0.055717321	0.769953224	0.979778435	cpeptide	pearson
hsa.mir.501.3p	0.051498989	0.779540844	0.979778435	cpeptide	pearson
hsa.mir.454.3p	0.038440212	0.83180593	0.979778435	cpeptide	pearson
hsa.mir.486.1.3p	-0.016759349	0.920449658	0.979778435	cpeptide	pearson
hsa.mir.323a.3p	-0.068374231	0.7195887	0.979778435	cpeptide	pearson
hsa.mir.144.5p	-0.088438644	0.597501162	0.977281128	cpeptide	pearson
hsa.mir.374a.5p	0.042410911	0.803180419	0.979778435	cpeptide	pearson
hsa.mir.148a.5p	0.245092116	0.162389633	0.799034712	cpeptide	pearson
hsa.mir.495.3p	-0.007168408	0.965930009	0.979778435	cpeptide	pearson
hsa.mir.493.5p	-0.005488568	0.973910803	0.979778435	cpeptide	pearson
hsa.mir.432.5p	0.088265006	0.642782462	0.977281128	cpeptide	pearson
hsa.mir.425.3p	0.151571124	0.38474963	0.977281128	cpeptide	pearson
hsa.mir.424.3p	0.158370105	0.40323122	0.977281128	cpeptide	pearson
hsa.mir.485.3p	0.126024971	0.457336402	0.977281128	cpeptide	pearson
hsa.mir.369.3p	-0.1026849	0.557213985	0.977281128	cpeptide	pearson
hsa.mir.330.3p	-0.123190251	0.467598653	0.977281128	cpeptide	pearson
hsa.mir.339.5p	0.027041308	0.870196974	0.979778435	cpeptide	pearson
hsa.mir.4433b.5p	-0.159433774	0.3323011	0.977281128	cpeptide	pearson
hsa.mir.23b.3p	-0.33651968	0.041696243	0.580272715	cpeptide	pearson
hsa.mir.1180.3p	0.094473912	0.578084396	0.977281128	cpeptide	pearson
hsa.mir.654.3p	0.033821686	0.838038835	0.979778435	cpeptide	pearson
hsa.mir.409.3p	-0.084623095	0.608512907	0.977281128	cpeptide	pearson
hsa.mir.181d.5p	-0.01544904	0.930895533	0.979778435	cpeptide	pearson
hsa.mir.19a.3p	0.021786546	0.904211814	0.979778435	cpeptide	pearson
hsa.mir.421	-0.020830575	0.908396889	0.979778435	cpeptide	pearson
hsa.mir.339.3p	0.016037929	0.926032756	0.979778435	cpeptide	pearson
hsa.mir.342.3p	-0.006632852	0.968920957	0.979778435	cpeptide	pearson
hsa.mir.183.5p	0.069115104	0.680108255	0.979778435	cpeptide	pearson
hsa.mir.340.3p	-0.050773798	0.758870861	0.979778435	cpeptide	pearson
hsa.mir.381.3p	0.204151295	0.212535703	0.825429357	cpeptide	pearson
hsa.mir.370.3p	0.085545166	0.614672071	0.977281128	cpeptide	pearson
hsa.mir.335.5p	0.035212014	0.8360888	0.979778435	cpeptide	pearson
hsa.mir.134.5p	0.043751255	0.7970882	0.979778435	cpeptide	pearson
hsa.mir.494.3p	0.213802047	0.256601085	0.892757943	cpeptide	pearson
hsa.mir.125b.1.5p	0.091036154	0.576388342	0.977281128	cpeptide	pearson
hsa.mir.130a.3p	-0.078103545	0.641149369	0.977281128	cpeptide	pearson
hsa.let.7e.5p	-0.088578392	0.591799503	0.977281128	cpeptide	pearson
hsa.mir.543	-0.269716715	0.092355795	0.684807288	cpeptide	pearson
hsa.mir.30b.5p	-0.211794913	0.201772645	0.821854432	cpeptide	pearson
hsa.mir.625.3p	-0.437978211	0.005956865	0.506656	cpeptide	pearson

hsa.mir.335.3p	-0.389501405	0.017177408	0.506656	cpeptide	pearson
hsa.mir.197.3p	0.037109662	0.824941653	0.979778435	cpeptide	pearson
hsa.mir.130b.5p	-0.271274511	0.104358531	0.684807288	cpeptide	pearson
hsa.mir.375	0.112860769	0.488071038	0.977281128	cpeptide	pearson
hsa.mir.18a.5p	0.144070915	0.381559005	0.977281128	cpeptide	pearson
hsa.mir.320b.1	0.160269332	0.343351236	0.977281128	cpeptide	pearson
hsa.mir.182.5p	0.049669661	0.760849751	0.979778435	cpeptide	pearson
hsa.mir.106a.5p	-0.031506788	0.846962513	0.979778435	cpeptide	pearson
hsa.mir.224.5p	0.025789242	0.874488673	0.979778435	cpeptide	pearson
hsa.mir.127.3p	0.013174416	0.941050888	0.979778435	cpeptide	pearson
hsa.mir.142.3p	0.212706147	0.193577935	0.821854432	cpeptide	pearson
hsa.mir.379.5p	-0.075748055	0.66060326	0.979778435	cpeptide	pearson
hsa.mir.151a.5p	-0.203902917	0.206945063	0.822852989	cpeptide	pearson
hsa.mir.20b.5p	0.026101649	0.872980566	0.979778435	cpeptide	pearson
hsa.mir.7.1.5p	0.008043103	0.960714701	0.979778435	cpeptide	pearson
hsa.let.7c.5p	-0.231854045	0.194177383	0.821854432	cpeptide	pearson
hsa.mir.660.5p	-0.081861721	0.62029931	0.977281128	cpeptide	pearson
hsa.mir.194.1.5p	0.21380712	0.185259204	0.821854432	cpeptide	pearson
hsa.mir.15b.5p	-0.150666681	0.35340977	0.977281128	cpeptide	pearson
hsa.mir.28.5p	-0.091902869	0.572747762	0.977281128	cpeptide	pearson
hsa.mir.142.5p	-0.064921961	0.690628206	0.979778435	cpeptide	pearson
hsa.mir.28.3p	-0.207857652	0.198083284	0.821854432	cpeptide	pearson
hsa.mir.223.5p	-0.277054229	0.124752071	0.684807288	cpeptide	pearson
hsa.mir.100.5p	0.067490441	0.683104295	0.979778435	cpeptide	pearson
hsa.mir.382.5p	-0.124029315	0.484637266	0.977281128	cpeptide	pearson
hsa.mir.29a.3p	0.117397582	0.470636538	0.977281128	cpeptide	pearson
hsa.mir.155.5p	-0.089146884	0.589414014	0.977281128	cpeptide	pearson
hsa.mir.152.3p	-0.38827481	0.01458311	0.506656	cpeptide	pearson
hsa.mir.30e.3p	-0.148533942	0.366826051	0.977281128	cpeptide	pearson
hsa.mir.106b.5p	-0.009047009	0.957008819	0.979778435	cpeptide	pearson
hsa.mir.1.1.3p	0.114253091	0.488580044	0.977281128	cpeptide	pearson
hsa.mir.139.5p	-0.078867176	0.628572131	0.977281128	cpeptide	pearson
hsa.mir.652.3p	-0.119024954	0.464464164	0.977281128	cpeptide	pearson
hsa.mir.98.5p	-0.278553765	0.081771509	0.684807288	cpeptide	pearson
hsa.mir.181b.1.5p	0.087841473	0.589897035	0.977281128	cpeptide	pearson
hsa.mir.629.5p	0.095662198	0.557079518	0.977281128	cpeptide	pearson
hsa.mir.374b.5p	-0.06566309	0.687274916	0.979778435	cpeptide	pearson
hsa.mir.144.3p	0.082494166	0.61281553	0.977281128	cpeptide	pearson
hsa.mir.19b.1.3p	0.044017656	0.787394931	0.979778435	cpeptide	pearson
hsa.mir.223.3p	-0.175063885	0.279936186	0.926421284	cpeptide	pearson
hsa.mir.363.3p	0.008121368	0.960332729	0.979778435	cpeptide	pearson
hsa.mir.361.5p	-0.240188528	0.135471948	0.706994227	cpeptide	pearson
hsa.mir.107	-0.065712227	0.687052799	0.979778435	cpeptide	pearson
hsa.mir.99b.5p	-0.125564236	0.440103283	0.977281128	cpeptide	pearson
hsa.mir.125a.5p	-0.103629779	0.524550909	0.977281128	cpeptide	pearson
hsa.mir.3615	0.190475506	0.239080265	0.891419372	cpeptide	pearson
hsa.mir.425.5p	0.023848766	0.883865673	0.979778435	cpeptide	pearson
hsa.mir.23a.3p	-0.219612474	0.173324989	0.821854432	cpeptide	pearson
hsa.mir.192.5p	0.126376575	0.437127042	0.977281128	cpeptide	pearson
hsa.mir.140.5p	-0.050312848	0.757845399	0.979778435	cpeptide	pearson
hsa.mir.106b.3p	-0.075415844	0.643718108	0.977281128	cpeptide	pearson
hsa.mir.1307.3p	-0.145960872	0.368817169	0.977281128	cpeptide	pearson
hsa.mir.150.5p	0.298101079	0.061720162	0.684807288	cpeptide	pearson
hsa.mir.340.5p	-0.323849992	0.041493719	0.580272715	cpeptide	pearson
hsa.mir.30a.5p	-0.0256544	0.875139738	0.979778435	cpeptide	pearson
hsa.mir.378a.3p	0.187553178	0.246498664	0.892757943	cpeptide	pearson

hsa.mir.584.5p	-0.253577725	0.114372757	0.684807288	cpeptide	pearson
hsa.mir.744.5p	-0.280472959	0.079602944	0.684807288	cpeptide	pearson
hsa.let.7d.5p	-0.266219915	0.096824161	0.684807288	cpeptide	pearson
hsa.mir.221.3p	-0.266502787	0.09645664	0.684807288	cpeptide	pearson
hsa.mir.140.3p	-0.038403353	0.826629218	0.979778435	cpeptide	pearson
hsa.mir.10b.5p	0.091729699	0.573474311	0.977281128	cpeptide	pearson
hsa.mir.10a.5p	-0.245288112	0.127119916	0.684807288	cpeptide	pearson
hsa.mir.30c.1.5p	-0.114546646	0.481553633	0.977281128	cpeptide	pearson
hsa.mir.199a.1.5p	-0.076193076	0.640294571	0.977281128	cpeptide	pearson
hsa.mir.328.3p	-0.179376073	0.274548006	0.926421284	cpeptide	pearson
hsa.mir.186.5p	-0.070538534	0.665365171	0.979778435	cpeptide	pearson
hsa.mir.148b.3p	-0.309024368	0.052349094	0.624449902	cpeptide	pearson
hsa.let.7d.3p	-0.255223442	0.111961269	0.684807288	cpeptide	pearson
hsa.mir.146b.5p	-0.371678987	0.01820321	0.506656	cpeptide	pearson
hsa.mir.143.3p	-0.162716676	0.315772354	0.977281128	cpeptide	pearson
hsa.mir.199a.1.3p	-0.269370742	0.092790677	0.684807288	cpeptide	pearson
hsa.mir.222.3p	-3.27E-05	0.99984003	0.99984003	cpeptide	pearson
hsa.mir.27b.3p	-0.116424891	0.474346454	0.977281128	cpeptide	pearson
hsa.mir.16.1.5p	-0.005340088	0.973911498	0.979778435	cpeptide	pearson
hsa.mir.532.5p	0.057836	0.72297556	0.979778435	cpeptide	pearson
hsa.mir.17.5p	-0.02965921	0.855839397	0.979778435	cpeptide	pearson
hsa.mir.484	-0.119510681	0.462630291	0.977281128	cpeptide	pearson
hsa.mir.15a.5p	0.041001119	0.801662144	0.979778435	cpeptide	pearson
hsa.mir.181a.1.5p	-0.248940192	0.121379245	0.684807288	cpeptide	pearson
hsa.mir.101.1.3p	-0.013911057	0.932106053	0.979778435	cpeptide	pearson
hsa.mir.99a.5p	0.03900151	0.811155047	0.979778435	cpeptide	pearson
hsa.mir.25.3p	-0.075865669	0.641735839	0.977281128	cpeptide	pearson
hsa.mir.93.5p	0.033110875	0.839270559	0.979778435	cpeptide	pearson
hsa.mir.122.5p	0.154186289	0.342145614	0.977281128	cpeptide	pearson
hsa.mir.423.5p	-0.080559943	0.621197522	0.977281128	cpeptide	pearson
hsa.mir.320a	-0.098727297	0.544454974	0.977281128	cpeptide	pearson
hsa.mir.185.5p	-0.076529016	0.638817097	0.977281128	cpeptide	pearson
hsa.mir.126.5p	-0.123434064	0.447960541	0.977281128	cpeptide	pearson
hsa.mir.27a.3p	0.093573954	0.565758207	0.977281128	cpeptide	pearson
hsa.mir.146a.5p	-0.184917732	0.253320232	0.892757943	cpeptide	pearson
hsa.let.7a.1.5p	-0.344607445	0.029438401	0.580272715	cpeptide	pearson
hsa.mir.24.1.3p	-0.111952234	0.491602167	0.977281128	cpeptide	pearson
hsa.mir.128.1.3p	-0.340448718	0.031588165	0.580272715	cpeptide	pearson
hsa.mir.26b.5p	-0.030243897	0.853028261	0.979778435	cpeptide	pearson
hsa.mir.20a.5p	-0.059358596	0.715982452	0.979778435	cpeptide	pearson
hsa.let.7b.5p	-0.027595107	0.865777299	0.979778435	cpeptide	pearson
hsa.mir.151a.3p	-0.388368044	0.013270095	0.506656	cpeptide	pearson
hsa.mir.103a.1.3p	-0.253231275	0.114885376	0.684807288	cpeptide	pearson
hsa.mir.423.3p	-0.254619414	0.112841846	0.684807288	cpeptide	pearson
hsa.mir.486.1.5p	-0.025204017	0.877314941	0.979778435	cpeptide	pearson
hsa.mir.148a.3p	0.087173412	0.592739526	0.977281128	cpeptide	pearson
hsa.let.7f.1.5p	-0.362784929	0.021408684	0.510750025	cpeptide	pearson
hsa.mir.191.5p	-0.245943901	0.126074428	0.684807288	cpeptide	pearson
hsa.mir.30e.5p	-0.173998545	0.282919075	0.926421284	cpeptide	pearson
hsa.mir.486.2.5p	-0.050592082	0.756542175	0.979778435	cpeptide	pearson
hsa.mir.92a.1.3p	0.1900294	0.240202825	0.891419372	cpeptide	pearson
hsa.let.7i.5p	-0.392478088	0.012246692	0.506656	cpeptide	pearson
hsa.mir.30d.5p	-0.324675817	0.040947458	0.580272715	cpeptide	pearson
hsa.mir.22.3p	0.104263529	0.522004266	0.977281128	cpeptide	pearson
hsa.mir.126.3p	-0.225039017	0.162677726	0.799034712	cpeptide	pearson
hsa.mir.21.5p	-0.26142504	0.103218747	0.684807288	cpeptide	pearson

hsa.let.7g.5p	-0.213739743	0.185401052	0.821854432	cpeptide	pearson
hsa.mir.451a	0.017531417	0.914494975	0.979778435	cpeptide	pearson
hsa.mir.26a.1.5p	-0.317606389	0.045818051	0.58858573	cpeptide	pearson
hsa.mir.369.5p	-0.045746778	0.794131728	0.961014482	norepi	pearson
hsa.mir.411.3p	-0.045360928	0.808544	0.964477486	norepi	pearson
hsa.mir.29b.1.3p	-0.269570833	0.135690936	0.587528341	norepi	pearson
hsa.mir.29b.2.3p	-0.192878757	0.298537792	0.772034446	norepi	pearson
hsa.mir.941.1	-0.17278531	0.361208584	0.781903033	norepi	pearson
hsa.mir.654.5p	0.006599585	0.970450869	0.987559367	norepi	pearson
hsa.mir.411.5p	-0.169517271	0.337843153	0.781903033	norepi	pearson
hsa.mir.181a.1.3p	-0.020455216	0.914560368	0.983741977	norepi	pearson
hsa.mir.1307.5p	0.013057242	0.942507283	0.983741977	norepi	pearson
hsa.mir.493.3p	0.098619959	0.555817606	0.915572391	norepi	pearson
hsa.mir.26b.3p	-0.033684835	0.857236703	0.97497815	norepi	pearson
hsa.mir.22.5p	0.255856774	0.164753652	0.681957551	norepi	pearson
hsa.mir.21.3p	0.247171864	0.187894843	0.703474308	norepi	pearson
hsa.mir.501.3p	-0.056723391	0.757809971	0.946599184	norepi	pearson
hsa.mir.454.3p	0.058180701	0.747749553	0.946599184	norepi	pearson
hsa.mir.486.1.3p	-0.029975333	0.8582143	0.97497815	norepi	pearson
hsa.mir.323a.3p	0.081097099	0.670096323	0.918867489	norepi	pearson
hsa.mir.144.5p	0.119246285	0.475797206	0.856823132	norepi	pearson
hsa.mir.374a.5p	0.216457165	0.198169063	0.703474308	norepi	pearson
hsa.mir.148a.5p	-0.222929753	0.205051285	0.703474308	norepi	pearson
hsa.mir.495.3p	-0.097778154	0.559211879	0.915572391	norepi	pearson
hsa.mir.493.5p	0.079898453	0.633477633	0.918867489	norepi	pearson
hsa.mir.432.5p	0.015078857	0.936964828	0.983741977	norepi	pearson
hsa.mir.425.3p	0.128960571	0.460317276	0.853770722	norepi	pearson
hsa.mir.424.3p	0.332882247	0.072263379	0.587528341	norepi	pearson
hsa.mir.485.3p	-0.228335061	0.174060154	0.692096329	norepi	pearson
hsa.mir.369.3p	0.004881337	0.977797966	0.987559367	norepi	pearson
hsa.mir.330.3p	-0.022206883	0.896203388	0.983741977	norepi	pearson
hsa.mir.339.5p	0.225537757	0.167426704	0.681957551	norepi	pearson
hsa.mir.4433b.5p	0.01958906	0.905778703	0.983741977	norepi	pearson
hsa.mir.23b.3p	0.078646328	0.64358995	0.918867489	norepi	pearson
hsa.mir.1180.3p	-0.138501452	0.413638928	0.803229082	norepi	pearson
hsa.mir.654.3p	-0.049459576	0.764933492	0.946599184	norepi	pearson
hsa.mir.409.3p	-0.038305984	0.816908912	0.966585229	norepi	pearson
hsa.mir.181d.5p	-0.137236783	0.438953197	0.842588321	norepi	pearson
hsa.mir.19a.3p	-0.035270342	0.845502001	0.97497815	norepi	pearson
hsa.mir.421	0.035749309	0.843429578	0.97497815	norepi	pearson
hsa.mir.339.3p	0.177451264	0.300492449	0.772034446	norepi	pearson
hsa.mir.342.3p	-0.210716666	0.21062105	0.703474308	norepi	pearson
hsa.mir.183.5p	-0.045743419	0.785080406	0.956995824	norepi	pearson
hsa.mir.340.3p	0.257175288	0.113987765	0.587528341	norepi	pearson
hsa.mir.381.3p	-0.12302098	0.455600425	0.853770722	norepi	pearson
hsa.mir.370.3p	-0.084844729	0.617583061	0.918867489	norepi	pearson
hsa.mir.335.5p	0.015682361	0.926599897	0.983741977	norepi	pearson
hsa.mir.134.5p	-0.096542293	0.569750738	0.916595269	norepi	pearson
hsa.mir.494.3p	-0.076515784	0.687775067	0.918867489	norepi	pearson
hsa.mir.125b.1.5p	-0.108804304	0.503938053	0.85875158	norepi	pearson
hsa.mir.130a.3p	-0.153427964	0.35775017	0.781903033	norepi	pearson
hsa.let.7e.5p	0.26279353	0.106029051	0.587528341	norepi	pearson
hsa.mir.543	0.092277816	0.571176074	0.916595269	norepi	pearson
hsa.mir.30b.5p	0.092271607	0.581649274	0.916595269	norepi	pearson
hsa.mir.625.3p	0.14114327	0.397974597	0.781903033	norepi	pearson
hsa.mir.335.3p	0.123842024	0.465228358	0.853770722	norepi	pearson

hsa.mir.197.3p	0.030403035	0.856212204	0.97497815	norepi	pearson
hsa.mir.130b.5p	0.144606852	0.393149319	0.781903033	norepi	pearson
hsa.mir.375	0.00880274	0.957007669	0.987559367	norepi	pearson
hsa.mir.18a.5p	0.079546072	0.630255984	0.918867489	norepi	pearson
hsa.mir.320b.1	0.041582596	0.806951083	0.964477486	norepi	pearson
hsa.mir.182.5p	-0.016130946	0.921303186	0.983741977	norepi	pearson
hsa.mir.106a.5p	-0.113301512	0.486362782	0.85875158	norepi	pearson
hsa.mir.224.5p	-0.068610296	0.673998959	0.918867489	norepi	pearson
hsa.mir.127.3p	0.110790887	0.532773662	0.889732016	norepi	pearson
hsa.mir.142.3p	-0.150344096	0.360948843	0.781903033	norepi	pearson
hsa.mir.379.5p	-0.003974519	0.981645837	0.987559367	norepi	pearson
hsa.mir.151a.5p	0.208874052	0.195849438	0.703474308	norepi	pearson
hsa.mir.20b.5p	-0.080959733	0.619461102	0.918867489	norepi	pearson
hsa.mir.7.1.5p	-0.079269929	0.626814259	0.918867489	norepi	pearson
hsa.let.7c.5p	0.040742894	0.821886842	0.966585229	norepi	pearson
hsa.mir.660.5p	-0.140360203	0.394069381	0.781903033	norepi	pearson
hsa.mir.194.1.5p	-0.275631808	0.085161121	0.587528341	norepi	pearson
hsa.mir.15b.5p	0.158601489	0.328330879	0.781903033	norepi	pearson
hsa.mir.28.5p	0.185149359	0.252715684	0.742626021	norepi	pearson
hsa.mir.142.5p	0.103718456	0.524194202	0.884246785	norepi	pearson
hsa.mir.28.3p	0.142139983	0.381617375	0.781903033	norepi	pearson
hsa.mir.223.5p	0.138746402	0.448860782	0.851815348	norepi	pearson
hsa.mir.100.5p	-0.317472497	0.048908346	0.548838148	norepi	pearson
hsa.mir.382.5p	0.206802775	0.240598853	0.742626021	norepi	pearson
hsa.mir.29a.3p	-0.073856644	0.650607912	0.918867489	norepi	pearson
hsa.mir.155.5p	0.111770782	0.498135874	0.85875158	norepi	pearson
hsa.mir.152.3p	0.327806617	0.041627738	0.534756328	norepi	pearson
hsa.mir.30e.3p	0.06427917	0.697449795	0.919906069	norepi	pearson
hsa.mir.106b.5p	-0.081029928	0.628660779	0.918867489	norepi	pearson
hsa.mir.1.1.3p	-0.437591675	0.005338727	0.534756328	norepi	pearson
hsa.mir.139.5p	0.259113084	0.106414897	0.587528341	norepi	pearson
hsa.mir.652.3p	0.153022453	0.345845704	0.781903033	norepi	pearson
hsa.mir.98.5p	0.327113201	0.03936921	0.534756328	norepi	pearson
hsa.mir.181b.1.5p	-0.066329868	0.684263064	0.918867489	norepi	pearson
hsa.mir.629.5p	-0.07402521	0.649861656	0.918867489	norepi	pearson
hsa.mir.374b.5p	0.166049832	0.305825498	0.773831185	norepi	pearson
hsa.mir.144.3p	-0.067691003	0.67812978	0.918867489	norepi	pearson
hsa.mir.19b.1.3p	-0.062952083	0.699569286	0.919906069	norepi	pearson
hsa.mir.223.3p	0.184859964	0.253471157	0.742626021	norepi	pearson
hsa.mir.363.3p	-0.145293827	0.371033136	0.781903033	norepi	pearson
hsa.mir.361.5p	0.212843723	0.187294765	0.703474308	norepi	pearson
hsa.mir.107	0.013564259	0.933794844	0.983741977	norepi	pearson
hsa.mir.99b.5p	0.257988696	0.107996257	0.587528341	norepi	pearson
hsa.mir.125a.5p	-0.021224252	0.89657282	0.983741977	norepi	pearson
hsa.mir.3615	-0.140018177	0.388837094	0.781903033	norepi	pearson
hsa.mir.425.5p	0.15397691	0.342809477	0.781903033	norepi	pearson
hsa.mir.23a.3p	0.07007524	0.667435763	0.918867489	norepi	pearson
hsa.mir.192.5p	-0.176745644	0.27526926	0.751263822	norepi	pearson
hsa.mir.140.5p	0.178951924	0.269224512	0.749341557	norepi	pearson
hsa.mir.106b.3p	-0.08280265	0.611483156	0.918867489	norepi	pearson
hsa.mir.1307.3p	0.151734775	0.349967908	0.781903033	norepi	pearson
hsa.mir.150.5p	-0.362718678	0.021434224	0.534756328	norepi	pearson
hsa.mir.340.5p	0.273760743	0.087388261	0.587528341	norepi	pearson
hsa.mir.30a.5p	-0.175431265	0.278912317	0.751263822	norepi	pearson
hsa.mir.378a.3p	-0.187340435	0.247044695	0.742626021	norepi	pearson
hsa.mir.584.5p	0.376990624	0.01648877	0.534756328	norepi	pearson

hsa.mir.744.5p	0.259139195	0.106378385	0.587528341	norepi	pearson
hsa.let.7d.5p	0.346289576	0.028603877	0.534756328	norepi	pearson
hsa.mir.221.3p	0.249373642	0.120711047	0.587528341	norepi	pearson
hsa.mir.140.3p	-0.004498797	0.979537511	0.987559367	norepi	pearson
hsa.mir.10b.5p	-0.138738124	0.393230967	0.781903033	norepi	pearson
hsa.mir.10a.5p	0.109584469	0.500866259	0.85875158	norepi	pearson
hsa.mir.30c.1.5p	0.193983625	0.230376382	0.742626021	norepi	pearson
hsa.mir.199a.1.5p	0.148730987	0.35969954	0.781903033	norepi	pearson
hsa.mir.328.3p	0.183026924	0.264735724	0.749336711	norepi	pearson
hsa.mir.186.5p	0.007922272	0.961304434	0.987559367	norepi	pearson
hsa.mir.148b.3p	0.202865103	0.209315636	0.703474308	norepi	pearson
hsa.let.7d.3p	0.330059812	0.037527778	0.534756328	norepi	pearson
hsa.mir.146b.5p	0.246222234	0.125632646	0.587528341	norepi	pearson
hsa.mir.143.3p	-0.280096697	0.080024521	0.587528341	norepi	pearson
hsa.mir.199a.1.3p	0.287462705	0.072082125	0.587528341	norepi	pearson
hsa.mir.222.3p	-0.089754491	0.581791009	0.916595269	norepi	pearson
hsa.mir.27b.3p	0.049029498	0.763843423	0.946599184	norepi	pearson
hsa.mir.16.1.5p	-0.081351744	0.617760448	0.918867489	norepi	pearson
hsa.mir.532.5p	-0.160036426	0.323916975	0.781903033	norepi	pearson
hsa.mir.17.5p	0.050803674	0.755555087	0.946599184	norepi	pearson
hsa.mir.484	0.115691714	0.477153002	0.856823132	norepi	pearson
hsa.mir.15a.5p	-0.18306211	0.258198305	0.74343305	norepi	pearson
hsa.mir.181a.1.5p	0.253545238	0.114420752	0.587528341	norepi	pearson
hsa.mir.101.1.3p	-0.067341219	0.679703975	0.918867489	norepi	pearson
hsa.mir.99a.5p	-0.239158481	0.137207217	0.587528341	norepi	pearson
hsa.mir.25.3p	-0.048736464	0.765214909	0.946599184	norepi	pearson
hsa.mir.93.5p	0.014542086	0.929033907	0.983741977	norepi	pearson
hsa.mir.122.5p	-0.289221522	0.070280581	0.587528341	norepi	pearson
hsa.mir.423.5p	0.31292087	0.04929684	0.548838148	norepi	pearson
hsa.mir.320a	0.021164047	0.896864626	0.983741977	norepi	pearson
hsa.mir.185.5p	0.044994372	0.782789789	0.956995824	norepi	pearson
hsa.mir.126.5p	0.250053759	0.119668165	0.587528341	norepi	pearson
hsa.mir.27a.3p	-0.144123443	0.374940338	0.781903033	norepi	pearson
hsa.mir.146a.5p	0.285399907	0.074241066	0.587528341	norepi	pearson
hsa.let.7a.1.5p	0.353650282	0.025178717	0.534756328	norepi	pearson
hsa.mir.24.1.3p	0.168038913	0.299985758	0.772034446	norepi	pearson
hsa.mir.128.1.3p	0.25357243	0.114380579	0.587528341	norepi	pearson
hsa.mir.26b.5p	-0.054759857	0.737171366	0.946599184	norepi	pearson
hsa.mir.20a.5p	0.021645113	0.894533335	0.983741977	norepi	pearson
hsa.let.7b.5p	-0.078612817	0.629683363	0.918867489	norepi	pearson
hsa.mir.151a.3p	0.251405491	0.117615572	0.587528341	norepi	pearson
hsa.mir.103a.1.3p	0.342089526	0.030725046	0.534756328	norepi	pearson
hsa.mir.423.3p	0.351177953	0.02628898	0.534756328	norepi	pearson
hsa.mir.486.1.5p	-0.054469093	0.738517734	0.946599184	norepi	pearson
hsa.mir.148a.3p	-0.244318159	0.12867811	0.587528341	norepi	pearson
hsa.let.7f.1.5p	0.296641652	0.063067212	0.587528341	norepi	pearson
hsa.mir.191.5p	0.345832342	0.028828757	0.534756328	norepi	pearson
hsa.mir.30e.5p	0.109300472	0.501983354	0.85875158	norepi	pearson
hsa.mir.486.2.5p	0.001767631	0.991363089	0.991363089	norepi	pearson
hsa.mir.92a.1.3p	-0.20446274	0.205674112	0.703474308	norepi	pearson
hsa.let.7i.5p	0.326088889	0.040026323	0.534756328	norepi	pearson
hsa.mir.30d.5p	0.37972779	0.015659803	0.534756328	norepi	pearson
hsa.mir.22.3p	-0.022961024	0.888160817	0.983741977	norepi	pearson
hsa.mir.126.3p	0.186015214	0.250464345	0.742626021	norepi	pearson
hsa.mir.21.5p	0.185668217	0.251364965	0.742626021	norepi	pearson
hsa.let.7g.5p	0.270506512	0.091368922	0.587528341	norepi	pearson

hsa.mir.451a	-0.156884151	0.333662427	0.781903033	norepi	pearson
hsa.mir.26a.1.5p	0.239466769	0.136686142	0.587528341	norepi	pearson
