# Supplementary material for: Effects of a short‐term cold exposure on circulating microRNAs and metabolic parameters in healthy adult subjects
Source: J Cell Mol Med. 2021 Dec 17;26(2):548–62. doi: 10.1111/jcmm.17121 (PMC8743656; doi:10.1111/jcmm.17121)
Supplement: Supplementary file 4 — Table S3 [file JCMM-26-548-s004.pdf]

miRNA-miRNA and miRNA-analyte correlation analysis using GeneNet. Col 1 and 2, interacting molecule pairs; col 3, pvalue of interaction; col 4, qvalue of interaction; col 5, probability of non-zero correlation; col 6, magnitude of partial correlation.

node1_name	node2_name	pval	qval	prob	pcor
copeptin	norepi	2.22E-16	1.72E-13	1	-0.297419208
mir-375	copeptin	2.22E-16	1.72E-13	1	-0.230443519
mir-143-3p	norepi	3.60E-11	1.39E-08	0.999999839	-0.184491815
mir-584-5p	mir-150-5p	1.34E-10	4.39E-08	0.999998393	-0.179073524
mir-10b-5p	mir-148a-3p	3.93E-10	1.22E-07	0.999991047	-0.174531652
mir-143-3p	mir-139-5p	6.87E-09	1.74E-06	0.999991047	-0.161801616
mir-543	mir-23a-3p	1.32E-08	2.83E-06	0.999991047	-0.158772285
mir-10a-5p	cpeptide	1.91E-08	3.60E-06	0.99995857	-0.15699184
mir-150-5p	norepi	7.51E-08	1.26E-05	0.999899318	-0.150335172
mir-375	mir-15b-5p	2.93E-07	3.87E-05	0.999798078	-0.14340744
mir-532-5p	mir-15b-5p	3.58E-07	4.53E-05	0.999708645	-0.142358135
mir-143-3p	cpeptide	5.66E-07	6.61E-05	0.999659387	-0.139937294
mir-182-5p	copeptin	7.55E-07	8.43E-05	0.999460555	-0.138387135
mir-125b-1-5p	mir-28-3p	1.43E-06	0.000141185	0.999419599	-0.134892336
mir-28-3p	mir-125b-2-5p	1.43E-06	0.000141185	0.999419599	-0.134892336
mir-23a-3p	norepi	1.44E-06	0.000141698	0.999318852	-0.134865788
mir-423-5p	mir-30a-5p	1.55E-06	0.000150127	0.998742191	-0.134458433
mir-151a-5p	mir-27a-3p	2.08E-06	0.000193493	0.998742191	-0.132819394
mir-223-3p	mir-30a-5p	3.03E-06	0.000267028	0.998262818	-0.130684257
mir-140-5p	copeptin	3.60E-06	0.000308316	0.998262818	-0.129698506
mir-484	norepi	3.76E-06	0.000319524	0.99622014	-0.129447962
mir-151a-5p	mir-320a	7.22E-06	0.000569203	0.99622014	-0.125641187
mir-143-3p	mir-125a-5p	7.76E-06	0.000605349	0.99622014	-0.125208968
mir-151a-5p	mir-99a-5p	9.44E-06	0.000711559	0.99622014	-0.124041531
mir-125a-5p	mir-28-3p	9.85E-06	0.000736432	0.995593229	-0.123786608
mir-3615	mir-143-3p	2.01E-05	0.00130795	0.992577293	-0.119442451
mir-378a-3p	mir-543	2.04E-05	0.001323757	0.992577293	-0.119351742
mir-584-5p	mir-30a-5p	2.37E-05	0.001496388	0.992577293	-0.118408001
mir-146b-5p	cpeptide	4.07E-05	0.002398151	0.982543506	-0.114969917
mir-29a-3p	mir-320a	4.82E-05	0.002768282	0.981704382	-0.113881163
mir-140-5p	mir-1307-3p	5.43E-05	0.003062152	0.981704382	-0.113099463
mir-99b-5p	mir-28-3p	6.04E-05	0.003343869	0.981704382	-0.112400303
mir-191-5p	mir-30a-5p	7.66E-05	0.004041733	0.981704382	-0.110830093
mir-106b-3p	mir-28-5p	9.49E-05	0.004756046	0.981704382	-0.109395417
mir-224-5p	mir-375	9.93E-05	0.004918487	0.981704382	-0.109087318
mir-143-3p	mir-150-5p	0.000102328	0.005025586	0.981704382	-0.108887272
mir-3615	mir-148b-3p	0.000116501	0.00556712	0.973438204	-0.108006016
mir-361-5p	mir-423-5p	0.000134754	0.006234632	0.973438204	-0.107009187
mir-148b-3p	cpeptide	0.000138917	0.006380961	0.973438204	-0.106799747
mir-223-3p	mir-28-3p	0.000152913	0.00685788	0.973438204	-0.106136425
mir-151a-3p	cpeptide	0.000172998	0.007504179	0.973438204	-0.105278039
mir-543	mir-92a-1-3p	0.000183461	0.007824328	0.973438204	-0.104867364
mir-92a-2-3p	mir-543	0.000183461	0.007824328	0.973438204	-0.104867364
mir-181b-2-5p	mir-3615	0.000189607	0.008007419	0.973438204	-0.104636272
mir-3615	mir-181b-1-5p	0.000189607	0.008007419	0.973438204	-0.104636272
mir-150-5p	mir-30e-5p	0.000195512	0.008180014	0.973438204	-0.104420745
mir-139-5p	copeptin	0.000221495	0.008983487	0.964275405	-0.103539715
mir-543	let-7g-5p	0.000234181	0.009363153	0.964275405	-0.10314426
mir-182-5p	mir-425-5p	0.000250574	0.009838088	0.964275405	-0.102661974
mir-27b-3p	mir-423-5p	0.000256353	0.010001478	0.964275405	-0.102498965
mir-148b-3p	mir-375	0.000260738	0.010124076	0.964275405	-0.102377567
mir-17-5p	mir-10b-5p	0.000279683	0.010640563	0.964275405	-0.101874161
mir-10b-5p	mir-15b-5p	0.000334799	0.01203127	0.964275405	-0.100572715
mir-25-3p	cpeptide	0.000361026	0.012640295	0.964275405	-0.100022461
mir-222-3p	mir-199a-1-5p	0.000376775	0.012991184	0.960980372	-0.099709731
mir-222-3p	mir-199a-2-5p	0.000376775	0.012991184	0.960980372	-0.099709731
mir-98-5p	mir-106a-5p	0.000409208	0.013772801	0.939827895	-0.099102419
mir-629-5p	mir-15b-5p	0.000454264	0.014954794	0.91917994	-0.098329336

mir-22-3p	copeptin	0.000525321	0.016807571	0.91917994	-0.097244556
mir-150-5p	mir-28-3p	0.000530014	0.016926286	0.91917994	-0.097177811
mir-30d-5p	copeptin	0.00061104	0.018942203	0.910181536	-0.096104686
mir-15a-5p	cpeptide	0.000668731	0.020325901	0.910181536	-0.095418527
mir-10b-5p	mir-423-3p	0.000693645	0.020906913	0.910181536	-0.095139063
mir-148a-3p	mir-15b-5p	0.00071583	0.02141614	0.910181536	-0.094897958
let-7g-5p	copeptin	0.000755201	0.022316501	0.897874621	-0.094486659
mir-1307-3p	let-7g-5p	0.000864596	0.024765261	0.897874621	-0.093440276
mir-182-5p	mir-139-5p	0.000915617	0.025856671	0.897874621	-0.092993633
mir-19b-2-3p	mir-30a-5p	0.000960143	0.026784306	0.897874621	-0.092622282
mir-19b-1-3p	mir-30a-5p	0.000960143	0.026784306	0.897874621	-0.092622282
mir-17-5p	norepi	0.00106917	0.028963176	0.897874621	-0.091776237
mir-543	cpeptide	0.001124035	0.030012641	0.897874621	-0.091380245
mir-143-3p	mir-484	0.001131357	0.030150431	0.897874621	-0.091328753
mir-423-3p	mir-10a-5p	0.001326346	0.033752157	0.889132582	-0.090059655
mir-584-5p	mir-224-5p	0.00137439	0.034593262	0.889132582	-0.08977351
mir-29a-3p	let-7i-5p	0.001385343	0.034782461	0.889132582	-0.089709565
mir-148a-3p	mir-20b-5p	0.001444138	0.035782213	0.889132582	-0.089374076
mir-451a	mir-363-3p	0.001446175	0.035816391	0.889132582	0.089362676
mir-425-5p	mir-103a-1-3p	0.001438495	0.035687403	0.889132582	0.089405724
mir-103a-2-3p	mir-425-5p	0.001438495	0.035687403	0.889132582	0.089405724
mir-181b-2-5p	mir-30a-5p	0.001387986	0.034827976	0.889132582	0.089694204
mir-181b-1-5p	mir-30a-5p	0.001387986	0.034827976	0.889132582	0.089694204
mir-340-5p	mir-30c-1-5p	0.00138023	0.034694259	0.889132582	0.089739357
mir-340-5p	mir-30c-2-5p	0.00138023	0.034694259	0.889132582	0.089739357
mir-378a-3p	let-7g-5p	0.001345642	0.034092182	0.889132582	0.089943604
mir-139-5p	cpeptide	0.001309933	0.033460539	0.889132582	0.090159604
mir-15a-5p	mir-92a-1-3p	0.001289442	0.033093355	0.889132582	0.09028602
mir-92a-2-3p	mir-15a-5p	0.001289442	0.033093355	0.889132582	0.09028602
mir-10b-5p	mir-27a-3p	0.001254158	0.032452878	0.889132582	0.090508098
mir-361-5p	mir-224-5p	0.001149673	0.030492808	0.889132582	0.091201282
mir-224-5p	mir-150-5p	0.001147067	0.030444295	0.897874621	0.091219303
let-7d-5p	mir-30a-5p	0.001120285	0.029941872	0.897874621	0.091406736
mir-378a-3p	mir-99a-5p	0.001097403	0.029507018	0.897874621	0.091570175
mir-182-5p	mir-140-5p	0.00108625	0.029293135	0.897874621	0.091650991
mir-584-5p	mir-423-5p	0.001071374	0.029005918	0.897874621	0.091759974
mir-378a-3p	mir-28-5p	0.001025574	0.028107217	0.897874621	0.092104522
mir-103a-2-3p	let-7g-5p	0.001012542	0.027847453	0.897874621	0.092205159
mir-103a-1-3p	let-7g-5p	0.001012542	0.027847453	0.897874621	0.092205159
mir-23a-3p	mir-221-3p	0.000980828	0.027207598	0.897874621	0.092455165
mir-26b-5p	mir-143-3p	0.000968451	0.0269549	0.897874621	0.092554761
mir-143-3p	mir-374b-5p	0.000880412	0.025106916	0.897874621	0.093299266
mir-151a-3p	mir-28-3p	0.000859866	0.024662494	0.897874621	0.093482911
mir-27b-3p	mir-199a-1-5p	0.000859232	0.024648694	0.897874621	0.093488644
mir-27b-3p	mir-199a-2-5p	0.000859232	0.024648694	0.897874621	0.093488644
mir-126-5p	cpeptide	0.000845339	0.024345159	0.897874621	0.093615225
mir-629-5p	mir-106b-3p	0.000841021	0.024250334	0.897874621	0.09365496
mir-423-3p	mir-1307-3p	0.000738301	0.021924319	0.897874621	0.094660712
mir-29a-3p	mir-223-3p	0.000735023	0.021850657	0.910181536	0.0946949
mir-222-3p	copeptin	0.000718564	0.021478377	0.910181536	0.094868725
mir-423-5p	mir-191-5p	0.00069573	0.020955092	0.910181536	0.095116102
mir-378a-3p	mir-99b-5p	0.000686306	0.02073677	0.910181536	0.095220406
mir-151a-5p	mir-30e-5p	0.000682958	0.020658885	0.910181536	0.095257777
mir-139-5p	let-7d-3p	0.000639312	0.019627118	0.910181536	0.095761239
mir-28-5p	mir-15b-5p	0.000613663	0.019006323	0.910181536	0.096072201
mir-125a-5p	mir-543	0.000564638	0.017788635	0.910181536	0.096701771
mir-29a-3p	mir-151a-5p	0.00056416	0.017776903	0.91917994	0.096708148
mir-361-5p	mir-30a-5p	0.000548138	0.017380599	0.91917994	0.096925139
mir-223-3p	mir-23a-3p	0.000535026	0.017052569	0.91917994	0.097107144
mir-140-5p	mir-148b-3p	0.000496416	0.016066623	0.91917994	0.097668272
mir-22-3p	mir-30c-1-5p	0.000475834	0.01552845	0.91917994	0.097984245
mir-22-3p	mir-30c-2-5p	0.000475834	0.01552845	0.91917994	0.097984245
mir-543	mir-221-3p	0.000434417	0.01441797	0.91917994	0.098660636
mir-99b-5p	mir-150-5p	0.000430491	0.014318674	0.939827895	0.098727841

mir-140-5p	mir-125a-5p	0.00041635	0.013957426	0.939827895	0.098974734
mir-1307-3p	mir-122-5p	0.000395636	0.01341788	0.939827895	0.099350882
mir-17-5p	mir-93-5p	0.000390009	0.013292046	0.960980372	0.099456244
mir-148b-3p	mir-186-5p	0.000383634	0.013147997	0.960980372	0.099577327
mir-148b-3p	mir-27a-3p	0.000375307	0.01295893	0.964275405	0.099738359
mir-106a-5p	mir-378a-3p	0.000367195	0.01277903	0.964275405	0.099898467
mir-126-5p	mir-10a-5p	0.000362089	0.012664317	0.964275405	0.100000958
mir-194-2-5p	mir-30a-5p	0.000361001	0.012639729	0.964275405	0.100022968
mir-194-1-5p	mir-30a-5p	0.000361001	0.012639729	0.964275405	0.100022968
mir-98-5p	let-7d-5p	0.000334034	0.012013043	0.964275405	0.100589344
mir-27a-3p	mir-10a-5p	0.000328869	0.011889109	0.964275405	0.100702679
mir-140-5p	cpeptide	0.00031477	0.011544108	0.964275405	0.101020747
mir-543	mir-128-2-3p	0.000311812	0.011470465	0.964275405	0.101089156
mir-128-1-3p	mir-543	0.000311812	0.011470465	0.964275405	0.101089156
mir-30d-5p	mir-128-1-3p	0.000306814	0.011344976	0.964275405	0.101206166
mir-30d-5p	mir-128-2-3p	0.000306814	0.011344976	0.964275405	0.101206166
mir-148a-3p	mir-378a-3p	0.000287822	0.010856122	0.964275405	0.101667617
mir-423-5p	mir-375	0.000284732	0.01077473	0.964275405	0.101745373
mir-181a-2-5p	mir-224-5p	0.000278725	0.010614969	0.964275405	0.101898818
mir-224-5p	mir-181a-1-5p	0.000278725	0.010614969	0.964275405	0.101898818
mir-425-5p	mir-423-5p	0.000260262	0.01011083	0.964275405	0.102390647
mir-143-3p	mir-27a-3p	0.000258273	0.01005529	0.964275405	0.102445585
mir-146b-5p	mir-10a-5p	0.000252299	0.009887093	0.964275405	0.102612934
mir-361-5p	mir-151a-5p	0.000233697	0.009348889	0.964275405	0.103158951
mir-27a-3p	cpeptide	0.000225452	0.00910308	0.964275405	0.103414135
mir-199a-2-3p	mir-199a-1-3p	0.000198336	0.008261414	0.964275405	0.104319837
mir-199a-2-3p	mir-199b-3p	0.000198336	0.008261414	0.964275405	0.104319837
mir-199a-1-3p	mir-199b-3p	0.000198336	0.008261414	0.964275405	0.104319837
mir-181b-2-5p	mir-224-5p	0.000186436	0.007913423	0.973438204	0.104754584
mir-181b-1-5p	mir-224-5p	0.000186436	0.007913423	0.973438204	0.104754584
mir-22-3p	mir-151a-5p	0.000183821	0.007835145	0.973438204	0.104853637
let-7i-5p	let-7g-5p	0.000174539	0.007552009	0.973438204	0.105216122
mir-223-3p	mir-126-5p	0.000171856	0.007468556	0.973438204	0.105324287
mir-584-5p	let-7d-5p	0.000170671	0.007431455	0.973438204	0.105372577
mir-182-5p	norepi	0.000127788	0.005984957	0.973438204	0.107373683
mir-484	mir-15b-5p	0.000117561	0.00560707	0.973438204	0.107944248
mir-584-5p	mir-28-5p	0.0001127	0.00542258	0.973438204	0.108231983
mir-143-3p	mir-224-5p	0.000104182	0.005091269	0.973438204	0.108765731
mir-1307-3p	mir-28-5p	0.000103809	0.005078133	0.981704382	0.108789969
mir-224-5p	mir-543	8.98E-05	0.004565098	0.981704382	0.109765399
mir-103a-2-3p	mir-28-3p	8.94E-05	0.004546759	0.981704382	0.109801407
mir-103a-1-3p	mir-28-3p	8.94E-05	0.004546759	0.981704382	0.109801407
mir-1307-3p	norepi	8.11E-05	0.004223165	0.981704382	0.110451779
mir-17-5p	mir-140-5p	7.93E-05	0.004152568	0.981704382	0.110597732
mir-101-1-3p	mir-101-2-3p	7.74E-05	0.00407425	0.981704382	0.110761504
mir-29a-3p	mir-143-3p	7.48E-05	0.003964951	0.981704382	0.110993478
mir-363-3p	copeptin	7.27E-05	0.003880632	0.981704382	0.1111753
mir-107	mir-28-5p	7.24E-05	0.003867023	0.981704382	0.111204889
mir-1307-3p	mir-543	6.65E-05	0.003611669	0.981704382	0.1117735
mir-139-5p	mir-20b-5p	6.52E-05	0.003558211	0.981704382	0.111895973
mir-194-2-5p	mir-122-5p	4.71E-05	0.002718163	0.982543506	0.114021839
mir-122-5p	mir-194-1-5p	4.71E-05	0.002718163	0.982543506	0.114021839
mir-148a-3p	let-7g-5p	4.47E-05	0.002599533	0.982543506	0.114362796
mir-28-3p	cpeptide	3.47E-05	0.002085935	0.982543506	0.11599545
mir-223-3p	mir-15b-5p	3.13E-05	0.001904269	0.982543506	0.116650337
mir-584-5p	mir-629-5p	2.92E-05	0.001789435	0.982965503	0.117092492
mir-19b-2-3p	mir-139-5p	2.58E-05	0.001601665	0.982965503	0.117868931
mir-19b-1-3p	mir-139-5p	2.58E-05	0.001601665	0.982965503	0.117868931
mir-224-5p	mir-24-2-3p	2.56E-05	0.001593884	0.992577293	0.117907946
mir-224-5p	mir-24-1-3p	2.56E-05	0.001593884	0.992577293	0.117907946
mir-27a-3p	mir-126-5p	2.12E-05	0.001370651	0.992577293	0.119087155
mir-148a-3p	mir-148b-3p	1.62E-05	0.001095487	0.992577293	0.12074868
mir-374b-5p	cpeptide	1.61E-05	0.001087397	0.995593229	0.120809225
mir-122-5p	mir-378a-3p	1.58E-05	0.00107232	0.995593229	0.120922808

mir-29a-3p	mir-139-5p	1.54E-05	0.001054796	0.995593229	0.121056057
mir-150-5p	mir-30a-5p	1.53E-05	0.001048058	0.995593229	0.121107662
mir-425-5p	norepi	1.37E-05	0.000961075	0.995593229	0.121793644
let-7i-5p	copeptin	1.33E-05	0.000938256	0.995593229	0.12198027
mir-182-5p	mir-150-5p	1.30E-05	0.000921045	0.995593229	0.122123046
mir-148b-3p	norepi	1.20E-05	0.000863742	0.995593229	0.12261194
mir-451a	copeptin	9.51E-06	0.000715655	0.99622014	0.123999121
mir-27b-3p	mir-148a-3p	8.98E-06	0.000683383	0.99622014	0.124338094
mir-148b-3p	mir-126-5p	8.27E-06	0.000638647	0.99622014	0.124827738
mir-151a-5p	mir-28-5p	6.86E-06	0.00054509	0.99622014	0.12594141
mir-543	mir-28-3p	6.67E-06	0.000532107	0.99622014	0.126107352
let-7i-5p	mir-30d-5p	5.43E-06	0.000445048	0.99622014	0.127311922
mir-29a-3p	mir-99a-5p	3.30E-06	0.000286964	0.998262818	0.130194977
mir-223-3p	mir-28-5p	2.89E-06	0.000256803	0.998262818	0.130946174
mir-29a-3p	norepi	2.36E-06	0.000215285	0.998262818	0.132102268
mir-126-3p	mir-27a-3p	2.24E-06	0.000206419	0.998742191	0.132387064
mir-222-3p	mir-30a-5p	2.01E-06	0.000188349	0.998742191	0.132997614
mir-425-5p	mir-30a-5p	1.18E-06	0.000121434	0.999419599	0.135964968
mir-15b-5p	copeptin	1.17E-06	0.000120429	0.999419599	0.136022553
mir-1307-3p	mir-28-3p	1.01E-06	0.000107178	0.999419599	0.136815738
mir-143-3p	mir-148b-3p	8.77E-07	9.55E-05	0.999419599	0.137574775
mir-99b-5p	norepi	8.50E-07	9.31E-05	0.999460555	0.137744667
mir-10b-5p	mir-143-3p	6.17E-07	7.09E-05	0.999460555	0.139476172
mir-192-5p	mir-148a-3p	6.13E-07	7.05E-05	0.999659387	0.139505939
mir-30a-5p	mir-28-3p	4.52E-07	5.50E-05	0.999659387	0.14113032
mir-378a-3p	cpeptide	4.18E-07	5.16E-05	0.999708645	0.141536366
mir-222-3p	mir-10a-5p	3.48E-07	4.43E-05	0.999798078	0.142510287
mir-148a-3p	mir-99a-5p	2.80E-07	3.74E-05	0.999841197	0.143642366
mir-223-3p	mir-151a-5p	2.64E-07	3.57E-05	0.999841197	0.143954984
mir-182-5p	mir-30a-5p	2.48E-07	3.41E-05	0.999841197	0.144264148
mir-423-5p	cpeptide	2.38E-07	3.30E-05	0.999841197	0.144478038
mir-27b-3p	mir-148b-3p	1.93E-07	2.78E-05	0.999841197	0.145563001
mir-375	mir-10a-5p	1.39E-07	2.11E-05	0.999841197	0.147228115
mir-139-5p	mir-28-3p	1.38E-07	2.09E-05	0.999899318	0.147292975
mir-29a-3p	copeptin	1.23E-07	1.91E-05	0.999899318	0.147866205
mir-29a-3p	mir-10b-5p	1.21E-07	1.89E-05	0.999899318	0.147939162
mir-19b-2-3p	mir-19b-1-3p	9.01E-08	1.47E-05	0.999899318	0.1494278
mir-10b-5p	mir-10a-5p	7.13E-08	1.20E-05	0.999899318	0.15059406
mir-10b-5p	mir-150-5p	2.58E-08	4.71E-06	0.999899318	0.155559485
mir-24-2-3p	mir-24-1-3p	1.90E-08	3.58E-06	0.999991047	0.157032051
mir-7-3-5p	mir-7-1-5p	1.86E-08	3.54E-06	0.999991047	0.157131434
mir-7-3-5p	mir-7-2-5p	1.86E-08	3.54E-06	0.999991047	0.157131434
mir-7-1-5p	mir-7-2-5p	1.86E-08	3.54E-06	0.999991047	0.157131434
mir-224-5p	cpeptide	1.83E-08	3.50E-06	0.999991047	0.157212206
mir-375	mir-23a-3p	1.06E-08	2.43E-06	0.999991047	0.159792154
mir-10b-5p	mir-375	8.37E-09	2.04E-06	0.999991047	0.160886566
mir-143-3p	mir-28-3p	7.81E-09	1.93E-06	0.999991047	0.161208443
mir-192-5p	mir-122-5p	7.12E-09	1.79E-06	0.999991047	0.161637169
mir-29a-3p	mir-375	5.50E-09	1.45E-06	0.999991047	0.162826651
mir-125a-5p	mir-99b-5p	1.28E-10	4.22E-08	0.999999775	0.179270937
mir-92a-2-3p	mir-92a-1-3p	6.18E-11	2.26E-08	0.999999775	0.18228403
mir-29a-3p	mir-425-5p	2.36E-11	9.63E-09	0.999999901	0.186184476
mir-103a-2-3p	mir-103a-1-3p	4.08E-12	1.81E-09	0.999999901	0.193101041
mir-30a-5p	mir-10a-5p	3.88E-12	1.74E-09	0.999999991	0.193294312
mir-122-5p	mir-148a-3p	1.13E-12	5.87E-10	0.999999991	0.197975543
mir-128-1-3p	mir-128-2-3p	3.53E-13	1.99E-10	0.999999995	0.202321925
mir-122-5p	mir-99a-5p	2.98E-14	1.85E-11	0.999999998	0.211217509
copeptin	cpeptide	2.93E-14	1.82E-11	1	0.211284404
mir-199a-1-5p	mir-199a-2-5p	2.22E-16	1.72E-13	1	0.227023917
mir-194-2-5p	mir-194-1-5p	2.22E-16	1.72E-13	1	0.260885657
mir-181a-2-5p	mir-181a-1-5p	2.22E-16	1.72E-13	1	0.282061521
mir-30c-1-5p	mir-30c-2-5p	2.22E-16	1.72E-13	1	0.341583471
mir-125b-1-5p	mir-125b-2-5p	2.22E-16	1.72E-13	1	0.489262878
mir-181b-2-5p	mir-181b-1-5p	2.22E-16	1.72E-13	1	0.581962787
