# Supplementary material for: Effects of a short‐term cold exposure on circulating microRNAs and metabolic parameters in healthy adult subjects
Source: J Cell Mol Med. 2021 Dec 17;26(2):548–62. doi: 10.1111/jcmm.17121 (PMC8743656; doi:10.1111/jcmm.17121)
Supplement: Supplementary file 5 — Table S4 [file JCMM-26-548-s003.pdf]

Differential correlation analysis of miRNAs at 14°C and RT. miRNA pairs are indicated in and 6, magnitude and significance of correlation at RT; col 7 and 8, difference in significance a difference

molecule X	molecule Y	r1	p1	r2	p2
mir-27b-3p	mir-26a-2-5p	0.508945102	0.021919419	0.954662888	6.52E-11
mir-27b-3p	mir-26a-1-5p	0.508945102	0.021919419	0.954662888	6.52E-11
let-7a-3-5p	mir-23a-3p	0.618848455	0.003624706	0.961085989	1.69E-11
let-7a-2-5p	mir-23a-3p	0.618848455	0.003624706	0.961085989	1.69E-11
let-7a-1-5p	mir-23a-3p	0.618848455	0.003624706	0.961085989	1.69E-11
mir-151a-3p	mir-139-5p	0.448270567	0.047448308	0.932640116	2.12E-09
mir-25-3p	mir-20a-5p	0.983404599	8.54E-15	0.842677901	3.12E-06
mir-27b-3p	mir-423-3p	0.078132716	0.743348611	0.838801393	3.83E-06
mir-191-5p	mir-151a-3p	0.765632712	8.34E-05	0.971689012	1.00E-12
let-7f-2-5p	mir-23a-3p	0.607720767	0.004478198	0.948111871	2.14E-10
let-7f-1-5p	mir-23a-3p	0.607720767	0.004478198	0.948111871	2.14E-10
let-7d-5p	mir-23a-3p	0.444669293	0.04947889	0.918922569	1.07E-08
mir-146b-5p	mir-23a-3p	0.569530648	0.008760707	0.9403515	7.30E-10
mir-151a-5p	mir-148b-3p	0.095822513	0.687783135	0.829823163	6.02E-06
mir-27b-3p	mir-21-5p	0.581569507	0.007151639	0.941408956	6.24E-10
mir-182-5p	mir-103a-2-3p	0.267856789	0.253546132	-0.664279795	0.001400859
mir-182-5p	mir-103a-1-3p	0.267856789	0.253546132	-0.664279795	0.001400859
mir-126-3p	mir-103a-2-3p	0.507205482	0.022452238	0.92531775	5.22E-09
mir-126-3p	mir-103a-1-3p	0.507205482	0.022452238	0.92531775	5.22E-09
mir-27b-3p	let-7f-2-5p	0.463713803	0.039456732	0.909707062	2.72E-08
mir-27b-3p	let-7f-1-5p	0.463713803	0.039456732	0.909707062	2.72E-08
mir-27b-3p	mir-340-5p	0.411844979	0.071186809	0.897860945	7.89E-08
mir-126-3p	mir-139-5p	0.471080938	0.036032896	0.907894721	3.23E-08
mir-24-2-3p	mir-151a-3p	0.671280326	0.001193149	0.947992047	2.19E-10
mir-151a-3p	mir-24-1-3p	0.671280326	0.001193149	0.947992047	2.19E-10
mir-146a-5p	mir-146b-5p	0.651070917	0.001875948	0.943776916	4.34E-10
mir-340-5p	mir-148a-3p	-0.399034082	0.08135614	0.508165398	0.022156975
mir-99b-5p	mir-99a-5p	-0.064470811	0.787131047	0.718081356	0.000363096
mir-744-5p	mir-125b-1-5p	-0.523638384	0.017808445	0.359655136	0.119349379
mir-744-5p	mir-125b-2-5p	-0.523638384	0.017808445	0.359655136	0.119349379
mir-423-3p	mir-148a-3p	-0.426230738	0.060935984	0.462726258	0.03993421
mir-148a-3p	mir-30c-1-5p	-0.437016512	0.054014342	0.451825474	0.045507223
mir-148a-3p	mir-30c-2-5p	-0.437016512	0.054014342	0.451825474	0.045507223
mir-128-1-3p	mir-139-5p	0.493682039	0.026953429	0.90418098	4.55E-08
mir-139-5p	mir-128-2-3p	0.493682039	0.026953429	0.90418098	4.55E-08
mir-1307-3p	mir-28-3p	0.462270102	0.040156266	0.896333244	8.97E-08
mir-101-1-3p	mir-25-3p	0.981639053	2.11E-14	0.883985605	2.36E-07
mir-25-3p	mir-101-2-3p	0.981639053	2.11E-14	0.883985605	2.36E-07
mir-27b-3p	mir-151a-5p	-0.030402782	0.898750908	0.723686261	0.000310047
mir-98-5p	mir-23a-3p	0.543085238	0.013340639	0.914153171	1.76E-08
mir-125a-5p	mir-99a-5p	-0.274664254	0.241215384	0.575793607	0.007890385
mir-182-5p	mir-151a-3p	-0.041713245	0.861385367	-0.752764359	0.00012816
mir-182-5p	mir-27b-3p	-0.221798565	0.347308101	-0.819872936	9.66E-06
mir-125b-1-5p	mir-99b-5p	-0.20944466	0.375487346	0.609488551	0.004332482
mir-99b-5p	mir-125b-2-5p	-0.20944466	0.375487346	0.609488551	0.004332482
mir-27b-3p	let-7d-5p	0.295260991	0.206290773	0.840654534	3.47E-06
mir-199a-2-3p	mir-629-5p	-0.321250227	0.167247293	-0.848542725	2.27E-06
mir-629-5p	mir-199a-1-3p	-0.321250227	0.167247293	-0.848542725	2.27E-06
mir-629-5p	mir-199b-3p	-0.321250227	0.167247293	-0.848542725	2.27E-06
mir-125a-5p	mir-125b-1-5p	-0.324427775	0.162849516	0.523446806	0.017857754
mir-125a-5p	mir-125b-2-5p	-0.324427775	0.162849516	0.523446806	0.017857754
mir-148b-3p	mir-106b-3p	0.441520007	0.051308582	-0.413936398	0.069621459
mir-103a-2-3p	mir-629-5p	0.159628362	0.501428042	-0.636416461	0.002553957
mir-103a-1-3p	mir-629-5p	0.159628362	0.501428042	-0.636416461	0.002553957
mir-103a-2-3p	mir-92a-2-3p	-0.251767435	0.284255385	-0.822209596	8.67E-06
mir-103a-2-3p	mir-92a-1-3p	-0.251767435	0.284255385	-0.822209596	8.67E-06
mir-92a-2-3p	mir-103a-1-3p	-0.251767435	0.284255385	-0.822209596	8.67E-06
mir-103a-1-3p	mir-92a-1-3p	-0.251767435	0.284255385	-0.822209596	8.67E-06
let-7a-3-5p	mir-125b-1-5p	-0.232827763	0.323222698	0.582590899	0.007027097

let-7a-3-5p	mir-125b-2-5p	-0.232827763	0.323222698	0.582590899	0.007027097
let-7a-2-5p	mir-125b-1-5p	-0.232827763	0.323222698	0.582590899	0.007027097
let-7a-2-5p	mir-125b-2-5p	-0.232827763	0.323222698	0.582590899	0.007027097
let-7a-1-5p	mir-125b-1-5p	-0.232827763	0.323222698	0.582590899	0.007027097
let-7a-1-5p	mir-125b-2-5p	-0.232827763	0.323222698	0.582590899	0.007027097
let-7a-3-5p	mir-99a-5p	-0.27242155	0.245234291	0.552479127	0.011533317
let-7a-2-5p	mir-99a-5p	-0.27242155	0.245234291	0.552479127	0.011533317
let-7a-1-5p	mir-99a-5p	-0.27242155	0.245234291	0.552479127	0.011533317
mir-146b-5p	mir-221-3p	0.776267351	5.73E-05	0.959286455	2.52E-11
mir-191-5p	mir-24-2-3p	0.711330124	0.000437083	0.945345711	3.39E-10
mir-191-5p	mir-24-1-3p	0.711330124	0.000437083	0.945345711	3.39E-10
mir-126-3p	mir-181a-2-5p	0.46395946	0.039338648	0.88270801	2.59E-07
mir-126-3p	mir-181a-1-5p	0.46395946	0.039338648	0.88270801	2.59E-07
mir-106b-3p	mir-20a-5p	0.930721691	2.71E-09	0.652316007	0.001826073
mir-27b-3p	mir-150-5p	0.579702582	0.007383949	-0.21782914	0.356224883
mir-146a-5p	mir-151a-3p	0.554243782	0.011217057	0.906510735	3.68E-08
mir-182-5p	mir-98-5p	-0.094147593	0.692983723	-0.74965327	0.000141649
mir-423-3p	mir-148b-3p	0.094612092	0.691540127	0.749268679	0.000143398
mir-21-5p	mir-148a-3p	-0.324465002	0.162798471	0.491942114	0.027580728
mir-30c-1-5p	mir-99a-5p	-0.34465048	0.136720725	0.47374804	0.034852089
mir-99a-5p	mir-30c-2-5p	-0.34465048	0.136720725	0.47374804	0.034852089
mir-28-3p	mir-20b-5p	-0.126264722	0.595803423	-0.761572722	9.58E-05
mir-146b-5p	mir-139-5p	0.341437548	0.140660435	0.841144778	3.39E-06
mir-199a-1-5p	mir-148a-3p	-0.405387504	0.076186385	0.407845204	0.07425366
mir-148a-3p	mir-199a-2-5p	-0.405387504	0.076186385	0.407845204	0.07425366
mir-146b-5p	mir-199a-2-3p	0.668241384	0.001279882	0.931186935	2.56E-09
mir-146b-5p	mir-199a-1-3p	0.668241384	0.001279882	0.931186935	2.56E-09
mir-146b-5p	mir-199b-3p	0.668241384	0.001279882	0.931186935	2.56E-09
mir-30d-5p	mir-139-5p	0.506479769	0.022677528	0.888755146	1.64E-07
mir-151a-5p	mir-148a-3p	-0.454499854	0.044087653	0.34904058	0.131464217
mir-27b-3p	mir-10b-5p	0.55453644	0.011165293	-0.224722857	0.340822921
mir-146a-5p	mir-629-5p	-0.401548962	0.079279613	-0.856190867	1.46E-06
let-7d-5p	mir-99a-5p	-0.418921441	0.065994508	0.384523171	0.094132517
mir-181a-2-5p	mir-151a-3p	0.490022853	0.028285954	0.882523277	2.62E-07
mir-151a-3p	mir-181a-1-5p	0.490022853	0.028285954	0.882523277	2.62E-07
mir-423-3p	mir-126-5p	0.093679928	0.694438188	0.737270483	0.000208099
mir-125b-1-5p	let-7d-3p	-0.207480756	0.380082545	0.564855755	0.009460073
let-7d-3p	mir-125b-2-5p	-0.207480756	0.380082545	0.564855755	0.009460073
mir-223-3p	mir-148a-3p	-0.356114851	0.123297556	0.443417759	0.050199931
mir-192-5p	mir-486-2-5p	0.921391886	8.16E-09	0.635524845	0.002601057
mir-125a-5p	mir-122-5p	-0.232317871	0.324313696	0.544385236	0.013077726
let-7f-2-5p	mir-98-5p	0.84058932	3.49E-06	0.968653777	2.48E-12
mir-98-5p	let-7f-1-5p	0.84058932	3.49E-06	0.968653777	2.48E-12
mir-103a-2-3p	mir-24-2-3p	0.487445571	0.029255189	0.880396321	3.06E-07
mir-103a-2-3p	mir-24-1-3p	0.487445571	0.029255189	0.880396321	3.06E-07
mir-103a-1-3p	mir-24-2-3p	0.487445571	0.029255189	0.880396321	3.06E-07
mir-103a-1-3p	mir-24-1-3p	0.487445571	0.029255189	0.880396321	3.06E-07
mir-182-5p	mir-28-3p	-0.193032478	0.414847014	-0.777517346	5.47E-05
mir-27b-3p	mir-103a-2-3p	0.305198164	0.190708617	0.819828216	9.68E-06
mir-27b-3p	mir-103a-1-3p	0.305198164	0.190708617	0.819828216	9.68E-06
mir-182-5p	mir-199a-2-3p	-0.313506152	0.178304695	-0.822672083	8.48E-06
mir-182-5p	mir-199a-1-3p	-0.313506152	0.178304695	-0.822672083	8.48E-06
mir-182-5p	mir-199b-3p	-0.313506152	0.178304695	-0.822672083	8.48E-06
mir-199a-2-3p	mir-23a-3p	0.607051083	0.004534446	0.912921178	1.99E-08
mir-199a-1-3p	mir-23a-3p	0.607051083	0.004534446	0.912921178	1.99E-08
mir-199b-3p	mir-23a-3p	0.607051083	0.004534446	0.912921178	1.99E-08
mir-27b-3p	mir-223-3p	0.469490575	0.036751672	0.873853454	4.82E-07
mir-146b-5p	mir-28-3p	0.468395428	0.037253078	0.87254141	5.26E-07
mir-629-5p	norepi	0.138742566	0.559653214	-0.60083287	0.005085149
mir-375	norepi	-0.242942178	0.302034706	0.525807635	0.017257679
mir-27b-3p	mir-146b-5p	0.583538116	0.006913193	0.905151116	4.16E-08
mir-25-3p	mir-148b-3p	0.515554197	0.019985998	-0.253952188	0.279956197
mir-21-5p	mir-28-3p	0.489589356	0.028447187	0.877354243	3.79E-07
mir-744-5p	mir-99a-5p	-0.381226122	0.097229421	0.40150305	0.079317166

mir-744-5p	mir-122-5p	-0.296930065	0.203616355	0.475595446	0.034052029
mir-25-3p	mir-186-5p	0.954145919	7.20E-11	0.783153911	4.44E-05
mir-27b-3p	mir-181a-2-5p	0.202907743	0.390904156	0.77277378	6.49E-05
mir-27b-3p	mir-181a-1-5p	0.202907743	0.390904156	0.77277378	6.49E-05
mir-92a-2-3p	mir-122-5p	0.121458583	0.609981682	-0.603517025	0.004841034
mir-122-5p	mir-92a-1-3p	0.121458583	0.609981682	-0.603517025	0.004841034
mir-148b-3p	mir-486-2-5p	0.422623303	0.063394542	-0.352688454	0.12720658
mir-26a-2-5p	mir-148a-3p	-0.322641055	0.165312448	0.449601866	0.046714075
mir-148a-3p	mir-26a-1-5p	-0.322641055	0.165312448	0.449601866	0.046714075
mir-106a-5p	mir-122-5p	0.281222673	0.229707067	-0.485048879	0.030179736
mir-92a-2-3p	mir-191-5p	-0.587632959	0.006438046	-0.903427179	4.87E-08
mir-191-5p	mir-92a-1-3p	-0.587632959	0.006438046	-0.903427179	4.87E-08
mir-199a-2-3p	mir-151a-3p	0.661037809	0.00150688	0.923211492	6.66E-09
mir-151a-3p	mir-199a-1-3p	0.661037809	0.00150688	0.923211492	6.66E-09
mir-151a-3p	mir-199b-3p	0.661037809	0.00150688	0.923211492	6.66E-09
let-7i-5p	mir-139-5p	0.176114167	0.457646252	0.758719023	0.000105393
mir-146b-5p	mir-1307-3p	0.46415171	0.039246426	0.866118131	7.99E-07
mir-126-3p	mir-182-5p	-0.211676001	0.370304645	-0.771062806	6.90E-05
mir-106b-3p	mir-186-5p	0.914826956	1.64E-08	0.635148565	0.002621151
mir-92a-2-3p	mir-99a-5p	0.076584929	0.748273583	-0.621771818	0.003424407
mir-99a-5p	mir-92a-1-3p	0.076584929	0.748273583	-0.621771818	0.003424407
mir-30d-5p	mir-126-5p	0.556259446	0.010864447	0.891822657	1.29E-07
mir-146a-5p	mir-15a-5p	-0.534304365	0.015230198	-0.884791037	2.22E-07
mir-194-2-5p	mir-15a-5p	0.844504127	2.83E-06	0.409837063	0.072714306
mir-194-1-5p	mir-15a-5p	0.844504127	2.83E-06	0.409837063	0.072714306
mir-148a-3p	mir-375	0.315310733	0.175684758	-0.440603481	0.051850663
mir-182-5p	mir-148b-3p	0.380020174	0.098380533	-0.378935131	0.099424705
mir-92a-2-3p	mir-151a-3p	-0.633783171	0.002695153	-0.913100422	1.95E-08
mir-151a-3p	mir-92a-1-3p	-0.633783171	0.002695153	-0.913100422	1.95E-08
mir-122-5p	mir-99b-5p	-0.03413339	0.886399864	0.642445819	0.002253747
mir-27b-3p	mir-98-5p	0.416134258	0.068004398	0.844936656	2.76E-06
mir-7-3-5p	mir-192-5p	0.92323064	6.64E-09	0.672779922	0.001152208
mir-192-5p	mir-7-1-5p	0.92323064	6.64E-09	0.672779922	0.001152208
mir-192-5p	mir-7-2-5p	0.92323064	6.64E-09	0.672779922	0.001152208
mir-584-5p	mir-99a-5p	-0.569891394	0.008708541	0.144363341	0.54369392
mir-27b-3p	mir-361-5p	0.571770371	0.008440907	0.894219614	1.07E-07
mir-151a-3p	mir-221-3p	0.722185527	0.000323557	0.935630515	1.42E-09
mir-182-5p	norepi	0.233128342	0.322580586	-0.500411344	0.024632379
mir-92a-2-3p	mir-194-2-5p	0.686205013	0.000835499	0.057516482	0.809665132
mir-92a-2-3p	mir-194-1-5p	0.686205013	0.000835499	0.057516482	0.809665132
mir-194-2-5p	mir-92a-1-3p	0.686205013	0.000835499	0.057516482	0.809665132
mir-194-1-5p	mir-92a-1-3p	0.686205013	0.000835499	0.057516482	0.809665132
mir-146a-5p	mir-23a-3p	0.670220969	0.001222804	0.920736397	8.77E-09
mir-629-5p	mir-148b-3p	0.41198621	0.071080281	-0.331309651	0.153599576
mir-486-2-5p	mir-28-3p	-0.207832919	0.37925623	-0.757624081	0.000109297
mir-26a-2-5p	mir-23a-3p	0.561834921	0.009935671	0.887938403	1.75E-07
mir-23a-3p	mir-26a-1-5p	0.561834921	0.009935671	0.887938403	1.75E-07
mir-146a-5p	mir-744-5p	0.80114606	2.19E-05	0.954166355	7.18E-11
mir-92a-2-3p	mir-125b-1-5p	0.297386747	0.202888633	-0.435026673	0.055243921
mir-92a-2-3p	mir-125b-2-5p	0.297386747	0.202888633	-0.435026673	0.055243921
mir-125b-1-5p	mir-92a-1-3p	0.297386747	0.202888633	-0.435026673	0.055243921
mir-125b-2-5p	mir-92a-1-3p	0.297386747	0.202888633	-0.435026673	0.055243921
mir-27b-3p	mir-146a-5p	0.50415333	0.023411854	0.868611035	6.81E-07
let-7f-2-5p	mir-125b-1-5p	-0.263980611	0.260743129	0.462249963	0.040166092
let-7f-2-5p	mir-125b-2-5p	-0.263980611	0.260743129	0.462249963	0.040166092
mir-125b-1-5p	let-7f-1-5p	-0.263980611	0.260743129	0.462249963	0.040166092
let-7f-1-5p	mir-125b-2-5p	-0.263980611	0.260743129	0.462249963	0.040166092
mir-182-5p	mir-146a-5p	-0.413338251	0.070066489	-0.836562119	4.30E-06
mir-106a-5p	mir-192-5p	0.896874039	8.57E-08	0.597144439	0.005437071
mir-423-3p	mir-151a-3p	0.321010453	0.167582417	0.800069072	2.29E-05
mir-26a-2-5p	mir-139-5p	0.518034112	0.019296608	0.871505873	5.64E-07
mir-139-5p	mir-26a-1-5p	0.518034112	0.019296608	0.871505873	5.64E-07
mir-125b-1-5p	let-7d-5p	-0.28490269	0.22340866	0.44024276	0.052065208
let-7d-5p	mir-125b-2-5p	-0.28490269	0.22340866	0.44024276	0.052065208

mir-30c-1-5p	mir-27a-3p	-0.141852086	0.550798593	0.549897454	0.012008972
mir-27a-3p	mir-30c-2-5p	-0.141852086	0.550798593	0.549897454	0.012008972
mir-148b-3p	mir-139-5p	-0.021317079	0.928919718	0.628434858	0.003002003
mir-182-5p	mir-191-5p	-0.186899719	0.430105111	-0.738590608	0.000199937
mir-148a-3p	mir-139-5p	-0.42811877	0.059678351	0.291694987	0.212082506
mir-148a-3p	mir-374b-5p	-0.155421475	0.512914983	0.537224594	0.014579343
mir-144-3p	norepi	0.156642353	0.509568518	-0.535268155	0.01501286
mir-98-5p	mir-629-5p	-0.184048216	0.437299534	-0.735461275	0.000219746
mir-148b-3p	mir-20b-5p	0.44836247	0.047397339	-0.265019478	0.258801709
mir-199a-1-5p	mir-125a-5p	0.819907743	9.65E-06	0.384016312	0.094603846
mir-125a-5p	mir-199a-2-5p	0.819907743	9.65E-06	0.384016312	0.094603846
mir-27b-3p	mir-139-5p	0.320779864	0.167905139	0.794741191	2.84E-05
mir-361-5p	mir-199a-2-3p	0.79641947	2.65E-05	0.95044122	1.43E-10
mir-361-5p	mir-199a-1-3p	0.79641947	2.65E-05	0.95044122	1.43E-10
mir-361-5p	mir-199b-3p	0.79641947	2.65E-05	0.95044122	1.43E-10
mir-340-5p	mir-126-5p	0.488448569	0.028874941	0.856833746	1.41E-06
mir-320a	mir-148b-3p	0.550082108	0.011974433	-0.127994702	0.590733787
mir-27b-3p	mir-199a-2-3p	0.519379651	0.018930579	0.867426906	7.35E-07
mir-27b-3p	mir-199a-1-3p	0.519379651	0.018930579	0.867426906	7.35E-07
mir-27b-3p	mir-199b-3p	0.519379651	0.018930579	0.867426906	7.35E-07
mir-99a-5p	let-7d-3p	-0.19300199	0.414922136	0.499295437	0.025005927
mir-22-3p	mir-93-5p	0.929874889	3.01E-09	0.723113911	0.000315141
mir-20a-5p	mir-486-2-5p	0.944994439	3.58E-10	0.778050204	5.37E-05
mir-146a-5p	mir-30c-1-5p	0.922786672	6.98E-09	0.69966086	0.000595259
mir-146a-5p	mir-30c-2-5p	0.922786672	6.98E-09	0.69966086	0.000595259
mir-423-3p	mir-99a-5p	-0.560158912	0.010207808	0.107096786	0.653137086
mir-103a-2-3p	mir-20b-5p	0.329369637	0.156169323	-0.378559597	0.099787967
mir-103a-1-3p	mir-20b-5p	0.329369637	0.156169323	-0.378559597	0.099787967
mir-744-5p	mir-151a-3p	0.541515434	0.013663781	0.873110675	5.06E-07
mir-125a-5p	mir-30c-1-5p	0.847398574	2.42E-06	0.469134578	0.036914084
mir-125a-5p	mir-30c-2-5p	0.847398574	2.42E-06	0.469134578	0.036914084
mir-103a-2-3p	mir-15a-5p	-0.02089722	0.930316416	-0.639411745	0.002400903
mir-103a-1-3p	mir-15a-5p	-0.02089722	0.930316416	-0.639411745	0.002400903
mir-103a-2-3p	mir-106b-3p	0.177648825	0.453673242	-0.505513078	0.022980406
mir-103a-1-3p	mir-106b-3p	0.177648825	0.453673242	-0.505513078	0.022980406
mir-27b-3p	mir-584-5p	-0.021412786	0.928601369	0.613438109	0.004021046
mir-98-5p	mir-15a-5p	-0.273543819	0.243217864	-0.767436927	7.84E-05
mir-125a-5p	norepi	0.154848423	0.514489335	0.710891524	0.000442303
mir-148a-3p	mir-486-2-5p	0.34052541	0.141793283	-0.360900195	0.117982624
mir-23a-3p	let-7d-3p	0.640519415	0.002346277	0.903515824	4.83E-08
mir-29a-3p	mir-30c-1-5p	0.033226293	0.889400804	0.642653827	0.002243941
mir-29a-3p	mir-30c-2-5p	0.033226293	0.889400804	0.642653827	0.002243941
mir-148b-3p	mir-30c-1-5p	0.153124624	0.519238942	0.708119453	0.000476539
mir-148b-3p	mir-30c-2-5p	0.153124624	0.519238942	0.708119453	0.000476539
mir-191-5p	mir-125b-1-5p	-0.292247757	0.211177771	0.403632871	0.077588971
mir-191-5p	mir-125b-2-5p	-0.292247757	0.211177771	0.403632871	0.077588971
let-7f-2-5p	mir-99a-5p	-0.236977316	0.314425518	0.452026683	0.045399212
let-7f-1-5p	mir-99a-5p	-0.236977316	0.314425518	0.452026683	0.045399212
mir-340-5p	mir-139-5p	0.543124208	0.013332696	0.870780383	5.91E-07
mir-101-1-3p	mir-486-2-5p	0.960241327	2.04E-11	0.840470251	3.51E-06
mir-486-2-5p	mir-101-2-3p	0.960241327	2.04E-11	0.840470251	3.51E-06
mir-199a-2-3p	mir-15a-5p	-0.507477957	0.022368112	-0.857789313	1.33E-06
mir-15a-5p	mir-199a-1-3p	-0.507477957	0.022368112	-0.857789313	1.33E-06
mir-15a-5p	mir-199b-3p	-0.507477957	0.022368112	-0.857789313	1.33E-06
mir-125b-1-5p	mir-30c-1-5p	-0.266668771	0.255738382	0.423678854	0.062667546
mir-125b-1-5p	mir-30c-2-5p	-0.266668771	0.255738382	0.423678854	0.062667546
mir-30c-1-5p	mir-125b-2-5p	-0.266668771	0.255738382	0.423678854	0.062667546
mir-30c-2-5p	mir-125b-2-5p	-0.266668771	0.255738382	0.423678854	0.062667546
mir-126-3p	mir-423-3p	0.304307854	0.192071405	0.777259935	5.53E-05
mir-3615	mir-28-3p	-0.239477877	0.309194451	-0.747307414	0.000152608
mir-27b-3p	mir-744-5p	0.449528461	0.04675433	0.835610649	4.51E-06
let-7f-2-5p	mir-139-5p	0.533325363	0.015453547	0.865969785	8.07E-07
mir-139-5p	let-7f-1-5p	0.533325363	0.015453547	0.865969785	8.07E-07
mir-199a-2-3p	mir-99a-5p	-0.245649291	0.296510657	0.438345926	0.053204555

mir-199a-1-3p	mir-99a-5p	-0.245649291	0.296510657	0.438345926	0.053204555
mir-199b-3p	mir-99a-5p	-0.245649291	0.296510657	0.438345926	0.053204555
mir-126-3p	mir-23a-3p	0.643880055	0.002186854	0.902485601	5.29E-08
mir-199a-1-5p	mir-28-3p	0.304509436	0.191762275	0.774039804	6.21E-05
mir-199a-2-5p	mir-28-3p	0.304509436	0.191762275	0.774039804	6.21E-05
mir-103a-2-3p	mir-486-2-5p	0.303210267	0.193760453	-0.381142953	0.097308491
mir-103a-1-3p	mir-486-2-5p	0.303210267	0.193760453	-0.381142953	0.097308491
mir-22-3p	mir-25-3p	0.868292535	6.95E-07	0.546082695	0.012740741
mir-139-5p	mir-99a-5p	-0.133900021	0.573566924	0.521247676	0.018431625
mir-27b-3p	let-7a-3-5p	0.433706948	0.056071088	0.826218492	7.17E-06
mir-27b-3p	let-7a-2-5p	0.433706948	0.056071088	0.826218492	7.17E-06
mir-27b-3p	let-7a-1-5p	0.433706948	0.056071088	0.826218492	7.17E-06
mir-221-3p	mir-99a-5p	-0.096857979	0.684574757	0.546769405	0.012606421
mir-532-5p	mir-122-5p	0.152405343	0.521226884	-0.505609293	0.022950118
mir-21-5p	mir-23a-3p	0.621160601	0.003465508	0.892838731	1.19E-07
mir-181a-2-5p	mir-28-5p	0.59705931	0.005445425	0.884702882	2.23E-07
mir-28-5p	mir-181a-1-5p	0.59705931	0.005445425	0.884702882	2.23E-07
mir-126-3p	mir-191-5p	0.729395837	0.000262949	0.926874661	4.35E-09
mir-122-5p	let-7d-5p	-0.374813524	0.103464703	0.304192911	0.192247823
mir-320a	mir-106b-3p	0.935309791	1.49E-09	0.757868469	0.000108415
mir-191-5p	mir-139-5p	0.665751005	0.001354857	0.906827365	3.57E-08
mir-126-3p	mir-361-5p	0.672999169	0.001146323	0.909030781	2.90E-08
mir-151a-3p	mir-15a-5p	-0.360619439	0.118289838	-0.793621301	2.97E-05
mir-92a-2-3p	mir-146a-5p	-0.561447179	0.009998099	-0.871297408	5.72E-07
mir-146a-5p	mir-92a-1-3p	-0.561447179	0.009998099	-0.871297408	5.72E-07
let-7i-5p	mir-126-5p	0.384577528	0.094082072	0.803583193	1.98E-05
mir-92a-2-3p	mir-199a-2-3p	-0.585990394	0.006625315	-0.879200474	3.33E-07
mir-92a-2-3p	mir-199a-1-3p	-0.585990394	0.006625315	-0.879200474	3.33E-07
mir-92a-2-3p	mir-199b-3p	-0.585990394	0.006625315	-0.879200474	3.33E-07
mir-199a-2-3p	mir-92a-1-3p	-0.585990394	0.006625315	-0.879200474	3.33E-07
mir-199a-1-3p	mir-92a-1-3p	-0.585990394	0.006625315	-0.879200474	3.33E-07
mir-199b-3p	mir-92a-1-3p	-0.585990394	0.006625315	-0.879200474	3.33E-07
mir-181a-2-5p	mir-24-2-3p	0.525029352	0.017453692	0.857377365	1.37E-06
mir-181a-2-5p	mir-24-1-3p	0.525029352	0.017453692	0.857377365	1.37E-06
mir-24-2-3p	mir-181a-1-5p	0.525029352	0.017453692	0.857377365	1.37E-06
mir-24-1-3p	mir-181a-1-5p	0.525029352	0.017453692	0.857377365	1.37E-06
mir-744-5p	mir-629-5p	-0.579625904	0.00739362	-0.876771165	3.95E-07
let-7a-3-5p	mir-122-5p	-0.282510537	0.227489886	0.386105946	0.092671757
let-7a-2-5p	mir-122-5p	-0.282510537	0.227489886	0.386105946	0.092671757
mir-122-5p	let-7a-1-5p	-0.282510537	0.227489886	0.386105946	0.092671757
let-7i-5p	mir-374b-5p	0.342177075	0.139746643	0.783408394	4.40E-05
mir-340-5p	mir-148b-3p	0.408431544	0.073798021	0.811194139	1.43E-05
mir-182-5p	mir-148a-3p	0.356547445	0.122810192	-0.312083837	0.180388271
mir-423-3p	mir-28-3p	0.409571764	0.072917946	0.811111106	1.43E-05
mir-122-5p	mir-15a-5p	0.091060911	0.702602372	-0.538710416	0.014256882
mir-182-5p	mir-221-3p	-0.449878132	0.04656281	-0.826565011	7.05E-06
mir-199a-2-3p	mir-125b-1-5p	-0.251972928	0.283849284	0.409542956	0.072940085
mir-199a-2-3p	mir-125b-2-5p	-0.251972928	0.283849284	0.409542956	0.072940085
mir-125b-1-5p	mir-199a-1-3p	-0.251972928	0.283849284	0.409542956	0.072940085
mir-125b-1-5p	mir-199b-3p	-0.251972928	0.283849284	0.409542956	0.072940085
mir-199a-1-3p	mir-125b-2-5p	-0.251972928	0.283849284	0.409542956	0.072940085
mir-199b-3p	mir-125b-2-5p	-0.251972928	0.283849284	0.409542956	0.072940085
mir-98-5p	let-7d-5p	0.858346888	1.29E-06	0.96244132	1.23E-11
mir-98-5p	mir-125b-1-5p	-0.129061801	0.587615773	0.508806651	0.021961453
mir-98-5p	mir-125b-2-5p	-0.129061801	0.587615773	0.508806651	0.021961453
mir-106a-5p	mir-148b-3p	0.449691507	0.046664953	-0.201759318	0.393648386
mir-486-2-5p	mir-186-5p	0.920408639	9.09E-09	0.717755943	0.000366399
mir-192-5p	mir-25-3p	0.899094441	7.11E-08	0.652199138	0.001830707
mir-182-5p	mir-181a-2-5p	0.119702283	0.615196736	-0.513663417	0.020524681
mir-182-5p	mir-181a-1-5p	0.119702283	0.615196736	-0.513663417	0.020524681
mir-182-5p	mir-146b-5p	-0.532533752	0.015636061	-0.856883836	1.41E-06
mir-629-5p	mir-151a-3p	-0.232982605	0.322891821	-0.728285512	0.000271596
mir-425-5p	mir-106b-3p	0.707217001	0.000488159	0.19189605	0.417652101
let-7a-3-5p	mir-139-5p	0.615387203	0.003874325	0.885998048	2.03E-07

let-7a-2-5p	mir-139-5p	0.615387203	0.003874325	0.885998048	2.03E-07
let-7a-1-5p	mir-139-5p	0.615387203	0.003874325	0.885998048	2.03E-07
mir-126-3p	mir-146a-5p	0.673436422	0.001134661	0.905533387	4.02E-08
mir-7-3-5p	mir-122-5p	0.182469083	0.441310784	-0.462266445	0.04015805
mir-122-5p	mir-7-1-5p	0.182469083	0.441310784	-0.462266445	0.04015805
mir-122-5p	mir-7-2-5p	0.182469083	0.441310784	-0.462266445	0.04015805
mir-146a-5p	mir-99a-5p	-0.23989882	0.308319056	0.412642369	0.070586915
mir-26a-2-5p	mir-99a-5p	-0.28422256	0.224564119	0.372273629	0.106012897
mir-99a-5p	mir-26a-1-5p	-0.28422256	0.224564119	0.372273629	0.106012897
mir-144-3p	mir-107	0.958878191	2.75E-11	0.847955066	2.34E-06
mir-103a-2-3p	mir-106a-5p	0.235757159	0.316997213	-0.414343799	0.069319561
mir-106a-5p	mir-103a-1-3p	0.235757159	0.316997213	-0.414343799	0.069319561
mir-125b-1-5p	mir-148a-3p	0.475654674	0.034026618	-0.161989091	0.49503709
mir-148a-3p	mir-125b-2-5p	0.475654674	0.034026618	-0.161989091	0.49503709
mir-125b-1-5p	mir-221-3p	-0.25105051	0.285675005	0.399953484	0.080592372
mir-221-3p	mir-125b-2-5p	-0.25105051	0.285675005	0.399953484	0.080592372
mir-122-5p	mir-199a-2-3p	-0.207843964	0.37923033	0.437380117	0.053791933
mir-122-5p	mir-199a-1-3p	-0.207843964	0.37923033	0.437380117	0.053791933
mir-122-5p	mir-199b-3p	-0.207843964	0.37923033	0.437380117	0.053791933
mir-103a-2-3p	mir-361-5p	0.419266162	0.065749064	0.809882616	1.51E-05
mir-361-5p	mir-103a-1-3p	0.419266162	0.065749064	0.809882616	1.51E-05
mir-223-3p	mir-148b-3p	0.362552556	0.116186123	0.785070432	4.13E-05
mir-126-3p	mir-26a-2-5p	0.762266579	9.36E-05	0.932850939	2.06E-09
mir-126-3p	mir-26a-1-5p	0.762266579	9.36E-05	0.932850939	2.06E-09
mir-340-5p	mir-99a-5p	-0.323451808	0.164191699	0.329249354	0.156329624
mir-26a-2-5p	mir-103a-2-3p	0.622919407	0.003348332	0.88672534	1.92E-07
mir-26a-2-5p	mir-103a-1-3p	0.622919407	0.003348332	0.88672534	1.92E-07
mir-103a-2-3p	mir-26a-1-5p	0.622919407	0.003348332	0.88672534	1.92E-07
mir-103a-1-3p	mir-26a-1-5p	0.622919407	0.003348332	0.88672534	1.92E-07
mir-126-3p	mir-151a-3p	0.77582402	5.82E-05	0.936736165	1.22E-09
mir-185-5p	mir-20b-5p	0.941669326	6.00E-10	0.792013655	3.16E-05
mir-629-5p	mir-107	0.916905595	1.32E-08	0.713249327	0.000414852
mir-103a-2-3p	mir-144-3p	0.33029373	0.154941579	-0.319969934	0.169042054
mir-144-3p	mir-103a-1-3p	0.33029373	0.154941579	-0.319969934	0.169042054
mir-98-5p	mir-99a-5p	-0.256904249	0.274211588	0.390021005	0.089129997
mir-181a-2-5p	mir-139-5p	0.58381914	0.006879692	0.872145723	5.40E-07
mir-139-5p	mir-181a-1-5p	0.58381914	0.006879692	0.872145723	5.40E-07
mir-27b-3p	mir-28-3p	0.501646933	0.02422393	0.841182106	3.38E-06
let-7b-5p	mir-148b-3p	0.381236506	0.097219552	-0.265452325	0.257995519
mir-629-5p	mir-15b-5p	0.540017354	0.013978014	-0.068977835	0.772612304
mir-27b-3p	mir-148b-3p	0.173364423	0.46480933	0.689322059	0.000773595
mir-101-1-3p	mir-106b-3p	0.941477591	6.18E-10	0.793111272	3.03E-05
mir-106b-3p	mir-101-2-3p	0.941477591	6.18E-10	0.793111272	3.03E-05
mir-182-5p	mir-128-1-3p	0.05608341	0.814327299	-0.546596957	0.012640044
mir-182-5p	mir-128-2-3p	0.05608341	0.814327299	-0.546596957	0.012640044
mir-148a-3p	mir-106b-3p	0.306560733	0.188635565	-0.338772488	0.1439883
mir-103a-2-3p	mir-139-5p	0.588140831	0.006381031	0.872691564	5.21E-07
mir-103a-1-3p	mir-139-5p	0.588140831	0.006381031	0.872691564	5.21E-07
mir-27b-3p	mir-199a-1-5p	0.524434102	0.017604806	0.848723937	2.24E-06
mir-27b-3p	mir-199a-2-5p	0.524434102	0.017604806	0.848723937	2.24E-06
mir-486-2-5p	mir-30e-5p	0.846539264	2.53E-06	0.51916408	0.018988845
mir-98-5p	mir-146b-5p	0.663339167	0.001430953	0.899059921	7.13E-08
mir-182-5p	mir-140-5p	0.556708198	0.010787181	-0.03973594	0.867899445
mir-27b-3p	let-7i-5p	-0.072447039	0.761485818	0.533443789	0.01542639
mir-30d-5p	mir-374b-5p	0.617045368	0.003753022	0.882670854	2.59E-07
mir-1307-3p	cpeptide	0.203214173	0.390173723	-0.430623389	0.058040463
mir-92a-2-3p	mir-139-5p	-0.496838053	0.025844317	-0.836820475	4.24E-06
mir-139-5p	mir-92a-1-3p	-0.496838053	0.025844317	-0.836820475	4.24E-06
mir-194-2-5p	mir-99b-5p	-0.601275368	0.005044221	-0.031830269	0.894022078
mir-194-1-5p	mir-99b-5p	-0.601275368	0.005044221	-0.031830269	0.894022078
mir-182-5p	mir-374b-5p	0.055777142	0.815324456	-0.542150422	0.013532319
mir-93-5p	mir-28-3p	-0.076969036	0.747050494	-0.629174031	0.00295794
mir-191-5p	mir-99a-5p	-0.166183894	0.483779934	0.458214477	0.042172832
mir-182-5p	let-7i-5p	0.378671112	0.099679996	-0.257871431	0.272345612

mir-1307-3p	mir-151a-3p	0.233673517	0.321417895	0.716150035	0.00038308
mir-22-3p	mir-106b-3p	0.751954536	0.000131561	0.306047395	0.189414782
let-7i-5p	mir-629-5p	0.398910446	0.081459254	-0.233812043	0.321122861
mir-425-5p	mir-629-5p	0.732604159	0.000239273	0.267291851	0.254587116
mir-629-5p	mir-28-3p	-0.29877493	0.200687201	-0.747995439	0.000149321
mir-92a-2-3p	mir-98-5p	-0.429294382	0.058905259	-0.807235603	1.69E-05
mir-98-5p	mir-92a-1-3p	-0.429294382	0.058905259	-0.807235603	1.69E-05
mir-423-3p	mir-122-5p	-0.413838527	0.06969413	0.216300744	0.359693043
mir-423-5p	mir-484	0.922205238	7.46E-09	0.736988988	0.000209876
mir-10b-5p	let-7b-5p	-0.217488193	0.356996868	0.412317855	0.070830596
mir-92a-2-3p	mir-181a-2-5p	-0.219874476	0.351613973	-0.707741432	0.000481378
mir-92a-2-3p	mir-181a-1-5p	-0.219874476	0.351613973	-0.707741432	0.000481378
mir-181a-2-5p	mir-92a-1-3p	-0.219874476	0.351613973	-0.707741432	0.000481378
mir-92a-1-3p	mir-181a-1-5p	-0.219874476	0.351613973	-0.707741432	0.000481378
mir-182-5p	mir-122-5p	0.048870184	0.837882217	-0.543770971	0.01320143
mir-181a-2-5p	mir-146b-5p	0.3362749	0.147156733	0.764646635	8.63E-05
mir-146b-5p	mir-181a-1-5p	0.3362749	0.147156733	0.764646635	8.63E-05
mir-106a-5p	mir-28-3p	-0.154182822	0.516320824	-0.670914484	0.001203321
mir-103a-2-3p	mir-146b-5p	0.389652697	0.089458877	0.788696564	3.60E-05
mir-146b-5p	mir-103a-1-3p	0.389652697	0.089458877	0.788696564	3.60E-05
mir-106a-5p	mir-148a-3p	0.428664485	0.059318534	-0.195513042	0.408759644
mir-744-5p	mir-199a-2-3p	0.848407294	2.28E-06	0.956800757	4.25E-11
mir-744-5p	mir-199a-1-3p	0.848407294	2.28E-06	0.956800757	4.25E-11
mir-744-5p	mir-199b-3p	0.848407294	2.28E-06	0.956800757	4.25E-11
mir-21-5p	mir-99a-5p	-0.169682686	0.47448871	0.449931537	0.046533613
mir-146b-5p	mir-122-5p	-0.087349286	0.714226224	0.513877543	0.020463103
mir-584-5p	mir-20b-5p	-0.214852053	0.362998157	-0.703115736	0.00054405
mir-182-5p	mir-126-5p	0.167811897	0.479445509	-0.450313612	0.046325134
mir-629-5p	mir-186-5p	0.895602843	9.53E-08	0.66171179	0.001484302
mir-1307-3p	mir-221-3p	0.551145594	0.011777068	0.854793836	1.59E-06
mir-10b-5p	mir-106b-3p	-0.185071386	0.434710797	0.435077733	0.055212105
mir-361-5p	mir-99a-5p	-0.129635367	0.585942722	0.479382686	0.032456721
mir-122-5p	mir-221-3p	-0.069862756	0.769770018	0.524317128	0.017634624
mir-361-5p	mir-122-5p	-0.054022842	0.821041389	0.535653512	0.014926662
mir-199a-1-5p	mir-151a-5p	0.434767833	0.055405418	0.806756264	1.73E-05
mir-151a-5p	mir-199a-2-5p	0.434767833	0.055405418	0.806756264	1.73E-05
mir-106a-5p	norepi	0.049254831	0.836622608	-0.537961805	0.014418624
mir-148b-3p	mir-15a-5p	0.308968956	0.185008866	-0.319491579	0.169716004
mir-486-2-5p	mir-185-5p	0.950748446	1.35E-10	0.830330999	5.87E-06
mir-146b-5p	mir-99a-5p	-0.089538438	0.707362847	0.508087537	0.022180809
mir-423-3p	mir-27a-3p	-0.574940728	0.008004567	-0.005138846	0.982845324
mir-182-5p	mir-744-5p	-0.582520671	0.007035603	-0.865590817	8.26E-07
mir-22-3p	mir-629-5p	0.789326163	3.51E-05	0.397989369	0.082230503
mir-148a-3p	let-7d-5p	-0.452148553	0.045333888	0.159117475	0.502816346
mir-629-5p	mir-126-5p	0.172403961	0.467324687	-0.441159917	0.051521039
mir-361-5p	mir-151a-3p	0.590987309	0.006069095	0.868485084	6.87E-07
mir-199a-2-3p	mir-24-2-3p	0.772038099	6.67E-05	0.931878741	2.34E-09
mir-199a-2-3p	mir-24-1-3p	0.772038099	6.67E-05	0.931878741	2.34E-09
mir-24-2-3p	mir-199a-1-3p	0.772038099	6.67E-05	0.931878741	2.34E-09
mir-24-2-3p	mir-199b-3p	0.772038099	6.67E-05	0.931878741	2.34E-09
mir-199a-1-3p	mir-24-1-3p	0.772038099	6.67E-05	0.931878741	2.34E-09
mir-199b-3p	mir-24-1-3p	0.772038099	6.67E-05	0.931878741	2.34E-09
mir-103a-2-3p	mir-151a-3p	0.723012899	0.000316048	0.915500017	1.53E-08
mir-103a-1-3p	mir-151a-3p	0.723012899	0.000316048	0.915500017	1.53E-08
mir-340-5p	mir-629-5p	-0.267310921	0.254551933	-0.725545466	0.000293979
mir-182-5p	mir-99a-5p	0.258843607	0.27047802	-0.3628956	0.11581563
mir-106b-3p	mir-30e-5p	0.783410374	4.40E-05	0.388822115	0.090203817
mir-363-3p	norepi	0.025194278	0.916031829	-0.550012082	0.011987521
mir-181a-2-5p	mir-125b-1-5p	-0.150320063	0.52701031	0.454590334	0.04404023
mir-181a-2-5p	mir-125b-2-5p	-0.150320063	0.52701031	0.454590334	0.04404023
mir-125b-1-5p	mir-181a-1-5p	-0.150320063	0.52701031	0.454590334	0.04404023
mir-125b-2-5p	mir-181a-1-5p	-0.150320063	0.52701031	0.454590334	0.04404023
mir-425-5p	mir-20b-5p	0.783930719	4.31E-05	0.392226938	0.087178804
mir-30d-5p	mir-125b-1-5p	-0.305768299	0.189839342	0.31397656	0.177619201

mir-30d-5p	mir-125b-2-5p	-0.305768299	0.189839342	0.31397656	0.177619201
mir-191-5p	mir-629-5p	-0.246943139	0.293892434	-0.712853556	0.000419356
mir-7-3-5p	mir-148b-3p	0.419024866	0.065920796	-0.191868864	0.41771933
mir-7-1-5p	mir-148b-3p	0.419024866	0.065920796	-0.191868864	0.41771933
mir-148b-3p	mir-7-2-5p	0.419024866	0.065920796	-0.191868864	0.41771933
mir-21-5p	mir-139-5p	0.543484192	0.013259505	0.848242199	2.31E-06
mir-629-5p	mir-30e-5p	0.765016914	8.52E-05	0.352424826	0.127510946
mir-182-5p	mir-24-2-3p	-0.252299813	0.283204024	-0.715209131	0.000393151
mir-182-5p	mir-24-1-3p	-0.252299813	0.283204024	-0.715209131	0.000393151
mir-98-5p	mir-744-5p	0.632799697	0.002749527	0.88223344	2.68E-07
mir-584-5p	mir-1307-3p	0.219440484	0.352589445	0.697603502	0.000627653
mir-24-2-3p	mir-125b-1-5p	-0.128742201	0.588548899	0.468863793	0.037037994
mir-24-2-3p	mir-125b-2-5p	-0.128742201	0.588548899	0.468863793	0.037037994
mir-125b-1-5p	mir-24-1-3p	-0.128742201	0.588548899	0.468863793	0.037037994
mir-24-1-3p	mir-125b-2-5p	-0.128742201	0.588548899	0.468863793	0.037037994
let-7i-5p	mir-106b-3p	0.483350695	0.030848584	-0.110105789	0.644001277
mir-7-3-5p	mir-194-2-5p	0.887485984	1.81E-07	0.648542882	0.00198073
mir-7-3-5p	mir-194-1-5p	0.887485984	1.81E-07	0.648542882	0.00198073
mir-194-2-5p	mir-7-1-5p	0.887485984	1.81E-07	0.648542882	0.00198073
mir-194-2-5p	mir-7-2-5p	0.887485984	1.81E-07	0.648542882	0.00198073
mir-7-1-5p	mir-194-1-5p	0.887485984	1.81E-07	0.648542882	0.00198073
mir-194-1-5p	mir-7-2-5p	0.887485984	1.81E-07	0.648542882	0.00198073
mir-15a-5p	norepi	-0.035482875	0.881938134	-0.586586496	0.006556844
mir-320a	mir-186-5p	0.928487639	3.58E-09	0.766054754	8.22E-05
mir-181a-2-5p	mir-99a-5p	-0.213691708	0.365657903	0.396426269	0.08355169
mir-99a-5p	mir-181a-1-5p	-0.213691708	0.365657903	0.396426269	0.08355169
mir-21-5p	mir-122-5p	-0.184831587	0.435316791	0.421173485	0.064403433
mir-629-5p	let-7d-5p	-0.362751476	0.115971183	-0.768185867	7.63E-05
mir-629-5p	mir-20a-5p	0.872349758	5.33E-07	0.609581313	0.004324946
mir-106a-5p	mir-99a-5p	0.414710007	0.069049028	-0.188853813	0.425211392
mir-1307-3p	mir-199a-2-3p	0.419078598	0.065882525	0.792758512	3.07E-05
mir-1307-3p	mir-199a-1-3p	0.419078598	0.065882525	0.792758512	3.07E-05
mir-1307-3p	mir-199b-3p	0.419078598	0.065882525	0.792758512	3.07E-05
mir-15a-5p	mir-28-3p	-0.39289154	0.08659717	-0.780758305	4.86E-05
mir-629-5p	mir-221-3p	-0.551223482	0.011762718	-0.848903744	2.22E-06
mir-106a-5p	mir-320a	0.932295817	2.22E-09	0.77950182	5.09E-05
mir-26b-5p	mir-25-3p	0.968947861	2.28E-12	0.894379781	1.05E-07
mir-25-3p	mir-148a-3p	0.330703418	0.154399417	-0.279605087	0.232511729
mir-106a-5p	mir-151a-3p	0.047735669	0.841599639	-0.52487796	0.017492026
mir-144-3p	mir-486-2-5p	0.942198604	5.54E-10	0.810172054	1.49E-05
mir-139-5p	let-7d-3p	0.642470171	0.002252597	0.883480914	2.45E-07
mir-30c-1-5p	mir-378a-3p	-0.529120368	0.016442974	0.040051569	0.866859071
mir-378a-3p	mir-30c-2-5p	-0.529120368	0.016442974	0.040051569	0.866859071
mir-144-3p	mir-199a-2-3p	-0.183257344	0.439306074	-0.671948259	0.001174765
mir-144-3p	mir-199a-1-3p	-0.183257344	0.439306074	-0.671948259	0.001174765
mir-144-3p	mir-199b-3p	-0.183257344	0.439306074	-0.671948259	0.001174765
mir-26a-2-5p	mir-28-3p	0.497829727	0.025503357	0.825970131	7.26E-06
mir-28-3p	mir-26a-1-5p	0.497829727	0.025503357	0.825970131	7.26E-06
mir-182-5p	mir-15a-5p	0.754571367	0.000120844	0.923490416	6.45E-09
mir-10b-5p	mir-199a-1-5p	0.125211608	0.598898374	-0.463385029	0.039615201
mir-10b-5p	mir-199a-2-5p	0.125211608	0.598898374	-0.463385029	0.039615201
mir-192-5p	mir-451a	0.897427079	8.18E-08	0.681490933	0.000937047
mir-25-3p	mir-425-5p	0.817182406	1.09E-05	0.479236494	0.032517197
mir-423-5p	mir-15b-5p	0.841309119	3.36E-06	0.536744471	0.014684786
mir-181a-2-5p	mir-629-5p	0.088781123	0.709734736	-0.49009997	0.028257347
mir-629-5p	mir-181a-1-5p	0.088781123	0.709734736	-0.49009997	0.028257347
mir-199a-1-5p	mir-148b-3p	0.207553523	0.379911722	0.683225511	0.000898534
mir-148b-3p	mir-199a-2-5p	0.207553523	0.379911722	0.683225511	0.000898534
mir-25-3p	mir-185-5p	0.964048984	8.36E-12	0.880023585	3.14E-07
mir-103a-2-3p	mir-340-5p	0.646907623	0.002051082	0.884091051	2.34E-07
mir-340-5p	mir-103a-1-3p	0.646907623	0.002051082	0.884091051	2.34E-07
mir-143-3p	mir-28-3p	-0.054745193	0.818686312	0.514708596	0.0202255
mir-182-5p	mir-21-5p	-0.439419989	0.0525571	-0.798772675	2.41E-05
mir-744-5p	mir-23a-3p	0.547356451	0.012492504	0.844447126	2.84E-06

mir-148a-3p	let-7b-5p	0.318378095	0.171291902	-0.283674595	0.225497879
mir-629-5p	mir-484	0.728065377	0.000273339	0.295052074	0.206627161
mir-451a	mir-148b-3p	0.38268182	0.095853051	-0.213878259	0.365229542
let-7i-5p	cpeptide	-0.639201118	0.002411409	-0.135779456	0.568149127
mir-106b-3p	mir-28-3p	-0.278947214	0.233658707	-0.719061562	0.000353297
mir-22-3p	mir-20b-5p	0.822568563	8.52E-06	0.497607775	0.025579358
mir-181a-2-5p	mir-148a-3p	-0.325951341	0.160769399	0.273084973	0.244041001
mir-148a-3p	mir-181a-1-5p	-0.325951341	0.160769399	0.273084973	0.244041001
mir-26b-5p	mir-148b-3p	0.567736604	0.009023934	0.026561367	0.911492241
mir-92a-2-3p	mir-148a-3p	0.320927266	0.167698793	-0.277210773	0.236703703
mir-148a-3p	mir-92a-1-3p	0.320927266	0.167698793	-0.277210773	0.236703703
mir-182-5p	mir-26a-2-5p	-0.377515087	0.100803427	-0.767459649	7.83E-05
mir-182-5p	mir-26a-1-5p	-0.377515087	0.100803427	-0.767459649	7.83E-05
mir-25-3p	mir-28-3p	-0.173797947	0.463676234	-0.659757555	0.001550564
mir-139-5p	mir-10a-5p	0.160933198	0.497890671	0.651338723	0.001865125
mir-98-5p	mir-20b-5p	-0.017339204	0.942159958	-0.559742346	0.010276377
mir-98-5p	mir-122-5p	-0.288174226	0.217905061	0.308074332	0.186350593
mir-125b-1-5p	mir-139-5p	-0.142898115	0.547834171	0.43859619	0.053053153
mir-139-5p	mir-125b-2-5p	-0.142898115	0.547834171	0.43859619	0.053053153
mir-106b-3p	mir-484	0.72381594	0.000308902	0.292809002	0.210261776
mir-340-5p	mir-122-5p	-0.333066755	0.151297588	0.261070367	0.266230627
mir-139-5p	mir-126-5p	0.475084869	0.034271698	0.811009788	1.44E-05
mir-26a-2-5p	mir-148b-3p	0.402895896	0.078183773	0.777877581	5.40E-05
mir-148b-3p	mir-26a-1-5p	0.402895896	0.078183773	0.777877581	5.40E-05
mir-126-3p	mir-199a-2-3p	0.749528517	0.000142214	0.919297241	1.03E-08
mir-126-3p	mir-199a-1-3p	0.749528517	0.000142214	0.919297241	1.03E-08
mir-126-3p	mir-199b-3p	0.749528517	0.000142214	0.919297241	1.03E-08
mir-30c-1-5p	mir-126-5p	0.480789718	0.031879156	0.813225624	1.30E-05
mir-126-5p	mir-30c-2-5p	0.480789718	0.031879156	0.813225624	1.30E-05
mir-182-5p	let-7d-5p	-0.361255182	0.117595003	-0.757655247	0.000109185
mir-146a-5p	mir-125b-1-5p	-0.170482811	0.472376602	0.413682302	0.06981025
mir-146a-5p	mir-125b-2-5p	-0.170482811	0.472376602	0.413682302	0.06981025
mir-103a-2-3p	let-7b-5p	0.159587869	0.501538011	-0.422685655	0.063351422
mir-103a-1-3p	let-7b-5p	0.159587869	0.501538011	-0.422685655	0.063351422
mir-629-5p	mir-139-5p	-0.171314121	0.470187198	-0.655385819	0.001707828
mir-126-5p	mir-20b-5p	0.347705218	0.133047726	-0.243786244	0.300305663
mir-103a-2-3p	mir-93-5p	0.420727953	0.064715885	-0.161458907	0.49646893
mir-103a-1-3p	mir-93-5p	0.420727953	0.064715885	-0.161458907	0.49646893
mir-151a-5p	mir-99a-5p	-0.56449662	0.009515625	-0.02801955	0.906653078
mir-192-5p	let-7b-5p	0.87183511	5.51E-07	0.622796975	0.003356381
mir-22-3p	mir-15a-5p	0.741874437	0.000180814	0.330642505	0.154479943
let-7f-2-5p	mir-148a-3p	-0.325384526	0.161541116	0.266702708	0.255675592
mir-148a-3p	let-7f-1-5p	-0.325384526	0.161541116	0.266702708	0.255675592
mir-223-3p	mir-99a-5p	-0.19546121	0.408886342	0.390825136	0.088415042
mir-148b-3p	mir-374b-5p	0.476171387	0.033805558	0.810593496	1.46E-05
mir-199a-1-5p	mir-99b-5p	0.70697391	0.00049133	0.264574396	0.259632349
mir-99b-5p	mir-199a-2-5p	0.70697391	0.00049133	0.264574396	0.259632349
mir-106b-3p	mir-15b-5p	0.531347903	0.015912705	-0.017570614	0.941389286
mir-182-5p	let-7a-3-5p	-0.307150448	0.187743082	-0.728983854	0.00026613
mir-182-5p	let-7a-2-5p	-0.307150448	0.187743082	-0.728983854	0.00026613
mir-182-5p	let-7a-1-5p	-0.307150448	0.187743082	-0.728983854	0.00026613
mir-98-5p	let-7d-3p	0.830757087	5.75E-06	0.946763035	2.69E-10
mir-106b-3p	norepi	0.107488237	0.65194585	-0.46282115	0.039888136
mir-26a-2-5p	mir-151a-3p	0.670922729	0.001203091	0.889749215	1.52E-07
mir-151a-3p	mir-26a-1-5p	0.670922729	0.001203091	0.889749215	1.52E-07
mir-320a	mir-20a-5p	0.964862133	6.83E-12	0.886160476	2.00E-07
mir-98-5p	mir-146a-5p	0.809535142	1.53E-05	0.939451984	8.33E-10
let-7a-3-5p	mir-10a-5p	0.316988344	0.173272796	0.733335711	0.000234137
let-7a-2-5p	mir-10a-5p	0.316988344	0.173272796	0.733335711	0.000234137
let-7a-1-5p	mir-10a-5p	0.316988344	0.173272796	0.733335711	0.000234137
mir-191-5p	mir-15a-5p	-0.447538861	0.047855611	-0.796553796	2.64E-05
mir-182-5p	let-7g-5p	0.578756978	0.007503949	0.053230729	0.823625602
mir-26a-2-5p	let-7d-3p	0.957308527	3.83E-11	0.863214531	9.59E-07
let-7d-3p	mir-26a-1-5p	0.957308527	3.83E-11	0.863214531	9.59E-07

mir-30c-1-5p	mir-10a-5p	0.107935795	0.65058487	0.613916252	0.003984635
mir-10a-5p	mir-30c-2-5p	0.107935795	0.65058487	0.613916252	0.003984635
mir-584-5p	mir-122-5p	-0.495139863	0.02643654	0.063180096	0.791301613
mir-223-3p	mir-28-3p	0.277069252	0.236952996	0.711335042	0.000437025
mir-125b-1-5p	norepi	-0.252254733	0.283292956	0.3340961	0.149960248
mir-125b-2-5p	norepi	-0.252254733	0.283292956	0.3340961	0.149960248
mir-98-5p	mir-139-5p	0.49548561	0.026315106	0.817195007	1.09E-05
mir-103a-2-3p	mir-25-3p	0.306070107	0.189380261	-0.28101548	0.230065077
mir-25-3p	mir-103a-1-3p	0.306070107	0.189380261	-0.28101548	0.230065077
mir-182-5p	mir-28-5p	-0.194808702	0.410483152	-0.665316903	0.001368296
mir-151a-5p	mir-27a-3p	-0.381163465	0.097288985	0.200502817	0.396663041
mir-26a-2-5p	mir-122-5p	-0.245369528	0.297078651	0.339293818	0.143333017
mir-122-5p	mir-26a-1-5p	-0.245369528	0.297078651	0.339293818	0.143333017
mir-30d-5p	mir-99a-5p	-0.262551886	0.263428102	0.321317157	0.16715383
mir-182-5p	mir-425-5p	0.668135247	0.001283005	0.2030664	0.390525874
mir-26a-2-5p	mir-126-5p	0.515109158	0.020111765	0.824573633	7.76E-06
mir-126-5p	mir-26a-1-5p	0.515109158	0.020111765	0.824573633	7.76E-06
mir-27b-3p	mir-486-2-5p	-0.315287668	0.175718078	-0.729507812	0.000262091
mir-181a-2-5p	mir-23a-3p	0.489249305	0.028574169	0.813094831	1.31E-05
mir-23a-3p	mir-181a-1-5p	0.489249305	0.028574169	0.813094831	1.31E-05
mir-27a-3p	let-7g-5p	0.255913722	0.276130854	0.697223677	0.000633794
mir-361-5p	mir-146b-5p	0.761320038	9.66E-05	0.921518963	8.05E-09
let-7f-2-5p	mir-122-5p	-0.229430363	0.330533242	0.350111016	0.130204538
mir-122-5p	let-7f-1-5p	-0.229430363	0.330533242	0.350111016	0.130204538
mir-423-5p	mir-375	-0.138265286	0.56101787	0.430075695	0.058395678
mir-192-5p	mir-107	0.871293816	5.72E-07	0.628982399	0.002969311
mir-199a-2-3p	mir-20b-5p	-0.137681416	0.562689292	-0.626456271	0.003122642
mir-199a-1-3p	mir-20b-5p	-0.137681416	0.562689292	-0.626456271	0.003122642
mir-199b-3p	mir-20b-5p	-0.137681416	0.562689292	-0.626456271	0.003122642
mir-21-5p	let-7d-3p	0.949893404	1.58E-10	0.843722885	2.95E-06
mir-125b-1-5p	mir-23a-3p	-0.045428329	0.849169894	0.501419867	0.024298586
mir-23a-3p	mir-125b-2-5p	-0.045428329	0.849169894	0.501419867	0.024298586
mir-146b-5p	mir-24-2-3p	0.635318062	0.002612083	0.873329024	4.99E-07
mir-146b-5p	mir-24-1-3p	0.635318062	0.002612083	0.873329024	4.99E-07
mir-122-5p	mir-24-2-3p	0.028134555	0.90627155	0.554169076	0.011230301
mir-122-5p	mir-24-1-3p	0.028134555	0.90627155	0.554169076	0.011230301
mir-126-3p	mir-10b-5p	0.470654834	0.036224394	-0.085056337	0.721437738
mir-126-3p	let-7f-2-5p	0.739479455	0.000194598	0.912970317	1.98E-08
mir-126-3p	let-7f-1-5p	0.739479455	0.000194598	0.912970317	1.98E-08
mir-151a-5p	mir-128-1-3p	0.087653627	0.713270783	0.593802349	0.005773035
mir-151a-5p	mir-128-2-3p	0.087653627	0.713270783	0.593802349	0.005773035
mir-146a-5p	mir-122-5p	-0.139530662	0.557403088	0.426125697	0.061006536
mir-99a-5p	norepi	-0.400659567	0.080009433	0.168789625	0.476851729
mir-151a-3p	mir-106b-3p	-0.039047772	0.87016851	-0.560444131	0.010161074
mir-122-5p	norepi	-0.468595955	0.037160873	0.084949322	0.721774868
mir-27b-3p	mir-30d-5p	0.238567297	0.311093225	0.683899266	0.000883939
mir-143-3p	mir-30c-1-5p	-0.222331424	0.346121073	0.351386113	0.128715207
mir-143-3p	mir-30c-2-5p	-0.222331424	0.346121073	0.351386113	0.128715207
let-7d-5p	mir-15a-5p	-0.494901985	0.026520345	-0.812782033	1.33E-05
mir-7-3-5p	norepi	0.083256715	0.727113628	-0.469071505	0.036942917
mir-7-1-5p	norepi	0.083256715	0.727113628	-0.469071505	0.036942917
mir-7-2-5p	norepi	0.083256715	0.727113628	-0.469071505	0.036942917
mir-17-5p	mir-629-5p	0.753034498	0.000127043	0.369451306	0.108897417
mir-148a-3p	mir-126-5p	-0.181077486	0.444861562	0.386443664	0.092362238
mir-125a-5p	mir-194-2-5p	-0.619027681	0.003612153	-0.13224649	0.578351931
mir-125a-5p	mir-194-1-5p	-0.619027681	0.003612153	-0.13224649	0.578351931
let-7a-3-5p	mir-629-5p	-0.369502191	0.108844914	-0.752001444	0.000131362
let-7a-2-5p	mir-629-5p	-0.369502191	0.108844914	-0.752001444	0.000131362
let-7a-1-5p	mir-629-5p	-0.369502191	0.108844914	-0.752001444	0.000131362
mir-106b-3p	mir-126-5p	0.118312531	0.619336027	-0.438736888	0.05296818
mir-25-3p	mir-30e-5p	0.864604812	8.79E-07	0.618429067	0.003654222
mir-98-5p	mir-144-3p	0.006641401	0.977830485	-0.523802717	0.017766235
mir-182-5p	mir-361-5p	-0.555885483	0.010929178	-0.838096591	3.97E-06
mir-22-3p	mir-182-5p	0.745205103	0.000163038	0.35828088	0.120871073

mir-340-5p	mir-125b-1-5p	-0.368186938	0.110207868	0.198044271	0.402598368
mir-340-5p	mir-125b-2-5p	-0.368186938	0.110207868	0.198044271	0.402598368
mir-24-2-3p	mir-99a-5p	0.106172405	0.655953296	0.600181382	0.005145904
mir-99a-5p	mir-24-1-3p	0.106172405	0.655953296	0.600181382	0.005145904
mir-584-5p	mir-15a-5p	-0.325136707	0.161879321	-0.727882943	0.00027479
mir-27b-3p	mir-106b-3p	-0.374564339	0.103712716	-0.753262039	0.000126108
mir-101-1-3p	mir-122-5p	0.161069802	0.497521045	-0.4004405	0.080189958
mir-122-5p	mir-101-2-3p	0.161069802	0.497521045	-0.4004405	0.080189958
let-7b-5p	norepi	0.102866894	0.666060232	-0.448914584	0.047092025
mir-10b-5p	mir-486-2-5p	-0.116599295	0.624454008	0.437364065	0.053801736
mir-148a-3p	mir-99a-5p	0.745589584	0.000161086	0.360127288	0.118829751
let-7i-5p	mir-486-2-5p	0.510022439	0.021594515	-0.023061522	0.923118857
let-7g-5p	cpeptide	-0.47263676	0.035340364	0.071770314	0.76365276
mir-22-3p	mir-484	0.807603778	1.67E-05	0.936018723	1.35E-09
mir-181a-2-5p	mir-15a-5p	-0.090587575	0.704081267	-0.588625335	0.006327026
mir-15a-5p	mir-181a-1-5p	-0.090587575	0.704081267	-0.588625335	0.006327026
mir-222-3p	mir-107	0.785365753	4.09E-05	0.927988098	3.80E-09
mir-7-3-5p	mir-425-5p	0.766757586	8.02E-05	0.403903707	0.077371231
mir-425-5p	mir-7-1-5p	0.766757586	8.02E-05	0.403903707	0.077371231
mir-425-5p	mir-7-2-5p	0.766757586	8.02E-05	0.403903707	0.077371231
let-7i-5p	mir-375	-0.375890316	0.102397926	0.186609023	0.430835656
mir-7-3-5p	mir-148a-3p	0.333658974	0.150527159	-0.232671869	0.323556027
mir-7-1-5p	mir-148a-3p	0.333658974	0.150527159	-0.232671869	0.323556027
mir-148a-3p	mir-7-2-5p	0.333658974	0.150527159	-0.232671869	0.323556027
mir-182-5p	let-7f-2-5p	-0.407832345	0.074263676	-0.768614276	7.52E-05
mir-182-5p	let-7f-1-5p	-0.407832345	0.074263676	-0.768614276	7.52E-05
mir-16-2-5p	mir-148b-3p	0.483940655	0.030614918	-0.05537027	0.816649588
mir-148b-3p	mir-16-1-5p	0.483940655	0.030614918	-0.05537027	0.816649588
mir-26b-5p	norepi	0.171793381	0.468927312	-0.388451712	0.090537494
mir-106b-3p	mir-185-5p	0.90983743	2.69E-08	0.736930059	0.000210249
mir-182-5p	mir-340-5p	-0.291919741	0.211714337	-0.70808311	0.000477003
let-7i-5p	mir-125b-1-5p	-0.197046057	0.405022024	0.365269024	0.113275494
let-7i-5p	mir-125b-2-5p	-0.197046057	0.405022024	0.365269024	0.113275494
mir-17-5p	mir-106b-3p	0.76432416	8.72E-05	0.400304158	0.080302465
mir-106a-5p	mir-194-2-5p	0.843439615	3.00E-06	0.572067089	0.008399264
mir-106a-5p	mir-194-1-5p	0.843439615	3.00E-06	0.572067089	0.008399264
mir-126-3p	mir-28-5p	0.745916612	0.000159442	0.913088382	1.95E-08
mir-423-3p	mir-374b-5p	0.476758843	0.033555596	0.800651073	2.23E-05
mir-21-5p	mir-1307-3p	0.368048494	0.110352045	0.747708849	0.000150682
mir-27b-3p	mir-3615	-0.277467743	0.236251475	-0.699427492	0.000598861
mir-423-3p	mir-629-5p	-0.379694787	0.09869282	-0.75341196	0.000125496
mir-27b-3p	mir-106a-5p	-0.208982332	0.37656628	-0.660084601	0.001539305
mir-92a-2-3p	norepi	-0.109859845	0.64474618	-0.598409888	0.005314141
mir-92a-1-3p	norepi	-0.109859845	0.64474618	-0.598409888	0.005314141
mir-22-3p	mir-486-2-5p	0.779716528	5.05E-05	0.433680893	0.056087513
mir-103a-2-3p	mir-7-3-5p	0.255162279	0.277592434	-0.308792286	0.185273311
mir-103a-2-3p	mir-7-1-5p	0.255162279	0.277592434	-0.308792286	0.185273311
mir-103a-2-3p	mir-7-2-5p	0.255162279	0.277592434	-0.308792286	0.185273311
mir-7-3-5p	mir-103a-1-3p	0.255162279	0.277592434	-0.308792286	0.185273311
mir-103a-1-3p	mir-7-1-5p	0.255162279	0.277592434	-0.308792286	0.185273311
mir-103a-1-3p	mir-7-2-5p	0.255162279	0.277592434	-0.308792286	0.185273311
mir-98-5p	mir-1307-3p	0.199675701	0.398654423	0.654155377	0.001754428
mir-182-5p	mir-15b-5p	0.522906591	0.017997383	0.000267887	0.999105667
mir-182-5p	mir-423-3p	-0.346100491	0.134968436	-0.735435533	0.000219915
mir-21-5p	mir-99b-5p	0.847402137	2.42E-06	0.583303075	0.006941315
mir-340-5p	mir-374b-5p	0.694454917	0.000680116	0.892853999	1.19E-07
mir-139-5p	cpeptide	0.158165491	0.505408247	-0.396571073	0.083428641
mir-148a-3p	mir-15a-5p	0.340263474	0.142119778	-0.220898285	0.349318984
mir-122-5p	mir-30c-1-5p	-0.290609731	0.213866211	0.272251869	0.245540094
mir-122-5p	mir-30c-2-5p	-0.290609731	0.213866211	0.272251869	0.245540094
let-7i-5p	mir-1307-3p	-0.360533404	0.118384095	0.19815035	0.402341277
mir-27b-3p	mir-30c-1-5p	0.432843659	0.056617246	0.778532047	5.27E-05
mir-27b-3p	mir-30c-2-5p	0.432843659	0.056617246	0.778532047	5.27E-05
mir-543	let-7d-3p	0.801283465	2.17E-05	0.480839726	0.031858778

mir-99b-5p	mir-375	0.095777575	0.687922491	0.587627108	0.006438705
mir-151a-3p	mir-28-5p	0.579104717	0.007459635	0.84505736	2.75E-06
let-7b-5p	mir-28-3p	-0.353954708	0.125751844	-0.73859162	0.000199931
mir-122-5p	mir-191-5p	-0.15463913	0.5150649	0.397915953	0.082292208
let-7d-5p	mir-375	-0.265517452	0.257874356	0.295627494	0.205701525
mir-146a-5p	mir-20b-5p	-0.228265007	0.333063192	-0.669013241	0.001257366
mir-101-1-3p	mir-451a	0.962240308	1.29E-11	0.885169354	2.16E-07
mir-451a	mir-101-2-3p	0.962240308	1.29E-11	0.885169354	2.16E-07
mir-484	mir-20b-5p	0.803556587	1.98E-05	0.487476034	0.029243583
mir-423-3p	mir-223-3p	0.465695699	0.038511844	0.793266202	3.01E-05
mir-146a-5p	mir-144-3p	-0.256530605	0.274934588	-0.68467869	0.000867306
mir-106a-5p	mir-378a-3p	0.861450009	1.07E-06	0.619442299	0.003583251
mir-192-5p	mir-15a-5p	0.776516206	5.68E-05	0.43175599	0.057311102
mir-144-3p	mir-122-5p	0.098806361	0.678551838	-0.442277472	0.050863855
mir-1307-3p	mir-744-5p	0.466346021	0.038205654	0.792928189	3.05E-05
mir-30c-1-5p	mir-221-3p	0.890863365	1.40E-07	0.692673308	0.000711413
mir-221-3p	mir-30c-2-5p	0.890863365	1.40E-07	0.692673308	0.000711413
mir-182-5p	mir-92a-2-3p	0.584691624	0.00677653	0.846184977	2.58E-06
mir-182-5p	mir-92a-1-3p	0.584691624	0.00677653	0.846184977	2.58E-06
mir-103a-2-3p	mir-126-5p	0.632364468	0.002773879	0.866322849	7.89E-07
mir-103a-1-3p	mir-126-5p	0.632364468	0.002773879	0.866322849	7.89E-07
mir-122-5p	let-7d-3p	-0.169070372	0.476108246	0.381535948	0.096935277
mir-28-3p	mir-363-3p	-0.250993381	0.285788318	-0.679912061	0.000973305
mir-182-5p	mir-17-5p	0.760326034	9.99E-05	0.400787571	0.079904089
let-7a-3-5p	mir-148a-3p	-0.411521144	0.071431524	0.134080932	0.573044445
let-7a-2-5p	mir-148a-3p	-0.411521144	0.071431524	0.134080932	0.573044445
let-7a-1-5p	mir-148a-3p	-0.411521144	0.071431524	0.134080932	0.573044445
mir-181b-2-5p	mir-451a	0.575024871	0.007993243	0.082528993	0.729412724
mir-181b-1-5p	mir-451a	0.575024871	0.007993243	0.082528993	0.729412724
mir-1307-3p	mir-223-3p	0.137641687	0.562803101	0.611177491	0.004196949
mir-19b-2-3p	mir-20b-5p	0.945053432	3.55E-10	0.837282132	4.14E-06
mir-19b-1-3p	mir-20b-5p	0.945053432	3.55E-10	0.837282132	4.14E-06
mir-192-5p	mir-106b-3p	0.845073616	2.74E-06	0.583379531	0.006932157
mir-451a	mir-122-5p	0.196178496	0.407134912	-0.355906319	0.123532982
mir-30e-5p	mir-20b-5p	0.827879259	6.62E-06	0.544586329	0.013037433
mir-148a-3p	mir-148b-3p	0.119549141	0.615652314	0.598412276	0.005313911
mir-425-5p	mir-486-2-5p	0.747317573	0.000152559	0.377426363	0.100890029
mir-3615	mir-148a-3p	0.407459092	0.074554849	-0.136167178	0.56703424
mir-199a-1-5p	mir-150-5p	0.070825016	0.76668253	-0.46086616	0.040845708
mir-150-5p	mir-199a-2-5p	0.070825016	0.76668253	-0.46086616	0.040845708
mir-7-3-5p	mir-99a-5p	0.363731086	0.11491684	-0.185234353	0.434299221
mir-7-1-5p	mir-99a-5p	0.363731086	0.11491684	-0.185234353	0.434299221
mir-7-2-5p	mir-99a-5p	0.363731086	0.11491684	-0.185234353	0.434299221
mir-150-5p	mir-20b-5p	0.031422638	0.895372092	0.537032236	0.014621514
mir-99b-5p	norepi	0.141695715	0.551242365	0.611246334	0.0041915
mir-148b-3p	mir-28-5p	0.135652793	0.56851355	0.607211552	0.004520915
mir-151a-5p	mir-126-5p	0.411230121	0.07165198	0.763718067	8.91E-05
mir-151a-3p	mir-486-2-5p	0.044447243	0.852392677	-0.480068024	0.032174387
mir-425-5p	mir-93-5p	0.882537701	2.62E-07	0.674894271	0.001096498
mir-744-5p	mir-148a-3p	-0.446808946	0.048264583	0.086375923	0.717284723
mir-15a-5p	mir-30e-5p	0.716546406	0.000378904	0.321738923	0.166565683
mir-126-3p	mir-148a-3p	-0.156136309	0.510954332	0.387841715	0.091088983
mir-21-5p	mir-125a-5p	0.850730065	2.01E-06	0.600128102	0.0051509
mir-584-5p	mir-148a-3p	-0.526096872	0.017185284	-0.019401827	0.93529254
mir-101-1-3p	norepi	0.086106851	0.718130933	-0.444935769	0.049326395
mir-101-2-3p	norepi	0.086106851	0.718130933	-0.444935769	0.049326395
mir-16-2-5p	mir-28-3p	-0.248277168	0.291207786	-0.673862666	0.001123389
mir-16-1-5p	mir-28-3p	-0.248277168	0.291207786	-0.673862666	0.001123389
mir-146a-5p	mir-1307-3p	0.504909835	0.02317103	0.807451775	1.68E-05
mir-148a-3p	mir-320a	0.301026615	0.197150371	-0.247836101	0.292093733
mir-361-5p	mir-98-5p	0.564799737	0.009468721	0.834732387	4.72E-06
mir-199a-2-3p	mir-148a-3p	-0.384888399	0.093793958	0.156262022	0.510609895
mir-148a-3p	mir-199a-1-3p	-0.384888399	0.093793958	0.156262022	0.510609895
mir-148a-3p	mir-199b-3p	-0.384888399	0.093793958	0.156262022	0.510609895

mir-584-5p	mir-23a-3p	0.35445679	0.125178319	0.732369225	0.000240943
mir-20a-5p	mir-20b-5p	0.921482518	8.08E-09	0.776118931	5.76E-05
mir-182-5p	mir-23a-3p	-0.323318619	0.164375454	-0.715548584	0.000389492
mir-146b-5p	mir-744-5p	0.762951348	9.14E-05	0.916382469	1.40E-08
mir-101-1-3p	mir-148b-3p	0.55336581	0.011373516	0.060920627	0.798615522
mir-148b-3p	mir-101-2-3p	0.55336581	0.011373516	0.060920627	0.798615522
mir-151a-3p	mir-20b-5p	0.010832084	0.963848718	-0.50014655	0.024720617
mir-25-3p	mir-320a	0.968548073	2.55E-12	0.906658291	3.63E-08
mir-27b-3p	mir-23a-3p	0.666041158	0.001345936	0.877133519	3.85E-07
mir-103a-2-3p	mir-21-5p	0.563286545	0.009704761	0.832761505	5.21E-06
mir-21-5p	mir-103a-1-3p	0.563286545	0.009704761	0.832761505	5.21E-06
mir-361-5p	mir-146a-5p	0.816693785	1.12E-05	0.936138035	1.33E-09
mir-361-5p	mir-629-5p	-0.585204937	0.00671643	-0.842345687	3.18E-06
mir-486-2-5p	mir-484	0.771453863	6.81E-05	0.435159503	0.055161184
mir-103a-2-3p	mir-125b-1-5p	-0.073980864	0.756580707	0.448481038	0.047331645
mir-103a-2-3p	mir-125b-2-5p	-0.073980864	0.756580707	0.448481038	0.047331645
mir-103a-1-3p	mir-125b-1-5p	-0.073980864	0.756580707	0.448481038	0.047331645
mir-103a-1-3p	mir-125b-2-5p	-0.073980864	0.756580707	0.448481038	0.047331645
mir-340-5p	let-7d-3p	0.922939999	6.86E-09	0.782685938	4.52E-05
mir-144-3p	mir-185-5p	0.930197842	2.89E-09	0.801847508	2.12E-05
mir-139-5p	mir-23a-3p	0.582077832	0.007089433	0.840031309	3.59E-06
mir-340-5p	let-7d-5p	0.920915418	8.60E-09	0.777768687	5.42E-05
mir-199a-2-3p	mir-30c-1-5p	0.899889399	6.64E-08	0.724257403	0.000305033
mir-199a-2-3p	mir-30c-2-5p	0.899889399	6.64E-08	0.724257403	0.000305033
mir-30c-1-5p	mir-199a-1-3p	0.899889399	6.64E-08	0.724257403	0.000305033
mir-30c-1-5p	mir-199b-3p	0.899889399	6.64E-08	0.724257403	0.000305033
mir-199a-1-3p	mir-30c-2-5p	0.899889399	6.64E-08	0.724257403	0.000305033
mir-199b-3p	mir-30c-2-5p	0.899889399	6.64E-08	0.724257403	0.000305033
let-7i-5p	mir-24-2-3p	0.095448219	0.688944149	0.572136997	0.008389477
let-7i-5p	mir-24-1-3p	0.095448219	0.688944149	0.572136997	0.008389477
mir-146b-5p	mir-151a-3p	0.699864762	0.000592127	0.89001093	1.49E-07
mir-26b-5p	mir-30e-5p	0.911042637	2.39E-08	0.752529315	0.000129139
mir-744-5p	mir-24-2-3p	0.64851683	0.001981835	0.868656254	6.79E-07
mir-744-5p	mir-24-1-3p	0.64851683	0.001981835	0.868656254	6.79E-07
mir-182-5p	mir-484	0.702290907	0.000555923	0.306992518	0.187981816
mir-27b-3p	mir-20b-5p	-0.239966823	0.308177777	-0.663571024	0.001423485
mir-186-5p	norepi	0.214965429	0.362738869	-0.323746256	0.163785966
mir-128-1-3p	mir-126-5p	0.539141337	0.014164444	0.819743184	9.72E-06
mir-128-2-3p	mir-126-5p	0.539141337	0.014164444	0.819743184	9.72E-06
mir-30d-5p	mir-148a-3p	-0.234043475	0.320630316	0.304210297	0.192221132
mir-144-3p	mir-151a-3p	-0.011891363	0.960316002	-0.511049462	0.021288358
mir-125b-1-5p	mir-15a-5p	0.34534883	0.135874803	-0.189705393	0.423088081
mir-15a-5p	mir-125b-2-5p	0.34534883	0.135874803	-0.189705393	0.423088081
mir-27b-3p	mir-375	0.468749803	0.037090252	-0.043564229	0.855295231
mir-423-3p	mir-106b-3p	-0.449292385	0.046883975	-0.776099877	5.76E-05
mir-122-5p	mir-486-2-5p	0.152901196	0.519856066	-0.376922096	0.101383262
mir-125b-1-5p	mir-10a-5p	0.421000887	0.064524341	0.761305099	9.66E-05
mir-10a-5p	mir-125b-2-5p	0.421000887	0.064524341	0.761305099	9.66E-05
mir-451a	mir-151a-3p	-0.011166178	0.962734436	-0.50811946	0.022171035
mir-10a-5p	norepi	-0.01004745	0.966465912	0.491800723	0.027632204
mir-103a-2-3p	mir-26b-5p	0.323407022	0.164253472	-0.209685123	0.374926866
mir-26b-5p	mir-103a-1-3p	0.323407022	0.164253472	-0.209685123	0.374926866
mir-375	mir-10a-5p	0.53911856	0.014169318	0.818133404	1.05E-05
mir-126-3p	mir-122-5p	0.01930739	0.935606874	0.513420325	0.020594768
mir-26a-2-5p	mir-125b-1-5p	-0.234341891	0.319995878	0.299495774	0.199550366
mir-26a-2-5p	mir-125b-2-5p	-0.234341891	0.319995878	0.299495774	0.199550366
mir-125b-1-5p	mir-26a-1-5p	-0.234341891	0.319995878	0.299495774	0.199550366
mir-26a-1-5p	mir-125b-2-5p	-0.234341891	0.319995878	0.299495774	0.199550366
mir-126-3p	mir-151a-5p	0.241492864	0.305017666	0.660371673	0.001529479
mir-103a-2-3p	mir-744-5p	0.39084467	0.088397727	0.744026314	0.00016915
mir-744-5p	mir-103a-1-3p	0.39084467	0.088397727	0.744026314	0.00016915
mir-103a-2-3p	mir-451a	0.166089904	0.484030769	-0.361636995	0.117179109
mir-451a	mir-103a-1-3p	0.166089904	0.484030769	-0.361636995	0.117179109
mir-98-5p	mir-28-3p	0.405383958	0.0761892	0.751503751	0.000133487

mir-148b-3p	mir-126-5p	0.644101902	0.002176657	0.864526653	8.84E-07
let-7b-5p	mir-106b-3p	0.970318379	1.52E-12	0.914155691	1.75E-08
mir-10b-5p	mir-423-5p	-0.299674201	0.199269637	0.231903207	0.325202551
mir-223-3p	mir-629-5p	-0.193909389	0.412689479	-0.630119125	0.002902388
mir-103a-2-3p	mir-1307-3p	0.051977768	0.827716863	0.534926162	0.015089693
mir-1307-3p	mir-103a-1-3p	0.051977768	0.827716863	0.534926162	0.015089693
mir-106a-5p	mir-199a-2-3p	-0.287743973	0.218623727	-0.686369073	0.000832139
mir-106a-5p	mir-199a-1-3p	-0.287743973	0.218623727	-0.686369073	0.000832139
mir-106a-5p	mir-199b-3p	-0.287743973	0.218623727	-0.686369073	0.000832139
mir-181a-2-5p	mir-21-5p	0.582227944	0.007071148	0.83670359	4.27E-06
mir-21-5p	mir-181a-1-5p	0.582227944	0.007071148	0.83670359	4.27E-06
let-7i-5p	mir-20b-5p	0.448685045	0.047218774	-0.060409306	0.800272938
mir-223-3p	mir-122-5p	-0.192243399	0.416793646	0.335158356	0.148588807
mir-15a-5p	mir-99a-5p	0.164943001	0.487096707	-0.359945582	0.119029535
mir-21-5p	mir-125b-1-5p	-0.21591077	0.360581045	0.312899599	0.179191235
mir-21-5p	mir-125b-2-5p	-0.21591077	0.360581045	0.312899599	0.179191235
mir-148b-3p	mir-23a-3p	0.003818486	0.987252592	0.497963	0.025457806
mir-148b-3p	mir-363-3p	0.410866024	0.071928507	-0.105724128	0.657320629
mir-148b-3p	mir-93-5p	0.439437937	0.052546332	-0.070685684	0.767129379
mir-1307-3p	mir-15b-5p	-0.491627823	0.027695255	0.003978384	0.986718842
mir-30c-1-5p	mir-30a-5p	-0.052935355	0.824589688	0.453353976	0.044691649
mir-30a-5p	mir-30c-2-5p	-0.052935355	0.824589688	0.453353976	0.044691649
mir-223-3p	mir-139-5p	0.565188594	0.009408825	0.828124109	6.54E-06
mir-451a	mir-28-3p	-0.312244931	0.180151455	-0.69863449	0.000611238
mir-146a-5p	mir-28-3p	0.552374479	0.011552296	0.822123123	8.70E-06
mir-92a-2-3p	mir-374b-5p	-0.325274428	0.161691309	-0.705691342	0.000508352
mir-374b-5p	mir-92a-1-3p	-0.325274428	0.161691309	-0.705691342	0.000508352
mir-181a-2-5p	mir-126-5p	0.630378075	0.002887319	0.857283695	1.37E-06
mir-126-5p	mir-181a-1-5p	0.630378075	0.002887319	0.857283695	1.37E-06
mir-26b-5p	mir-320a	0.934419228	1.68E-09	0.818230593	1.04E-05
mir-128-1-3p	mir-374b-5p	0.666034851	0.001346129	0.872618486	5.23E-07
mir-374b-5p	mir-128-2-3p	0.666034851	0.001346129	0.872618486	5.23E-07
mir-451a	norepi	-0.018032595	0.939850883	-0.506664822	0.02261991
mir-223-3p	mir-151a-3p	0.482069327	0.031360909	0.78784667	3.72E-05
mir-143-3p	mir-151a-5p	-0.096276334	0.68637634	0.416505841	0.06773382
mir-320a	mir-486-2-5p	0.957542231	3.65E-11	0.879926484	3.16E-07
mir-629-5p	mir-128-1-3p	-0.097154548	0.683656797	-0.563142027	0.009727552
mir-629-5p	mir-128-2-3p	-0.097154548	0.683656797	-0.563142027	0.009727552
mir-146a-5p	mir-24-2-3p	0.757351729	0.000110287	0.910409145	2.54E-08
mir-146a-5p	mir-24-1-3p	0.757351729	0.000110287	0.910409145	2.54E-08
mir-224-5p	norepi	-0.318472454	0.171157969	0.206365759	0.382705434
mir-629-5p	mir-185-5p	0.868184261	7.00E-07	0.657154256	0.001642674
mir-532-5p	mir-194-2-5p	0.83363679	4.98E-06	0.580206317	0.007320667
mir-532-5p	mir-194-1-5p	0.83363679	4.98E-06	0.580206317	0.007320667
mir-139-5p	mir-374b-5p	0.505891769	0.022861377	0.798283357	2.46E-05
mir-92a-2-3p	mir-224-5p	-0.259386683	0.26943825	-0.664776901	0.001385171
mir-224-5p	mir-92a-1-3p	-0.259386683	0.26943825	-0.664776901	0.001385171
mir-1307-3p	mir-148b-3p	-0.27553158	0.239672571	0.247292168	0.293188576
mir-98-5p	mir-224-5p	0.297329543	0.202979693	0.686762346	0.000824133
mir-20b-5p	norepi	0.119402618	0.616088328	-0.391774798	0.087576143
mir-28-5p	mir-99a-5p	0.051621954	0.828879476	0.526116087	0.017180483
mir-92a-2-3p	mir-221-3p	-0.791376298	3.24E-05	-0.922863946	6.92E-09
mir-221-3p	mir-92a-1-3p	-0.791376298	3.24E-05	-0.922863946	6.92E-09
mir-26b-5p	mir-28-3p	-0.189518218	0.423554291	-0.619756143	0.003561501
mir-192-5p	mir-185-5p	0.881413485	2.84E-07	0.690792273	0.000745772
mir-181a-2-5p	mir-122-5p	-0.256652233	0.274699108	0.263659322	0.261345409
mir-122-5p	mir-181a-1-5p	-0.256652233	0.274699108	0.263659322	0.261345409
let-7f-2-5p	mir-629-5p	-0.446477832	0.048450989	-0.766856041	8.00E-05
mir-629-5p	let-7f-1-5p	-0.446477832	0.048450989	-0.766856041	8.00E-05
mir-30c-1-5p	mir-30e-5p	-0.097702672	0.681961333	0.408887731	0.073444968
mir-30e-5p	mir-30c-2-5p	-0.097702672	0.681961333	0.408887731	0.073444968
mir-222-3p	mir-28-3p	-0.00529728	0.982316512	-0.490626393	0.028062674
mir-361-5p	mir-125b-1-5p	-0.202878347	0.390974269	0.31475927	0.176482585
mir-361-5p	mir-125b-2-5p	-0.202878347	0.390974269	0.31475927	0.176482585

mir-92a-2-3p	mir-24-2-3p	-0.754593396	0.000120757	-0.907734862	3.28E-08
mir-92a-2-3p	mir-24-1-3p	-0.754593396	0.000120757	-0.907734862	3.28E-08
mir-24-2-3p	mir-92a-1-3p	-0.754593396	0.000120757	-0.907734862	3.28E-08
mir-24-1-3p	mir-92a-1-3p	-0.754593396	0.000120757	-0.907734862	3.28E-08
mir-122-5p	mir-375	0.553832919	0.011290056	0.092675343	0.69756598
let-7i-5p	let-7b-5p	0.45681349	0.042887318	-0.03756078	0.87507481
mir-98-5p	mir-151a-3p	0.612627741	0.004083387	0.846573154	2.53E-06
mir-25-3p	mir-126-5p	0.325911197	0.160823972	-0.190210777	0.421830656
mir-27b-3p	mir-25-3p	-0.23548841	0.317565339	-0.647381041	0.002030503
mir-151a-3p	mir-363-3p	-0.040810046	0.864359844	-0.516350981	0.019762395
mir-222-3p	mir-185-5p	0.781948789	4.65E-05	0.918731697	1.09E-08
mir-423-3p	mir-128-1-3p	0.434135172	0.055801664	0.759579434	0.00010241
mir-423-3p	mir-128-2-3p	0.434135172	0.055801664	0.759579434	0.00010241
mir-24-2-3p	norepi	0.049395502	0.836162043	0.522311283	0.018152262
mir-24-1-3p	norepi	0.049395502	0.836162043	0.522311283	0.018152262
let-7g-5p	norepi	0.575786389	0.007891345	0.12556257	0.597866207
mir-224-5p	mir-125b-1-5p	0.126002698	0.596572852	0.57599067	0.007864192
mir-224-5p	mir-125b-2-5p	0.126002698	0.596572852	0.57599067	0.007864192
mir-146a-5p	mir-148a-3p	-0.373341398	0.104936162	0.136324005	0.56658356
mir-532-5p	norepi	-0.053706001	0.822074849	-0.524828814	0.017504485
mir-182-5p	mir-192-5p	0.759799933	0.000101658	0.435447073	0.054982386
mir-423-5p	mir-30e-5p	0.899614262	6.80E-08	0.73596637	0.000216439
mir-375	cpeptide	0.354101453	0.125584025	-0.156854697	0.508987543
mir-125a-5p	mir-375	0.038232959	0.872856414	0.512601018	0.02083239
mir-23a-3p	norepi	-0.050206076	0.833509196	0.444155301	0.049774048
mir-423-3p	mir-139-5p	0.441522965	0.051306839	0.762323749	9.34E-05
mir-148b-3p	mir-107	0.481357306	0.03164846	-0.002809648	0.990620269
mir-16-2-5p	norepi	0.084250127	0.723978758	-0.41617231	0.067976653
mir-16-1-5p	norepi	0.084250127	0.723978758	-0.41617231	0.067976653
mir-423-3p	mir-143-3p	-0.232685218	0.323527476	0.28170005	0.228883586
mir-25-3p	mir-122-5p	0.126242958	0.595867318	-0.379591271	0.09879232
mir-122-5p	mir-148a-3p	0.7702331	7.11E-05	0.457871423	0.042346929
mir-21-5p	mir-148b-3p	0.298409135	0.201265741	0.682625034	0.000911712
let-7i-5p	mir-15a-5p	0.192152799	0.417017466	-0.319306305	0.169977528
let-7i-5p	mir-181a-2-5p	0.537643417	0.01448786	0.809666034	1.52E-05
let-7i-5p	mir-181a-1-5p	0.537643417	0.01448786	0.809666034	1.52E-05
mir-374b-5p	mir-126-5p	0.754551949	0.000120921	0.906621813	3.64E-08
mir-320a	cpeptide	-0.32456652	0.162659325	0.18542786	0.43381078
mir-30d-5p	mir-128-1-3p	0.7861335	3.97E-05	0.919443617	1.01E-08
mir-30d-5p	mir-128-2-3p	0.7861335	3.97E-05	0.919443617	1.01E-08
mir-199a-2-3p	mir-363-3p	-0.271594711	0.246726743	-0.665519171	0.001362021
mir-199a-1-3p	mir-363-3p	-0.271594711	0.246726743	-0.665519171	0.001362021
mir-199b-3p	mir-363-3p	-0.271594711	0.246726743	-0.665519171	0.001362021
mir-744-5p	norepi	0.184204283	0.436904144	0.610926272	0.004216884
mir-103a-2-3p	mir-363-3p	0.166849402	0.482005729	-0.341030672	0.141164974
mir-103a-1-3p	mir-363-3p	0.166849402	0.482005729	-0.341030672	0.141164974
mir-629-5p	mir-374b-5p	-0.101130048	0.671393245	-0.554694423	0.01113743
mir-3615	mir-148b-3p	0.13527446	0.569602665	-0.368961803	0.109403418
mir-744-5p	mir-28-3p	0.505165848	0.023089978	0.793001709	3.04E-05
mir-144-3p	mir-320a	0.893859466	1.10E-07	0.724793868	0.000300387
mir-199a-2-3p	mir-28-3p	0.555697467	0.01096184	0.817645374	1.07E-05
mir-199a-1-3p	mir-28-3p	0.555697467	0.01096184	0.817645374	1.07E-05
mir-199b-3p	mir-28-3p	0.555697467	0.01096184	0.817645374	1.07E-05
mir-143-3p	mir-320a	-0.279763984	0.232235244	-0.669800311	0.00123475
mir-25-3p	norepi	0.098623753	0.679115534	-0.400302307	0.080303993
mir-361-5p	mir-23a-3p	0.727842321	0.000275114	0.895072373	9.95E-08
mir-19b-2-3p	norepi	0.104557093	0.660885221	-0.394785915	0.084954988
mir-19b-1-3p	norepi	0.104557093	0.660885221	-0.394785915	0.084954988
mir-181a-2-5p	mir-128-1-3p	0.614338683	0.003952693	0.844871409	2.77E-06
mir-181a-2-5p	mir-128-2-3p	0.614338683	0.003952693	0.844871409	2.77E-06
mir-128-1-3p	mir-181a-1-5p	0.614338683	0.003952693	0.844871409	2.77E-06
mir-128-2-3p	mir-181a-1-5p	0.614338683	0.003952693	0.844871409	2.77E-06
mir-148a-3p	mir-150-5p	0.354864427	0.124714048	-0.14980912	0.528431935
mir-26b-5p	mir-122-5p	0.003570923	0.988078983	-0.476391762	0.033711619

mir-103a-2-3p	mir-3615	0.108573274	0.648648195	-0.390886912	0.088360292
mir-3615	mir-103a-1-3p	0.108573274	0.648648195	-0.390886912	0.088360292
mir-199a-2-3p	mir-221-3p	0.894770282	1.02E-07	0.961623315	1.49E-11
mir-199a-1-3p	mir-221-3p	0.894770282	1.02E-07	0.961623315	1.49E-11
mir-199b-3p	mir-221-3p	0.894770282	1.02E-07	0.961623315	1.49E-11
mir-122-5p	let-7b-5p	0.076340162	0.749053286	-0.417696341	0.06687237
mir-24-2-3p	mir-139-5p	0.688251672	0.0007944	0.87768537	3.70E-07
mir-139-5p	mir-24-1-3p	0.688251672	0.0007944	0.87768537	3.70E-07
mir-361-5p	mir-148a-3p	-0.239444739	0.309263428	0.269318333	0.250865595
mir-103a-2-3p	mir-423-5p	0.673656068	0.001128841	0.289099333	0.21636508
mir-423-5p	mir-103a-1-3p	0.673656068	0.001128841	0.289099333	0.21636508
mir-144-3p	mir-192-5p	0.888728405	1.65E-07	0.71445479	0.000401387
mir-146b-5p	mir-28-5p	0.588717164	0.006316833	0.832188634	5.36E-06
let-7i-5p	mir-23a-3p	0.129433856	0.586530284	0.57139141	0.008494337
mir-181a-2-5p	mir-224-5p	0.438711253	0.052983654	0.757373556	0.000110208
mir-224-5p	mir-181a-1-5p	0.438711253	0.052983654	0.757373556	0.000110208
mir-126-3p	let-7a-3-5p	0.750440166	0.000138127	0.90393355	4.65E-08
mir-126-3p	let-7a-2-5p	0.750440166	0.000138127	0.90393355	4.65E-08
mir-126-3p	let-7a-1-5p	0.750440166	0.000138127	0.90393355	4.65E-08
mir-27b-3p	mir-221-3p	0.612646789	0.004081912	0.843261848	3.02E-06
mir-186-5p	mir-20b-5p	0.899912583	6.62E-08	0.740971783	0.000185907
mir-361-5p	norepi	0.063380121	0.790654929	0.524545847	0.017576358
mir-181b-2-5p	copeptin	0.151929947	0.52254274	-0.350387295	0.129880808
mir-181b-1-5p	copeptin	0.151929947	0.52254274	-0.350387295	0.129880808
mir-182-5p	mir-1307-3p	-0.534062258	0.015285189	-0.805752342	1.80E-05
mir-1307-3p	mir-150-5p	0.18307959	0.439757722	-0.321860257	0.166396748
mir-126-3p	mir-28-3p	0.421646965	0.064072629	0.748065024	0.000148992
mir-22-3p	mir-30c-1-5p	-0.111455293	0.639919783	0.385775834	0.09297504
mir-22-3p	mir-30c-2-5p	-0.111455293	0.639919783	0.385775834	0.09297504
mir-146a-5p	mir-106a-5p	-0.380462438	0.097957231	-0.72557396	0.000293738
mir-629-5p	mir-320a	0.860534679	1.13E-06	0.650821419	0.001886078
mir-148b-3p	cpeptide	-0.551625817	0.011688814	-0.101916407	0.668976825
mir-182-5p	mir-106a-5p	0.737112085	0.000209097	0.898095382	7.74E-08
mir-17-5p	mir-25-3p	0.87708876	3.86E-07	0.688444105	0.000790625
mir-126-3p	let-7d-5p	0.64288657	0.002233011	0.856717724	1.42E-06
mir-103a-2-3p	mir-223-3p	0.582736496	0.00700949	0.828614046	6.39E-06
mir-223-3p	mir-103a-1-3p	0.582736496	0.00700949	0.828614046	6.39E-06
mir-151a-3p	mir-151a-5p	0.157881952	0.506181461	0.589099087	0.006274583
let-7d-5p	mir-28-3p	0.430778446	0.057940197	0.752119841	0.00013086
let-7d-5p	mir-139-5p	0.55597439	0.01091376	0.815695806	1.17E-05
mir-320a	mir-28-3p	-0.099270854	0.677118727	-0.548611888	0.012251676
mir-126-3p	mir-98-5p	0.685429253	0.000851539	0.875434412	4.33E-07
mir-22-3p	mir-26b-5p	0.881261563	2.87E-07	0.698743556	0.000609523
mir-126-3p	mir-125b-1-5p	-0.010728672	0.964193636	0.466489834	0.038138198
mir-126-3p	mir-125b-2-5p	-0.010728672	0.964193636	0.466489834	0.038138198
mir-146b-5p	mir-224-5p	0.237132473	0.314099398	0.639765379	0.002383349
mir-146b-5p	let-7a-3-5p	0.802859249	2.04E-05	0.924997703	5.42E-09
mir-146b-5p	let-7a-2-5p	0.802859249	2.04E-05	0.924997703	5.42E-09
mir-146b-5p	let-7a-1-5p	0.802859249	2.04E-05	0.924997703	5.42E-09
mir-584-5p	mir-151a-3p	0.340139735	0.142274199	0.701234245	0.000571453
mir-122-5p	mir-363-3p	0.100570933	0.673113255	-0.391936938	0.087433502
mir-148b-3p	mir-185-5p	0.567769562	0.009019041	0.128750284	0.588525292
mir-103a-2-3p	mir-374b-5p	0.698066633	0.000620233	0.880529352	3.03E-07
mir-103a-1-3p	mir-374b-5p	0.698066633	0.000620233	0.880529352	3.03E-07
let-7f-2-5p	mir-1307-3p	0.318068947	0.171731203	0.687507148	0.000809148
mir-1307-3p	let-7f-1-5p	0.318068947	0.171731203	0.687507148	0.000809148
mir-144-3p	mir-584-5p	-0.221344121	0.348322314	-0.627559638	0.00305488
mir-151a-3p	let-7b-5p	-0.125976834	0.596648822	-0.564030706	0.00958809
mir-182-5p	mir-30e-5p	0.701652846	0.000565257	0.344073366	0.137422588
mir-1307-3p	let-7d-5p	0.31231246	0.180052248	0.683067174	0.000901993
mir-27b-3p	mir-629-5p	-0.488378103	0.028901528	-0.780099669	4.98E-05
mir-584-5p	mir-629-5p	-0.225829777	0.338386735	-0.629205266	0.00295609
mir-486-2-5p	mir-126-5p	0.200972498	0.395534674	-0.297089108	0.203362723
mir-103a-2-3p	mir-99a-5p	0.017698581	0.940963131	0.483307376	0.030865796

mir-103a-1-3p	mir-99a-5p	0.017698581	0.940963131	0.483307376	0.030865796
mir-486-2-5p	mir-15b-5p	0.602473225	0.004934788	0.185556695	0.433485744
mir-26a-2-5p	mir-629-5p	-0.422023054	0.063810783	-0.743940212	0.000169604
mir-629-5p	mir-26a-1-5p	-0.422023054	0.063810783	-0.743940212	0.000169604
let-7a-3-5p	mir-340-5p	0.923616252	6.36E-09	0.801965248	2.11E-05
mir-340-5p	let-7a-2-5p	0.923616252	6.36E-09	0.801965248	2.11E-05
mir-340-5p	let-7a-1-5p	0.923616252	6.36E-09	0.801965248	2.11E-05
mir-423-3p	mir-125b-1-5p	-0.512058199	0.02099102	-0.05659014	0.812678074
mir-423-3p	mir-125b-2-5p	-0.512058199	0.02099102	-0.05659014	0.812678074
let-7d-3p	norepi	0.252910612	0.282000762	0.64535103	0.002119979
mir-30e-5p	mir-93-5p	0.898273513	7.62E-08	0.74210367	0.000179539
mir-451a	mir-99a-5p	0.398126581	0.082115269	-0.086270885	0.717615022
mir-361-5p	mir-1307-3p	0.513494408	0.020573389	0.79141735	3.24E-05
mir-146b-5p	let-7d-5p	0.78038803	4.93E-05	0.914438848	1.71E-08
mir-29a-3p	mir-423-3p	-0.421830141	0.063944995	0.057137397	0.810897804
mir-27b-3p	mir-451a	-0.209583322	0.375164089	-0.616813214	0.003769813
mir-375	let-7d-3p	-0.050892793	0.83126306	0.426880005	0.060501261
mir-103a-2-3p	mir-224-5p	0.30316076	0.193836872	0.674990174	0.001094026
mir-224-5p	mir-103a-1-3p	0.30316076	0.193836872	0.674990174	0.001094026
mir-148a-3p	let-7d-3p	-0.355571401	0.123911765	0.134017459	0.573227734
mir-1307-3p	mir-28-5p	0.314928815	0.176237035	0.681517852	0.000936439
mir-199a-2-3p	mir-106b-3p	-0.362689271	0.116038367	-0.709212029	0.000462786
mir-106b-3p	mir-199a-1-3p	-0.362689271	0.116038367	-0.709212029	0.000462786
mir-106b-3p	mir-199b-3p	-0.362689271	0.116038367	-0.709212029	0.000462786
mir-182-5p	mir-27a-3p	0.511718372	0.021090817	0.059403005	0.803537191
mir-143-3p	copeptin	-0.09232199	0.698667269	0.390981338	0.088276653
mir-98-5p	mir-93-5p	-0.000150266	0.999498341	-0.466442052	0.0381606
mir-182-5p	mir-20a-5p	0.833185461	5.10E-06	0.600118326	0.005151816
mir-98-5p	mir-106a-5p	-0.19639745	0.406601098	-0.606754872	0.004559511
let-7d-5p	mir-10a-5p	0.166520027	0.482883419	0.586791309	0.006533452
let-7b-5p	mir-150-5p	-0.018462996	0.938417822	0.451162479	0.045864521
let-7a-3-5p	mir-375	-0.041050699	0.863567136	0.432837515	0.056621148
let-7a-2-5p	mir-375	-0.041050699	0.863567136	0.432837515	0.056621148
let-7a-1-5p	mir-375	-0.041050699	0.863567136	0.432837515	0.056621148
mir-151a-3p	let-7d-5p	0.613039905	0.004051581	0.839097702	3.77E-06
mir-25-3p	mir-151a-3p	0.074345775	0.755415045	-0.405173615	0.076356341
mir-122-5p	mir-139-5p	-0.144046068	0.544589223	0.344244925	0.13721368
mir-144-3p	mir-28-3p	-0.254663684	0.278564869	-0.643139556	0.002221181
mir-191-5p	mir-148a-3p	-0.248126123	0.291510996	0.244367798	0.299117882
mir-144-3p	mir-99a-5p	0.247015316	0.293746796	-0.244896118	0.298041316
mir-101-1-3p	mir-320a	0.945715314	3.19E-10	0.85851796	1.28E-06
mir-320a	mir-101-2-3p	0.945715314	3.19E-10	0.85851796	1.28E-06
mir-199a-1-5p	mir-143-3p	0.096941645	0.684315746	0.53635062	0.01477174
mir-143-3p	mir-199a-2-5p	0.096941645	0.684315746	0.53635062	0.01477174
mir-27b-3p	mir-191-5p	0.49089243	0.027964695	0.777306158	5.52E-05
let-7f-2-5p	mir-27a-3p	-0.167208759	0.481049051	0.320740283	0.167960576
mir-27a-3p	let-7f-1-5p	-0.167208759	0.481049051	0.320740283	0.167960576
let-7d-5p	mir-30a-5p	-0.104556977	0.660885573	0.376477904	0.101819185
mir-126-3p	mir-1307-3p	0.318186331	0.171564308	0.680596858	0.000957436
mir-126-3p	mir-30d-5p	0.548570235	0.012259606	0.806468003	1.75E-05
mir-181b-2-5p	mir-15a-5p	0.473213649	0.03508623	0.01372032	0.954218101
mir-181b-1-5p	mir-15a-5p	0.473213649	0.03508623	0.01372032	0.954218101
mir-194-2-5p	mir-378a-3p	0.757809154	0.000108629	0.903510099	4.83E-08
mir-194-1-5p	mir-378a-3p	0.757809154	0.000108629	0.903510099	4.83E-08
mir-744-5p	mir-15a-5p	-0.76149491	9.60E-05	-0.905053373	4.20E-08
mir-182-5p	mir-139-5p	-0.249261046	0.289237482	-0.637678516	0.002488505
let-7f-2-5p	mir-146a-5p	0.814798717	1.22E-05	0.927558557	4.00E-09
mir-146a-5p	let-7f-1-5p	0.814798717	1.22E-05	0.927558557	4.00E-09
mir-24-2-3p	mir-30d-5p	0.482694947	0.031109943	0.772184442	6.63E-05
mir-30d-5p	mir-24-1-3p	0.482694947	0.031109943	0.772184442	6.63E-05
mir-425-5p	mir-15a-5p	0.606310171	0.004597356	0.201232375	0.394911105
mir-148a-3p	mir-221-3p	-0.293148457	0.209709036	0.194509497	0.411216494
mir-1307-3p	mir-23a-3p	0.425636474	0.061335946	0.741327134	0.000183887
mir-144-3p	mir-106b-3p	0.914939983	1.62E-08	0.784984817	4.15E-05

mir-340-5p	mir-23a-3p	0.488365749	0.028906191	0.774796498	6.04E-05
mir-15a-5p	mir-126-5p	0.012434106	0.958506215	-0.45084704	0.046035272
mir-486-2-5p	mir-150-5p	0.048678729	0.838509321	0.497885462	0.025484299
mir-27b-3p	mir-15b-5p	-0.17425354	0.462486971	0.311031478	0.181940484
mir-532-5p	mir-99a-5p	0.276036088	0.238778075	-0.21090278	0.372095976
mir-126-3p	mir-99a-5p	0.147453969	0.535007779	0.56898418	0.008840215
let-7d-5p	mir-20b-5p	-0.227257606	0.335259402	-0.621788919	0.003423263
mir-3615	mir-99a-5p	0.460287621	0.041132464	0.001344819	0.995510382
mir-98-5p	mir-486-2-5p	-0.112098958	0.63797655	-0.543324758	0.01329188
mir-92a-2-3p	let-7d-5p	-0.589800527	0.006197591	-0.825328949	7.48E-06
let-7d-5p	mir-92a-1-3p	-0.589800527	0.006197591	-0.825328949	7.48E-06
mir-101-1-3p	mir-99a-5p	0.371419263	0.106880183	-0.104914854	0.659791721
mir-101-2-3p	mir-99a-5p	0.371419263	0.106880183	-0.104914854	0.659791721
mir-101-1-3p	mir-192-5p	0.904851239	4.28E-08	0.762940764	9.14E-05
mir-192-5p	mir-101-2-3p	0.904851239	4.28E-08	0.762940764	9.14E-05
mir-222-3p	mir-532-5p	0.615401037	0.0038733	0.837359274	4.12E-06
let-7a-3-5p	mir-151a-3p	0.689853201	0.000763444	0.872194009	5.38E-07
let-7a-2-5p	mir-151a-3p	0.689853201	0.000763444	0.872194009	5.38E-07
let-7a-1-5p	mir-151a-3p	0.689853201	0.000763444	0.872194009	5.38E-07
mir-92a-2-3p	mir-128-1-3p	-0.515575136	0.019980097	-0.787395911	3.78E-05
mir-92a-2-3p	mir-128-2-3p	-0.515575136	0.019980097	-0.787395911	3.78E-05
mir-128-1-3p	mir-92a-1-3p	-0.515575136	0.019980097	-0.787395911	3.78E-05
mir-128-2-3p	mir-92a-1-3p	-0.515575136	0.019980097	-0.787395911	3.78E-05
mir-26b-5p	mir-425-5p	0.803684491	1.97E-05	0.547474977	0.012469606
mir-29a-3p	mir-340-5p	-0.096459151	0.68580991	0.377699464	0.100623634
mir-222-3p	let-7b-5p	0.68829011	0.000793645	0.871381221	5.68E-07
let-7a-3-5p	mir-1307-3p	0.331509531	0.153336495	0.684976041	0.00086103
mir-1307-3p	let-7a-2-5p	0.331509531	0.153336495	0.684976041	0.00086103
mir-1307-3p	let-7a-1-5p	0.331509531	0.153336495	0.684976041	0.00086103
mir-182-5p	mir-194-2-5p	0.721562867	0.000329308	0.394398699	0.08528877
mir-182-5p	mir-194-1-5p	0.721562867	0.000329308	0.394398699	0.08528877
mir-30d-5p	mir-27a-3p	-0.032983367	0.890204724	0.430370308	0.058204398
mir-10a-5p	let-7d-3p	0.397400374	0.082726513	0.722919123	0.000316891
let-7i-5p	mir-30c-1-5p	0.229927837	0.329456713	0.620579898	0.003504935
let-7i-5p	mir-30c-2-5p	0.229927837	0.329456713	0.620579898	0.003504935
mir-146b-5p	mir-125b-1-5p	-0.111355665	0.640220764	0.362705666	0.116020657
mir-146b-5p	mir-125b-2-5p	-0.111355665	0.640220764	0.362705666	0.116020657
mir-143-3p	mir-194-2-5p	-0.067876297	0.776154237	-0.507551765	0.022345366
mir-143-3p	mir-194-1-5p	-0.067876297	0.776154237	-0.507551765	0.022345366
mir-103a-2-3p	let-7g-5p	0.83524847	4.59E-06	0.6131313	0.004044555
mir-103a-1-3p	let-7g-5p	0.83524847	4.59E-06	0.6131313	0.004044555
mir-92a-2-3p	mir-148b-3p	0.088619658	0.710240772	-0.381791272	0.096693366
mir-148b-3p	mir-92a-1-3p	0.088619658	0.710240772	-0.381791272	0.096693366
let-7i-5p	mir-144-3p	0.515194299	0.020087655	0.078900547	0.740908961
mir-98-5p	mir-25-3p	-0.082931392	0.728141148	-0.518150711	0.019264668
mir-19b-2-3p	mir-25-3p	0.941046002	6.59E-10	0.850096029	2.08E-06
mir-25-3p	mir-19b-1-3p	0.941046002	6.59E-10	0.850096029	2.08E-06
mir-126-5p	let-7g-5p	0.413012286	0.070309908	0.730362705	0.000255612
mir-21-5p	mir-126-5p	0.463138167	0.039734511	0.757978395	0.000108021
mir-122-5p	mir-185-5p	0.049616574	0.83543835	-0.41380991	0.069715391
mir-98-5p	let-7a-3-5p	0.879433339	3.27E-07	0.952923725	9.09E-11
mir-98-5p	let-7a-2-5p	0.879433339	3.27E-07	0.952923725	9.09E-11
mir-98-5p	let-7a-1-5p	0.879433339	3.27E-07	0.952923725	9.09E-11
mir-340-5p	mir-151a-5p	0.475510956	0.034088303	0.764151118	8.77E-05
mir-224-5p	mir-151a-5p	-0.123011531	0.605385466	0.349839135	0.130523671
mir-1307-3p	mir-191-5p	0.309032488	0.184913831	0.668681278	0.001267008
mir-148a-3p	mir-28-5p	-0.197959727	0.40280333	0.280520357	0.230922069
mir-146a-5p	norepi	0.203001162	0.390681395	0.600949006	0.00507438
mir-10b-5p	mir-15a-5p	-0.208335571	0.378078558	0.26994888	0.249714745
mir-26a-2-5p	mir-181a-2-5p	0.679418728	0.000984874	0.865833059	8.14E-07
mir-26a-2-5p	mir-181a-1-5p	0.679418728	0.000984874	0.865833059	8.14E-07
mir-181a-2-5p	mir-26a-1-5p	0.679418728	0.000984874	0.865833059	8.14E-07
mir-26a-1-5p	mir-181a-1-5p	0.679418728	0.000984874	0.865833059	8.14E-07
mir-423-3p	mir-23a-3p	0.336900051	0.146359141	0.685120794	0.000857989

mir-223-3p	mir-151a-5p	0.602847688	0.004900983	0.829058262	6.25E-06
mir-199a-1-5p	mir-486-2-5p	-0.52598898	0.01721226	-0.790045327	3.41E-05
mir-486-2-5p	mir-199a-2-5p	-0.52598898	0.01721226	-0.790045327	3.41E-05
mir-532-5p	mir-148a-3p	0.368406627	0.109979359	-0.100042134	0.674741441
mir-3615	mir-584-5p	-0.442762906	0.050580399	-0.745334451	0.00016238
mir-148a-3p	mir-93-5p	0.243350552	0.30119741	-0.233962114	0.32080342
mir-126-3p	let-7i-5p	0.287625251	0.218822306	0.65412624	0.001755544
mir-30c-1-5p	mir-99b-5p	0.748571519	0.000146615	0.44882436	0.047141814
mir-99b-5p	mir-30c-2-5p	0.748571519	0.000146615	0.44882436	0.047141814
mir-181b-2-5p	mir-363-3p	0.552146162	0.011593792	0.134317186	0.572362443
mir-181b-1-5p	mir-363-3p	0.552146162	0.011593792	0.134317186	0.572362443
mir-182-5p	mir-629-5p	0.777527601	5.47E-05	0.909532203	2.77E-08
mir-139-5p	let-7g-5p	0.150837862	0.525571431	0.563142496	0.009727478
mir-27b-3p	let-7d-3p	0.575148117	0.007976679	0.814514403	1.23E-05
let-7f-2-5p	mir-126-5p	0.415572504	0.068414995	0.729389118	0.000263001
mir-126-5p	let-7f-1-5p	0.415572504	0.068414995	0.729389118	0.000263001
let-7a-3-5p	mir-24-2-3p	0.690289903	0.000755182	0.870109809	6.18E-07
let-7a-3-5p	mir-24-1-3p	0.690289903	0.000755182	0.870109809	6.18E-07
let-7a-2-5p	mir-24-2-3p	0.690289903	0.000755182	0.870109809	6.18E-07
let-7a-2-5p	mir-24-1-3p	0.690289903	0.000755182	0.870109809	6.18E-07
let-7a-1-5p	mir-24-2-3p	0.690289903	0.000755182	0.870109809	6.18E-07
let-7a-1-5p	mir-24-1-3p	0.690289903	0.000755182	0.870109809	6.18E-07
mir-340-5p	mir-151a-3p	0.67690571	0.001045625	0.863756742	9.27E-07
mir-181b-2-5p	mir-20b-5p	0.57835604	0.007555308	0.173659983	0.464036677
mir-181b-1-5p	mir-20b-5p	0.57835604	0.007555308	0.173659983	0.464036677
mir-191-5p	mir-20b-5p	-0.044661362	0.85168912	-0.484738168	0.030301255
mir-15a-5p	mir-374b-5p	-0.185351318	0.43400395	-0.586213681	0.006599599
let-7b-5p	mir-30e-5p	0.80290285	2.03E-05	0.552857121	0.011464973
let-7b-5p	mir-320a	0.92468136	5.62E-09	0.813221103	1.30E-05
mir-181a-2-5p	mir-1307-3p	0.113260795	0.634474706	0.53516331	0.015036381
mir-1307-3p	mir-181a-1-5p	0.113260795	0.634474706	0.53516331	0.015036381
mir-181a-2-5p	mir-191-5p	0.73450038	0.000226155	0.889956275	1.50E-07
mir-191-5p	mir-181a-1-5p	0.73450038	0.000226155	0.889956275	1.50E-07
mir-144-3p	let-7d-5p	-0.202142303	0.392732039	-0.596277574	0.005522626
let-7a-3-5p	mir-27a-3p	-0.099641991	0.675974417	0.36444841	0.114149171
let-7a-2-5p	mir-27a-3p	-0.099641991	0.675974417	0.36444841	0.114149171
let-7a-1-5p	mir-27a-3p	-0.099641991	0.675974417	0.36444841	0.114149171
mir-423-3p	mir-1307-3p	0.462015486	0.040280628	0.753792593	0.000123952
mir-30d-5p	mir-148b-3p	0.496966515	0.025799947	0.772598297	6.54E-05
mir-744-5p	mir-139-5p	0.498215723	0.025371607	0.773070335	6.43E-05
mir-451a	mir-199a-2-3p	-0.297598894	0.202551166	-0.657193852	0.00164124
mir-451a	mir-199a-1-3p	-0.297598894	0.202551166	-0.657193852	0.00164124
mir-451a	mir-199b-3p	-0.297598894	0.202551166	-0.657193852	0.00164124
mir-16-2-5p	mir-122-5p	0.089925602	0.70615126	-0.371367054	0.106933348
mir-122-5p	mir-16-1-5p	0.089925602	0.70615126	-0.371367054	0.106933348
mir-122-5p	mir-106b-3p	0.042810055	0.857775694	-0.411414901	0.071511946
mir-20b-5p	mir-15b-5p	0.627463677	0.003060724	0.251723597	0.284342065
mir-543	mir-99a-5p	0.089930992	0.706134397	0.515188781	0.020089217
mir-151a-3p	mir-374b-5p	0.546193131	0.012719062	0.797655027	2.52E-05
mir-222-3p	mir-122-5p	0.123124637	0.605051266	-0.340792308	0.141461142
mir-103a-2-3p	mir-28-5p	0.684720509	0.000866421	0.86590072	8.10E-07
mir-103a-1-3p	mir-28-5p	0.684720509	0.000866421	0.86590072	8.10E-07
mir-103a-2-3p	mir-320a	0.38099709	0.097447279	-0.07702292	0.746878959
mir-103a-1-3p	mir-320a	0.38099709	0.097447279	-0.07702292	0.746878959
let-7i-5p	mir-27a-3p	0.162701982	0.493115007	0.566525858	0.009205192
mir-146b-5p	mir-423-3p	0.554551007	0.011162721	0.801530702	2.15E-05
mir-122-5p	mir-543	0.082900877	0.72823755	0.508515691	0.022049998
mir-103a-2-3p	mir-16-2-5p	0.255490467	0.276953507	-0.212645312	0.368065959
mir-103a-2-3p	mir-16-1-5p	0.255490467	0.276953507	-0.212645312	0.368065959
mir-16-2-5p	mir-103a-1-3p	0.255490467	0.276953507	-0.212645312	0.368065959
mir-103a-1-3p	mir-16-1-5p	0.255490467	0.276953507	-0.212645312	0.368065959
mir-128-1-3p	mir-106b-3p	0.049120403	0.837062778	-0.40366682	0.077561652
mir-106b-3p	mir-128-2-3p	0.049120403	0.837062778	-0.40366682	0.077561652
mir-222-3p	mir-93-5p	0.761133387	9.72E-05	0.900635612	6.22E-08

mir-125a-5p	mir-10a-5p	0.352669297	0.127228679	0.688486448	0.000789797
mir-16-2-5p	mir-25-3p	0.974763242	3.60E-13	0.93583948	1.38E-09
mir-25-3p	mir-16-1-5p	0.974763242	3.60E-13	0.93583948	1.38E-09
mir-10b-5p	mir-126-5p	0.259306424	0.269591755	-0.207799232	0.379335229
mir-27b-3p	mir-7-3-5p	-0.240695934	0.306665482	-0.617845332	0.003695636
mir-27b-3p	mir-7-1-5p	-0.240695934	0.306665482	-0.617845332	0.003695636
mir-27b-3p	mir-7-2-5p	-0.240695934	0.306665482	-0.617845332	0.003695636
mir-30a-5p	mir-23a-3p	0.104991658	0.659557057	0.523516274	0.017839861
mir-24-2-3p	let-7d-5p	0.5604583	0.010158757	0.803807324	1.96E-05
let-7d-5p	mir-24-1-3p	0.5604583	0.010158757	0.803807324	1.96E-05
mir-103a-2-3p	mir-143-3p	-0.421679734	0.064049782	0.026005746	0.91333694
mir-143-3p	mir-103a-1-3p	-0.421679734	0.064049782	0.026005746	0.91333694
mir-144-3p	mir-1307-3p	-0.615485339	0.003867057	-0.831609088	5.51E-06
mir-340-5p	mir-223-3p	0.7412168	0.000184513	0.891338391	1.34E-07
mir-182-5p	mir-30d-5p	-0.031231696	0.896004558	-0.467138163	0.037835247
mir-103a-2-3p	mir-221-3p	0.605972093	0.0046263	0.826707815	7.00E-06
mir-103a-1-3p	mir-221-3p	0.605972093	0.0046263	0.826707815	7.00E-06
mir-98-5p	mir-30a-5p	-0.113064382	0.635066184	0.346095651	0.134974259
mir-181b-2-5p	mir-486-2-5p	0.497444433	0.025635405	0.071282197	0.765216811
mir-181b-1-5p	mir-486-2-5p	0.497444433	0.025635405	0.071282197	0.765216811
mir-423-3p	mir-486-2-5p	-0.417548817	0.066978669	-0.725337621	0.000295739
mir-30c-1-5p	mir-186-5p	-0.314400832	0.177002476	0.14746772	0.534969276
mir-186-5p	mir-30c-2-5p	-0.314400832	0.177002476	0.14746772	0.534969276
mir-103a-2-3p	mir-101-1-3p	0.30421044	0.192220913	-0.158468531	0.504582484
mir-103a-2-3p	mir-101-2-3p	0.30421044	0.192220913	-0.158468531	0.504582484
mir-101-1-3p	mir-103a-1-3p	0.30421044	0.192220913	-0.158468531	0.504582484
mir-103a-1-3p	mir-101-2-3p	0.30421044	0.192220913	-0.158468531	0.504582484
mir-486-2-5p	norepi	0.148181662	0.532971962	-0.313709292	0.178008452
mir-26a-2-5p	mir-125a-5p	0.828510451	6.42E-06	0.61038407	0.004260176
mir-125a-5p	mir-26a-1-5p	0.828510451	6.42E-06	0.61038407	0.004260176
mir-28-5p	mir-20b-5p	-0.042116736	0.86005714	-0.474381373	0.034576177
mir-151a-5p	mir-374b-5p	0.447197113	0.04804676	0.741883899	0.000180761
mir-98-5p	mir-24-2-3p	0.582214076	0.007072836	0.814083687	1.26E-05
mir-98-5p	mir-24-1-3p	0.582214076	0.007072836	0.814083687	1.26E-05
mir-451a	mir-148a-3p	0.29014444	0.214633973	-0.172649907	0.46667992
let-7i-5p	mir-3615	0.268538178	0.252294176	-0.194929293	0.410187789
mir-425-5p	mir-186-5p	0.870567627	6.00E-07	0.697646727	0.000626957
mir-361-5p	let-7d-5p	0.670871059	0.001204533	0.857728007	1.34E-06
mir-22-3p	mir-7-3-5p	0.790101269	3.41E-05	0.536633946	0.014709146
mir-22-3p	mir-7-1-5p	0.790101269	3.41E-05	0.536633946	0.014709146
mir-22-3p	mir-7-2-5p	0.790101269	3.41E-05	0.536633946	0.014709146
mir-629-5p	mir-30d-5p	-0.045243106	0.849778161	-0.47578454	0.033970953
mir-192-5p	mir-320a	0.871421014	5.67E-07	0.699766613	0.000593633
mir-144-3p	mir-25-3p	0.92404411	6.05E-09	0.815744213	1.17E-05
mir-182-5p	mir-186-5p	0.84685989	2.49E-06	0.648838938	0.001968211
mir-29a-3p	let-7f-2-5p	-0.109518243	0.645781352	0.346688559	0.134262315
mir-29a-3p	let-7f-1-5p	-0.109518243	0.645781352	0.346688559	0.134262315
mir-99b-5p	mir-10a-5p	0.459200696	0.041675411	0.747737897	0.000150544
mir-103a-2-3p	mir-191-5p	0.827569576	6.72E-06	0.929082519	3.32E-09
mir-103a-1-3p	mir-191-5p	0.827569576	6.72E-06	0.929082519	3.32E-09
mir-92a-2-3p	mir-192-5p	0.553795902	0.011296652	0.151689179	0.523209759
mir-192-5p	mir-92a-1-3p	0.553795902	0.011296652	0.151689179	0.523209759
mir-10b-5p	mir-107	-0.030670817	0.897862735	0.413271286	0.070116445
mir-361-5p	mir-744-5p	0.810614933	1.46E-05	0.921469529	8.09E-09
let-7f-2-5p	mir-148b-3p	0.252235854	0.283330204	0.621466508	0.003444887
mir-148b-3p	let-7f-1-5p	0.252235854	0.283330204	0.621466508	0.003444887
mir-451a	mir-20b-5p	0.909569088	2.76E-08	0.963615613	9.30E-12
mir-181a-2-5p	mir-106b-3p	0.025115179	0.916294564	-0.416969439	0.067397373
mir-106b-3p	mir-181a-1-5p	0.025115179	0.916294564	-0.416969439	0.067397373
mir-199a-2-3p	mir-486-2-5p	-0.285705154	0.222050375	-0.642840594	0.002235167
mir-486-2-5p	mir-199a-1-3p	-0.285705154	0.222050375	-0.642840594	0.002235167
mir-486-2-5p	mir-199b-3p	-0.285705154	0.222050375	-0.642840594	0.002235167
mir-106b-3p	mir-150-5p	0.0202374	0.932511762	0.453625861	0.044547764
mir-16-2-5p	mir-192-5p	0.895527765	9.58E-08	0.753152596	0.000126557

mir-192-5p	mir-16-1-5p	0.895527765	9.58E-08	0.753152596	0.000126557
mir-92a-2-3p	let-7i-5p	-0.0960414	0.687104488	-0.511721214	0.021089981
let-7i-5p	mir-92a-1-3p	-0.0960414	0.687104488	-0.511721214	0.021089981
mir-423-5p	mir-629-5p	0.647943029	0.002006298	0.29418586	0.208025779
mir-19b-2-3p	mir-629-5p	0.877711235	3.70E-07	0.715070528	0.000394653
mir-19b-1-3p	mir-629-5p	0.877711235	3.70E-07	0.715070528	0.000394653
mir-361-5p	mir-24-2-3p	0.75977854	0.00010173	0.898405509	7.53E-08
mir-361-5p	mir-24-1-3p	0.75977854	0.00010173	0.898405509	7.53E-08
mir-451a	mir-20a-5p	0.907482677	3.36E-08	0.779819231	5.03E-05
mir-374b-5p	mir-20b-5p	0.123901595	0.60275758	-0.330694056	0.154411793
mir-103a-2-3p	mir-146a-5p	0.623910771	0.003283748	0.833496641	5.02E-06
mir-146a-5p	mir-103a-1-3p	0.623910771	0.003283748	0.833496641	5.02E-06
mir-126-3p	mir-146b-5p	0.774926303	6.01E-05	0.905222265	4.14E-08
mir-126-3p	mir-221-3p	0.784047463	4.30E-05	0.909280427	2.83E-08
mir-223-3p	mir-30a-5p	-0.122910586	0.605683798	0.33085155	0.154203712
mir-423-3p	mir-20b-5p	-0.384304343	0.094335794	-0.702542092	0.000552284
mir-92a-2-3p	let-7a-3-5p	-0.657855743	0.001617414	-0.849897882	2.10E-06
mir-92a-2-3p	let-7a-2-5p	-0.657855743	0.001617414	-0.849897882	2.10E-06
mir-92a-2-3p	let-7a-1-5p	-0.657855743	0.001617414	-0.849897882	2.10E-06
let-7a-3-5p	mir-92a-1-3p	-0.657855743	0.001617414	-0.849897882	2.10E-06
let-7a-2-5p	mir-92a-1-3p	-0.657855743	0.001617414	-0.849897882	2.10E-06
let-7a-1-5p	mir-92a-1-3p	-0.657855743	0.001617414	-0.849897882	2.10E-06
mir-126-3p	mir-744-5p	0.641170772	0.002314643	0.841620212	3.30E-06
mir-223-3p	mir-20b-5p	-0.031331463	0.89567409	-0.460426524	0.041063474
mir-27b-3p	mir-151a-3p	0.622456519	0.003378847	0.832262618	5.34E-06
mir-126-3p	mir-21-5p	0.823690947	8.09E-06	0.926686091	4.44E-09
mir-26a-2-5p	mir-1307-3p	0.374184461	0.104091637	0.695899631	0.0006556
mir-1307-3p	mir-26a-1-5p	0.374184461	0.104091637	0.695899631	0.0006556
mir-101-1-3p	cpeptide	-0.200406306	0.39689512	0.25691111	0.274198322
mir-101-2-3p	cpeptide	-0.200406306	0.39689512	0.25691111	0.274198322
let-7d-5p	mir-27a-3p	-0.210737855	0.372478695	0.246642928	0.294498673
mir-584-5p	mir-10a-5p	-0.055545814	0.816077802	0.388535925	0.090461551
mir-451a	mir-425-5p	0.6919231	0.000724952	0.36794621	0.110458653
mir-143-3p	mir-125b-1-5p	0.356440946	0.122930047	-0.092106196	0.699340116
mir-143-3p	mir-125b-2-5p	0.356440946	0.122930047	-0.092106196	0.699340116
mir-182-5p	let-7d-3p	-0.448299396	0.047432315	-0.738438878	0.000200861
mir-126-3p	mir-223-3p	0.71983457	0.00034573	0.879019786	3.37E-07
mir-122-5p	mir-151a-5p	-0.43055943	0.058081859	0.003527447	0.988224114
mir-98-5p	mir-106b-3p	-0.222288693	0.346216177	-0.598081885	0.005345782
mir-98-5p	mir-3615	-0.326773193	0.159654978	-0.665844399	0.00135198
mir-122-5p	mir-30d-5p	-0.325893753	0.16084769	0.124962678	0.599630917
mir-143-3p	let-7d-5p	-0.164730159	0.487666734	0.288874678	0.216738392
mir-3615	mir-151a-3p	-0.147322551	0.535375821	-0.545481187	0.012859343
mir-199a-1-5p	mir-151a-3p	0.290338563	0.214313435	0.642247118	0.002263148
mir-151a-3p	mir-199a-2-5p	0.290338563	0.214313435	0.642247118	0.002263148
mir-584-5p	mir-28-3p	0.407012805	0.074904111	0.713840856	0.000408197
mir-26b-5p	mir-10b-5p	-0.143185899	0.547019866	0.308166227	0.18621247
mir-146b-5p	mir-148a-3p	-0.170361278	0.47269711	0.282712359	0.227143697
mir-26a-2-5p	mir-223-3p	0.772998418	6.44E-05	0.903358832	4.90E-08
mir-223-3p	mir-26a-1-5p	0.772998418	6.44E-05	0.903358832	4.90E-08
mir-10b-5p	mir-543	0.412925418	0.070374885	-0.023209486	0.922626994
mir-222-3p	mir-29a-3p	0.700062279	0.000589107	0.385037715	0.093655802
mir-140-5p	cpeptide	-0.24577315	0.296259401	0.207488677	0.380063949
mir-26a-2-5p	let-7a-3-5p	0.953595834	8.00E-11	0.88719043	1.85E-07
mir-26a-2-5p	let-7a-2-5p	0.953595834	8.00E-11	0.88719043	1.85E-07
mir-26a-2-5p	let-7a-1-5p	0.953595834	8.00E-11	0.88719043	1.85E-07
let-7a-3-5p	mir-26a-1-5p	0.953595834	8.00E-11	0.88719043	1.85E-07
let-7a-2-5p	mir-26a-1-5p	0.953595834	8.00E-11	0.88719043	1.85E-07
let-7a-1-5p	mir-26a-1-5p	0.953595834	8.00E-11	0.88719043	1.85E-07
mir-103a-2-3p	mir-23a-3p	0.559611518	0.010297989	0.798179541	2.47E-05
mir-103a-1-3p	mir-23a-3p	0.559611518	0.010297989	0.798179541	2.47E-05
mir-222-3p	mir-20a-5p	0.829843795	6.02E-06	0.928674262	3.50E-09
mir-143-3p	mir-223-3p	-0.086055393	0.7182928	0.358135129	0.12103327
mir-199a-1-5p	mir-126-5p	0.354607225	0.125006841	0.681296262	0.000941455

mir-126-5p	mir-199a-2-5p	0.354607225	0.125006841	0.681296262	0.000941455
mir-27b-3p	mir-374b-5p	0.535531119	0.014953996	0.784892272	4.16E-05
mir-15a-5p	mir-15b-5p	0.413931056	0.069625424	-0.019703223	0.934289413
mir-423-3p	mir-24-2-3p	0.36984178	0.108494995	0.690087573	0.000759001
mir-423-3p	mir-24-1-3p	0.36984178	0.108494995	0.690087573	0.000759001
mir-423-3p	mir-28-5p	0.409701599	0.072818234	0.71384601	0.00040814
mir-106a-5p	mir-128-1-3p	0.110957431	0.641424385	-0.33479083	0.149062313
mir-106a-5p	mir-128-2-3p	0.110957431	0.641424385	-0.33479083	0.149062313
mir-21-5p	mir-151a-3p	0.706813575	0.000493431	0.871724581	5.56E-07
mir-27b-3p	mir-28-5p	0.627540201	0.003056063	0.832587571	5.25E-06
mir-92a-2-3p	mir-1307-3p	-0.390497417	0.088705907	-0.70210309	0.000558657
mir-1307-3p	mir-92a-1-3p	-0.390497417	0.088705907	-0.70210309	0.000558657
mir-532-5p	mir-10b-5p	-0.170365586	0.472685747	0.279359324	0.232939778
mir-125a-5p	mir-148a-3p	-0.443292718	0.05027241	-0.017298516	0.94229547
mir-423-3p	let-7b-5p	-0.417135615	0.067277081	-0.717701728	0.000366952
mir-423-5p	mir-20b-5p	0.742701981	0.000176249	0.460409132	0.041072107
let-7a-3-5p	mir-224-5p	0.346822303	0.134102087	0.67526828	0.001086884
let-7a-2-5p	mir-224-5p	0.346822303	0.134102087	0.67526828	0.001086884
mir-224-5p	let-7a-1-5p	0.346822303	0.134102087	0.67526828	0.001086884
mir-126-3p	mir-629-5p	-0.411899337	0.071145794	-0.714324402	0.000402825
mir-532-5p	mir-192-5p	0.825647153	7.37E-06	0.614858726	0.003913662
mir-340-5p	mir-106b-3p	-0.277401715	0.236367621	-0.630595	0.002874746
mir-7-3-5p	mir-151a-3p	0.0218194	0.927248966	-0.409868055	0.072690545
mir-7-1-5p	mir-151a-3p	0.0218194	0.927248966	-0.409868055	0.072690545
mir-151a-3p	mir-7-2-5p	0.0218194	0.927248966	-0.409868055	0.072690545
mir-122-5p	mir-28-5p	0.052023513	0.827567416	0.46912741	0.03691736
mir-19b-2-3p	mir-98-5p	0.043789044	0.854556071	-0.390826522	0.088413814
mir-98-5p	mir-19b-1-3p	0.043789044	0.854556071	-0.390826522	0.088413814
mir-3615	mir-122-5p	0.287799024	0.218531687	-0.158772402	0.503755111
mir-10b-5p	mir-93-5p	-0.245848694	0.29610622	0.20235931	0.392213342
mir-26b-5p	mir-98-5p	0.012698507	0.957624634	-0.416286227	0.06789364
mir-144-3p	mir-221-3p	-0.417286218	0.067168201	-0.716367302	0.000380786
mir-144-3p	mir-423-5p	0.704406042	0.000525909	0.397244823	0.082857876
mir-584-5p	mir-375	-0.163515961	0.490924853	0.282372065	0.227727607
mir-584-5p	mir-125b-1-5p	-0.227786887	0.334104469	0.219531849	0.352383956
mir-584-5p	mir-125b-2-5p	-0.227786887	0.334104469	0.219531849	0.352383956
mir-199a-1-5p	mir-106b-3p	-0.566318397	0.009236546	-0.799454114	2.34E-05
mir-106b-3p	mir-199a-2-5p	-0.566318397	0.009236546	-0.799454114	2.34E-05
mir-146b-5p	mir-191-5p	0.662069628	0.00147243	0.848680062	2.25E-06
mir-1307-3p	mir-126-5p	-0.134997098	0.57040169	0.308415817	0.185837672
mir-7-3-5p	mir-28-3p	-0.252781062	0.282255707	-0.612530214	0.004090943
mir-7-1-5p	mir-28-3p	-0.252781062	0.282255707	-0.612530214	0.004090943
mir-7-2-5p	mir-28-3p	-0.252781062	0.282255707	-0.612530214	0.004090943
mir-122-5p	mir-93-5p	0.030820601	0.897366453	-0.399973285	0.080575982
let-7i-5p	mir-25-3p	0.468932354	0.03700659	0.054847013	0.818354471
mir-629-5p	let-7d-3p	-0.468959469	0.036994176	-0.745263008	0.000162743
mir-181a-2-5p	mir-27a-3p	0.103951616	0.662737368	0.505975224	0.022835212
mir-27a-3p	mir-181a-1-5p	0.103951616	0.662737368	0.505975224	0.022835212
let-7i-5p	mir-451a	0.409403124	0.073047614	-0.017968124	0.940065557
mir-22-3p	mir-92a-2-3p	0.507484171	0.022366196	0.106354433	0.655398376
mir-22-3p	mir-92a-1-3p	0.507484171	0.022366196	0.106354433	0.655398376
mir-584-5p	mir-27a-3p	-0.381056064	0.097391148	0.05068813	0.831932346
mir-425-5p	let-7b-5p	0.707451164	0.000485121	0.405494231	0.076101685
mir-23a-3p	mir-28-5p	0.715576728	0.00038919	0.874038428	4.76E-07
mir-629-5p	mir-23a-3p	-0.449754196	0.046630623	-0.733153697	0.000235406
let-7i-5p	mir-106a-5p	0.410372933	0.072304275	-0.015023574	0.949874415
mir-224-5p	mir-191-5p	0.430663409	0.058014572	0.721813823	0.00032698
mir-29a-3p	mir-151a-5p	-0.103209887	0.66500888	0.333782915	0.150366268
mir-103a-2-3p	mir-122-5p	-0.067349468	0.777849739	0.365225279	0.113321946
mir-122-5p	mir-103a-1-3p	-0.067349468	0.777849739	0.365225279	0.113321946
mir-24-2-3p	cpeptide	0.107708241	0.651276708	-0.329441106	0.156074129
mir-24-1-3p	cpeptide	0.107708241	0.651276708	-0.329441106	0.156074129
mir-532-5p	mir-148b-3p	0.290055783	0.214780468	-0.150452815	0.526641239
mir-126-3p	mir-584-5p	0.309217435	0.184637367	0.646815348	0.002055113

mir-29a-3p	let-7a-3-5p	-0.001021902	0.996588419	0.420929848	0.064574155
mir-29a-3p	let-7a-2-5p	-0.001021902	0.996588419	0.420929848	0.064574155
mir-29a-3p	let-7a-1-5p	-0.001021902	0.996588419	0.420929848	0.064574155
mir-744-5p	mir-194-2-5p	-0.698397945	0.000614971	-0.392405585	0.087022177
mir-744-5p	mir-194-1-5p	-0.698397945	0.000614971	-0.392405585	0.087022177
mir-17-5p	mir-20b-5p	0.828770012	6.34E-06	0.625933732	0.003155166
let-7f-2-5p	mir-28-3p	0.529131424	0.016440307	0.777172769	5.54E-05
let-7f-1-5p	mir-28-3p	0.529131424	0.016440307	0.777172769	5.54E-05
mir-29a-3p	mir-7-3-5p	0.464563124	0.039049635	0.054012071	0.821076516
mir-29a-3p	mir-7-1-5p	0.464563124	0.039049635	0.054012071	0.821076516
mir-29a-3p	mir-7-2-5p	0.464563124	0.039049635	0.054012071	0.821076516
mir-92a-2-3p	mir-744-5p	-0.733672342	0.000231806	-0.882182082	2.69E-07
mir-744-5p	mir-92a-1-3p	-0.733672342	0.000231806	-0.882182082	2.69E-07
mir-26b-5p	mir-186-5p	0.965454357	5.87E-12	0.917307029	1.27E-08
mir-122-5p	mir-107	0.044142489	0.853394231	-0.383921572	0.094692136
mir-106a-5p	mir-191-5p	-0.158493486	0.504514513	-0.543173709	0.013322612
mir-224-5p	mir-15b-5p	-0.016865726	0.943736956	0.406899603	0.074992897
mir-26b-5p	cpeptide	-0.231122241	0.326880522	0.210071799	0.374026581
mir-425-5p	mir-30c-1-5p	-0.035257877	0.8826818	0.391062726	0.088204611
mir-425-5p	mir-30c-2-5p	-0.035257877	0.8826818	0.391062726	0.088204611
mir-26b-5p	mir-629-5p	0.916083422	1.44E-08	0.806384985	1.76E-05
mir-320a	mir-363-3p	0.896070275	9.16E-08	0.763441855	8.99E-05
mir-98-5p	mir-363-3p	-0.198066851	0.402543634	-0.570313437	0.008647832
mir-181a-2-5p	mir-20b-5p	0.14955371	0.529143244	-0.288170293	0.217911622
mir-20b-5p	mir-181a-1-5p	0.14955371	0.529143244	-0.288170293	0.217911622
mir-103a-2-3p	let-7f-2-5p	0.656311171	0.001673471	0.843608395	2.97E-06
mir-103a-2-3p	let-7f-1-5p	0.656311171	0.001673471	0.843608395	2.97E-06
let-7f-2-5p	mir-103a-1-3p	0.656311171	0.001673471	0.843608395	2.97E-06
mir-103a-1-3p	let-7f-1-5p	0.656311171	0.001673471	0.843608395	2.97E-06
mir-29a-3p	mir-25-3p	0.52672657	0.017028515	0.137835114	0.562249093
mir-21-5p	mir-20b-5p	-0.325889938	0.160852876	-0.655451686	0.001705363
mir-629-5p	mir-28-5p	-0.274137894	0.242154789	-0.621737212	0.003426724
mir-16-2-5p	mir-20a-5p	0.950425138	1.43E-10	0.883119194	2.51E-07
mir-20a-5p	mir-16-1-5p	0.950425138	1.43E-10	0.883119194	2.51E-07
let-7b-5p	mir-484	0.733101106	0.000235774	0.453530472	0.044598205
mir-151a-3p	mir-125b-1-5p	-0.053872651	0.821531239	0.373350693	0.104926823
mir-151a-3p	mir-125b-2-5p	-0.053872651	0.821531239	0.373350693	0.104926823
mir-10b-5p	mir-1307-3p	0.117919086	0.620509893	-0.316342927	0.174198043
mir-22-3p	mir-16-2-5p	0.844757356	2.79E-06	0.659292983	0.001566676
mir-22-3p	mir-16-1-5p	0.844757356	2.79E-06	0.659292983	0.001566676
let-7a-3-5p	mir-30a-5p	0.024471549	0.918432761	0.438523074	0.053097352
let-7a-2-5p	mir-30a-5p	0.024471549	0.918432761	0.438523074	0.053097352
let-7a-1-5p	mir-30a-5p	0.024471549	0.918432761	0.438523074	0.053097352
let-7i-5p	mir-143-3p	-0.438873199	0.052885955	-0.02503604	0.916557442
let-7i-5p	mir-363-3p	0.406696447	0.07515243	-0.013933576	0.953507241
mir-92a-2-3p	mir-425-5p	0.372703969	0.105577985	-0.053655966	0.822238077
mir-425-5p	mir-92a-1-3p	0.372703969	0.105577985	-0.053655966	0.822238077
mir-98-5p	mir-101-1-3p	-0.043877116	0.854266533	-0.453556187	0.044584603
mir-98-5p	mir-101-2-3p	-0.043877116	0.854266533	-0.453556187	0.044584603
mir-423-3p	mir-15a-5p	-0.516172534	0.019812299	-0.7683743	7.58E-05
mir-378a-3p	mir-486-2-5p	0.837975282	4.00E-06	0.646423239	0.002072318
mir-19b-2-3p	mir-29a-3p	0.60923959	0.004352763	0.257057983	0.273914458
mir-29a-3p	mir-19b-1-3p	0.60923959	0.004352763	0.257057983	0.273914458
mir-139-5p	mir-20b-5p	-0.000563273	0.998119532	-0.418042682	0.066623309
mir-144-3p	mir-191-5p	-0.080103274	0.737092291	-0.481484288	0.031597027
mir-146a-5p	mir-199a-2-3p	0.958801356	2.80E-11	0.982856996	1.14E-14
mir-146a-5p	mir-199a-1-3p	0.958801356	2.80E-11	0.982856996	1.14E-14
mir-146a-5p	mir-199b-3p	0.958801356	2.80E-11	0.982856996	1.14E-14
mir-17-5p	mir-486-2-5p	0.810512406	1.47E-05	0.594183146	0.005733915
mir-125a-5p	mir-378a-3p	-0.565242723	0.009400512	-0.193624776	0.413389069
mir-629-5p	mir-93-5p	0.883214412	2.49E-07	0.737881763	0.000204285
mir-15a-5p	mir-484	0.585134928	0.006724601	0.222164223	0.346493285
mir-106a-5p	mir-425-5p	0.679713551	0.000977947	0.366469273	0.112006314
mir-146a-5p	mir-363-3p	-0.397830921	0.082363719	-0.698766872	0.000609157

mir-451a	mir-320a	0.904936006	4.25E-08	0.783798409	4.34E-05
mir-140-5p	mir-106b-3p	0.416814575	0.067509623	0.000285582	0.999046592
mir-125b-1-5p	mir-128-1-3p	-0.073554109	0.757944562	0.353821537	0.125904278
mir-125b-1-5p	mir-128-2-3p	-0.073554109	0.757944562	0.353821537	0.125904278
mir-128-1-3p	mir-125b-2-5p	-0.073554109	0.757944562	0.353821537	0.125904278
mir-128-2-3p	mir-125b-2-5p	-0.073554109	0.757944562	0.353821537	0.125904278
mir-26a-2-5p	mir-423-3p	0.752774154	0.00012812	0.890173408	1.47E-07
mir-423-3p	mir-26a-1-5p	0.752774154	0.00012812	0.890173408	1.47E-07
mir-103a-2-3p	mir-185-5p	0.460416777	0.041068312	0.054443459	0.819669873
mir-103a-1-3p	mir-185-5p	0.460416777	0.041068312	0.054443459	0.819669873
mir-26a-2-5p	mir-27a-3p	-0.115307378	0.628324369	0.316224911	0.174367592
mir-27a-3p	mir-26a-1-5p	-0.115307378	0.628324369	0.316224911	0.174367592
mir-340-5p	mir-28-3p	0.458201491	0.042179412	0.734418182	0.000226711
mir-16-2-5p	mir-98-5p	-0.058124424	0.80768919	-0.463214736	0.039697475
mir-98-5p	mir-16-1-5p	-0.058124424	0.80768919	-0.463214736	0.039697475
mir-21-5p	mir-629-5p	-0.45205555	0.045383733	-0.730820939	0.000252197
mir-10b-5p	mir-25-3p	-0.067674973	0.776802048	0.358395875	0.120743212
mir-224-5p	let-7d-5p	0.287298359	0.21936969	0.628099357	0.003022181
mir-99a-5p	mir-363-3p	0.291148519	0.212979439	-0.141656261	0.551354358
mir-21-5p	mir-543	0.774324775	6.14E-05	0.528934147	0.016487938
mir-423-5p	mir-125b-1-5p	-0.042445501	0.858975168	0.379217396	0.099152298
mir-423-5p	mir-125b-2-5p	-0.042445501	0.858975168	0.379217396	0.099152298
mir-484	norepi	0.371838967	0.106453494	-0.050160338	0.833658841
mir-451a	mir-126-5p	0.180305087	0.446838807	-0.252673788	0.282466923
mir-361-5p	mir-148b-3p	0.118540672	0.618655762	0.507667544	0.022309724
mir-106a-5p	mir-126-5p	0.188843955	0.425236004	-0.24391048	0.300051678
mir-17-5p	mir-26b-5p	0.881398765	2.85E-07	0.736249232	0.000214607
mir-361-5p	mir-15a-5p	-0.689797448	0.000764504	-0.858409158	1.28E-06
mir-425-5p	mir-320a	0.820357403	9.45E-06	0.615936946	0.003833754
mir-194-2-5p	mir-150-5p	0.346780508	0.134152144	0.664731725	0.00138659
mir-194-1-5p	mir-150-5p	0.346780508	0.134152144	0.664731725	0.00138659
mir-374b-5p	mir-99a-5p	-0.092647235	0.697653561	0.333157307	0.15117961
let-7i-5p	mir-151a-5p	0.164311367	0.488789295	0.540461763	0.013884195
mir-199a-1-5p	mir-20b-5p	-0.350950341	0.129222829	-0.66677192	0.001323687
mir-199a-2-5p	mir-20b-5p	-0.350950341	0.129222829	-0.66677192	0.001323687
mir-20a-5p	norepi	0.191925286	0.417579808	-0.238778461	0.310652274
mir-93-5p	norepi	0.186845066	0.430242408	-0.243651619	0.300581034
mir-221-3p	cpeptide	-0.069788784	0.770007499	-0.467809396	0.037523564
mir-192-5p	mir-93-5p	0.83424834	4.83E-06	0.643847219	0.002188367
mir-194-2-5p	mir-30c-1-5p	-0.448019436	0.047587799	-0.045004426	0.850562099
mir-194-2-5p	mir-30c-2-5p	-0.448019436	0.047587799	-0.045004426	0.850562099
mir-194-1-5p	mir-30c-1-5p	-0.448019436	0.047587799	-0.045004426	0.850562099
mir-194-1-5p	mir-30c-2-5p	-0.448019436	0.047587799	-0.045004426	0.850562099
mir-126-3p	mir-543	0.804046171	1.94E-05	0.586841869	0.006527688
mir-98-5p	mir-7-3-5p	-0.088999427	0.70905075	-0.482464026	0.031202393
mir-98-5p	mir-7-1-5p	-0.088999427	0.70905075	-0.482464026	0.031202393
mir-98-5p	mir-7-2-5p	-0.088999427	0.70905075	-0.482464026	0.031202393
mir-10b-5p	mir-629-5p	-0.184020587	0.437369552	0.245562709	0.29668637
mir-10b-5p	mir-24-2-3p	0.358026773	0.121153954	-0.061619085	0.796352855
mir-10b-5p	mir-24-1-3p	0.358026773	0.121153954	-0.061619085	0.796352855
mir-320a	mir-30e-5p	0.867072781	7.52E-07	0.708905497	0.00046661
mir-26b-5p	mir-486-2-5p	0.951892262	1.10E-10	0.888633191	1.66E-07
mir-192-5p	cpeptide	-0.038232684	0.872857323	0.377459288	0.100857885
mir-139-5p	mir-30a-5p	-0.115586984	0.627485921	0.308776762	0.185296559
mir-25-3p	mir-484	0.78999865	3.42E-05	0.562266413	0.009866572
mir-103a-2-3p	mir-199a-2-3p	0.662110748	0.001471071	0.843067869	3.06E-06
mir-103a-2-3p	mir-199a-1-3p	0.662110748	0.001471071	0.843067869	3.06E-06
mir-103a-2-3p	mir-199b-3p	0.662110748	0.001471071	0.843067869	3.06E-06
mir-199a-2-3p	mir-103a-1-3p	0.662110748	0.001471071	0.843067869	3.06E-06
mir-103a-1-3p	mir-199a-1-3p	0.662110748	0.001471071	0.843067869	3.06E-06
mir-103a-1-3p	mir-199b-3p	0.662110748	0.001471071	0.843067869	3.06E-06
mir-199a-2-3p	let-7b-5p	-0.403895207	0.077378058	-0.697789001	0.000624672
let-7b-5p	mir-199a-1-3p	-0.403895207	0.077378058	-0.697789001	0.000624672
let-7b-5p	mir-199b-3p	-0.403895207	0.077378058	-0.697789001	0.000624672

mir-101-1-3p	mir-20a-5p	0.965412861	5.93E-12	0.919546884	9.99E-09
mir-20a-5p	mir-101-2-3p	0.965412861	5.93E-12	0.919546884	9.99E-09
mir-143-3p	mir-150-5p	0.088352033	0.711079776	-0.33221232	0.152413969
mir-125b-1-5p	mir-28-5p	0.068715123	0.773456648	0.464139368	0.039252341
mir-28-5p	mir-125b-2-5p	0.068715123	0.773456648	0.464139368	0.039252341
mir-744-5p	mir-28-5p	0.515875372	0.019895625	0.763413936	9.00E-05
mir-146b-5p	mir-223-3p	0.524190211	0.017667023	0.768123466	7.65E-05
mir-144-3p	mir-148b-3p	0.447955877	0.047623152	0.048517228	0.839038387
let-7f-2-5p	mir-143-3p	-0.044183938	0.853257996	0.370773464	0.107539148
mir-143-3p	let-7f-1-5p	-0.044183938	0.853257996	0.370773464	0.107539148
mir-3615	norepi	-0.065291416	0.784482393	-0.460976049	0.040791416
let-7f-2-5p	mir-224-5p	0.307751141	0.186836914	0.635871665	0.002582651
mir-224-5p	let-7f-1-5p	0.307751141	0.186836914	0.635871665	0.002582651
let-7f-2-5p	mir-181a-2-5p	0.605756249	0.004644857	0.812735325	1.33E-05
let-7f-2-5p	mir-181a-1-5p	0.605756249	0.004644857	0.812735325	1.33E-05
mir-181a-2-5p	let-7f-1-5p	0.605756249	0.004644857	0.812735325	1.33E-05
let-7f-1-5p	mir-181a-1-5p	0.605756249	0.004644857	0.812735325	1.33E-05
mir-16-2-5p	mir-425-5p	0.781692372	4.69E-05	0.548989922	0.012179899
mir-425-5p	mir-16-1-5p	0.781692372	4.69E-05	0.548989922	0.012179899
mir-29a-3p	let-7d-5p	-0.193099107	0.414682867	0.232569972	0.323774011
mir-223-3p	mir-106b-3p	-0.310979712	0.182017071	-0.63746083	0.002499694
mir-181b-2-5p	mir-106b-3p	0.460626748	0.040964186	0.065805741	0.782823475
mir-181b-1-5p	mir-106b-3p	0.460626748	0.040964186	0.065805741	0.782823475
mir-30c-1-5p	let-7g-5p	0.145223744	0.541269352	0.52149214	0.018367113
let-7g-5p	mir-30c-2-5p	0.145223744	0.541269352	0.52149214	0.018367113
mir-532-5p	mir-30c-1-5p	-0.621609908	0.003435255	-0.287528486	0.218984247
mir-532-5p	mir-30c-2-5p	-0.621609908	0.003435255	-0.287528486	0.218984247
mir-223-3p	mir-486-2-5p	-0.176321512	0.457108425	-0.543876591	0.013180092
mir-27b-3p	mir-10a-5p	0.697562378	0.000628315	0.406273115	0.075485675
mir-143-3p	mir-375	0.191085313	0.419659497	-0.233459757	0.321873482
mir-29a-3p	mir-26a-2-5p	-0.07860404	0.74185078	0.338613089	0.144189073
mir-29a-3p	mir-26a-1-5p	-0.07860404	0.74185078	0.338613089	0.144189073
mir-27b-3p	mir-126-5p	0.427795357	0.059892371	0.710627094	0.000445476
mir-451a	mir-185-5p	0.906810074	3.57E-08	0.792515584	3.10E-05
mir-15b-5p	norepi	0.458541847	0.04200721	0.064321789	0.787612285
mir-27b-3p	mir-15a-5p	-0.528721041	0.016539514	-0.769512812	7.29E-05
mir-144-3p	cpeptide	-0.102433738	0.667388812	0.316607888	0.173817798
mir-27b-3p	norepi	-0.124908806	0.599789499	0.295740092	0.205520719
mir-92a-2-3p	mir-423-3p	-0.376967662	0.10133862	-0.678569955	0.001005051
mir-423-3p	mir-92a-1-3p	-0.376967662	0.10133862	-0.678569955	0.001005051
mir-223-3p	mir-128-1-3p	0.568918283	0.008849842	0.791609819	3.21E-05
mir-223-3p	mir-128-2-3p	0.568918283	0.008849842	0.791609819	3.21E-05
mir-7-3-5p	mir-199a-2-3p	-0.246057723	0.295682622	-0.591839524	0.00597818
mir-7-3-5p	mir-199a-1-3p	-0.246057723	0.295682622	-0.591839524	0.00597818
mir-7-3-5p	mir-199b-3p	-0.246057723	0.295682622	-0.591839524	0.00597818
mir-199a-2-3p	mir-7-1-5p	-0.246057723	0.295682622	-0.591839524	0.00597818
mir-199a-2-3p	mir-7-2-5p	-0.246057723	0.295682622	-0.591839524	0.00597818
mir-7-1-5p	mir-199a-1-3p	-0.246057723	0.295682622	-0.591839524	0.00597818
mir-7-1-5p	mir-199b-3p	-0.246057723	0.295682622	-0.591839524	0.00597818
mir-199a-1-3p	mir-7-2-5p	-0.246057723	0.295682622	-0.591839524	0.00597818
mir-7-2-5p	mir-199b-3p	-0.246057723	0.295682622	-0.591839524	0.00597818
mir-185-5p	norepi	0.213480712	0.36614274	-0.209061951	0.37638035
mir-99b-5p	mir-30a-5p	0.088209288	0.711527413	0.475400907	0.034135597
let-7f-2-5p	mir-30a-5p	-0.001720001	0.994257871	0.402352612	0.078624415
mir-30a-5p	let-7f-1-5p	-0.001720001	0.994257871	0.402352612	0.078624415
mir-744-5p	mir-224-5p	0.336885646	0.146377486	0.651936008	0.001841175
mir-30c-1-5p	mir-23a-3p	0.587527668	0.006449919	0.80099433	2.20E-05
mir-23a-3p	mir-30c-2-5p	0.587527668	0.006449919	0.80099433	2.20E-05
mir-144-3p	mir-744-5p	-0.517235482	0.019516516	-0.761591804	9.57E-05
mir-361-5p	mir-28-5p	0.624286354	0.003259552	0.820758191	9.27E-06
mir-224-5p	mir-15a-5p	-0.19521191	0.409496028	-0.554312213	0.011204936
mir-148a-3p	mir-128-1-3p	0.066172612	0.781640709	0.456676664	0.042957595
mir-148a-3p	mir-128-2-3p	0.066172612	0.781640709	0.456676664	0.042957595
mir-103a-2-3p	mir-181a-2-5p	0.764631953	8.63E-05	0.892372611	1.24E-07

mir-103a-2-3p	mir-181a-1-5p	0.764631953	8.63E-05	0.892372611	1.24E-07
mir-181a-2-5p	mir-103a-1-3p	0.764631953	8.63E-05	0.892372611	1.24E-07
mir-103a-1-3p	mir-181a-1-5p	0.764631953	8.63E-05	0.892372611	1.24E-07
mir-26b-5p	mir-151a-3p	0.011509052	0.961590938	-0.392301197	0.087113674
mir-27a-3p	mir-30e-5p	0.437804988	0.053532933	0.714036702	0.000406014
mir-199a-1-5p	mir-122-5p	-0.202360681	0.392210068	0.216740219	0.358693828
mir-122-5p	mir-199a-2-5p	-0.202360681	0.392210068	0.216740219	0.358693828
mir-106a-5p	mir-532-5p	0.880741577	2.98E-07	0.947268064	2.47E-10
mir-222-3p	mir-186-5p	0.769707539	7.24E-05	0.894686215	1.03E-07
mir-106a-5p	mir-486-2-5p	0.957111409	3.99E-11	0.902402823	5.33E-08
mir-126-3p	mir-150-5p	0.34845715	0.132154414	-0.061238161	0.797586674
mir-30a-5p	let-7d-3p	0.054810785	0.818472538	0.446094363	0.048667557
mir-16-2-5p	mir-148a-3p	0.2928952	0.210121327	-0.122409763	0.607164819
mir-148a-3p	mir-16-1-5p	0.2928952	0.210121327	-0.122409763	0.607164819
mir-744-5p	mir-30c-1-5p	0.78340962	4.40E-05	0.55768233	0.010620989
mir-744-5p	mir-30c-2-5p	0.78340962	4.40E-05	0.55768233	0.010620989
mir-25-3p	mir-199a-2-3p	-0.221143802	0.348769925	-0.5709794	0.00855274
mir-25-3p	mir-199a-1-3p	-0.221143802	0.348769925	-0.5709794	0.00855274
mir-25-3p	mir-199b-3p	-0.221143802	0.348769925	-0.5709794	0.00855274
mir-182-5p	mir-125b-1-5p	0.207761873	0.37942285	-0.210047088	0.374084078
mir-182-5p	mir-125b-2-5p	0.207761873	0.37942285	-0.210047088	0.374084078
mir-29a-3p	mir-744-5p	-0.3169117	0.173382494	0.095348839	0.689252525
mir-182-5p	mir-10b-5p	-0.187077281	0.429659205	0.23015703	0.328961442
mir-584-5p	mir-143-3p	-0.100948685	0.671951002	0.311643051	0.181037326
let-7i-5p	mir-224-5p	0.097817909	0.681605064	0.479017461	0.032607969
mir-146b-5p	mir-194-2-5p	-0.630071049	0.002905192	-0.308053779	0.186381495
mir-146b-5p	mir-194-1-5p	-0.630071049	0.002905192	-0.308053779	0.186381495
mir-148a-3p	mir-20b-5p	0.167345964	0.480684035	-0.2488276	0.290104482
mir-10b-5p	mir-20b-5p	-0.060790683	0.79903665	0.346883069	0.134029333
mir-16-2-5p	mir-151a-3p	0.005341544	0.98216877	-0.394496643	0.085204251
mir-151a-3p	mir-16-1-5p	0.005341544	0.98216877	-0.394496643	0.085204251
mir-182-5p	mir-223-3p	-0.270441347	0.248818262	-0.60421845	0.004778861
mir-451a	mir-194-2-5p	0.851771126	1.89E-06	0.685890213	0.000841977
mir-451a	mir-194-1-5p	0.851771126	1.89E-06	0.685890213	0.000841977
mir-92a-2-3p	mir-30e-5p	0.41629828	0.067884861	0.020902287	0.930299559
mir-30e-5p	mir-92a-1-3p	0.41629828	0.067884861	0.020902287	0.930299559
mir-140-5p	mir-629-5p	0.411519402	0.071432842	0.015239488	0.949154913
mir-181b-2-5p	mir-194-2-5p	0.581864893	0.007115437	0.238473364	0.311289497
mir-181b-2-5p	mir-194-1-5p	0.581864893	0.007115437	0.238473364	0.311289497
mir-181b-1-5p	mir-194-2-5p	0.581864893	0.007115437	0.238473364	0.311289497
mir-181b-1-5p	mir-194-1-5p	0.581864893	0.007115437	0.238473364	0.311289497
mir-22-3p	let-7g-5p	0.472429446	0.03543204	0.733045468	0.000236163
mir-221-3p	mir-28-3p	0.652443064	0.001821046	0.834102222	4.87E-06
mir-101-1-3p	mir-151a-3p	0.093779157	0.694129493	-0.316568281	0.173874602
mir-151a-3p	mir-101-2-3p	0.093779157	0.694129493	-0.316568281	0.173874602
mir-30e-5p	norepi	0.316132193	0.174500874	-0.094007491	0.69341934
let-7i-5p	mir-320a	0.578315538	0.007560512	0.234012593	0.320696014
mir-192-5p	mir-20a-5p	0.877311135	3.80E-07	0.736360759	0.000213888
mir-98-5p	mir-143-3p	-0.076445823	0.748716674	0.331712629	0.153069498
mir-23a-3p	cpeptide	-0.030435337	0.898643024	-0.423336986	0.062902309
mir-423-3p	mir-199a-1-5p	0.69787083	0.000623361	0.857579716	1.35E-06
mir-423-3p	mir-199a-2-5p	0.69787083	0.000623361	0.857579716	1.35E-06
mir-1307-3p	mir-151a-5p	0.038854308	0.870806584	0.429784877	0.058584961
mir-27b-3p	mir-363-3p	-0.248891459	0.289976648	-0.588231794	0.006370863
mir-10b-5p	mir-122-5p	0.454508397	0.044083174	0.070149851	0.768848498
mir-181a-2-5p	mir-374b-5p	0.71301017	0.000417569	0.865115444	8.51E-07
mir-374b-5p	mir-181a-1-5p	0.71301017	0.000417569	0.865115444	8.51E-07
mir-10b-5p	mir-28-5p	0.373404968	0.104872309	-0.027638961	0.907915811
mir-26b-5p	mir-20a-5p	0.949061552	1.82E-10	0.885836643	2.05E-07
mir-340-5p	mir-99b-5p	0.753105141	0.000126752	0.508343099	0.022102655
mir-146a-5p	mir-106b-3p	-0.483146996	0.030929586	-0.738239693	0.00020208
mir-98-5p	let-7b-5p	-0.230061556	0.329167701	-0.574176243	0.008108059
mir-24-2-3p	mir-15b-5p	0.10736793	0.652311874	0.483195713	0.030910199
mir-24-1-3p	mir-15b-5p	0.10736793	0.652311874	0.483195713	0.030910199

mir-29a-3p	mir-21-5p	-0.026600324	0.911362918	0.373712605	0.104563701
mir-126-3p	mir-148b-3p	0.335991737	0.147519002	0.646169039	0.002083535
mir-340-5p	mir-20b-5p	-0.157062264	0.508419946	-0.520759058	0.01856111
mir-10b-5p	norepi	-0.282267433	0.227907341	0.128050906	0.590569388
mir-199a-1-5p	mir-30c-1-5p	0.830783706	5.74E-06	0.647963555	0.002005419
mir-199a-1-5p	mir-30c-2-5p	0.830783706	5.74E-06	0.647963555	0.002005419
mir-30c-1-5p	mir-199a-2-5p	0.830783706	5.74E-06	0.647963555	0.002005419
mir-199a-2-5p	mir-30c-2-5p	0.830783706	5.74E-06	0.647963555	0.002005419
mir-144-3p	let-7a-3-5p	-0.214668598	0.363417934	-0.56265178	0.00980519
mir-144-3p	let-7a-2-5p	-0.214668598	0.363417934	-0.56265178	0.00980519
mir-144-3p	let-7a-1-5p	-0.214668598	0.363417934	-0.56265178	0.00980519
mir-16-2-5p	mir-30e-5p	0.862948113	9.75E-07	0.709543083	0.000458686
mir-16-1-5p	mir-30e-5p	0.862948113	9.75E-07	0.709543083	0.000458686
mir-181b-2-5p	mir-107	0.515089262	0.020117402	0.150186504	0.527381744
mir-181b-1-5p	mir-107	0.515089262	0.020117402	0.150186504	0.527381744
mir-29a-3p	mir-30d-5p	-0.005828105	0.980544806	0.390316615	0.088866677
mir-423-5p	mir-148b-3p	0.508758053	0.021976223	0.142800778	0.548109719
mir-10b-5p	mir-148a-3p	-0.000247143	0.999174919	-0.394766636	0.084971584
let-7b-5p	mir-186-5p	0.90292981	5.09E-08	0.789870118	3.44E-05
mir-146b-5p	mir-374b-5p	0.427438647	0.0601291	0.703159026	0.000543433
mir-25-3p	mir-15b-5p	0.61993828	0.003548929	0.298935478	0.200433631
mir-23a-3p	mir-221-3p	0.814056831	1.26E-05	0.91468063	1.66E-08
mir-21-5p	mir-143-3p	0.052596875	0.825694767	0.437453327	0.053747236
mir-21-5p	mir-423-3p	0.68083937	0.000951869	0.847449905	2.41E-06
mir-7-3-5p	mir-30e-5p	0.812831164	1.33E-05	0.616637623	0.003782555
mir-7-1-5p	mir-30e-5p	0.812831164	1.33E-05	0.616637623	0.003782555
mir-7-2-5p	mir-30e-5p	0.812831164	1.33E-05	0.616637623	0.003782555
mir-25-3p	mir-629-5p	0.908329614	3.10E-08	0.801327244	2.17E-05
mir-629-5p	mir-150-5p	-0.081553469	0.732498193	0.321871392	0.16638125
mir-122-5p	mir-186-5p	-0.047474101	0.84245717	-0.432352412	0.056929832
mir-451a	mir-27a-3p	0.683900949	0.000883903	0.397920606	0.082288296
mir-25-3p	mir-194-2-5p	0.847548938	2.40E-06	0.681770301	0.000930751
mir-25-3p	mir-194-1-5p	0.847548938	2.40E-06	0.681770301	0.000930751
mir-146a-5p	mir-451a	-0.404332405	0.077027507	-0.68780872	0.000803147
mir-103a-2-3p	mir-125a-5p	0.352702679	0.127190171	0.654649779	0.001735577
mir-125a-5p	mir-103a-1-3p	0.352702679	0.127190171	0.654649779	0.001735577
mir-1307-3p	mir-15a-5p	-0.685779306	0.000844269	-0.849612388	2.14E-06
mir-30c-1-5p	mir-15b-5p	0.156910714	0.508834334	0.517447069	0.019458061
mir-30c-2-5p	mir-15b-5p	0.156910714	0.508834334	0.517447069	0.019458061
mir-106b-3p	let-7g-5p	0.505081989	0.023116503	0.140554944	0.554484646
mir-103a-2-3p	mir-532-5p	-0.046877309	0.844414344	-0.431325431	0.057587544
mir-532-5p	mir-103a-1-3p	-0.046877309	0.844414344	-0.431325431	0.057587544
mir-26b-5p	mir-192-5p	0.842277693	3.19E-06	0.672058543	0.001171753
mir-181a-2-5p	mir-10a-5p	0.108610968	0.648533747	0.480405563	0.032036041
mir-10a-5p	mir-181a-1-5p	0.108610968	0.648533747	0.480405563	0.032036041
mir-30c-1-5p	mir-20a-5p	-0.298123408	0.201718416	0.10646848	0.655050789
mir-20a-5p	mir-30c-2-5p	-0.298123408	0.201718416	0.10646848	0.655050789
mir-139-5p	mir-27a-3p	0.042110943	0.860076208	0.427080444	0.060367531
mir-378a-3p	mir-106b-3p	0.766952224	7.97E-05	0.536565306	0.014724291
mir-26b-5p	mir-363-3p	0.897293824	8.28E-08	0.953726068	7.81E-11
mir-26b-5p	mir-15b-5p	0.684110247	0.00087941	0.400057214	0.080506536
mir-10b-5p	mir-223-3p	0.23968809	0.308757104	-0.166739765	0.482297793
mir-103a-2-3p	mir-148a-3p	-0.009146841	0.969470354	0.382922834	0.095626553
mir-103a-1-3p	mir-148a-3p	-0.009146841	0.969470354	0.382922834	0.095626553
mir-320a	mir-15a-5p	0.781949686	4.65E-05	0.563382773	0.009689609
mir-22-3p	mir-21-5p	-0.319690237	0.169435895	0.081015005	0.73420299
mir-25-3p	mir-150-5p	0.099341752	0.676900078	0.471532572	0.035830784
mir-181b-2-5p	mir-7-3-5p	0.598886224	0.005268465	0.272079429	0.24585112
mir-181b-2-5p	mir-7-1-5p	0.598886224	0.005268465	0.272079429	0.24585112
mir-181b-2-5p	mir-7-2-5p	0.598886224	0.005268465	0.272079429	0.24585112
mir-7-3-5p	mir-181b-1-5p	0.598886224	0.005268465	0.272079429	0.24585112
mir-181b-1-5p	mir-7-1-5p	0.598886224	0.005268465	0.272079429	0.24585112
mir-181b-1-5p	mir-7-2-5p	0.598886224	0.005268465	0.272079429	0.24585112
mir-146a-5p	let-7d-5p	0.843820778	2.94E-06	0.928375304	3.63E-09

mir-181b-2-5p	let-7b-5p	0.519377662	0.018931116	0.16316744	0.491862025
mir-181b-1-5p	let-7b-5p	0.519377662	0.018931116	0.16316744	0.491862025
mir-223-3p	mir-30c-1-5p	0.773617806	6.30E-05	0.89367384	1.12E-07
mir-223-3p	mir-30c-2-5p	0.773617806	6.30E-05	0.89367384	1.12E-07
mir-629-5p	cpeptide	-0.08280001	0.728556238	0.316165337	0.174453222
mir-19b-2-3p	mir-106b-3p	0.857645293	1.34E-06	0.70346342	0.00053911
mir-19b-1-3p	mir-106b-3p	0.857645293	1.34E-06	0.70346342	0.00053911
let-7f-2-5p	mir-24-2-3p	0.626650522	0.003110622	0.816442043	1.13E-05
let-7f-2-5p	mir-24-1-3p	0.626650522	0.003110622	0.816442043	1.13E-05
mir-24-2-3p	let-7f-1-5p	0.626650522	0.003110622	0.816442043	1.13E-05
let-7f-1-5p	mir-24-1-3p	0.626650522	0.003110622	0.816442043	1.13E-05
mir-103a-2-3p	mir-107	0.298061231	0.201817014	-0.102219995	0.668044759
mir-103a-1-3p	mir-107	0.298061231	0.201817014	-0.102219995	0.668044759
mir-128-1-3p	mir-20b-5p	0.124594778	0.600714239	-0.277147508	0.236815125
mir-128-2-3p	mir-20b-5p	0.124594778	0.600714239	-0.277147508	0.236815125
mir-126-3p	mir-340-5p	0.734800733	0.000224135	0.873782087	4.84E-07
mir-103a-2-3p	mir-148b-3p	0.465128331	0.03878053	0.722840838	0.000317597
mir-103a-1-3p	mir-148b-3p	0.465128331	0.03878053	0.722840838	0.000317597
mir-192-5p	norepi	-0.026774314	0.91078536	-0.41061925	0.072116382
mir-182-5p	let-7b-5p	0.814373158	1.24E-05	0.913667097	1.84E-08
mir-584-5p	mir-93-5p	-0.105449464	0.658158926	-0.473906332	0.034782969
mir-21-5p	mir-486-2-5p	-0.40406363	0.077242874	-0.684542936	0.000870184
mir-584-5p	mir-24-2-3p	0.341269807	0.140868285	0.643782625	0.002191345
mir-584-5p	mir-24-1-3p	0.341269807	0.140868285	0.643782625	0.002191345
mir-224-5p	mir-30d-5p	0.271334009	0.247198514	0.596247516	0.005525612
mir-26a-2-5p	let-7i-5p	0.448394646	0.047379505	0.712057172	0.000428545
let-7i-5p	mir-26a-1-5p	0.448394646	0.047379505	0.712057172	0.000428545
mir-146a-5p	mir-486-2-5p	-0.377310317	0.101003381	-0.667135143	0.001312744
mir-16-2-5p	mir-107	0.967595092	3.33E-12	0.928111201	3.74E-09
mir-107	mir-16-1-5p	0.967595092	3.33E-12	0.928111201	3.74E-09
mir-19b-2-3p	mir-103a-2-3p	0.337259946	0.145901346	-0.057250499	0.810529984
mir-19b-2-3p	mir-103a-1-3p	0.337259946	0.145901346	-0.057250499	0.810529984
mir-103a-2-3p	mir-19b-1-3p	0.337259946	0.145901346	-0.057250499	0.810529984
mir-19b-1-3p	mir-103a-1-3p	0.337259946	0.145901346	-0.057250499	0.810529984
mir-194-2-5p	mir-629-5p	0.752122816	0.000130848	0.515020631	0.020136857
mir-629-5p	mir-194-1-5p	0.752122816	0.000130848	0.515020631	0.020136857
mir-22-3p	let-7b-5p	0.746411208	0.000156982	0.505523467	0.022977134
mir-122-5p	mir-320a	0.09429729	0.692518375	-0.303517722	0.193286317
mir-340-5p	mir-143-3p	-0.134725714	0.571183964	0.265667755	0.257594868
mir-181b-2-5p	mir-375	-0.423547735	0.062757508	-0.044305761	0.852857622
mir-181b-1-5p	mir-375	-0.423547735	0.062757508	-0.044305761	0.852857622
mir-1307-3p	mir-20b-5p	-0.636330429	0.00255847	-0.8209107	9.21E-06
mir-181a-2-5p	mir-28-3p	0.439051003	0.052778847	0.705495603	0.000510993
mir-28-3p	mir-181a-1-5p	0.439051003	0.052778847	0.705495603	0.000510993
mir-101-1-3p	mir-629-5p	0.894760964	1.02E-07	0.777258849	5.53E-05
mir-629-5p	mir-101-2-3p	0.894760964	1.02E-07	0.777258849	5.53E-05
mir-24-2-3p	mir-148a-3p	-0.201721798	0.393738221	0.199440594	0.399221473
mir-148a-3p	mir-24-1-3p	-0.201721798	0.393738221	0.199440594	0.399221473
mir-15a-5p	mir-28-5p	-0.375019622	0.1032599	-0.664460068	0.001395153
mir-151a-3p	mir-126-5p	0.513296716	0.020630479	0.750272117	0.000138873
mir-150-5p	mir-93-5p	-0.043788588	0.85455757	0.347296682	0.133534861
mir-144-3p	mir-425-5p	0.738889728	0.000198127	0.494757497	0.02657135
mir-16-2-5p	mir-320a	0.93694697	1.19E-09	0.86349321	9.42E-07
mir-320a	mir-16-1-5p	0.93694697	1.19E-09	0.86349321	9.42E-07
mir-128-1-3p	mir-486-2-5p	0.112224677	0.637597268	-0.284618166	0.223891558
mir-486-2-5p	mir-128-2-3p	0.112224677	0.637597268	-0.284618166	0.223891558
mir-30e-5p	let-7g-5p	0.723383441	0.000312733	0.866769037	7.67E-07
mir-182-5p	mir-224-5p	-0.107007698	0.653408305	-0.471875419	0.035677947
mir-181a-2-5p	mir-30d-5p	0.7587044	0.000105445	0.884948463	2.19E-07
mir-30d-5p	mir-181a-1-5p	0.7587044	0.000105445	0.884948463	2.19E-07
mir-7-3-5p	mir-20a-5p	0.914986536	1.61E-08	0.818574405	1.03E-05
mir-7-1-5p	mir-20a-5p	0.914986536	1.61E-08	0.818574405	1.03E-05
mir-20a-5p	mir-7-2-5p	0.914986536	1.61E-08	0.818574405	1.03E-05
mir-340-5p	mir-1307-3p	0.249713635	0.288333892	0.577915343	0.007612092

mir-224-5p	mir-24-2-3p	0.626374671	0.003127702	0.814282718	1.24E-05
mir-224-5p	mir-24-1-3p	0.626374671	0.003127702	0.814282718	1.24E-05
mir-10b-5p	mir-143-3p	0.395017463	0.084755855	0.013563173	0.954741951
let-7i-5p	mir-28-5p	0.266671508	0.255733318	0.589797856	0.006197883
mir-98-5p	mir-484	0.403732321	0.077508964	0.024187738	0.919375775
mir-223-3p	mir-23a-3p	0.65894182	0.001578948	0.832066002	5.39E-06
mir-126-3p	mir-224-5p	0.532872212	0.015557815	0.760753607	9.85E-05
mir-148a-3p	mir-15b-5p	-0.157385966	0.507535381	0.240335082	0.307413386
mir-16-2-5p	mir-101-1-3p	0.971916146	9.32E-13	0.938125912	1.01E-09
mir-16-2-5p	mir-101-2-3p	0.971916146	9.32E-13	0.938125912	1.01E-09
mir-101-1-3p	mir-16-1-5p	0.971916146	9.32E-13	0.938125912	1.01E-09
mir-16-1-5p	mir-101-2-3p	0.971916146	9.32E-13	0.938125912	1.01E-09
let-7b-5p	mir-20a-5p	0.901949503	5.55E-08	0.792823193	3.06E-05
mir-182-5p	mir-584-5p	-0.373866226	0.104409842	-0.66197306	0.001475626
mir-126-5p	mir-363-3p	0.205525443	0.384688852	-0.19227527	0.416714924
mir-374b-5p	mir-486-2-5p	-0.026797503	0.910708385	-0.405251183	0.076294673
mir-425-5p	mir-423-5p	0.827482023	6.75E-06	0.650994214	0.001879057
mir-148a-3p	mir-99b-5p	-0.338382075	0.144480399	0.05068657	0.831937448
mir-222-3p	mir-126-5p	0.473170792	0.03510506	0.110787594	0.641937963
mir-486-2-5p	let-7g-5p	0.609014298	0.004371184	0.29538366	0.206093426
mir-103a-2-3p	mir-30c-1-5p	0.591403668	0.006024536	0.794156863	2.90E-05
mir-103a-2-3p	mir-30c-2-5p	0.591403668	0.006024536	0.794156863	2.90E-05
mir-103a-1-3p	mir-30c-1-5p	0.591403668	0.006024536	0.794156863	2.90E-05
mir-103a-1-3p	mir-30c-2-5p	0.591403668	0.006024536	0.794156863	2.90E-05
mir-182-5p	mir-199a-1-5p	-0.42297604	0.063150894	-0.692997007	0.000705638
mir-182-5p	mir-199a-2-5p	-0.42297604	0.063150894	-0.692997007	0.000705638
mir-144-3p	mir-484	0.794166392	2.90E-05	0.592006018	0.005960549
mir-181b-2-5p	let-7g-5p	0.020294687	0.932321137	0.398612961	0.08170776
mir-181b-1-5p	let-7g-5p	0.020294687	0.932321137	0.398612961	0.08170776
mir-107	norepi	0.138329292	0.560834777	-0.256555902	0.274885601
mir-106b-3p	mir-374b-5p	-0.157526781	0.507150815	-0.508294666	0.02211745
mir-19b-2-3p	mir-28-3p	-0.165104236	0.486665111	-0.513910958	0.020453507
mir-19b-1-3p	mir-28-3p	-0.165104236	0.486665111	-0.513910958	0.020453507
mir-199a-1-5p	mir-15b-5p	-0.135532408	0.568860006	0.258600976	0.270943371
mir-199a-2-5p	mir-15b-5p	-0.135532408	0.568860006	0.258600976	0.270943371
mir-192-5p	mir-363-3p	0.836938675	4.21E-06	0.669746913	0.001236273
let-7a-3-5p	mir-143-3p	-0.126961653	0.593758915	0.266184371	0.256635687
let-7a-2-5p	mir-143-3p	-0.126961653	0.593758915	0.266184371	0.256635687
mir-143-3p	let-7a-1-5p	-0.126961653	0.593758915	0.266184371	0.256635687
mir-30d-5p	mir-106b-3p	-0.020639581	0.931173572	-0.397789691	0.08239841
mir-486-2-5p	mir-99a-5p	0.310648953	0.182506938	-0.078763718	0.741343539
mir-26b-5p	mir-199a-2-3p	-0.212958709	0.367343798	-0.548621172	0.01224991
mir-26b-5p	mir-199a-1-3p	-0.212958709	0.367343798	-0.548621172	0.01224991
mir-26b-5p	mir-199b-3p	-0.212958709	0.367343798	-0.548621172	0.01224991
mir-21-5p	mir-27a-3p	-0.034605152	0.884839721	0.349611848	0.130790885
mir-191-5p	mir-126-5p	0.588951836	0.006290845	0.791461392	3.23E-05
mir-143-3p	mir-28-5p	-0.167447667	0.480413556	0.22597707	0.338063334
mir-744-5p	mir-363-3p	-0.525604772	0.0173086	-0.75435621	0.000121696
mir-144-3p	mir-194-2-5p	0.816219308	1.14E-05	0.633090994	0.002733327
mir-144-3p	mir-194-1-5p	0.816219308	1.14E-05	0.633090994	0.002733327
mir-181b-2-5p	mir-25-3p	0.539607963	0.014064891	0.202315072	0.392319051
mir-25-3p	mir-181b-1-5p	0.539607963	0.014064891	0.202315072	0.392319051
mir-106b-3p	mir-99a-5p	0.254837434	0.278225755	-0.13647915	0.566137869
mir-28-5p	mir-28-3p	0.507514916	0.02235672	0.74288963	0.000175228
mir-340-5p	mir-125a-5p	0.784977244	4.15E-05	0.578834368	0.007494068
mir-29a-3p	mir-629-5p	0.367516027	0.110907835	-0.011873763	0.960374692
mir-148b-3p	mir-20a-5p	0.549551836	0.012073836	0.216828224	0.358493927
mir-98-5p	mir-451a	-0.193005747	0.414912879	-0.531914452	0.01578005
mir-320a	norepi	0.201550267	0.39414907	-0.19042118	0.421307751
mir-148a-3p	mir-107	0.214159645	0.364583963	-0.177629843	0.453722273
mir-146b-5p	mir-15a-5p	-0.750649705	0.000137202	-0.879033973	3.37E-07
mir-543	mir-10a-5p	0.588446989	0.006346861	0.271402379	0.247074735
mir-140-5p	mir-15b-5p	0.658806678	0.001583692	0.829810904	6.03E-06
mir-194-2-5p	let-7b-5p	0.812169271	1.37E-05	0.627069847	0.003084807

let-7b-5p	mir-194-1-5p	0.812169271	1.37E-05	0.627069847	0.003084807
mir-146b-5p	mir-126-5p	0.280596364	0.230790376	0.594807967	0.005670198
mir-584-5p	mir-363-3p	-0.408639052	0.073637269	-0.680824425	0.000952211
mir-146a-5p	mir-93-5p	-0.173140038	0.465396357	-0.516386771	0.019752399
mir-143-3p	mir-122-5p	0.325166312	0.161838892	-0.05899921	0.804847914
mir-340-5p	mir-543	0.694126326	0.000685799	0.429678998	0.058653989
mir-92a-2-3p	mir-30d-5p	-0.421677477	0.064051355	-0.688932098	0.000781121
mir-30d-5p	mir-92a-1-3p	-0.421677477	0.064051355	-0.688932098	0.000781121
mir-26a-2-5p	mir-20b-5p	-0.286515618	0.220684044	-0.598573822	0.005298384
mir-26a-1-5p	mir-20b-5p	-0.286515618	0.220684044	-0.598573822	0.005298384
mir-194-2-5p	mir-486-2-5p	0.827145944	6.86E-06	0.654461165	0.001742749
mir-194-1-5p	mir-486-2-5p	0.827145944	6.86E-06	0.654461165	0.001742749
mir-191-5p	mir-106b-3p	-0.256465402	0.275060876	-0.576863437	0.007749042
mir-16-2-5p	mir-126-5p	0.326371179	0.160199436	-0.05625782	0.813759573
mir-16-1-5p	mir-126-5p	0.326371179	0.160199436	-0.05625782	0.813759573
mir-143-3p	mir-23a-3p	-0.009146819	0.969470425	0.367722071	0.110692527
mir-103a-2-3p	mir-199a-1-5p	0.310085033	0.183344184	0.614003876	0.003977992
mir-103a-2-3p	mir-199a-2-5p	0.310085033	0.183344184	0.614003876	0.003977992
mir-199a-1-5p	mir-103a-1-3p	0.310085033	0.183344184	0.614003876	0.003977992
mir-103a-1-3p	mir-199a-2-5p	0.310085033	0.183344184	0.614003876	0.003977992
mir-28-3p	mir-185-5p	-0.085715148	0.719363375	-0.446689332	0.048331858
mir-744-5p	mir-221-3p	0.836144795	4.39E-06	0.922088384	7.55E-09
mir-106a-5p	mir-20a-5p	0.903516257	4.83E-08	0.799257787	2.36E-05
mir-122-5p	mir-15b-5p	-0.367633863	0.110784664	0.00852362	0.971549659
mir-148b-3p	mir-128-1-3p	0.500796788	0.024504383	0.737289984	0.000207976
mir-148b-3p	mir-128-2-3p	0.500796788	0.024504383	0.737289984	0.000207976
mir-221-3p	norepi	0.171791044	0.468933451	0.513261042	0.020640795
mir-10b-5p	mir-374b-5p	0.10404512	0.662451216	-0.281350269	0.229486775
mir-15a-5p	mir-139-5p	-0.416467981	0.067761352	-0.684204934	0.000877384
mir-126-3p	mir-92a-2-3p	-0.777683801	5.44E-05	-0.892234183	1.25E-07
mir-126-3p	mir-92a-1-3p	-0.777683801	5.44E-05	-0.892234183	1.25E-07
mir-122-5p	mir-150-5p	0.691713288	0.000728777	0.428410462	0.059485819
mir-122-5p	mir-20b-5p	0.047602781	0.842035281	-0.33246121	0.152088186
mir-25-3p	mir-99a-5p	0.324615999	0.162591537	-0.056334697	0.813509357
let-7d-5p	mir-486-2-5p	-0.301130857	0.19698765	-0.606675432	0.004566253
mir-24-2-3p	mir-28-5p	0.756942632	0.000111789	0.88129876	2.87E-07
mir-28-5p	mir-24-1-3p	0.756942632	0.000111789	0.88129876	2.87E-07
mir-629-5p	mir-20b-5p	0.917882234	1.19E-08	0.828403985	6.45E-06
mir-361-5p	mir-223-3p	0.643854701	0.002188022	0.820033654	9.59E-06
mir-3615	mir-23a-3p	-0.339422989	0.143170981	-0.632488774	0.002766906
mir-744-5p	mir-375	-0.158466013	0.504589343	0.228175627	0.333257703
mir-125a-5p	mir-30a-5p	-0.010781934	0.964015987	0.363812502	0.114829523
mir-222-3p	mir-151a-3p	0.165652158	0.485199835	-0.220937038	0.349232285
mir-340-5p	mir-486-2-5p	-0.242742562	0.302444497	-0.564505464	0.009514254
mir-103a-2-3p	mir-423-3p	0.516430886	0.019740082	0.745580754	0.000161131
mir-423-3p	mir-103a-1-3p	0.516430886	0.019740082	0.745580754	0.000161131
mir-101-1-3p	mir-28-3p	-0.211106112	0.371624439	-0.541139673	0.013742059
mir-101-2-3p	mir-28-3p	-0.211106112	0.371624439	-0.541139673	0.013742059
mir-423-5p	mir-15a-5p	0.514612465	0.020252872	0.175811653	0.458431516
mir-98-5p	mir-148a-3p	-0.268068288	0.253157111	0.115934905	0.626443231
mir-361-5p	let-7i-5p	0.078072022	0.743541558	0.437742932	0.053570702
mir-144-3p	mir-28-5p	-0.048538255	0.8389695	-0.413384201	0.070032227
mir-17-5p	mir-15a-5p	0.641173655	0.002314504	0.353519937	0.126249989
mir-425-5p	mir-363-3p	0.697207174	0.000634061	0.439288616	0.052635969
mir-374b-5p	mir-150-5p	0.129510433	0.586306974	-0.254232437	0.279407656
mir-17-5p	mir-93-5p	0.928041555	3.78E-09	0.849424935	2.16E-06
mir-182-5p	mir-29a-3p	0.338693692	0.144087524	-0.037010428	0.876891807
mir-584-5p	mir-486-2-5p	-0.293374991	0.209340703	-0.59911296	0.005246837
mir-199a-2-3p	mir-93-5p	-0.128067789	0.590520009	-0.476340628	0.033733397
mir-199a-1-3p	mir-93-5p	-0.128067789	0.590520009	-0.476340628	0.033733397
mir-199b-3p	mir-93-5p	-0.128067789	0.590520009	-0.476340628	0.033733397
mir-7-3-5p	mir-484	0.765808157	8.29E-05	0.551615698	0.011690668
mir-7-1-5p	mir-484	0.765808157	8.29E-05	0.551615698	0.011690668
mir-7-2-5p	mir-484	0.765808157	8.29E-05	0.551615698	0.011690668

mir-26b-5p	mir-584-5p	-0.194847169	0.410388922	-0.527525623	0.016831233
mir-106a-5p	mir-224-5p	0.086383575	0.71726066	-0.293693404	0.208823702
mir-22-3p	mir-451a	0.727129711	0.000280853	0.488135917	0.028993052
mir-16-2-5p	mir-181b-2-5p	0.55992297	0.0102466	0.239091936	0.309998372
mir-16-2-5p	mir-181b-1-5p	0.55992297	0.0102466	0.239091936	0.309998372
mir-181b-2-5p	mir-16-1-5p	0.55992297	0.0102466	0.239091936	0.309998372
mir-181b-1-5p	mir-16-1-5p	0.55992297	0.0102466	0.239091936	0.309998372
mir-106a-5p	mir-125b-1-5p	0.325858408	0.160895753	-0.050572242	0.832311372
mir-106a-5p	mir-125b-2-5p	0.325858408	0.160895753	-0.050572242	0.832311372
mir-199a-2-3p	norepi	0.270131857	0.249381415	0.581833422	0.007119287
mir-199a-1-3p	norepi	0.270131857	0.249381415	0.581833422	0.007119287
mir-199b-3p	norepi	0.270131857	0.249381415	0.581833422	0.007119287
mir-21-5p	mir-374b-5p	0.653026967	0.001798095	0.823794227	8.05E-06
mir-107	mir-28-3p	-0.253593214	0.280659804	-0.569505422	0.008764364
mir-26a-2-5p	mir-28-5p	0.716288223	0.00038162	0.858473921	1.28E-06
mir-28-5p	mir-26a-1-5p	0.716288223	0.00038162	0.858473921	1.28E-06
mir-30d-5p	mir-15a-5p	-0.23987292	0.308372875	-0.559474969	0.010320586
let-7i-5p	mir-30d-5p	0.803254741	2.00E-05	0.904258557	4.52E-08
mir-26b-5p	mir-148a-3p	0.272691791	0.244747762	-0.107105556	0.653110387
let-7i-5p	mir-223-3p	0.283813303	0.225261274	0.590635528	0.006106953
mir-7-3-5p	mir-1307-3p	-0.541809881	0.013602694	-0.75883404	0.00010499
mir-1307-3p	mir-7-1-5p	-0.541809881	0.013602694	-0.75883404	0.00010499
mir-1307-3p	mir-7-2-5p	-0.541809881	0.013602694	-0.75883404	0.00010499
mir-29a-3p	mir-451a	0.499341963	0.024990264	0.160350844	0.499467915
mir-7-3-5p	mir-126-5p	0.212578736	0.368219473	-0.169121544	0.475972793
mir-7-1-5p	mir-126-5p	0.212578736	0.368219473	-0.169121544	0.475972793
mir-7-2-5p	mir-126-5p	0.212578736	0.368219473	-0.169121544	0.475972793
mir-27b-3p	cpeptide	0.078533107	0.742076142	-0.29776363	0.202289377
mir-98-5p	mir-221-3p	0.700322572	0.000585147	0.849299046	2.17E-06
mir-101-1-3p	mir-199a-2-3p	-0.206152715	0.383207745	-0.533192764	0.015483999
mir-101-1-3p	mir-199a-1-3p	-0.206152715	0.383207745	-0.533192764	0.015483999
mir-101-1-3p	mir-199b-3p	-0.206152715	0.383207745	-0.533192764	0.015483999
mir-199a-2-3p	mir-101-2-3p	-0.206152715	0.383207745	-0.533192764	0.015483999
mir-199a-1-3p	mir-101-2-3p	-0.206152715	0.383207745	-0.533192764	0.015483999
mir-101-2-3p	mir-199b-3p	-0.206152715	0.383207745	-0.533192764	0.015483999
mir-26b-5p	mir-484	0.821590626	8.92E-06	0.650645587	0.001893245
mir-29a-3p	mir-146b-5p	-0.168428704	0.477808384	0.211956218	0.369656674
mir-26a-2-5p	mir-99b-5p	0.766163228	8.19E-05	0.555227349	0.011043856
mir-99b-5p	mir-26a-1-5p	0.766163228	8.19E-05	0.555227349	0.011043856
mir-378a-3p	mir-28-3p	-0.085811144	0.719061273	-0.439041954	0.052784294
mir-106a-5p	mir-744-5p	-0.556008868	0.010907786	-0.7665804	8.07E-05
mir-3615	let-7d-5p	-0.500813054	0.024498993	-0.732859253	0.000237471
mir-1307-3p	mir-24-2-3p	0.49395892	0.02685465	0.728522365	0.000269731
mir-1307-3p	mir-24-1-3p	0.49395892	0.02685465	0.728522365	0.000269731
mir-15a-5p	mir-99b-5p	-0.84355361	2.98E-06	-0.690689036	0.000747698
mir-103a-2-3p	mir-128-1-3p	0.801308494	2.17E-05	0.902610217	5.23E-08
mir-103a-2-3p	mir-128-2-3p	0.801308494	2.17E-05	0.902610217	5.23E-08
mir-103a-1-3p	mir-128-1-3p	0.801308494	2.17E-05	0.902610217	5.23E-08
mir-103a-1-3p	mir-128-2-3p	0.801308494	2.17E-05	0.902610217	5.23E-08
mir-423-3p	mir-151a-5p	0.523836378	0.0177576	0.746648205	0.000155815
mir-22-3p	mir-146b-5p	-0.518694253	0.01911633	-0.188652918	0.425713125
mir-199a-2-3p	mir-139-5p	0.711394138	0.000436326	0.854757387	1.59E-06
mir-139-5p	mir-199a-1-3p	0.711394138	0.000436326	0.854757387	1.59E-06
mir-139-5p	mir-199b-3p	0.711394138	0.000436326	0.854757387	1.59E-06
mir-181a-2-5p	mir-144-3p	0.171133568	0.47066228	-0.207457066	0.380138167
mir-144-3p	mir-181a-1-5p	0.171133568	0.47066228	-0.207457066	0.380138167
mir-181a-2-5p	mir-486-2-5p	0.072568381	0.76109745	-0.301009672	0.197176829
mir-486-2-5p	mir-181a-1-5p	0.072568381	0.76109745	-0.301009672	0.197176829
mir-340-5p	mir-27a-3p	-0.06460789	0.786688442	0.308112844	0.186292698
mir-629-5p	mir-24-2-3p	-0.469500489	0.036747157	-0.71259334	0.00042234
mir-629-5p	mir-24-1-3p	-0.469500489	0.036747157	-0.71259334	0.00042234
mir-143-3p	mir-139-5p	-0.349227848	0.131243222	0.01806804	0.939732857
mir-199a-1-5p	mir-23a-3p	0.407325501	0.074659269	0.672335418	0.001164218
mir-23a-3p	mir-199a-2-5p	0.407325501	0.074659269	0.672335418	0.001164218

mir-378a-3p	mir-150-5p	0.307923618	0.186577274	0.604875127	0.004721253
mir-15a-5p	mir-107	0.873375231	4.98E-07	0.746322146	0.000157423
let-7i-5p	mir-10a-5p	0.056815273	0.811945591	0.413076459	0.070261937
mir-126-3p	mir-106b-3p	-0.318640567	0.170919531	-0.612263713	0.00411165
mir-22-3p	mir-423-3p	-0.302998322	0.194087754	0.069453622	0.77108377
mir-30d-5p	cpeptide	-0.514676416	0.020234659	-0.184721065	0.435596238
mir-15a-5p	mir-221-3p	-0.722172579	0.000323676	-0.86024393	1.15E-06
mir-30e-5p	cpeptide	-0.337456907	0.14565123	0.03081638	0.89738044
mir-29a-3p	mir-30a-5p	0.277445181	0.23629116	0.582827691	0.00699848
mir-125a-5p	mir-151a-5p	0.553574461	0.011336174	0.237123459	0.31411834
mir-22-3p	mir-340-5p	-0.151505594	0.523718628	0.225125169	0.339936301
mir-27b-3p	mir-101-1-3p	-0.153920252	0.517044171	-0.490510758	0.028105345
mir-27b-3p	mir-101-2-3p	-0.153920252	0.517044171	-0.490510758	0.028105345
mir-19b-2-3p	mir-148b-3p	0.512927582	0.020737417	0.18348154	0.438736771
mir-19b-1-3p	mir-148b-3p	0.512927582	0.020737417	0.18348154	0.438736771
mir-194-2-5p	mir-106b-3p	0.77799975	5.38E-05	0.577856242	0.007619734
mir-194-1-5p	mir-106b-3p	0.77799975	5.38E-05	0.577856242	0.007619734
let-7f-2-5p	mir-151a-3p	0.731016455	0.000250751	0.864768198	8.70E-07
mir-151a-3p	let-7f-1-5p	0.731016455	0.000250751	0.864768198	8.70E-07
mir-146a-5p	mir-7-3-5p	-0.341497006	0.140586811	-0.627221351	0.003075524
mir-146a-5p	mir-7-1-5p	-0.341497006	0.140586811	-0.627221351	0.003075524
mir-146a-5p	mir-7-2-5p	-0.341497006	0.140586811	-0.627221351	0.003075524
mir-199a-2-3p	mir-10a-5p	0.226487213	0.336944652	0.545172324	0.012920588
mir-199a-1-3p	mir-10a-5p	0.226487213	0.336944652	0.545172324	0.012920588
mir-199b-3p	mir-10a-5p	0.226487213	0.336944652	0.545172324	0.012920588
mir-484	mir-93-5p	0.85746428	1.36E-06	0.717615182	0.000367836
mir-139-5p	mir-28-5p	0.671708937	0.001181325	0.832033548	5.40E-06
mir-629-5p	let-7g-5p	0.410671497	0.072076575	0.055575262	0.815981893
mir-26a-2-5p	mir-543	0.722300293	0.000322506	0.486674826	0.029550051
mir-543	mir-26a-1-5p	0.722300293	0.000322506	0.486674826	0.029550051
mir-16-2-5p	mir-150-5p	0.0875115	0.71371692	0.436840906	0.054122008
mir-16-1-5p	mir-150-5p	0.0875115	0.71371692	0.436840906	0.054122008
mir-21-5p	mir-106b-3p	-0.438327537	0.053215693	-0.691348185	0.000735475
mir-26b-5p	mir-150-5p	-0.043980458	0.853926822	0.324085359	0.163319555
mir-144-3p	mir-125b-1-5p	0.310762661	0.182338431	-0.058742301	0.805682103
mir-144-3p	mir-125b-2-5p	0.310762661	0.182338431	-0.058742301	0.805682103
mir-21-5p	mir-151a-5p	0.449013549	0.047037457	0.69806502	0.000620259
mir-191-5p	mir-363-3p	-0.190632367	0.420783248	-0.517553617	0.019428677
mir-26b-5p	mir-126-5p	0.367466459	0.110959676	0.005785771	0.980686097
mir-122-5p	mir-20a-5p	0.084484608	0.723239431	-0.286585356	0.220566732
let-7b-5p	cpeptide	-0.192535914	0.416071447	0.182470835	0.441306324
mir-423-5p	mir-10a-5p	-0.130170518	0.584383549	0.243588646	0.300709895
mir-15a-5p	mir-363-3p	0.805525835	1.82E-05	0.903987262	4.63E-08
mir-146a-5p	mir-25-3p	-0.318554626	0.171041394	-0.610331862	0.004264363
let-7i-5p	mir-93-5p	0.460700063	0.040927876	0.118259749	0.619493454
let-7i-5p	mir-148a-3p	-0.099583822	0.676153724	0.271902731	0.246170086
mir-19b-2-3p	mir-199a-2-3p	-0.107533447	0.651808323	-0.45163274	0.045610868
mir-19b-2-3p	mir-199a-1-3p	-0.107533447	0.651808323	-0.45163274	0.045610868
mir-19b-2-3p	mir-199b-3p	-0.107533447	0.651808323	-0.45163274	0.045610868
mir-199a-2-3p	mir-19b-1-3p	-0.107533447	0.651808323	-0.45163274	0.045610868
mir-19b-1-3p	mir-199a-1-3p	-0.107533447	0.651808323	-0.45163274	0.045610868
mir-19b-1-3p	mir-199b-3p	-0.107533447	0.651808323	-0.45163274	0.045610868
mir-340-5p	mir-128-1-3p	0.782854994	4.49E-05	0.891958611	1.28E-07
mir-340-5p	mir-128-2-3p	0.782854994	4.49E-05	0.891958611	1.28E-07
mir-16-2-5p	mir-99a-5p	0.283629029	0.225575639	-0.086790785	0.715980634
mir-16-1-5p	mir-99a-5p	0.283629029	0.225575639	-0.086790785	0.715980634
mir-25-3p	mir-128-1-3p	0.151765776	0.522997512	-0.221757483	0.347399715
mir-25-3p	mir-128-2-3p	0.151765776	0.522997512	-0.221757483	0.347399715
mir-122-5p	mir-629-5p	-0.098876054	0.678336745	-0.44428198	0.049701178
mir-361-5p	mir-139-5p	0.611284277	0.004188499	0.796612626	2.63E-05
mir-148b-3p	mir-28-3p	0.104835665	0.660033708	0.449014875	0.047036727
mir-181b-2-5p	mir-30d-5p	0.024837699	0.91721631	0.382352372	0.096163292
mir-181b-1-5p	mir-30d-5p	0.024837699	0.91721631	0.382352372	0.096163292
mir-146b-5p	mir-148b-3p	0.123965804	0.602568187	0.464088244	0.039276852

mir-106b-3p	mir-363-3p	0.93697377	1.18E-09	0.870480117	6.03E-07
mir-144-3p	mir-15b-5p	0.602731081	0.004911489	0.309708088	0.183905278
mir-222-3p	mir-106a-5p	0.738813726	0.000198585	0.868033589	7.07E-07
mir-26a-2-5p	mir-486-2-5p	-0.361730131	0.11707782	-0.638722806	0.00243541
mir-486-2-5p	mir-26a-1-5p	-0.361730131	0.11707782	-0.638722806	0.00243541
mir-199a-2-3p	let-7d-3p	0.94932631	1.74E-10	0.89526706	9.79E-08
mir-199a-1-3p	let-7d-3p	0.94932631	1.74E-10	0.89526706	9.79E-08
mir-199b-3p	let-7d-3p	0.94932631	1.74E-10	0.89526706	9.79E-08
mir-361-5p	mir-92a-2-3p	-0.730533611	0.000254334	-0.86347248	9.44E-07
mir-361-5p	mir-92a-1-3p	-0.730533611	0.000254334	-0.86347248	9.44E-07
mir-23a-3p	mir-20b-5p	-0.240045256	0.308014878	-0.552290316	0.011567578
mir-378a-3p	mir-99b-5p	-0.410692235	0.072060779	-0.059837356	0.80212785
mir-126-3p	mir-106a-5p	-0.250604448	0.286560481	-0.559790459	0.010268439
mir-98-5p	mir-10a-5p	0.282753945	0.227072407	0.582993208	0.006978533
mir-451a	mir-224-5p	0.159667111	0.50132282	-0.211891858	0.369805442
mir-224-5p	mir-28-5p	0.4748087	0.034390976	0.712360433	0.000425026
mir-182-5p	mir-423-5p	0.593741774	0.005779278	0.298505042	0.201113948
mir-27a-3p	mir-30a-5p	0.34373549	0.137834675	0.625396117	0.003188922
mir-451a	mir-128-1-3p	0.099896871	0.675188955	-0.268516345	0.25233423
mir-451a	mir-128-2-3p	0.099896871	0.675188955	-0.268516345	0.25233423
let-7i-5p	mir-99a-5p	-0.133267762	0.575394536	0.23660401	0.315210992
mir-451a	mir-484	0.692701046	0.000710917	0.444898465	0.049347722
mir-92a-2-3p	mir-28-5p	-0.531191988	0.015949368	-0.74712772	0.000153476
mir-28-5p	mir-92a-1-3p	-0.531191988	0.015949368	-0.74712772	0.000153476
mir-361-5p	mir-106a-5p	-0.484822716	0.03026815	-0.718132766	0.000362576
mir-151a-5p	mir-23a-3p	0.314804779	0.176416653	0.604429159	0.004760313
mir-15a-5p	mir-186-5p	0.819036299	1.00E-05	0.652563925	0.001816276
mir-22-3p	mir-107	0.819700397	9.74E-06	0.653738225	0.001770466
mir-26b-5p	mir-194-2-5p	0.805118564	1.85E-05	0.628812644	0.002979415
mir-26b-5p	mir-194-1-5p	0.805118564	1.85E-05	0.628812644	0.002979415
mir-26b-5p	mir-106b-3p	0.916860344	1.33E-08	0.832381257	5.31E-06
mir-532-5p	mir-199a-2-3p	-0.523366132	0.01787855	-0.741548994	0.000182636
mir-532-5p	mir-199a-1-3p	-0.523366132	0.01787855	-0.741548994	0.000182636
mir-532-5p	mir-199b-3p	-0.523366132	0.01787855	-0.741548994	0.000182636
mir-98-5p	mir-140-5p	0.62967054	0.002928646	0.35226176	0.12769947
mir-181a-2-5p	mir-223-3p	0.663430667	0.001428002	0.82479255	7.68E-06
mir-223-3p	mir-181a-1-5p	0.663430667	0.001428002	0.82479255	7.68E-06
mir-27b-3p	let-7b-5p	-0.472305106	0.035487114	-0.70916006	0.000463433
mir-25-3p	mir-27a-3p	0.657417495	0.001633157	0.393956614	0.085671032
mir-151a-3p	mir-23a-3p	0.684321571	0.000874894	0.83633745	4.35E-06
mir-29a-3p	mir-99b-5p	-0.060620771	0.799587386	0.301122272	0.197001048
mir-27b-3p	mir-320a	-0.242612101	0.3027125	-0.550416362	0.011912115
mir-146b-5p	mir-584-5p	0.551341307	0.011741036	0.757976401	0.000108028
mir-186-5p	mir-93-5p	0.927133196	4.21E-09	0.852891068	1.77E-06
let-7a-3-5p	mir-28-3p	0.506262409	0.022745352	0.729838468	0.000259569
let-7a-2-5p	mir-28-3p	0.506262409	0.022745352	0.729838468	0.000259569
let-7a-1-5p	mir-28-3p	0.506262409	0.022745352	0.729838468	0.000259569
mir-21-5p	mir-223-3p	0.74177045	0.000181394	0.868008708	7.08E-07
mir-744-5p	mir-27a-3p	-0.361195481	0.117660128	-0.007813311	0.973919739
mir-19b-2-3p	mir-425-5p	0.843635975	2.96E-06	0.697971418	0.000621752
mir-425-5p	mir-19b-1-3p	0.843635975	2.96E-06	0.697971418	0.000621752
mir-16-2-5p	mir-186-5p	0.93738267	1.12E-09	0.873108547	5.07E-07
mir-16-1-5p	mir-186-5p	0.93738267	1.12E-09	0.873108547	5.07E-07
let-7f-2-5p	mir-361-5p	0.759665576	0.000102116	0.877633603	3.72E-07
mir-361-5p	let-7f-1-5p	0.759665576	0.000102116	0.877633603	3.72E-07
mir-423-5p	mir-150-5p	-0.262649242	0.263244593	0.100250676	0.674099166
mir-106b-3p	mir-375	-0.20289028	0.390945805	0.16232043	0.494143286
let-7i-5p	mir-146a-5p	0.247279542	0.29321402	0.552524333	0.011525126
mir-26a-2-5p	mir-106b-3p	-0.433094001	0.056458453	-0.682122401	0.000922868
mir-106b-3p	mir-26a-1-5p	-0.433094001	0.056458453	-0.682122401	0.000922868
mir-199a-1-5p	mir-99a-5p	-0.330082386	0.155221777	0.026153747	0.912845523
mir-99a-5p	mir-199a-2-5p	-0.330082386	0.155221777	0.026153747	0.912845523
mir-107	mir-150-5p	0.011410214	0.961920561	0.363049814	0.115649353
mir-106a-5p	mir-185-5p	0.901890002	5.58E-08	0.80522695	1.84E-05

mir-185-5p	cpeptide	-0.223067339	0.344485596	0.14110872	0.552909649
mir-106a-5p	mir-10b-5p	-0.038107988	0.873268787	0.31911971	0.170241196
mir-629-5p	mir-99a-5p	0.060520443	0.799912624	-0.298565317	0.201018588
mir-125b-1-5p	mir-375	0.508211317	0.022142928	0.730020972	0.000258185
mir-375	mir-125b-2-5p	0.508211317	0.022142928	0.730020972	0.000258185
mir-16-2-5p	mir-15b-5p	0.647777243	0.002013413	0.382570381	0.095957911
mir-16-1-5p	mir-15b-5p	0.647777243	0.002013413	0.382570381	0.095957911
mir-151a-5p	mir-28-3p	0.289497093	0.215705155	0.582562873	0.007030491
mir-148a-3p	mir-185-5p	0.246511661	0.29476399	-0.115961845	0.626362524
mir-423-3p	mir-340-5p	0.745916202	0.000159444	0.869700787	6.35E-07
mir-19b-2-3p	mir-126-5p	0.481516559	0.031583967	0.155958644	0.511441296
mir-19b-1-3p	mir-126-5p	0.481516559	0.031583967	0.155958644	0.511441296
mir-103a-2-3p	mir-151a-5p	0.344646214	0.136725904	0.621153236	0.003466005
mir-103a-1-3p	mir-151a-5p	0.344646214	0.136725904	0.621153236	0.003466005
mir-199a-1-5p	mir-543	0.544625002	0.013029695	0.238569482	0.311088661
mir-543	mir-199a-2-5p	0.544625002	0.013029695	0.238569482	0.311088661
mir-222-3p	mir-26b-5p	0.782901576	4.49E-05	0.889649553	1.53E-07
mir-484	cpeptide	-0.281884252	0.228566348	0.077466323	0.745467874
mir-320a	mir-150-5p	0.044827007	0.851144917	0.390342816	0.088843365
mir-16-2-5p	mir-10b-5p	-0.042190668	0.859813809	0.314099124	0.17744089
mir-10b-5p	mir-16-1-5p	-0.042190668	0.859813809	0.314099124	0.17744089
mir-30d-5p	mir-375	-0.17686213	0.455707656	0.186313551	0.431578877
mir-320a	mir-375	-0.148160983	0.533029765	0.214566523	0.363651621
mir-151a-3p	mir-320a	0.190060172	0.422205159	-0.172861134	0.466126537
mir-125a-5p	mir-151a-3p	0.519206706	0.018977313	0.736212537	0.000214844
mir-146b-5p	mir-543	0.801264758	2.18E-05	0.6263975	0.003126286
let-7d-5p	mir-93-5p	-0.205527313	0.384684432	-0.519060985	0.019016761
mir-3615	mir-150-5p	0.22769316	0.334308817	0.535611829	0.014935966
let-7f-2-5p	mir-20b-5p	-0.308018853	0.186434014	-0.594375027	0.005714285
let-7f-1-5p	mir-20b-5p	-0.308018853	0.186434014	-0.594375027	0.005714285
mir-10b-5p	mir-28-3p	0.038302874	0.872625725	-0.316476956	0.174005627
mir-92a-2-3p	mir-126-5p	-0.295921141	0.205230219	-0.585629274	0.006667079
mir-126-5p	mir-92a-1-3p	-0.295921141	0.205230219	-0.585629274	0.006667079
mir-92a-2-3p	mir-584-5p	-0.34329871	0.138368677	-0.619215388	0.003599045
mir-584-5p	mir-92a-1-3p	-0.34329871	0.138368677	-0.619215388	0.003599045
mir-584-5p	mir-451a	-0.44407808	0.049818508	-0.687474681	0.000809796
mir-140-5p	mir-25-3p	0.563980412	0.009595939	0.266699733	0.255681095
mir-25-3p	mir-223-3p	-0.113453135	0.633895699	-0.445534716	0.048984955
mir-22-3p	mir-30e-5p	0.861555383	1.06E-06	0.930806266	2.68E-09
let-7d-5p	mir-106b-3p	-0.387444784	0.091449164	-0.649071162	0.001958438
mir-29a-3p	mir-106b-3p	0.349184135	0.131294784	-0.000243574	0.999186835
mir-146b-5p	mir-629-5p	-0.705246716	0.000514368	-0.846002958	2.61E-06
let-7b-5p	mir-15b-5p	0.507605075	0.02232895	0.192796708	0.415428135
mir-7-3-5p	mir-128-1-3p	0.118236816	0.619561861	-0.240623985	0.306814515
mir-7-3-5p	mir-128-2-3p	0.118236816	0.619561861	-0.240623985	0.306814515
mir-7-1-5p	mir-128-1-3p	0.118236816	0.619561861	-0.240623985	0.306814515
mir-7-1-5p	mir-128-2-3p	0.118236816	0.619561861	-0.240623985	0.306814515
mir-128-1-3p	mir-7-2-5p	0.118236816	0.619561861	-0.240623985	0.306814515
mir-7-2-5p	mir-128-2-3p	0.118236816	0.619561861	-0.240623985	0.306814515
mir-191-5p	mir-30c-1-5p	0.831585828	5.52E-06	0.679995695	0.000971355
mir-191-5p	mir-30c-2-5p	0.831585828	5.52E-06	0.679995695	0.000971355
mir-191-5p	mir-486-2-5p	-0.124411416	0.601254469	-0.453567233	0.044578761
mir-151a-3p	mir-30c-1-5p	0.461371065	0.040596715	0.697816508	0.000624231
mir-151a-3p	mir-30c-2-5p	0.461371065	0.040596715	0.697816508	0.000624231
mir-27b-3p	mir-92a-2-3p	-0.590762437	0.006093273	-0.778941415	5.20E-05
mir-27b-3p	mir-92a-1-3p	-0.590762437	0.006093273	-0.778941415	5.20E-05
mir-192-5p	mir-20b-5p	0.839830765	3.63E-06	0.694626058	0.000677171
let-7b-5p	mir-99a-5p	0.22953361	0.330309646	-0.128379493	0.589608644
mir-22-3p	mir-30d-5p	0.106744092	0.654211071	0.438094796	0.053356812
mir-222-3p	mir-148b-3p	0.379667612	0.098718934	0.03704896	0.876764573
mir-30c-1-5p	let-7d-3p	0.842011737	3.23E-06	0.699086453	0.000604157
let-7d-3p	mir-30c-2-5p	0.842011737	3.23E-06	0.699086453	0.000604157
mir-106a-5p	mir-101-1-3p	0.942141124	5.59E-10	0.884100993	2.34E-07
mir-106a-5p	mir-101-2-3p	0.942141124	5.59E-10	0.884100993	2.34E-07

mir-3615	mir-192-5p	0.871988447	5.46E-07	0.752575839	0.000128945
mir-224-5p	mir-151a-3p	0.503351898	0.023669137	0.724104207	0.000306371
mir-125a-5p	mir-223-3p	0.746286183	0.000157601	0.538786901	0.01424044
mir-122-5p	mir-125b-1-5p	0.634261245	0.002669046	0.368289538	0.110101107
mir-122-5p	mir-125b-2-5p	0.634261245	0.002669046	0.368289538	0.110101107
mir-122-5p	mir-374b-5p	-0.094800905	0.690953617	0.259983415	0.268298644
mir-126-3p	mir-451a	-0.186731349	0.430528159	-0.500528893	0.024593288
mir-16-2-5p	let-7i-5p	0.450489994	0.046229134	0.123806937	0.603036832
let-7i-5p	mir-16-1-5p	0.450489994	0.046229134	0.123806937	0.603036832
let-7d-5p	mir-363-3p	-0.378339543	0.100001279	-0.640390841	0.002352564
mir-15a-5p	mir-20a-5p	0.76672458	8.03E-05	0.572852402	0.008289857
mir-23a-3p	mir-28-3p	0.612448055	0.004097318	0.790706214	3.33E-05
mir-181a-2-5p	let-7d-5p	0.602562601	0.004926702	0.784647013	4.20E-05
let-7d-5p	mir-181a-1-5p	0.602562601	0.004926702	0.784647013	4.20E-05
mir-140-5p	mir-27a-3p	0.452039539	0.045392318	0.689710951	0.000766152
mir-140-5p	mir-20b-5p	0.551917604	0.011635453	0.255393951	0.277141312
mir-144-3p	mir-224-5p	0.099520229	0.676349767	-0.254105686	0.279655669
mir-744-5p	mir-10a-5p	0.151237908	0.524461028	0.471532867	0.035830653
mir-425-5p	norepi	0.342609432	0.13921434	-0.00256109	0.991450025
mir-425-5p	mir-107	0.785071064	4.13E-05	0.603767473	0.004818758
mir-26a-2-5p	mir-15a-5p	-0.548252378	0.012320254	-0.750859961	0.000136279
mir-15a-5p	mir-26a-1-5p	-0.548252378	0.012320254	-0.750859961	0.000136279
mir-24-2-3p	mir-148b-3p	0.121807868	0.60894668	0.447395141	0.047935926
mir-148b-3p	mir-24-1-3p	0.121807868	0.60894668	0.447395141	0.047935926
mir-151a-3p	mir-93-5p	0.026178841	0.912762208	-0.321026145	0.167560471
mir-98-5p	mir-320a	-0.026072466	0.913115403	-0.366921876	0.111530388
mir-15a-5p	mir-128-1-3p	-0.295003803	0.206704936	-0.580021985	0.007343772
mir-15a-5p	mir-128-2-3p	-0.295003803	0.206704936	-0.580021985	0.007343772
mir-1307-3p	mir-543	0.375728181	0.102558039	0.637219042	0.00251217
mir-126-3p	mir-374b-5p	0.697693123	0.000626211	0.839967196	3.60E-06
mir-143-3p	mir-99a-5p	0.245043303	0.297741816	-0.107705484	0.65128509
mir-26b-5p	let-7i-5p	0.494027436	0.02683025	0.181103188	0.444795844
mir-3615	mir-1307-3p	-0.561036772	0.010064523	-0.758384134	0.000106575
mir-223-3p	mir-125b-1-5p	-0.112951468	0.635406309	0.239803203	0.308517769
mir-223-3p	mir-125b-2-5p	-0.112951468	0.635406309	0.239803203	0.308517769
mir-29a-3p	mir-20b-5p	0.468613117	0.03715299	0.149336102	0.529749628
mir-629-5p	mir-363-3p	0.919625807	9.90E-09	0.842229316	3.20E-06
mir-29a-3p	mir-3615	0.449511419	0.04676368	0.125676497	0.597531311
mir-423-5p	cpeptide	-0.244819319	0.298197663	0.107303612	0.652507588
mir-92a-2-3p	mir-10b-5p	-0.297109754	0.203329814	0.051134951	0.830471303
mir-10b-5p	mir-92a-1-3p	-0.297109754	0.203329814	0.051134951	0.830471303
mir-126-3p	mir-126-5p	0.651044896	0.001877002	0.812551462	1.34E-05
mir-16-2-5p	cpeptide	-0.155504345	0.512687502	0.197853037	0.403062067
mir-16-1-5p	cpeptide	-0.155504345	0.512687502	0.197853037	0.403062067
mir-222-3p	mir-30c-1-5p	-0.306756375	0.188339161	0.040287445	0.866081713
mir-222-3p	mir-30c-2-5p	-0.306756375	0.188339161	0.040287445	0.866081713
mir-24-2-3p	mir-30e-5p	-0.181519047	0.443733272	0.171914656	0.468608772
mir-30e-5p	mir-24-1-3p	-0.181519047	0.443733272	0.171914656	0.468608772
mir-181b-2-5p	mir-532-5p	0.517158245	0.019537889	0.212496918	0.368408184
mir-532-5p	mir-181b-1-5p	0.517158245	0.019537889	0.212496918	0.368408184
mir-224-5p	mir-99a-5p	0.369727853	0.108612297	0.63200697	0.002794017
mir-125b-1-5p	let-7g-5p	0.00815382	0.972783539	0.349102721	0.131390856
let-7g-5p	mir-125b-2-5p	0.00815382	0.972783539	0.349102721	0.131390856
mir-199a-1-5p	mir-25-3p	-0.485370573	0.030054325	-0.709504537	0.000459162
mir-25-3p	mir-199a-2-5p	-0.485370573	0.030054325	-0.709504537	0.000459162
mir-143-3p	mir-15b-5p	-0.563492	0.009672434	-0.274688457	0.241172245
mir-106a-5p	mir-23a-3p	-0.263443631	0.261750228	-0.555105412	0.01106521
let-7d-5p	mir-30c-1-5p	0.82537209	7.47E-06	0.673756024	0.0011262
let-7d-5p	mir-30c-2-5p	0.82537209	7.47E-06	0.673756024	0.0011262
mir-128-1-3p	mir-15b-5p	0.357217246	0.122058302	0.622747309	0.003359651
mir-128-2-3p	mir-15b-5p	0.357217246	0.122058302	0.622747309	0.003359651
mir-30d-5p	mir-20b-5p	0.065202509	0.784769247	-0.282027355	0.228320091
mir-101-1-3p	mir-107	0.942783326	5.07E-10	0.886954539	1.89E-07
mir-107	mir-101-2-3p	0.942783326	5.07E-10	0.886954539	1.89E-07

mir-139-5p	copeptin	-0.087137087	0.71489264	-0.41562639	0.068375528
mir-27a-3p	let-7d-3p	-0.024206221	0.919314361	0.319159886	0.170184402
mir-7-3-5p	mir-15b-5p	0.544901994	0.012974385	0.250882971	0.286007388
mir-7-1-5p	mir-15b-5p	0.544901994	0.012974385	0.250882971	0.286007388
mir-7-2-5p	mir-15b-5p	0.544901994	0.012974385	0.250882971	0.286007388
mir-532-5p	mir-125b-1-5p	0.28717165	0.219582107	-0.059024884	0.80476456
mir-532-5p	mir-125b-2-5p	0.28717165	0.219582107	-0.059024884	0.80476456
mir-423-5p	mir-186-5p	0.795870276	2.71E-05	0.624714566	0.003232147
mir-221-3p	mir-20b-5p	-0.373413751	0.104863489	-0.633309831	0.00272121
mir-19b-2-3p	mir-122-5p	0.014593539	0.951307558	-0.327207824	0.159067785
mir-122-5p	mir-19b-1-3p	0.014593539	0.951307558	-0.327207824	0.159067785
mir-181b-2-5p	cpeptide	-0.056611918	0.812607215	0.289060175	0.21643012
mir-181b-1-5p	cpeptide	-0.056611918	0.812607215	0.289060175	0.21643012
mir-15a-5p	mir-23a-3p	-0.521169854	0.0184522	-0.731487462	0.000247298
mir-15a-5p	mir-150-5p	-0.085290114	0.720701441	0.261927015	0.264607866
mir-16-2-5p	mir-199a-2-3p	-0.274430426	0.24163241	-0.561663209	0.009963278
mir-16-2-5p	mir-199a-1-3p	-0.274430426	0.24163241	-0.561663209	0.009963278
mir-16-2-5p	mir-199b-3p	-0.274430426	0.24163241	-0.561663209	0.009963278
mir-199a-2-3p	mir-16-1-5p	-0.274430426	0.24163241	-0.561663209	0.009963278
mir-16-1-5p	mir-199a-1-3p	-0.274430426	0.24163241	-0.561663209	0.009963278
mir-16-1-5p	mir-199b-3p	-0.274430426	0.24163241	-0.561663209	0.009963278
mir-222-3p	norepi	0.040731053	0.864620072	-0.302754472	0.194464781
mir-92a-2-3p	mir-23a-3p	-0.628806234	0.002979797	-0.797873341	2.50E-05
mir-23a-3p	mir-92a-1-3p	-0.628806234	0.002979797	-0.797873341	2.50E-05
mir-194-2-5p	mir-363-3p	0.8123159	1.36E-05	0.653013136	0.001798636
mir-194-1-5p	mir-363-3p	0.8123159	1.36E-05	0.653013136	0.001798636
mir-28-5p	cpeptide	0.083433271	0.726556169	-0.263250283	0.262113451
mir-26b-5p	mir-146a-5p	-0.298431242	0.201230745	-0.578860471	0.007490738
let-7i-5p	mir-148b-3p	0.55046508	0.011903054	0.749400194	0.000142797
mir-192-5p	mir-125a-5p	-0.585906749	0.006634969	-0.3085922	0.185573113
mir-361-5p	mir-224-5p	0.42855852	0.059388273	0.669838791	0.001233653
mir-423-3p	mir-224-5p	0.083068911	0.727706745	0.409764968	0.072769603
mir-148b-3p	mir-221-3p	0.083452359	0.726495909	0.410069591	0.07253617
mir-126-3p	mir-27b-3p	0.812375098	1.35E-05	0.902589555	5.24E-08
mir-144-3p	mir-20a-5p	0.892569319	1.22E-07	0.794158621	2.90E-05
let-7d-5p	mir-126-5p	0.439299384	0.052629501	0.676781489	0.001048708
mir-29a-3p	mir-320a	0.422630722	0.06338941	0.098785847	0.678615154
mir-20a-5p	mir-28-3p	-0.088607212	0.710279784	-0.414121998	0.069483801
mir-181a-2-5p	mir-143-3p	-0.230259733	0.328739649	0.116642536	0.624324628
mir-143-3p	mir-181a-1-5p	-0.230259733	0.328739649	0.116642536	0.624324628
mir-24-2-3p	mir-221-3p	0.888392298	1.69E-07	0.943152454	4.79E-10
mir-221-3p	mir-24-1-3p	0.888392298	1.69E-07	0.943152454	4.79E-10
mir-16-2-5p	mir-629-5p	0.906395414	3.71E-08	0.819523077	9.82E-06
mir-629-5p	mir-16-1-5p	0.906395414	3.71E-08	0.819523077	9.82E-06
mir-150-5p	mir-126-5p	0.079532398	0.738903146	-0.265330213	0.258222795
mir-182-5p	mir-125a-5p	-0.541452232	0.013676922	-0.743231722	0.000173379
mir-106b-3p	mir-27a-3p	0.537444446	0.014531263	0.244208992	0.299441946
mir-361-5p	mir-10b-5p	0.195333008	0.40919981	-0.15220894	0.521770319
mir-584-5p	mir-106b-3p	-0.346178471	0.134874651	-0.61214753	0.004120705
mir-451a	mir-191-5p	-0.210609655	0.372776345	-0.511709507	0.021093426
mir-27b-3p	mir-1307-3p	0.521571753	0.018346143	0.730434212	0.000255077
mir-584-5p	mir-30a-5p	-0.291800203	0.2119101	0.050316495	0.83314795
mir-144-3p	mir-128-1-3p	0.137531757	0.563118068	-0.20932688	0.375762042
mir-144-3p	mir-128-2-3p	0.137531757	0.563118068	-0.20932688	0.375762042
mir-16-2-5p	mir-584-5p	-0.31560492	0.175260149	-0.589970156	0.006179089
mir-584-5p	mir-16-1-5p	-0.31560492	0.175260149	-0.589970156	0.006179089
mir-106a-5p	let-7d-5p	-0.373527615	0.104749196	-0.631033515	0.002849469
let-7a-3-5p	mir-20b-5p	-0.243194807	0.301516569	-0.536099032	0.014827502
let-7a-2-5p	mir-20b-5p	-0.243194807	0.301516569	-0.536099032	0.014827502
let-7a-1-5p	mir-20b-5p	-0.243194807	0.301516569	-0.536099032	0.014827502
mir-194-2-5p	mir-107	0.820884547	9.22E-06	0.669196439	0.001252071
mir-194-1-5p	mir-107	0.820884547	9.22E-06	0.669196439	0.001252071
mir-224-5p	mir-363-3p	0.091367959	0.701643576	-0.252925998	0.281970492
mir-92a-2-3p	mir-181b-2-5p	0.362755303	0.11596705	0.029996105	0.900098714

mir-92a-2-3p	mir-181b-1-5p	0.362755303	0.11596705	0.029996105	0.900098714
mir-181b-2-5p	mir-92a-1-3p	0.362755303	0.11596705	0.029996105	0.900098714
mir-181b-1-5p	mir-92a-1-3p	0.362755303	0.11596705	0.029996105	0.900098714
mir-27b-3p	mir-16-2-5p	-0.265635815	0.257654244	-0.552599472	0.011511522
mir-27b-3p	mir-16-1-5p	-0.265635815	0.257654244	-0.552599472	0.011511522
mir-29a-3p	mir-375	0.639076034	0.002417666	0.385929943	0.092833366
mir-146a-5p	mir-10a-5p	0.226504177	0.33690749	0.522721403	0.018045449
mir-185-5p	mir-363-3p	0.892047532	1.27E-07	0.794049543	2.92E-05
let-7i-5p	let-7d-3p	0.401210923	0.079556416	0.649539048	0.001938871
mir-139-5p	mir-30e-5p	0.003068854	0.98975498	0.338460971	0.144380859
mir-29a-3p	mir-486-2-5p	0.407927767	0.074189375	0.083879989	0.725146296
mir-192-5p	mir-30c-1-5p	-0.424807196	0.061897395	-0.104124314	0.662208895
mir-192-5p	mir-30c-2-5p	-0.424807196	0.061897395	-0.104124314	0.662208895
mir-146a-5p	mir-3615	-0.517103242	0.019553121	-0.726515681	0.00028588
mir-128-1-3p	mir-99a-5p	0.213473516	0.366159282	0.512175534	0.02095665
mir-128-2-3p	mir-99a-5p	0.213473516	0.366159282	0.512175534	0.02095665
let-7f-2-5p	mir-146b-5p	0.880331174	3.07E-07	0.938592393	9.43E-10
mir-146b-5p	let-7f-1-5p	0.880331174	3.07E-07	0.938592393	9.43E-10
mir-29a-3p	mir-150-5p	0.565301054	0.00939156	0.283743574	0.225380196
mir-101-1-3p	mir-20b-5p	0.928747517	3.46E-09	0.861888362	1.04E-06
mir-101-2-3p	mir-20b-5p	0.928747517	3.46E-09	0.861888362	1.04E-06
mir-21-5p	mir-10b-5p	0.190683618	0.420656014	-0.154146561	0.516420691
mir-29a-3p	mir-106a-5p	0.437617823	0.05364691	0.120531035	0.612733653
mir-1307-3p	norepi	-0.007256433	0.975778014	0.328154951	0.157793378
mir-361-5p	mir-15b-5p	-0.07352094	0.758050595	0.267627976	0.253967429
mir-7-3-5p	mir-27a-3p	0.639023549	0.002420296	0.387410385	0.091480428
mir-7-1-5p	mir-27a-3p	0.639023549	0.002420296	0.387410385	0.091480428
mir-27a-3p	mir-7-2-5p	0.639023549	0.002420296	0.387410385	0.091480428
mir-125a-5p	mir-629-5p	-0.650964563	0.001880261	-0.809198192	1.56E-05
mir-16-2-5p	mir-185-5p	0.948252466	2.09E-10	0.898911837	7.22E-08
mir-16-1-5p	mir-185-5p	0.948252466	2.09E-10	0.898911837	7.22E-08
mir-10b-5p	mir-451a	0.060786606	0.799049864	0.386900788	0.091944491
mir-10b-5p	mir-378a-3p	0.004266936	0.985755653	0.33768579	0.145360956
mir-543	mir-28-3p	0.302990373	0.194100036	0.578283783	0.007564595
mir-423-3p	mir-10a-5p	-0.203088161	0.390474005	0.14018766	0.555530347
mir-16-2-5p	mir-194-2-5p	0.856210495	1.46E-06	0.731504684	0.000247173
mir-16-2-5p	mir-194-1-5p	0.856210495	1.46E-06	0.731504684	0.000247173
mir-194-2-5p	mir-16-1-5p	0.856210495	1.46E-06	0.731504684	0.000247173
mir-194-1-5p	mir-16-1-5p	0.856210495	1.46E-06	0.731504684	0.000247173
mir-361-5p	mir-191-5p	0.70431954	0.000527109	0.840437114	3.51E-06
mir-543	norepi	-0.04558717	0.84864833	0.292412715	0.210908273
mir-10b-5p	mir-186-5p	-0.194716909	0.410708058	0.148412821	0.532326013
mir-98-5p	mir-374b-5p	0.853653612	1.70E-06	0.727196516	0.000280311
mir-126-5p	mir-93-5p	0.356169457	0.123235961	0.025961369	0.913484292
mir-103a-2-3p	let-7d-3p	0.610341128	0.00426362	0.784120144	4.28E-05
mir-103a-1-3p	let-7d-3p	0.610341128	0.00426362	0.784120144	4.28E-05
let-7f-2-5p	mir-10a-5p	0.361428216	0.117406393	0.619905645	0.003551179
let-7f-1-5p	mir-10a-5p	0.361428216	0.117406393	0.619905645	0.003551179
mir-125a-5p	cpeptide	0.010951038	0.963451973	-0.323280869	0.164427562
mir-24-2-3p	mir-150-5p	0.375012338	0.103267134	0.048083837	0.840458463
mir-150-5p	mir-24-1-3p	0.375012338	0.103267134	0.048083837	0.840458463
mir-98-5p	mir-107	0.007178353	0.976038573	-0.326370864	0.160199862
mir-25-3p	mir-374b-5p	0.037499144	0.875278273	-0.298991343	0.200345446
mir-10b-5p	mir-320a	-0.125095922	0.599238766	0.216107981	0.360131823
mir-192-5p	mir-99b-5p	-0.511451156	0.021169555	-0.216031554	0.360305875
mir-101-1-3p	let-7b-5p	0.924434542	5.79E-09	0.85474339	1.59E-06
let-7b-5p	mir-101-2-3p	0.924434542	5.79E-09	0.85474339	1.59E-06
mir-146a-5p	let-7b-5p	-0.490784122	0.028004551	-0.707487852	0.000484647
mir-126-3p	norepi	0.168258642	0.478259482	0.473576474	0.034927127
mir-3615	mir-125a-5p	-0.721707655	0.000327963	-0.512990954	0.020719027
mir-122-5p	mir-378a-3p	0.510027351	0.021593042	0.214990449	0.362681666
mir-19b-2-3p	mir-146a-5p	-0.179345072	0.449302679	-0.482063538	0.031363238
mir-146a-5p	mir-19b-1-3p	-0.179345072	0.449302679	-0.482063538	0.031363238
let-7i-5p	mir-199a-1-5p	0.050306356	0.833181118	0.375240783	0.103040457

let-7i-5p	mir-199a-2-5p	0.050306356	0.833181118	0.375240783	0.103040457
mir-92a-2-3p	copeptin	-0.121259381	0.610572279	0.2186761	0.354311328
mir-92a-1-3p	copeptin	-0.121259381	0.610572279	0.2186761	0.354311328
mir-26a-2-5p	mir-374b-5p	0.754828394	0.000119833	0.868799903	6.73E-07
mir-374b-5p	mir-26a-1-5p	0.754828394	0.000119833	0.868799903	6.73E-07
mir-21-5p	mir-194-2-5p	-0.505464276	0.022995781	-0.209546211	0.375250588
mir-21-5p	mir-194-1-5p	-0.505464276	0.022995781	-0.209546211	0.375250588
mir-29a-3p	mir-92a-2-3p	0.082216679	0.730400103	-0.255685178	0.276574871
mir-29a-3p	mir-92a-1-3p	0.082216679	0.730400103	-0.255685178	0.276574871
mir-107	mir-106b-3p	0.901870292	5.59E-08	0.813846647	1.27E-05
let-7i-5p	mir-7-3-5p	0.41812726	0.066562594	0.101442551	0.670432575
let-7i-5p	mir-7-1-5p	0.41812726	0.066562594	0.101442551	0.670432575
let-7i-5p	mir-7-2-5p	0.41812726	0.066562594	0.101442551	0.670432575
mir-222-3p	mir-99a-5p	0.376094605	0.102196443	0.051878394	0.828041529
mir-92a-2-3p	mir-484	0.319145492	0.170204749	-0.01279725	0.957295411
mir-484	mir-92a-1-3p	0.319145492	0.170204749	-0.01279725	0.957295411
mir-126-3p	mir-20b-5p	-0.19933765	0.399469901	-0.497055614	0.025769208
mir-532-5p	mir-99b-5p	-0.789245308	3.52E-05	-0.620669216	0.003498846
mir-224-5p	copeptin	0.216061637	0.360237358	-0.123134512	0.60502209
mir-21-5p	mir-28-5p	0.711124599	0.000439523	0.843368609	3.01E-06
mir-122-5p	mir-99a-5p	0.910163847	2.60E-08	0.829108873	6.24E-06
mir-486-2-5p	mir-23a-3p	-0.292194548	0.211264748	-0.567629238	0.009039889
let-7f-2-5p	mir-194-2-5p	-0.540417614	0.013893492	-0.255879367	0.27619757
let-7f-2-5p	mir-194-1-5p	-0.540417614	0.013893492	-0.255879367	0.27619757
mir-194-2-5p	let-7f-1-5p	-0.540417614	0.013893492	-0.255879367	0.27619757
mir-194-1-5p	let-7f-1-5p	-0.540417614	0.013893492	-0.255879367	0.27619757
mir-30c-1-5p	mir-107	-0.377627888	0.100693402	-0.054556396	0.819301704
mir-107	mir-30c-2-5p	-0.377627888	0.100693402	-0.054556396	0.819301704
mir-103a-2-3p	mir-484	0.654663398	0.00173506	0.414341221	0.069321468
mir-103a-1-3p	mir-484	0.654663398	0.00173506	0.414341221	0.069321468
mir-140-5p	mir-486-2-5p	0.487304904	0.029308831	0.188325245	0.426532165
mir-181a-2-5p	mir-25-3p	0.150595042	0.526245959	-0.187575263	0.428409947
mir-25-3p	mir-181a-1-5p	0.150595042	0.526245959	-0.187575263	0.428409947
mir-26b-5p	mir-451a	0.883560902	2.43E-07	0.939447655	8.33E-10
mir-1307-3p	copeptin	-0.366451803	0.112024713	-0.042852372	0.857636479
mir-125b-1-5p	mir-28-3p	-0.253035996	0.28175415	0.082475608	0.729581473
mir-28-3p	mir-125b-2-5p	-0.253035996	0.28175415	0.082475608	0.729581473
mir-181a-2-5p	mir-363-3p	0.065047522	0.785269369	-0.269228564	0.251029712
mir-363-3p	mir-181a-1-5p	0.065047522	0.785269369	-0.269228564	0.251029712
mir-194-2-5p	mir-24-2-3p	-0.333902279	0.150211431	-0.006085328	0.979686319
mir-194-2-5p	mir-24-1-3p	-0.333902279	0.150211431	-0.006085328	0.979686319
mir-24-2-3p	mir-194-1-5p	-0.333902279	0.150211431	-0.006085328	0.979686319
mir-194-1-5p	mir-24-1-3p	-0.333902279	0.150211431	-0.006085328	0.979686319
let-7a-3-5p	mir-15a-5p	-0.551954457	0.011628727	-0.745238498	0.000162868
let-7a-2-5p	mir-15a-5p	-0.551954457	0.011628727	-0.745238498	0.000162868
let-7a-1-5p	mir-15a-5p	-0.551954457	0.011628727	-0.745238498	0.000162868
mir-584-5p	mir-224-5p	0.063630929	0.789844253	0.383918988	0.094694545
mir-144-3p	let-7d-3p	-0.285096424	0.223080241	-0.560605866	0.01013465
mir-144-3p	mir-30e-5p	0.816760375	1.11E-05	0.667691889	0.001296117
mir-26b-5p	mir-99a-5p	0.176453559	0.45676608	-0.160031551	0.500333713
mir-23a-3p	mir-93-5p	-0.143007672	0.547524108	-0.449058514	0.047012681
mir-148b-3p	mir-186-5p	0.592982984	0.005857955	0.330228319	0.155028262
mir-3615	mir-20b-5p	0.807210816	1.69E-05	0.897281487	8.28E-08
mir-30a-5p	mir-28-5p	0.066402199	0.780900772	0.384676071	0.093990672
mir-199a-1-5p	mir-629-5p	-0.460810594	0.040873182	-0.684390994	0.000873414
mir-629-5p	mir-199a-2-5p	-0.460810594	0.040873182	-0.684390994	0.000873414
mir-584-5p	mir-30c-1-5p	0.731447604	0.000247589	0.532004001	0.015759164
mir-584-5p	mir-30c-2-5p	0.731447604	0.000247589	0.532004001	0.015759164
mir-199a-1-5p	mir-451a	-0.448519357	0.047310428	-0.675869397	0.001071581
mir-451a	mir-199a-2-5p	-0.448519357	0.047310428	-0.675869397	0.001071581
mir-30c-1-5p	cpeptide	0.042132937	0.860003818	-0.287741408	0.218628016
mir-30c-2-5p	cpeptide	0.042132937	0.860003818	-0.287741408	0.218628016
mir-146b-5p	mir-425-5p	-0.400504342	0.080137317	-0.086075688	0.718228958
mir-192-5p	mir-186-5p	0.809320878	1.55E-05	0.656899549	0.001651928

let-7b-5p	mir-185-5p	0.897826687	7.91E-08	0.808741933	1.59E-05
mir-24-2-3p	mir-151a-5p	0.243144537	0.301619629	0.526422045	0.017104185
mir-151a-5p	mir-24-1-3p	0.243144537	0.301619629	0.526422045	0.017104185
mir-99a-5p	mir-15b-5p	-0.185964742	0.432457133	0.14778284	0.534087274
let-7b-5p	mir-128-1-3p	-0.027912922	0.907006831	-0.349520576	0.130898299
let-7b-5p	mir-128-2-3p	-0.027912922	0.907006831	-0.349520576	0.130898299
mir-140-5p	mir-15a-5p	0.279319781	0.2330087	-0.049941194	0.834375912
mir-3615	mir-126-5p	-6.13E-05	0.999795354	-0.324704149	0.162470815
mir-16-2-5p	mir-486-2-5p	0.961538517	1.52E-11	0.925990001	4.83E-09
mir-486-2-5p	mir-16-1-5p	0.961538517	1.52E-11	0.925990001	4.83E-09
mir-10b-5p	mir-151a-3p	0.17356472	0.464285642	-0.15969016	0.501260238
mir-181a-2-5p	mir-106a-5p	0.028166136	0.906166784	-0.298811154	0.200629971
mir-106a-5p	mir-181a-1-5p	0.028166136	0.906166784	-0.298811154	0.200629971
mir-103a-2-3p	let-7d-5p	0.639199052	0.002411512	0.798029195	2.49E-05
mir-103a-1-3p	let-7d-5p	0.639199052	0.002411512	0.798029195	2.49E-05
mir-26b-5p	mir-423-5p	0.756894042	0.000111969	0.573536874	0.008195451
mir-30a-5p	mir-15b-5p	-0.317876806	0.172004624	0.0068255	0.977216102
let-7f-2-5p	mir-375	-0.077675573	0.744802226	0.252650898	0.282512005
mir-375	let-7f-1-5p	-0.077675573	0.744802226	0.252650898	0.282512005
mir-22-3p	mir-320a	0.803459984	1.99E-05	0.648293633	0.001991321
mir-148a-3p	mir-378a-3p	0.587948118	0.006402616	0.32637731	0.160191122
mir-222-3p	mir-101-1-3p	0.794429783	2.87E-05	0.889405291	1.56E-07
mir-222-3p	mir-101-2-3p	0.794429783	2.87E-05	0.889405291	1.56E-07
mir-744-5p	mir-126-5p	0.264661846	0.259469011	0.541879132	0.013588359
mir-10b-5p	mir-185-5p	-0.120600528	0.6125273	0.211048561	0.37175787
mir-7-3-5p	mir-186-5p	0.88949236	1.55E-07	0.79470652	2.84E-05
mir-7-1-5p	mir-186-5p	0.88949236	1.55E-07	0.79470652	2.84E-05
mir-186-5p	mir-7-2-5p	0.88949236	1.55E-07	0.79470652	2.84E-05
mir-140-5p	let-7g-5p	0.710107384	0.000451769	0.840348459	3.53E-06
mir-101-1-3p	mir-194-2-5p	0.837443405	4.11E-06	0.705227497	0.00051463
mir-101-1-3p	mir-194-1-5p	0.837443405	4.11E-06	0.705227497	0.00051463
mir-194-2-5p	mir-101-2-3p	0.837443405	4.11E-06	0.705227497	0.00051463
mir-194-1-5p	mir-101-2-3p	0.837443405	4.11E-06	0.705227497	0.00051463
mir-30d-5p	mir-151a-3p	0.73494487	0.000223171	0.854960173	1.57E-06
mir-29a-3p	mir-101-1-3p	0.545186108	0.012917849	0.269992384	0.249635469
mir-29a-3p	mir-101-2-3p	0.545186108	0.012917849	0.269992384	0.249635469
mir-222-3p	mir-7-3-5p	0.742418437	0.000177802	0.859246484	1.22E-06
mir-222-3p	mir-7-1-5p	0.742418437	0.000177802	0.859246484	1.22E-06
mir-222-3p	mir-7-2-5p	0.742418437	0.000177802	0.859246484	1.22E-06
mir-222-3p	mir-27b-3p	-0.107835898	0.650888553	-0.415480188	0.068482648
mir-126-3p	mir-15a-5p	-0.582778635	0.0070044	-0.761853293	9.49E-05
mir-26a-2-5p	mir-24-2-3p	0.667948993	0.0012885	0.814718931	1.22E-05
mir-26a-2-5p	mir-24-1-3p	0.667948993	0.0012885	0.814718931	1.22E-05
mir-24-2-3p	mir-26a-1-5p	0.667948993	0.0012885	0.814718931	1.22E-05
mir-24-1-3p	mir-26a-1-5p	0.667948993	0.0012885	0.814718931	1.22E-05
mir-7-3-5p	mir-125b-1-5p	0.387304406	0.091576795	0.074700975	0.754280894
mir-7-3-5p	mir-125b-2-5p	0.387304406	0.091576795	0.074700975	0.754280894
mir-7-1-5p	mir-125b-1-5p	0.387304406	0.091576795	0.074700975	0.754280894
mir-7-1-5p	mir-125b-2-5p	0.387304406	0.091576795	0.074700975	0.754280894
mir-125b-1-5p	mir-7-2-5p	0.387304406	0.091576795	0.074700975	0.754280894
mir-7-2-5p	mir-125b-2-5p	0.387304406	0.091576795	0.074700975	0.754280894
let-7b-5p	mir-126-5p	0.045233774	0.849808811	-0.280645014	0.230706109
let-7i-5p	mir-128-1-3p	0.673403934	0.001135524	0.817913515	1.06E-05
let-7i-5p	mir-128-2-3p	0.673403934	0.001135524	0.817913515	1.06E-05
mir-106b-3p	mir-28-5p	-0.337486781	0.14561332	-0.594553614	0.005696065
mir-92a-2-3p	mir-423-5p	0.266648097	0.255776638	-0.060021294	0.801531203
mir-423-5p	mir-92a-1-3p	0.266648097	0.255776638	-0.060021294	0.801531203
mir-192-5p	mir-125b-1-5p	0.502482311	0.023950828	0.216361091	0.35955574
mir-192-5p	mir-125b-2-5p	0.502482311	0.023950828	0.216361091	0.35955574
mir-140-5p	mir-378a-3p	0.19202569	0.417331592	0.483101546	0.030947683
mir-92a-2-3p	mir-15b-5p	0.069910294	0.769617409	-0.256655316	0.27469314
mir-92a-1-3p	mir-15b-5p	0.069910294	0.769617409	-0.256655316	0.27469314
mir-107	mir-30e-5p	0.871509954	5.64E-07	0.764542465	8.66E-05
mir-486-2-5p	mir-28-5p	-0.178983916	0.450231403	-0.472388533	0.035450155

mir-7-3-5p	mir-28-5p	-0.151435448	0.523913122	-0.450090657	0.046446701
mir-7-1-5p	mir-28-5p	-0.151435448	0.523913122	-0.450090657	0.046446701
mir-7-2-5p	mir-28-5p	-0.151435448	0.523913122	-0.450090657	0.046446701
mir-629-5p	mir-486-2-5p	0.905309988	4.10E-08	0.82386845	8.02E-06
mir-375	mir-28-3p	0.241991196	0.303989999	-0.085054168	0.72144457
mir-107	mir-15b-5p	0.670075614	0.00122692	0.44522872	0.049159166
mir-98-5p	mir-185-5p	0.089142611	0.708602243	-0.238090644	0.312089956
mir-146a-5p	mir-101-1-3p	-0.314760721	0.176480484	-0.576833998	0.007752904
mir-146a-5p	mir-101-2-3p	-0.314760721	0.176480484	-0.576833998	0.007752904
mir-182-5p	mir-20b-5p	0.749408319	0.00014276	0.862584257	9.97E-07
mir-150-5p	mir-99a-5p	0.747138608	0.000153424	0.561381717	0.01000867
mir-10b-5p	mir-7-3-5p	-0.036526841	0.878488869	0.286471804	0.220757766
mir-10b-5p	mir-7-1-5p	-0.036526841	0.878488869	0.286471804	0.220757766
mir-10b-5p	mir-7-2-5p	-0.036526841	0.878488869	0.286471804	0.220757766
mir-340-5p	let-7b-5p	-0.326585725	0.15990871	-0.585127698	0.006725446
mir-20a-5p	mir-363-3p	0.89718503	8.35E-08	0.809811135	1.52E-05
mir-106a-5p	mir-1307-3p	-0.618404945	0.003655926	-0.783160967	4.44E-05
mir-486-2-5p	cpeptide	-0.192298764	0.416656901	0.135439871	0.569126381
mir-26a-2-5p	mir-224-5p	0.409275031	0.07314622	0.644236838	0.002170474
mir-224-5p	mir-26a-1-5p	0.409275031	0.07314622	0.644236838	0.002170474
mir-181b-2-5p	mir-584-5p	-0.138303364	0.560908941	0.189054721	0.42470994
mir-584-5p	mir-181b-1-5p	-0.138303364	0.560908941	0.189054721	0.42470994
mir-106a-5p	mir-27a-3p	0.598580393	0.005297754	0.345584541	0.135590118
let-7d-5p	mir-148b-3p	0.271981276	0.246028268	0.543683884	0.013219044
mir-29a-3p	mir-125a-5p	-0.108351278	0.649322378	0.21798028	0.355882974
mir-150-5p	mir-23a-3p	0.273486739	0.243320165	-0.049411998	0.836108038
mir-103a-2-3p	mir-99b-5p	0.34653684	0.134444241	0.598977434	0.005259756
mir-103a-1-3p	mir-99b-5p	0.34653684	0.134444241	0.598977434	0.005259756
mir-17-5p	mir-28-3p	0.071147645	0.765648103	-0.253067232	0.281692735
mir-107	mir-484	0.789251335	3.52E-05	0.628986117	0.002969091
mir-186-5p	cpeptide	-0.209595076	0.375136695	0.116017594	0.626195521
mir-22-3p	mir-17-5p	0.936650403	1.24E-09	0.88121766	2.88E-07
mir-22-3p	mir-26a-2-5p	-0.208328678	0.378094692	0.116597766	0.624458583
mir-22-3p	mir-26a-1-5p	-0.208328678	0.378094692	0.116597766	0.624458583
mir-423-5p	mir-93-5p	0.824289646	7.86E-06	0.68661155	0.000827195
mir-139-5p	norepi	0.267940239	0.253392593	0.539139782	0.014164777
mir-181a-2-5p	mir-451a	0.036219447	0.879504284	-0.283727109	0.225408282
mir-451a	mir-181a-1-5p	0.036219447	0.879504284	-0.283727109	0.225408282
mir-126-3p	mir-363-3p	-0.221707794	0.347510543	-0.502788445	0.023851359
mir-15a-5p	let-7d-3p	-0.629185774	0.002957245	-0.788568181	3.62E-05
mir-20a-5p	cpeptide	-0.193946473	0.412598371	0.13002031	0.584821004
mir-21-5p	mir-425-5p	-0.219175319	0.353186218	0.1038338	0.663097984
mir-151a-5p	mir-378a-3p	-0.527782525	0.016768196	-0.254550885	0.278785159
let-7f-2-5p	mir-486-2-5p	-0.342485783	0.139366426	-0.593843791	0.005768767
mir-486-2-5p	let-7f-1-5p	-0.342485783	0.139366426	-0.593843791	0.005768767
mir-22-3p	mir-151a-5p	-0.012527239	0.958195678	0.304103722	0.192384788
mir-30a-5p	mir-10a-5p	0.499708931	0.024866992	0.704086081	0.000530359
mir-144-3p	mir-126-5p	0.24454456	0.298757428	-0.076684788	0.747955552
mir-224-5p	mir-629-5p	-0.079893856	0.737756424	-0.385228668	0.093479339
mir-26a-2-5p	mir-143-3p	0.064342072	0.787546779	0.371815919	0.106476893
mir-143-3p	mir-26a-1-5p	0.064342072	0.787546779	0.371815919	0.106476893
mir-106a-5p	mir-15b-5p	0.473569291	0.034930271	0.186569842	0.430934171
mir-128-1-3p	mir-30c-1-5p	0.57938676	0.007423851	0.756161596	0.000114705
mir-128-1-3p	mir-30c-2-5p	0.57938676	0.007423851	0.756161596	0.000114705
mir-30c-1-5p	mir-128-2-3p	0.57938676	0.007423851	0.756161596	0.000114705
mir-128-2-3p	mir-30c-2-5p	0.57938676	0.007423851	0.756161596	0.000114705
mir-146b-5p	mir-144-3p	-0.521436811	0.018381698	-0.718181019	0.000362089
mir-375	mir-185-5p	-0.140984502	0.553262764	0.181270163	0.444369043
mir-1307-3p	mir-30d-5p	0.107623613	0.651534073	0.408012462	0.074123472
let-7f-2-5p	mir-28-5p	0.710697279	0.000444632	0.8377002	4.05E-06
let-7f-1-5p	mir-28-5p	0.710697279	0.000444632	0.8377002	4.05E-06
mir-320a	mir-126-5p	0.320530099	0.168255178	0.007852508	0.973788946
mir-532-5p	mir-151a-3p	-0.307033796	0.187919398	-0.566010208	0.009283284
mir-361-5p	mir-423-3p	0.592763015	0.005880927	0.764158577	8.77E-05

mir-27b-3p	mir-144-3p	-0.258537233	0.271065711	-0.52890514	0.01649495
mir-361-5p	mir-126-5p	0.421416867	0.06423323	0.648829487	0.00196861
mir-532-5p	mir-28-3p	-0.446877504	0.048226057	-0.666229673	0.001340167
mir-181a-2-5p	let-7b-5p	0.007730433	0.974196289	-0.305115133	0.190835437
let-7b-5p	mir-181a-1-5p	0.007730433	0.974196289	-0.305115133	0.190835437
mir-126-3p	mir-15b-5p	0.17211257	0.46808917	0.459449527	0.041550627
mir-107	mir-126-5p	0.33629439	0.147131821	0.027174607	0.90945675
mir-3615	mir-99b-5p	-0.652127212	0.001833563	-0.427065904	0.060377224
mir-3615	mir-423-3p	-0.532353107	0.015677952	-0.724031576	0.000307007
mir-224-5p	let-7g-5p	-0.016969729	0.943390541	0.296484904	0.204327385
mir-150-5p	mir-28-5p	0.237917766	0.312451936	-0.079861215	0.737859953
mir-744-5p	mir-20b-5p	-0.481782281	0.031476586	-0.689965697	0.000761309
mir-25-3p	mir-106b-3p	0.955224468	5.84E-11	0.916353478	1.40E-08
mir-29a-3p	let-7d-3p	0.001987386	0.993365239	0.313537446	0.178259037
mir-223-3p	mir-30d-5p	0.612517558	0.004091925	0.776035995	5.78E-05
mir-7-3-5p	mir-374b-5p	0.048188679	0.840114887	-0.267487587	0.254226137
mir-7-1-5p	mir-374b-5p	0.048188679	0.840114887	-0.267487587	0.254226137
mir-374b-5p	mir-7-2-5p	0.048188679	0.840114887	-0.267487587	0.254226137
let-7a-3-5p	mir-28-5p	0.75789051	0.000108336	0.865104564	8.52E-07
let-7a-2-5p	mir-28-5p	0.75789051	0.000108336	0.865104564	8.52E-07
let-7a-1-5p	mir-28-5p	0.75789051	0.000108336	0.865104564	8.52E-07
let-7d-3p	mir-20b-5p	-0.272494155	0.245103517	-0.538152817	0.014377215
mir-22-3p	mir-106a-5p	0.730082454	0.000257721	0.541990621	0.013565306
let-7b-5p	mir-375	-0.203835013	0.38869616	0.114602959	0.630438636
mir-584-5p	mir-25-3p	-0.284514476	0.224067712	-0.54723271	0.012516447
mir-194-2-5p	mir-186-5p	0.771673776	6.75E-05	0.606189396	0.004607678
mir-194-1-5p	mir-186-5p	0.771673776	6.75E-05	0.606189396	0.004607678
mir-181a-2-5p	mir-148b-3p	0.46126204	0.040650381	0.675284069	0.00108648
mir-148b-3p	mir-181a-1-5p	0.46126204	0.040650381	0.675284069	0.00108648
mir-3615	mir-199a-2-3p	-0.474475855	0.034535162	-0.684305317	0.000875241
mir-3615	mir-199a-1-3p	-0.474475855	0.034535162	-0.684305317	0.000875241
mir-3615	mir-199b-3p	-0.474475855	0.034535162	-0.684305317	0.000875241
mir-126-3p	mir-425-5p	-0.055952511	0.814753451	0.259199901	0.269795578
mir-486-2-5p	mir-375	-0.091112654	0.702440767	0.225740276	0.338583333
mir-1307-3p	mir-139-5p	0.357417671	0.121833954	0.601138446	0.005056856
mir-106a-5p	mir-28-5p	-0.209782479	0.37470008	-0.488237918	0.028954477
mir-224-5p	mir-23a-3p	0.470113281	0.036468919	0.680883787	0.000950852
mir-10a-5p	copeptin	0.037756213	0.874429727	-0.275249062	0.240174419
mir-146a-5p	mir-148b-3p	0.226677946	0.336526957	0.501037097	0.024424848
mir-378a-3p	mir-30a-5p	0.102887325	0.665997589	0.39966552	0.080831016
mir-3615	mir-125b-1-5p	0.408231686	0.073953093	0.113067412	0.635057058
mir-3615	mir-125b-2-5p	0.408231686	0.073953093	0.113067412	0.635057058
let-7i-5p	mir-101-1-3p	0.492148247	0.027505816	0.215539387	0.361427879
let-7i-5p	mir-101-2-3p	0.492148247	0.027505816	0.215539387	0.361427879
mir-340-5p	mir-744-5p	0.858943819	1.24E-06	0.748561791	0.000146661
mir-192-5p	mir-28-3p	-0.174970776	0.460617882	-0.459290779	0.041630203
mir-140-5p	mir-125b-1-5p	0.062447103	0.793672504	0.364230021	0.114382496
mir-140-5p	mir-125b-2-5p	0.062447103	0.793672504	0.364230021	0.114382496
mir-361-5p	mir-181b-2-5p	-0.279179502	0.233253303	0.032378601	0.89220653
mir-361-5p	mir-181b-1-5p	-0.279179502	0.233253303	0.032378601	0.89220653
mir-629-5p	mir-106b-3p	0.929935372	2.99E-09	0.871382584	5.68E-07
mir-101-1-3p	mir-125b-1-5p	0.337144805	0.146047699	0.031865469	0.893905515
mir-101-1-3p	mir-125b-2-5p	0.337144805	0.146047699	0.031865469	0.893905515
mir-125b-1-5p	mir-101-2-3p	0.337144805	0.146047699	0.031865469	0.893905515
mir-101-2-3p	mir-125b-2-5p	0.337144805	0.146047699	0.031865469	0.893905515
mir-222-3p	mir-103a-2-3p	0.268556032	0.252261426	-0.043594392	0.855196053
mir-222-3p	mir-103a-1-3p	0.268556032	0.252261426	-0.043594392	0.855196053
mir-106a-5p	mir-30e-5p	0.747180225	0.000153222	0.570092895	0.008679513
mir-374b-5p	mir-28-3p	0.363011872	0.115690246	0.603857641	0.004810759
mir-361-5p	mir-106b-3p	-0.602066821	0.004971694	-0.767881654	7.72E-05
mir-106b-3p	mir-93-5p	0.862784391	9.85E-07	0.755472964	0.00011733
mir-26a-2-5p	mir-151a-5p	0.59524672	0.005625804	0.763386846	9.01E-05
mir-151a-5p	mir-26a-1-5p	0.59524672	0.005625804	0.763386846	9.01E-05
mir-320a	mir-99a-5p	0.288924972	0.216654782	-0.021099437	0.929643697

mir-103a-2-3p	mir-186-5p	0.334041888	0.150030476	0.029161481	0.902865636
mir-103a-1-3p	mir-186-5p	0.334041888	0.150030476	0.029161481	0.902865636
mir-16-2-5p	mir-484	0.785041953	4.14E-05	0.629480613	0.002939823
mir-16-1-5p	mir-484	0.785041953	4.14E-05	0.629480613	0.002939823
mir-92a-2-3p	mir-378a-3p	0.42070673	0.064730797	0.13013897	0.584475414
mir-378a-3p	mir-92a-1-3p	0.42070673	0.064730797	0.13013897	0.584475414
mir-19b-2-3p	mir-222-3p	0.80326427	2.00E-05	0.890719995	1.41E-07
mir-222-3p	mir-19b-1-3p	0.80326427	2.00E-05	0.890719995	1.41E-07
mir-27a-3p	mir-20b-5p	0.581525805	0.007157008	0.333925789	0.150180949
mir-106a-5p	mir-423-3p	-0.456640568	0.042976149	-0.669910165	0.001231621
mir-543	mir-30a-5p	0.357230143	0.122043856	0.056215984	0.813895747
mir-106a-5p	mir-374b-5p	-0.074947345	0.753494521	-0.373335357	0.104942231
mir-26a-2-5p	mir-128-1-3p	0.738067414	0.000203139	0.851982295	1.87E-06
mir-26a-2-5p	mir-128-2-3p	0.738067414	0.000203139	0.851982295	1.87E-06
mir-128-1-3p	mir-26a-1-5p	0.738067414	0.000203139	0.851982295	1.87E-06
mir-128-2-3p	mir-26a-1-5p	0.738067414	0.000203139	0.851982295	1.87E-06
mir-199a-2-3p	mir-375	-0.073627813	0.757708964	0.238654655	0.31091076
mir-199a-1-3p	mir-375	-0.073627813	0.757708964	0.238654655	0.31091076
mir-375	mir-199b-3p	-0.073627813	0.757708964	0.238654655	0.31091076
mir-146a-5p	mir-143-3p	-0.065017412	0.785366537	0.246696358	0.294390721
mir-191-5p	let-7b-5p	-0.275378797	0.239943882	-0.536800702	0.014672405
mir-222-3p	mir-148a-3p	0.203607868	0.389236392	-0.10981057	0.644895464
mir-106a-5p	mir-21-5p	-0.38185237	0.096635543	-0.616067937	0.003824139
mir-26a-2-5p	mir-92a-2-3p	-0.638931679	0.002424905	-0.790438645	3.36E-05
mir-26a-2-5p	mir-92a-1-3p	-0.638931679	0.002424905	-0.790438645	3.36E-05
mir-92a-2-3p	mir-26a-1-5p	-0.638931679	0.002424905	-0.790438645	3.36E-05
mir-26a-1-5p	mir-92a-1-3p	-0.638931679	0.002424905	-0.790438645	3.36E-05
mir-17-5p	mir-106a-5p	0.750965135	0.00013582	0.577676575	0.007643003
mir-16-2-5p	mir-146a-5p	-0.359648793	0.119356371	-0.599661891	0.005194778
mir-146a-5p	mir-16-1-5p	-0.359648793	0.119356371	-0.599661891	0.005194778
mir-22-3p	mir-140-5p	0.690060915	0.000759505	0.8224031	8.59E-06
mir-181a-2-5p	mir-744-5p	0.516721033	0.019659227	0.710344618	0.000448887
mir-744-5p	mir-181a-1-5p	0.516721033	0.019659227	0.710344618	0.000448887
mir-423-3p	mir-15b-5p	0.055684129	0.815627346	0.355132224	0.124409715
mir-21-5p	let-7a-3-5p	0.930351788	2.84E-09	0.873022589	5.09E-07
mir-21-5p	let-7a-2-5p	0.930351788	2.84E-09	0.873022589	5.09E-07
mir-21-5p	let-7a-1-5p	0.930351788	2.84E-09	0.873022589	5.09E-07
mir-22-3p	mir-128-1-3p	0.084851837	0.722082017	0.380276365	0.098135164
mir-22-3p	mir-128-2-3p	0.084851837	0.722082017	0.380276365	0.098135164
let-7b-5p	let-7d-5p	-0.393578659	0.085998843	-0.623826724	0.003289183
mir-139-5p	mir-221-3p	0.75501251	0.000119113	0.861623269	1.06E-06
mir-192-5p	mir-194-2-5p	0.895769625	9.39E-08	0.943109121	4.82E-10
mir-192-5p	mir-194-1-5p	0.895769625	9.39E-08	0.943109121	4.82E-10
mir-30d-5p	mir-486-2-5p	0.039244416	0.869520024	-0.268995947	0.251455306
mir-181b-2-5p	mir-151a-5p	-0.082361112	0.729943431	0.228349692	0.332878961
mir-181b-1-5p	mir-151a-5p	-0.082361112	0.729943431	0.228349692	0.332878961
mir-29a-3p	norepi	-0.130905931	0.582243791	0.181047787	0.444937501
mir-15a-5p	mir-185-5p	0.7530992	0.000126777	0.582168262	0.007078413
mir-106a-5p	mir-221-3p	-0.459919915	0.041315527	-0.670327332	0.001219798
mir-92a-2-3p	let-7d-3p	-0.73929959	0.000195668	-0.851880997	1.88E-06
let-7d-3p	mir-92a-1-3p	-0.73929959	0.000195668	-0.851880997	1.88E-06
mir-126-3p	mir-24-2-3p	0.880916234	2.95E-07	0.934625407	1.63E-09
mir-126-3p	mir-24-1-3p	0.880916234	2.95E-07	0.934625407	1.63E-09
mir-223-3p	mir-15a-5p	-0.34590536	0.135203317	-0.5880663	0.006389372
mir-26a-2-5p	mir-378a-3p	-0.479388639	0.03245426	-0.20565489	0.384382941
mir-378a-3p	mir-26a-1-5p	-0.479388639	0.03245426	-0.20565489	0.384382941
mir-191-5p	mir-27a-3p	-0.002139371	0.992857859	0.301229384	0.196833933
mir-103a-2-3p	mir-20a-5p	0.412186008	0.070929781	0.124731129	0.600312643
mir-103a-1-3p	mir-20a-5p	0.412186008	0.070929781	0.124731129	0.600312643
mir-22-3p	norepi	0.134319324	0.572356274	-0.175738128	0.458622474
mir-584-5p	let-7b-5p	-0.330395625	0.154806612	-0.575677045	0.007905911
mir-93-5p	mir-15b-5p	0.70782194	0.000480344	0.515443666	0.020017175
mir-98-5p	mir-199a-2-3p	0.820754189	9.28E-06	0.899904978	6.63E-08
mir-98-5p	mir-199a-1-3p	0.820754189	9.28E-06	0.899904978	6.63E-08

mir-98-5p	mir-199b-3p	0.820754189	9.28E-06	0.899904978	6.63E-08
mir-140-5p	mir-425-5p	0.679648992	0.00097946	0.814713363	1.22E-05
mir-101-1-3p	mir-148a-3p	0.353157755	0.126666035	0.05673498	0.812206813
mir-148a-3p	mir-101-2-3p	0.353157755	0.126666035	0.05673498	0.812206813
mir-532-5p	mir-744-5p	-0.718563155	0.000358251	-0.838691565	3.85E-06
mir-185-5p	copeptin	0.078437718	0.742379238	-0.229257132	0.3309086
mir-10b-5p	let-7g-5p	-0.215354551	0.361849772	0.092931226	0.696768834
mir-101-1-3p	mir-185-5p	0.955497461	5.53E-11	0.918523669	1.11E-08
mir-101-2-3p	mir-185-5p	0.955497461	5.53E-11	0.918523669	1.11E-08
mir-181a-2-5p	mir-93-5p	0.250731946	0.286307212	-0.055505393	0.816209455
mir-93-5p	mir-181a-1-5p	0.250731946	0.286307212	-0.055505393	0.816209455
mir-92a-2-3p	mir-28-3p	-0.4787041	0.032738178	-0.682102424	0.000923314
mir-28-3p	mir-92a-1-3p	-0.4787041	0.032738178	-0.682102424	0.000923314
mir-21-5p	mir-224-5p	0.470842055	0.036140156	0.676600985	0.001053201
mir-146a-5p	let-7a-3-5p	0.881852262	2.75E-07	0.934863707	1.58E-09
mir-146a-5p	let-7a-2-5p	0.881852262	2.75E-07	0.934863707	1.58E-09
mir-146a-5p	let-7a-1-5p	0.881852262	2.75E-07	0.934863707	1.58E-09
mir-1307-3p	mir-451a	-0.671833303	0.001177912	-0.809530038	1.53E-05
mir-19b-2-3p	mir-486-2-5p	0.882259072	2.67E-07	0.791120581	3.27E-05
mir-19b-1-3p	mir-486-2-5p	0.882259072	2.67E-07	0.791120581	3.27E-05
mir-340-5p	mir-25-3p	-0.213891816	0.365198423	-0.484040921	0.030575344
mir-30c-1-5p	mir-543	0.535690415	0.014918428	0.279543065	0.232619706
mir-543	mir-30c-2-5p	0.535690415	0.014918428	0.279543065	0.232619706
mir-146a-5p	mir-532-5p	-0.598116032	0.005342481	-0.762058697	9.42E-05
mir-10b-5p	mir-30e-5p	-0.257042225	0.273944905	0.047847301	0.841233716
mir-146b-5p	mir-27a-3p	-0.141832854	0.550853165	0.16642727	0.483130735
mir-150-5p	mir-28-3p	-0.086364314	0.717321224	-0.377689408	0.100633434
mir-106a-5p	mir-7-3-5p	0.875719375	4.24E-07	0.93126471	2.53E-09
mir-106a-5p	mir-7-1-5p	0.875719375	4.24E-07	0.93126471	2.53E-09
mir-106a-5p	mir-7-2-5p	0.875719375	4.24E-07	0.93126471	2.53E-09
let-7b-5p	mir-20b-5p	0.882041464	2.72E-07	0.934842946	1.58E-09
mir-340-5p	mir-199a-2-3p	0.907922125	3.22E-08	0.835186859	4.61E-06
mir-340-5p	mir-199a-1-3p	0.907922125	3.22E-08	0.835186859	4.61E-06
mir-340-5p	mir-199b-3p	0.907922125	3.22E-08	0.835186859	4.61E-06
mir-361-5p	let-7a-3-5p	0.764219516	8.75E-05	0.865979958	8.06E-07
mir-361-5p	let-7a-2-5p	0.764219516	8.75E-05	0.865979958	8.06E-07
mir-361-5p	let-7a-1-5p	0.764219516	8.75E-05	0.865979958	8.06E-07
mir-181a-2-5p	let-7d-3p	0.624903598	0.00322011	0.779073575	5.17E-05
let-7d-3p	mir-181a-1-5p	0.624903598	0.00322011	0.779073575	5.17E-05
mir-378a-3p	norepi	-0.056647145	0.812492592	-0.351101724	0.129046325
mir-181b-2-5p	mir-144-3p	0.537737128	0.014467454	0.283065075	0.226539503
mir-144-3p	mir-181b-1-5p	0.537737128	0.014467454	0.283065075	0.226539503
mir-27a-3p	mir-363-3p	0.610344208	0.004263373	0.379558173	0.098824149
mir-192-5p	mir-150-5p	0.293917665	0.208460082	0.545979259	0.012761073
mir-28-3p	copeptin	-0.289535595	0.215641346	0.011706826	0.960931386
mir-451a	let-7d-5p	-0.378376158	0.099965763	-0.609335264	0.00434496
mir-181b-2-5p	mir-3615	0.307226967	0.187627486	0.007899616	0.973631754
mir-3615	mir-181b-1-5p	0.307226967	0.187627486	0.007899616	0.973631754
mir-107	mir-99b-5p	-0.580298106	0.007309184	-0.33940695	0.143191093
let-7g-5p	mir-28-3p	0.327262863	0.158993534	0.030413101	0.898716712
mir-148a-3p	copeptin	-0.177125327	0.4550265	0.129559138	0.586164958
mir-128-1-3p	mir-363-3p	0.060705279	0.79931346	-0.243409683	0.30107629
mir-128-2-3p	mir-363-3p	0.060705279	0.79931346	-0.243409683	0.30107629
mir-3615	mir-10b-5p	-0.009777067	0.967367868	0.290558061	0.213951381
mir-27b-3p	mir-93-5p	-0.327342756	0.158885795	-0.570855281	0.008570398
mir-125b-1-5p	mir-374b-5p	-0.08436693	0.723610445	0.220423844	0.350381417
mir-374b-5p	mir-125b-2-5p	-0.08436693	0.723610445	0.220423844	0.350381417
mir-19b-2-3p	mir-99a-5p	0.221877479	0.347132157	-0.082571658	0.729277873
mir-19b-1-3p	mir-99a-5p	0.221877479	0.347132157	-0.082571658	0.729277873
mir-181b-2-5p	mir-93-5p	0.545543834	0.012846949	0.294640725	0.207290558
mir-181b-1-5p	mir-93-5p	0.545543834	0.012846949	0.294640725	0.207290558
mir-27b-3p	mir-532-5p	-0.475651319	0.034028057	-0.678149051	0.001015184
mir-192-5p	mir-99a-5p	0.57862605	0.007520689	0.338242163	0.144657038
mir-21-5p	mir-30a-5p	0.06397023	0.78874787	0.356012223	0.123413381

mir-126-3p	mir-486-2-5p	-0.273945912	0.242498005	-0.529424253	0.01636981
mir-92a-2-3p	mir-27a-3p	0.071290355	0.765190664	-0.232299924	0.324352137
mir-27a-3p	mir-92a-1-3p	0.071290355	0.765190664	-0.232299924	0.324352137
mir-22-3p	mir-126-3p	-0.152600184	0.520688031	0.152968795	0.519669318
mir-199a-1-5p	mir-744-5p	0.837039631	4.19E-06	0.717990654	0.000364014
mir-744-5p	mir-199a-2-5p	0.837039631	4.19E-06	0.717990654	0.000364014
mir-23a-3p	mir-99a-5p	0.26839654	0.252554092	0.524658391	0.017547744
let-7i-5p	norepi	0.533880354	0.015326611	0.280333863	0.231245402
mir-181b-2-5p	mir-146b-5p	-0.373231451	0.105046665	-0.084630035	0.722781011
mir-146b-5p	mir-181b-1-5p	-0.373231451	0.105046665	-0.084630035	0.722781011
mir-7-3-5p	mir-223-3p	-0.188829725	0.425271533	-0.460805144	0.040875877
mir-223-3p	mir-7-1-5p	-0.188829725	0.425271533	-0.460805144	0.040875877
mir-223-3p	mir-7-2-5p	-0.188829725	0.425271533	-0.460805144	0.040875877
mir-98-5p	mir-423-5p	0.366604465	0.111864002	0.077140176	0.746505729
mir-148b-3p	mir-150-5p	-0.169588548	0.474737519	-0.444975278	0.049303817
mir-146b-5p	mir-21-5p	0.862292996	1.01E-06	0.923058833	6.77E-09
mir-29a-3p	let-7i-5p	0.028161887	0.90618088	0.323167655	0.164583903
mir-92a-2-3p	mir-140-5p	0.011688259	0.960993304	-0.286715821	0.22034738
mir-140-5p	mir-92a-1-3p	0.011688259	0.960993304	-0.286715821	0.22034738
mir-425-5p	mir-185-5p	0.834635163	4.74E-06	0.714780744	0.00039781
mir-584-5p	mir-148b-3p	0.194169351	0.412051043	0.464436506	0.039110118
mir-99b-5p	mir-27a-3p	-0.068449845	0.77430949	0.233139728	0.322556277
mir-181b-2-5p	mir-28-3p	-0.323630995	0.163944707	-0.029642616	0.901270455
mir-181b-1-5p	mir-28-3p	-0.323630995	0.163944707	-0.029642616	0.901270455
mir-21-5p	mir-451a	-0.382188854	0.096317548	-0.609758682	0.004310565
mir-144-3p	mir-23a-3p	-0.335932019	0.147595482	-0.575103001	0.007982739
mir-222-3p	mir-363-3p	0.710170551	0.000451	0.831524105	5.54E-06
mir-451a	mir-374b-5p	-0.054037913	0.820992238	-0.344843387	0.136486684
mir-224-5p	mir-20b-5p	0.124870831	0.599901295	-0.177964642	0.452857849
mir-423-5p	norepi	0.508558514	0.022036949	0.249970358	0.287822122
let-7f-2-5p	mir-144-3p	-0.286968696	0.219922622	-0.537285964	0.014565909
mir-144-3p	let-7f-1-5p	-0.286968696	0.219922622	-0.537285964	0.014565909
mir-223-3p	mir-150-5p	0.097934514	0.681244635	-0.203680375	0.389063898
mir-181a-2-5p	mir-378a-3p	-0.250254124	0.287257094	0.048729468	0.83834312
mir-378a-3p	mir-181a-1-5p	-0.250254124	0.287257094	0.048729468	0.83834312
mir-20a-5p	mir-185-5p	0.974461958	4.00E-13	0.953551424	8.07E-11
mir-99a-5p	mir-20b-5p	0.208670004	0.37729615	-0.092082595	0.699413719
mir-103a-2-3p	let-7a-3-5p	0.723002972	0.000316137	0.839014081	3.79E-06
mir-103a-2-3p	let-7a-2-5p	0.723002972	0.000316137	0.839014081	3.79E-06
mir-103a-2-3p	let-7a-1-5p	0.723002972	0.000316137	0.839014081	3.79E-06
let-7a-3-5p	mir-103a-1-3p	0.723002972	0.000316137	0.839014081	3.79E-06
let-7a-2-5p	mir-103a-1-3p	0.723002972	0.000316137	0.839014081	3.79E-06
mir-103a-1-3p	let-7a-1-5p	0.723002972	0.000316137	0.839014081	3.79E-06
mir-17-5p	norepi	0.224187006	0.342005936	-0.075679596	0.751158673
mir-629-5p	mir-148a-3p	0.111166427	0.640792609	-0.189846269	0.422737372
mir-126-3p	mir-144-3p	-0.202178164	0.392646297	-0.468926427	0.037009304
mir-425-5p	mir-99b-5p	-0.410226664	0.072416024	-0.131474134	0.58059284
mir-22-3p	mir-194-2-5p	0.763419576	9.00E-05	0.604830978	0.004725108
mir-22-3p	mir-194-1-5p	0.763419576	9.00E-05	0.604830978	0.004725108
mir-3615	mir-27a-3p	0.568151662	0.00896247	0.328527699	0.157293771
let-7f-2-5p	mir-106a-5p	-0.384021717	0.094598811	-0.609622732	0.004321584
mir-106a-5p	let-7f-1-5p	-0.384021717	0.094598811	-0.609622732	0.004321584
mir-146b-5p	norepi	0.218132029	0.355539877	0.481571141	0.031561886
mir-101-1-3p	mir-126-5p	0.335294696	0.14841342	0.045418663	0.849201633
mir-126-5p	mir-101-2-3p	0.335294696	0.14841342	0.045418663	0.849201633
mir-151a-3p	mir-185-5p	0.204817687	0.38636383	-0.095125253	0.689946489
mir-125a-5p	mir-23a-3p	0.591041978	0.006063229	0.754100044	0.000122717
mir-148a-3p	mir-363-3p	0.212493765	0.368415457	-0.086722598	0.716194922
mir-194-2-5p	mir-221-3p	-0.495200488	0.026415215	-0.235975498	0.316536104
mir-194-1-5p	mir-221-3p	-0.495200488	0.026415215	-0.235975498	0.316536104
mir-584-5p	mir-151a-5p	0.719683724	0.000347195	0.540308837	0.013916422
mir-185-5p	mir-15b-5p	0.725120084	0.000297591	0.54842415	0.012287449
mir-192-5p	mir-30e-5p	0.766209958	8.18E-05	0.610158311	0.004278309
let-7f-2-5p	mir-223-3p	0.702376027	0.000554687	0.825363157	7.47E-06

mir-223-3p	let-7f-1-5p	0.702376027	0.000554687	0.825363157	7.47E-06
let-7a-3-5p	mir-191-5p	0.920993436	8.53E-09	0.860195977	1.15E-06
let-7a-2-5p	mir-191-5p	0.920993436	8.53E-09	0.860195977	1.15E-06
let-7a-1-5p	mir-191-5p	0.920993436	8.53E-09	0.860195977	1.15E-06
let-7a-3-5p	mir-194-2-5p	-0.459959548	0.041295765	-0.193526285	0.413631313
let-7a-3-5p	mir-194-1-5p	-0.459959548	0.041295765	-0.193526285	0.413631313
let-7a-2-5p	mir-194-2-5p	-0.459959548	0.041295765	-0.193526285	0.413631313
let-7a-2-5p	mir-194-1-5p	-0.459959548	0.041295765	-0.193526285	0.413631313
mir-194-2-5p	let-7a-1-5p	-0.459959548	0.041295765	-0.193526285	0.413631313
let-7a-1-5p	mir-194-1-5p	-0.459959548	0.041295765	-0.193526285	0.413631313
mir-629-5p	mir-125b-1-5p	0.149054284	0.530535417	-0.149589858	0.529042546
mir-629-5p	mir-125b-2-5p	0.149054284	0.530535417	-0.149589858	0.529042546
mir-191-5p	mir-148b-3p	0.366902278	0.111550966	0.595108558	0.005639753
mir-222-3p	mir-423-5p	0.523383508	0.017874069	0.707179303	0.00048865
mir-181a-2-5p	mir-340-5p	0.672106117	0.001170455	0.805798132	1.80E-05
mir-340-5p	mir-181a-1-5p	0.672106117	0.001170455	0.805798132	1.80E-05
mir-222-3p	mir-425-5p	0.796632124	2.63E-05	0.657835742	0.00161813
mir-126-3p	mir-30e-5p	-0.071491131	0.764547227	0.22478873	0.340677657
mir-98-5p	mir-375	-0.006451956	0.978462728	0.285622202	0.222190532
mir-126-3p	mir-30c-1-5p	0.662628756	0.00145404	0.79963148	2.33E-05
mir-126-3p	mir-30c-2-5p	0.662628756	0.00145404	0.79963148	2.33E-05
let-7d-5p	mir-543	0.683956454	0.00088271	0.490274455	0.028192704
let-7f-2-5p	mir-15a-5p	-0.598079147	0.005346047	-0.757320558	0.000110401
mir-15a-5p	let-7f-1-5p	-0.598079147	0.005346047	-0.757320558	0.000110401
mir-425-5p	cpeptide	-0.106690265	0.654375035	0.190304236	0.421598345
mir-25-3p	mir-191-5p	-0.120051647	0.614157927	-0.397127023	0.082957461
mir-101-1-3p	mir-30c-1-5p	-0.385731233	0.093016073	-0.106817606	0.653987161
mir-101-1-3p	mir-30c-2-5p	-0.385731233	0.093016073	-0.106817606	0.653987161
mir-30c-1-5p	mir-101-2-3p	-0.385731233	0.093016073	-0.106817606	0.653987161
mir-101-2-3p	mir-30c-2-5p	-0.385731233	0.093016073	-0.106817606	0.653987161
mir-1307-3p	mir-30e-5p	-0.579340315	0.007429734	-0.347051865	0.133827382
mir-29a-3p	mir-16-2-5p	0.489329122	0.028544324	0.231559172	0.325941108
mir-29a-3p	mir-16-1-5p	0.489329122	0.028544324	0.231559172	0.325941108
mir-21-5p	mir-340-5p	0.944511837	3.87E-10	0.901274611	5.89E-08
mir-29a-3p	mir-223-3p	0.30259045	0.19471866	0.545187741	0.012917525
mir-374b-5p	let-7g-5p	0.418976604	0.065955185	0.63248265	0.002767249
let-7f-2-5p	mir-340-5p	0.925797211	4.94E-09	0.869020573	6.63E-07
mir-340-5p	let-7f-1-5p	0.925797211	4.94E-09	0.869020573	6.63E-07
mir-146a-5p	mir-221-3p	0.90588802	3.89E-08	0.947127413	2.53E-10
mir-122-5p	mir-224-5p	0.363926162	0.114707706	0.591737995	0.005988952
mir-26b-5p	let-7d-5p	-0.250697852	0.286374925	-0.504212518	0.023392941
mir-128-1-3p	mir-150-5p	0.163740734	0.490320903	-0.132707931	0.577014882
mir-150-5p	mir-128-2-3p	0.163740734	0.490320903	-0.132707931	0.577014882
mir-374b-5p	mir-15b-5p	0.431795991	0.05728547	0.6412779	0.002309474
mir-99a-5p	mir-185-5p	0.239128285	0.309922603	-0.054292854	0.820160896
mir-17-5p	mir-103a-2-3p	0.579155968	0.007453123	0.348067888	0.132616335
mir-17-5p	mir-103a-1-3p	0.579155968	0.007453123	0.348067888	0.132616335
let-7i-5p	mir-107	0.495048428	0.026468728	0.240028186	0.308050327
mir-151a-3p	let-7d-3p	0.741569983	0.000182518	0.848792496	2.24E-06
mir-151a-3p	mir-28-3p	0.629760324	0.002923375	0.777343433	5.51E-05
mir-181b-2-5p	mir-629-5p	0.498763666	0.025185504	0.245205724	0.297411527
mir-181b-1-5p	mir-629-5p	0.498763666	0.025185504	0.245205724	0.297411527
mir-484	mir-363-3p	0.699709583	0.00059451	0.514942455	0.020159036
mir-451a	mir-28-5p	-0.184546932	0.436036715	-0.449428414	0.046809239
mir-320a	copeptin	0.026899665	0.910369285	-0.263966412	0.260769729
mir-17-5p	mir-186-5p	0.904249103	4.52E-08	0.833025273	5.14E-06
mir-16-2-5p	mir-106b-3p	0.928185651	3.71E-09	0.87366432	4.88E-07
mir-106b-3p	mir-16-1-5p	0.928185651	3.71E-09	0.87366432	4.88E-07
mir-340-5p	mir-375	-0.291629645	0.212189622	-0.003677728	0.987722456
mir-143-3p	mir-221-3p	-0.130475724	0.583495114	0.163862922	0.489992746
let-7i-5p	mir-146b-5p	0.280814534	0.230412638	0.526107038	0.017182744
mir-532-5p	mir-425-5p	0.616392675	0.003800389	0.399516502	0.080954717
mir-361-5p	mir-374b-5p	0.527665491	0.01679689	0.707847994	0.00048001
let-7a-3-5p	mir-30d-5p	0.864042749	9.11E-07	0.767091874	7.93E-05

let-7a-2-5p	mir-30d-5p	0.864042749	9.11E-07	0.767091874	7.93E-05
let-7a-1-5p	mir-30d-5p	0.864042749	9.11E-07	0.767091874	7.93E-05
mir-194-2-5p	mir-148b-3p	0.276196788	0.238493606	-0.012245936	0.959133647
mir-148b-3p	mir-194-1-5p	0.276196788	0.238493606	-0.012245936	0.959133647
mir-106a-5p	mir-340-5p	-0.259268623	0.269664075	-0.508675388	0.022001364
let-7i-5p	mir-423-3p	0.37263507	0.105647529	0.596085408	0.005541741
mir-21-5p	mir-744-5p	0.888578237	1.67E-07	0.807586207	1.67E-05
mir-146b-5p	mir-10b-5p	0.205629084	0.384443915	-0.086237212	0.717720917
mir-26b-5p	mir-185-5p	0.940178459	7.49E-10	0.894640287	1.03E-07
mir-151a-3p	norepi	0.241550571	0.304898555	0.493816861	0.026905294
mir-126-3p	mir-25-3p	-0.188317754	0.426550898	-0.450420085	0.046267165
mir-21-5p	mir-25-3p	-0.365026855	0.113532821	-0.589667304	0.006212154
mir-103a-2-3p	mir-192-5p	0.292547077	0.210688929	0.007201626	0.975960907
mir-192-5p	mir-103a-1-3p	0.292547077	0.210688929	0.007201626	0.975960907
mir-148a-3p	mir-20a-5p	0.327132061	0.159170034	0.045452214	0.849091464
mir-143-3p	mir-30d-5p	-0.292889978	0.210129834	-0.007954034	0.973450172
mir-24-2-3p	mir-128-1-3p	0.682154285	0.000922157	0.809890394	1.51E-05
mir-24-2-3p	mir-128-2-3p	0.682154285	0.000922157	0.809890394	1.51E-05
mir-128-1-3p	mir-24-1-3p	0.682154285	0.000922157	0.809890394	1.51E-05
mir-128-2-3p	mir-24-1-3p	0.682154285	0.000922157	0.809890394	1.51E-05
mir-26b-5p	mir-532-5p	0.848302719	2.30E-06	0.91270406	2.03E-08
mir-26b-5p	mir-181b-2-5p	0.515788368	0.019920074	0.27036702	0.248953435
mir-26b-5p	mir-181b-1-5p	0.515788368	0.019920074	0.27036702	0.248953435
mir-361-5p	mir-194-2-5p	-0.522075379	0.018213929	-0.278336839	0.234726145
mir-361-5p	mir-194-1-5p	-0.522075379	0.018213929	-0.278336839	0.234726145
mir-26a-2-5p	mir-106a-5p	-0.376100329	0.102190801	-0.59700864	0.005450401
mir-106a-5p	mir-26a-1-5p	-0.376100329	0.102190801	-0.59700864	0.005450401
mir-103a-2-3p	mir-10a-5p	0.226142282	0.337700806	0.479771586	0.032296272
mir-103a-1-3p	mir-10a-5p	0.226142282	0.337700806	0.479771586	0.032296272
let-7i-5p	mir-30a-5p	-0.114848102	0.629702541	0.17528485	0.459800636
mir-148a-3p	mir-28-3p	0.029494778	0.901760566	0.310812005	0.18226534
mir-532-5p	mir-224-5p	-0.077554232	0.745188205	-0.353648436	0.126102614
mir-181a-2-5p	mir-15b-5p	0.446208858	0.048602817	0.648005798	0.00200361
mir-181a-1-5p	mir-15b-5p	0.446208858	0.048602817	0.648005798	0.00200361
mir-143-3p	mir-199a-2-3p	-0.058726743	0.805732625	0.228903996	0.331674557
mir-143-3p	mir-199a-1-3p	-0.058726743	0.805732625	0.228903996	0.331674557
mir-143-3p	mir-199b-3p	-0.058726743	0.805732625	0.228903996	0.331674557
mir-3615	mir-128-1-3p	-0.083619404	0.725968617	-0.358778385	0.120318601
mir-3615	mir-128-2-3p	-0.083619404	0.725968617	-0.358778385	0.120318601
mir-361-5p	mir-181a-2-5p	0.551555955	0.01170162	0.722102778	0.000324316
mir-361-5p	mir-181a-1-5p	0.551555955	0.01170162	0.722102778	0.000324316
mir-451a	mir-223-3p	-0.182139784	0.442149676	-0.44270461	0.050614375
mir-423-3p	mir-150-5p	-0.386780885	0.092053932	-0.603704836	0.004824321
mir-25-3p	mir-224-5p	0.115533548	0.627646125	-0.173117578	0.465455137
mir-199a-1-5p	let-7d-3p	0.774458025	6.11E-05	0.629486874	0.002939454
mir-199a-2-5p	let-7d-3p	0.774458025	6.11E-05	0.629486874	0.002939454
mir-29a-3p	mir-144-3p	0.439756252	0.052355639	0.179207312	0.449656817
mir-151a-5p	mir-139-5p	0.37539697	0.102885687	0.594986292	0.00565212
mir-16-2-5p	let-7d-5p	-0.304777147	0.191352251	-0.540773567	0.013818673
let-7d-5p	mir-16-1-5p	-0.304777147	0.191352251	-0.540773567	0.013818673
mir-151a-5p	mir-20a-5p	-0.298188632	0.201615024	-0.017093406	0.942978606
mir-106a-5p	mir-146b-5p	-0.558392084	0.010501214	-0.726283636	0.0002878
mir-21-5p	mir-15a-5p	-0.639330714	0.00240494	-0.780742791	4.86E-05
mir-22-3p	mir-199a-1-5p	-0.267779864	0.253687718	0.015751089	0.947450228
mir-22-3p	mir-199a-2-5p	-0.267779864	0.253687718	0.015751089	0.947450228
mir-140-5p	mir-199a-2-3p	0.548498594	0.012273254	0.315279146	0.17573039
mir-140-5p	mir-199a-1-3p	0.548498594	0.012273254	0.315279146	0.17573039
mir-140-5p	mir-199b-3p	0.548498594	0.012273254	0.315279146	0.17573039
mir-361-5p	mir-144-3p	-0.54989773	0.01200892	-0.720198597	0.000342214
mir-423-5p	mir-363-3p	0.608730903	0.004394447	0.39454979	0.085158414
mir-25-3p	let-7d-5p	-0.310656178	0.182496229	-0.544784277	0.012997868
mir-191-5p	mir-28-5p	0.767308763	7.87E-05	0.86257398	9.98E-07
mir-22-3p	mir-363-3p	0.740670263	0.000187636	0.580237312	0.007316788
mir-223-3p	mir-99b-5p	0.654443853	0.001743408	0.457748735	0.042409327

let-7i-5p	mir-221-3p	0.251596904	0.284592667	0.497220695	0.025712332
mir-92a-2-3p	let-7g-5p	0.036514389	0.878529999	-0.246744431	0.294293615
let-7g-5p	mir-92a-1-3p	0.036514389	0.878529999	-0.246744431	0.294293615
mir-199a-1-5p	mir-125b-1-5p	-0.309094139	0.184821642	-0.031073949	0.896527117
mir-199a-1-5p	mir-125b-2-5p	-0.309094139	0.184821642	-0.031073949	0.896527117
mir-125b-1-5p	mir-199a-2-5p	-0.309094139	0.184821642	-0.031073949	0.896527117
mir-199a-2-5p	mir-125b-2-5p	-0.309094139	0.184821642	-0.031073949	0.896527117
mir-24-2-3p	mir-28-3p	0.547887811	0.012390112	0.71810285	0.000362878
mir-24-1-3p	mir-28-3p	0.547887811	0.012390112	0.71810285	0.000362878
mir-186-5p	mir-28-3p	-0.158633047	0.504134463	-0.420415781	0.064935491
mir-29a-3p	mir-93-5p	0.479494827	0.032410391	0.229934242	0.329442866
mir-194-2-5p	mir-93-5p	0.784215301	4.27E-05	0.646086739	0.002087178
mir-194-1-5p	mir-93-5p	0.784215301	4.27E-05	0.646086739	0.002087178
mir-7-3-5p	let-7d-5p	-0.297070851	0.203391828	-0.532719138	0.015593164
mir-7-1-5p	let-7d-5p	-0.297070851	0.203391828	-0.532719138	0.015593164
let-7d-5p	mir-7-2-5p	-0.297070851	0.203391828	-0.532719138	0.015593164
mir-224-5p	mir-106b-3p	-0.018467612	0.938402456	-0.296459118	0.204368624
mir-106a-5p	mir-584-5p	-0.339684293	0.142843583	-0.565500391	0.009361021
mir-224-5p	mir-28-3p	0.320946986	0.167671201	0.550824902	0.011836304
mir-451a	mir-378a-3p	0.809361359	1.55E-05	0.684938924	0.000861812
mir-191-5p	let-7d-3p	0.905969865	3.86E-08	0.839023448	3.78E-06
mir-194-2-5p	let-7d-3p	-0.458243205	0.042158277	-0.205591816	0.384531982
mir-194-1-5p	let-7d-3p	-0.458243205	0.042158277	-0.205591816	0.384531982
mir-26b-5p	mir-181a-2-5p	0.182780886	0.440517234	-0.100981708	0.671849433
mir-26b-5p	mir-181a-1-5p	0.182780886	0.440517234	-0.100981708	0.671849433
mir-584-5p	mir-28-5p	0.513712503	0.020510552	0.69307093	0.000704324
mir-15a-5p	let-7g-5p	0.318514975	0.171097639	0.044062385	0.853657523
mir-17-5p	mir-98-5p	0.21387593	0.365234888	-0.068549597	0.773988768
mir-101-1-3p	mir-23a-3p	-0.226962452	0.33590447	-0.475129224	0.034252571
mir-101-2-3p	mir-23a-3p	-0.226962452	0.33590447	-0.475129224	0.034252571
mir-361-5p	mir-128-1-3p	0.530814831	0.016038337	0.704840274	0.000519921
mir-361-5p	mir-128-2-3p	0.530814831	0.016038337	0.704840274	0.000519921
let-7f-2-5p	mir-543	0.692827434	0.000708658	0.513808382	0.020482976
mir-543	let-7f-1-5p	0.692827434	0.000708658	0.513808382	0.020482976
mir-7-3-5p	mir-150-5p	0.147273154	0.535514187	0.408511879	0.073735756
mir-7-1-5p	mir-150-5p	0.147273154	0.535514187	0.408511879	0.073735756
mir-150-5p	mir-7-2-5p	0.147273154	0.535514187	0.408511879	0.073735756
mir-144-3p	let-7b-5p	0.923363484	6.54E-09	0.868254032	6.97E-07
mir-99a-5p	mir-93-5p	0.158297057	0.505049659	-0.125086389	0.599266819
let-7b-5p	mir-99b-5p	-0.707780885	0.000480871	-0.535207788	0.015026399
mir-181a-2-5p	mir-221-3p	0.631072714	0.002847218	0.773293256	6.38E-05
mir-221-3p	mir-181a-1-5p	0.631072714	0.002847218	0.773293256	6.38E-05
mir-10b-5p	mir-363-3p	-0.002885096	0.990368407	0.275090429	0.2404565
mir-7-3-5p	cpeptide	-0.108663837	0.648373239	0.174192976	0.462644975
mir-7-1-5p	cpeptide	-0.108663837	0.648373239	0.174192976	0.462644975
mir-7-2-5p	cpeptide	-0.108663837	0.648373239	0.174192976	0.462644975
mir-7-3-5p	mir-378a-3p	0.755725883	0.00011636	0.605209646	0.004692128
mir-7-1-5p	mir-378a-3p	0.755725883	0.00011636	0.605209646	0.004692128
mir-378a-3p	mir-7-2-5p	0.755725883	0.00011636	0.605209646	0.004692128
mir-182-5p	mir-532-5p	0.788393032	3.64E-05	0.874501705	4.61E-07
mir-10b-5p	mir-20a-5p	-0.105401522	0.658305289	0.177051504	0.455217502
mir-584-5p	mir-7-3-5p	-0.327476816	0.158705122	-0.554404809	0.011188551
mir-584-5p	mir-7-1-5p	-0.327476816	0.158705122	-0.554404809	0.011188551
mir-584-5p	mir-7-2-5p	-0.327476816	0.158705122	-0.554404809	0.011188551
mir-144-3p	mir-24-2-3p	-0.322071858	0.166102415	-0.55017426	0.011957226
mir-144-3p	mir-24-1-3p	-0.322071858	0.166102415	-0.55017426	0.011957226
mir-122-5p	mir-151a-3p	0.159272288	0.502395457	0.418025778	0.066635449
mir-340-5p	mir-15b-5p	0.264096084	0.260526882	0.504175975	0.023404616
mir-27b-3p	mir-148a-3p	0.269575939	0.250395015	0.508511545	0.022051262
mir-194-2-5p	mir-125b-1-5p	0.580096857	0.00733438	0.361411684	0.117424404
mir-194-2-5p	mir-125b-2-5p	0.580096857	0.00733438	0.361411684	0.117424404
mir-125b-1-5p	mir-194-1-5p	0.580096857	0.00733438	0.361411684	0.117424404
mir-194-1-5p	mir-125b-2-5p	0.580096857	0.00733438	0.361411684	0.117424404
mir-30d-5p	mir-10a-5p	0.20003587	0.397786597	0.451705402	0.045571772

mir-744-5p	mir-378a-3p	-0.61006771	0.004285604	-0.401217516	0.07955101
mir-451a	mir-15a-5p	0.800524969	2.24E-05	0.881777636	2.77E-07
let-7g-5p	mir-20b-5p	0.509962012	0.021612636	0.272296272	0.245460047
mir-222-3p	copeptin	0.161506128	0.496341321	-0.119744068	0.615072454
mir-182-5p	mir-320a	0.787939283	3.70E-05	0.654301433	0.001748841
let-7i-5p	mir-21-5p	0.426655305	0.060651445	0.628260252	0.00301249
mir-125a-5p	mir-320a	-0.552573481	0.011516226	-0.326978151	0.15937789
let-7f-2-5p	mir-106b-3p	-0.432279967	0.05697604	-0.63232718	0.002775974
mir-106b-3p	let-7f-1-5p	-0.432279967	0.05697604	-0.63232718	0.002775974
mir-103a-2-3p	mir-30a-5p	-0.019220795	0.93589511	0.256860012	0.274297124
mir-103a-1-3p	mir-30a-5p	-0.019220795	0.93589511	0.256860012	0.274297124
mir-26a-2-5p	mir-146b-5p	0.834307181	4.82E-06	0.902233106	5.41E-08
mir-146b-5p	mir-26a-1-5p	0.834307181	4.82E-06	0.902233106	5.41E-08
mir-532-5p	let-7b-5p	0.962178712	1.31E-11	0.934492838	1.66E-09
mir-451a	mir-186-5p	0.868805829	6.73E-07	0.780437575	4.92E-05
mir-320a	mir-15b-5p	0.657401844	0.001633721	0.467322824	0.037749301
mir-26b-5p	mir-144-3p	0.912506878	2.07E-08	0.94922281	1.77E-10
mir-25-3p	mir-28-5p	-0.143331835	0.546607138	-0.401772283	0.079097138
mir-140-5p	mir-7-3-5p	0.523183644	0.017925665	0.290725582	0.213675331
mir-140-5p	mir-7-1-5p	0.523183644	0.017925665	0.290725582	0.213675331
mir-140-5p	mir-7-2-5p	0.523183644	0.017925665	0.290725582	0.213675331
mir-122-5p	cpeptide	0.268069666	0.253154577	-0.006466514	0.978414142
mir-181b-2-5p	mir-320a	0.549262859	0.012128288	0.324077024	0.163331008
mir-181b-1-5p	mir-320a	0.549262859	0.012128288	0.324077024	0.163331008
mir-146a-5p	cpeptide	-0.053699552	0.822095888	-0.322749986	0.165161562
mir-106b-3p	cpeptide	-0.20081915	0.395902885	0.077218714	0.746255767
mir-101-1-3p	mir-27a-3p	0.699371567	0.000599727	0.526428429	0.017102596
mir-27a-3p	mir-101-2-3p	0.699371567	0.000599727	0.526428429	0.017102596
mir-125b-1-5p	mir-126-5p	0.023980339	0.920064966	0.295712404	0.205565171
mir-126-5p	mir-125b-2-5p	0.023980339	0.920064966	0.295712404	0.205565171
mir-128-1-3p	mir-28-5p	0.617385243	0.003728551	0.762254475	9.36E-05
mir-128-2-3p	mir-28-5p	0.617385243	0.003728551	0.762254475	9.36E-05
mir-30d-5p	mir-28-5p	0.594122654	0.005740115	0.746372567	0.000157173
mir-140-5p	mir-148a-3p	0.03013379	0.89964237	0.301074774	0.197075185
mir-199a-2-3p	mir-185-5p	-0.062859696	0.792337742	-0.33040339	0.15479633
mir-199a-1-3p	mir-185-5p	-0.062859696	0.792337742	-0.33040339	0.15479633
mir-199b-3p	mir-185-5p	-0.062859696	0.792337742	-0.33040339	0.15479633
mir-144-3p	mir-20b-5p	0.921790099	7.81E-09	0.866945443	7.58E-07
mir-26a-2-5p	mir-25-3p	-0.32967228	0.155766494	-0.55294558	0.011449026
mir-25-3p	mir-26a-1-5p	-0.32967228	0.155766494	-0.55294558	0.011449026
let-7f-2-5p	let-7d-5p	0.929153914	3.30E-09	0.958919986	2.73E-11
let-7d-5p	let-7f-1-5p	0.929153914	3.30E-09	0.958919986	2.73E-11
mir-27a-3p	mir-150-5p	0.617743224	0.00370292	0.41469292	0.069061633
mir-10b-5p	mir-151a-5p	-0.085703968	0.71939856	-0.349963555	0.130377558
mir-25-3p	mir-378a-3p	0.78389374	4.32E-05	0.650368745	0.001904575
mir-17-5p	mir-148b-3p	0.586115681	0.006610875	0.373334411	0.104943181
mir-374b-5p	mir-363-3p	-0.033174927	0.889570782	-0.302741069	0.194485519
mir-26b-5p	mir-191-5p	-0.10153157	0.670159011	-0.363622396	0.11503348
mir-374b-5p	mir-93-5p	0.13498331	0.570441422	-0.142208471	0.549787798
mir-29a-3p	mir-584-5p	-0.162691205	0.493144038	0.114311872	0.631313123
let-7a-3-5p	mir-378a-3p	-0.438901622	0.052868822	-0.189587301	0.42338219
let-7a-2-5p	mir-378a-3p	-0.438901622	0.052868822	-0.189587301	0.42338219
let-7a-1-5p	mir-378a-3p	-0.438901622	0.052868822	-0.189587301	0.42338219
mir-144-3p	mir-139-5p	-0.143734457	0.545469199	-0.400017014	0.080539794
mir-17-5p	mir-92a-2-3p	0.328107007	0.157857719	0.061697257	0.796099715
mir-17-5p	mir-92a-1-3p	0.328107007	0.157857719	0.061697257	0.796099715
let-7i-5p	mir-191-5p	0.557010628	0.01073536	0.719865128	0.000345433
mir-148a-3p	mir-543	-0.176759188	0.455974215	0.09983248	0.675387357
mir-17-5p	mir-423-5p	0.838909622	3.81E-06	0.73467962	0.000224948
mir-194-2-5p	mir-543	-0.264927882	0.258972515	0.007233636	0.97585409
mir-194-1-5p	mir-543	-0.264927882	0.258972515	0.007233636	0.97585409
mir-144-3p	mir-7-3-5p	0.925583861	5.06E-09	0.873659209	4.88E-07
mir-144-3p	mir-7-1-5p	0.925583861	5.06E-09	0.873659209	4.88E-07
mir-144-3p	mir-7-2-5p	0.925583861	5.06E-09	0.873659209	4.88E-07

mir-106a-5p	mir-223-3p	-0.194993706	0.41003007	-0.442809551	0.050553226
mir-126-3p	mir-7-3-5p	-0.212543237	0.368301345	-0.457364723	0.04260509
mir-126-3p	mir-7-1-5p	-0.212543237	0.368301345	-0.457364723	0.04260509
mir-126-3p	mir-7-2-5p	-0.212543237	0.368301345	-0.457364723	0.04260509
mir-126-3p	mir-99b-5p	0.826893642	6.94E-06	0.716385751	0.000380592
mir-340-5p	mir-451a	-0.242023827	0.303922779	-0.481430005	0.031619006
mir-423-3p	mir-25-3p	-0.46404945	0.039295459	-0.652754383	0.001808779
mir-106a-5p	let-7d-3p	-0.377695197	0.100627792	-0.588244917	0.006369397
mir-107	mir-20b-5p	0.924774373	5.56E-09	0.872549381	5.26E-07
mir-150-5p	mir-30e-5p	-0.203054265	0.3905548	0.071305974	0.765140605
mir-1307-3p	mir-128-1-3p	0.226649728	0.336588734	0.468317715	0.03728886
mir-1307-3p	mir-128-2-3p	0.226649728	0.336588734	0.468317715	0.03728886
mir-629-5p	mir-27a-3p	0.437361804	0.053803117	0.189554958	0.423462759
mir-107	mir-375	-0.049195668	0.836816326	0.223978614	0.342466657
mir-199a-2-3p	mir-28-5p	0.747181577	0.000153215	0.846480431	2.54E-06
mir-199a-1-3p	mir-28-5p	0.747181577	0.000153215	0.846480431	2.54E-06
mir-199b-3p	mir-28-5p	0.747181577	0.000153215	0.846480431	2.54E-06
mir-151a-5p	mir-28-5p	0.496625768	0.025917771	0.67601792	0.001067828
mir-486-2-5p	mir-27a-3p	0.544205184	0.013113889	0.321630476	0.166716775
mir-451a	mir-30e-5p	0.722603763	0.000319742	0.562520974	0.00982599
mir-144-3p	mir-543	-0.256136132	0.27569918	-0.491824178	0.027623659
mir-7-3-5p	mir-224-5p	0.126526251	0.59503586	-0.147942841	0.533639698
mir-224-5p	mir-7-1-5p	0.126526251	0.59503586	-0.147942841	0.533639698
mir-224-5p	mir-7-2-5p	0.126526251	0.59503586	-0.147942841	0.533639698
mir-22-3p	let-7f-2-5p	-0.315864262	0.174886415	-0.050804298	0.831552443
mir-22-3p	let-7f-1-5p	-0.315864262	0.174886415	-0.050804298	0.831552443
mir-25-3p	mir-23a-3p	-0.234965148	0.318673241	-0.474091281	0.034702344
mir-26a-2-5p	mir-10b-5p	0.073814777	0.757111416	-0.199178839	0.399853317
mir-10b-5p	mir-26a-1-5p	0.073814777	0.757111416	-0.199178839	0.399853317
mir-192-5p	mir-122-5p	0.452169374	0.045322735	0.208566558	0.377538065
mir-143-3p	mir-10a-5p	0.309260648	0.184572811	0.044085098	0.853582865
mir-98-5p	mir-532-5p	-0.411721815	0.071279808	-0.612696629	0.004078057
mir-106b-3p	mir-99b-5p	-0.653215076	0.001790753	-0.466350353	0.03820362
mir-361-5p	cpeptide	-0.117731696	0.621069293	-0.374582871	0.103694256
mir-151a-3p	mir-148b-3p	0.369322209	0.1090307	0.579986483	0.007348229
mir-126-3p	mir-194-2-5p	-0.279810384	0.232154548	-0.012812383	0.957244957
mir-126-3p	mir-194-1-5p	-0.279810384	0.232154548	-0.012812383	0.957244957
mir-125b-1-5p	mir-15b-5p	-0.094298281	0.692515295	0.178141649	0.452401174
mir-125b-2-5p	mir-15b-5p	-0.094298281	0.692515295	0.178141649	0.452401174
mir-484	copeptin	-0.130721422	0.582780324	-0.385134199	0.093566608
mir-182-5p	mir-185-5p	0.7615593	9.58E-05	0.620328979	0.003522085
mir-181a-2-5p	mir-7-3-5p	0.094099037	0.693134688	-0.177723526	0.453480307
mir-181a-2-5p	mir-7-1-5p	0.094099037	0.693134688	-0.177723526	0.453480307
mir-181a-2-5p	mir-7-2-5p	0.094099037	0.693134688	-0.177723526	0.453480307
mir-7-3-5p	mir-181a-1-5p	0.094099037	0.693134688	-0.177723526	0.453480307
mir-7-1-5p	mir-181a-1-5p	0.094099037	0.693134688	-0.177723526	0.453480307
mir-7-2-5p	mir-181a-1-5p	0.094099037	0.693134688	-0.177723526	0.453480307
mir-144-3p	mir-125a-5p	-0.539439513	0.014100764	-0.705058548	0.000516933
let-7i-5p	mir-122-5p	-0.312920384	0.179160807	-0.049889463	0.834545203
mir-3615	mir-139-5p	-0.251599713	0.284587109	-0.485949375	0.029829712
mir-92a-2-3p	mir-107	0.61000881	0.004290353	0.409940834	0.072634768
mir-107	mir-92a-1-3p	0.61000881	0.004290353	0.409940834	0.072634768
mir-92a-2-3p	mir-629-5p	0.709054044	0.000464754	0.820538231	9.37E-06
mir-629-5p	mir-92a-1-3p	0.709054044	0.000464754	0.820538231	9.37E-06
mir-23a-3p	mir-10a-5p	0.537129135	0.014600258	0.702910732	0.000546981
mir-10b-5p	mir-148b-3p	-0.118795513	0.617896235	-0.373322411	0.104955238
let-7i-5p	mir-185-5p	0.62452481	0.003244267	0.429705857	0.058636472
let-7a-3-5p	mir-126-5p	0.517213384	0.019522629	0.688601356	0.000787552
let-7a-2-5p	mir-126-5p	0.517213384	0.019522629	0.688601356	0.000787552
let-7a-1-5p	mir-126-5p	0.517213384	0.019522629	0.688601356	0.000787552
mir-103a-2-3p	mir-27a-3p	0.221794084	0.347318093	0.460748165	0.040904066
mir-103a-1-3p	mir-27a-3p	0.221794084	0.347318093	0.460748165	0.040904066
mir-423-5p	mir-24-2-3p	-0.067993736	0.775776414	0.20150088	0.394267405
mir-423-5p	mir-24-1-3p	-0.067993736	0.775776414	0.20150088	0.394267405

mir-320a	mir-20b-5p	0.895057011	9.96E-08	0.825719971	7.34E-06
mir-125a-5p	mir-139-5p	0.561079932	0.010057521	0.719485143	0.000349133
mir-139-5p	mir-106b-3p	-0.272699634	0.244733652	-0.501821728	0.024166583
mir-181b-2-5p	mir-21-5p	-0.126859919	0.594057178	0.143230481	0.546893768
mir-21-5p	mir-181b-1-5p	-0.126859919	0.594057178	0.143230481	0.546893768
mir-17-5p	mir-7-3-5p	0.772365764	6.59E-05	0.637779564	0.002483326
mir-17-5p	mir-7-1-5p	0.772365764	6.59E-05	0.637779564	0.002483326
mir-17-5p	mir-7-2-5p	0.772365764	6.59E-05	0.637779564	0.002483326
mir-543	mir-99b-5p	0.756284176	0.000114243	0.614459931	0.003943564
mir-27b-3p	mir-30e-5p	-0.237332881	0.313678468	0.029464597	0.901860628
mir-29a-3p	let-7g-5p	0.214664296	0.363427781	0.453789854	0.04446115
mir-26a-2-5p	mir-194-2-5p	-0.459069557	0.041741292	-0.221026859	0.349031386
mir-26a-2-5p	mir-194-1-5p	-0.459069557	0.041741292	-0.221026859	0.349031386
mir-194-2-5p	mir-26a-1-5p	-0.459069557	0.041741292	-0.221026859	0.349031386
mir-194-1-5p	mir-26a-1-5p	-0.459069557	0.041741292	-0.221026859	0.349031386
mir-101-1-3p	mir-363-3p	0.945109809	3.52E-10	0.90740726	3.38E-08
mir-101-2-3p	mir-363-3p	0.945109809	3.52E-10	0.90740726	3.38E-08
let-7f-2-5p	mir-374b-5p	0.633819773	0.002693146	0.769375389	7.32E-05
mir-374b-5p	let-7f-1-5p	0.633819773	0.002693146	0.769375389	7.32E-05
mir-140-5p	mir-30e-5p	0.742199407	0.000179009	0.841520159	3.32E-06
mir-181a-2-5p	mir-3615	-0.095489155	0.688817137	-0.351069225	0.129084202
mir-3615	mir-181a-1-5p	-0.095489155	0.688817137	-0.351069225	0.129084202
mir-98-5p	mir-21-5p	0.789185809	3.53E-05	0.871708361	5.56E-07
mir-451a	mir-15b-5p	0.468882491	0.037029427	0.233465894	0.321860396
mir-192-5p	mir-629-5p	0.744354188	0.000167431	0.597590283	0.005393496
mir-103a-2-3p	mir-543	0.255016451	0.277876631	0.48653435	0.029604043
mir-103a-1-3p	mir-543	0.255016451	0.277876631	0.48653435	0.029604043
mir-29a-3p	mir-15a-5p	0.289223626	0.216158723	0.027144697	0.909556017
mir-106a-5p	cpeptide	-0.147521821	0.534817803	0.121292167	0.610475056
mir-222-3p	mir-25-3p	0.841049914	3.40E-06	0.904280614	4.51E-08
mir-451a	mir-150-5p	0.269407753	0.250702184	0.498002009	0.025444486
mir-99b-5p	cpeptide	-0.020088002	0.933008902	-0.282233881	0.227964996
mir-221-3p	mir-363-3p	-0.488893131	0.028707649	-0.666589821	0.001329202
mir-222-3p	mir-361-5p	-0.241446077	0.305114259	-0.474589823	0.034485739
mir-222-3p	mir-16-2-5p	0.808935727	1.57E-05	0.883918149	2.37E-07
mir-222-3p	mir-16-1-5p	0.808935727	1.57E-05	0.883918149	2.37E-07
mir-25-3p	let-7g-5p	0.560956046	0.010077631	0.349497403	0.130925581
mir-378a-3p	mir-27a-3p	0.579858996	0.007364251	0.731280956	0.000248807
mir-423-5p	mir-486-2-5p	0.734920649	0.000223332	0.585141028	0.006723889
mir-139-5p	mir-15b-5p	0.238674981	0.310868312	0.47185577	0.035686692
mir-106a-5p	let-7a-3-5p	-0.353079362	0.126756216	-0.563387966	0.009688792
mir-106a-5p	let-7a-2-5p	-0.353079362	0.126756216	-0.563387966	0.009688792
mir-106a-5p	let-7a-1-5p	-0.353079362	0.126756216	-0.563387966	0.009688792
mir-222-3p	mir-30e-5p	0.688551997	0.000788515	0.805375036	1.83E-05
mir-27a-3p	copeptin	0.218081496	0.355654107	-0.046652057	0.845153286
mir-194-2-5p	mir-191-5p	-0.316175203	0.174439039	-0.059289118	0.803906818
mir-191-5p	mir-194-1-5p	-0.316175203	0.174439039	-0.059289118	0.803906818
mir-19b-2-3p	mir-151a-3p	0.03959468	0.868365137	-0.224382052	0.341575049
mir-19b-1-3p	mir-151a-3p	0.03959468	0.868365137	-0.224382052	0.341575049
mir-425-5p	mir-28-5p	0.09178696	0.700335894	0.345087855	0.136190493
mir-126-5p	mir-23a-3p	0.482539224	0.031172264	0.660667215	0.001519418
mir-744-5p	mir-15b-5p	-0.094997667	0.690342594	0.17053721	0.472233175
mir-320a	mir-27a-3p	0.603929037	0.004804433	0.406833947	0.075044427
mir-24-2-3p	mir-15a-5p	-0.659262785	0.001567728	-0.785253721	4.10E-05
mir-15a-5p	mir-24-1-3p	-0.659262785	0.001567728	-0.785253721	4.10E-05
mir-27b-3p	mir-543	0.631779004	0.002806922	0.443894529	0.04992431
mir-122-5p	mir-128-1-3p	0.090754562	0.703559416	0.343622443	0.137972746
mir-122-5p	mir-128-2-3p	0.090754562	0.703559416	0.343622443	0.137972746
mir-423-5p	mir-28-3p	0.038840769	0.87085124	-0.22419526	0.341987697
mir-144-3p	mir-374b-5p	0.082608209	0.72916235	-0.18183279	0.442932495
mir-320a	mir-185-5p	0.961917652	1.39E-11	0.935948248	1.36E-09
mir-144-3p	mir-30d-5p	0.074190428	0.755911218	-0.189796431	0.422861426
mir-199a-2-3p	mir-107	-0.233024672	0.322801965	-0.464987537	0.03884743
mir-107	mir-199a-1-3p	-0.233024672	0.322801965	-0.464987537	0.03884743

mir-107	mir-199b-3p	-0.233024672	0.322801965	-0.464987537	0.03884743
mir-423-3p	mir-451a	-0.520956274	0.01850876	-0.687837994	0.000802566
mir-320a	mir-107	0.904823988	4.29E-08	0.843185431	3.04E-06
mir-24-2-3p	mir-23a-3p	0.774726215	6.06E-05	0.861224177	1.08E-06
mir-23a-3p	mir-24-1-3p	0.774726215	6.06E-05	0.861224177	1.08E-06
mir-125a-5p	mir-27a-3p	-0.139560631	0.557317602	0.124701229	0.600400699
mir-378a-3p	copeptin	0.24292994	0.30205982	-0.017874505	0.940377302
mir-532-5p	mir-106b-3p	0.93266908	2.11E-09	0.888111734	1.73E-07
mir-423-5p	mir-106b-3p	0.623655718	0.003300265	0.434545429	0.055544468
mir-1307-3p	mir-122-5p	0.275310005	0.240066107	0.499034483	0.025093923
mir-150-5p	mir-15b-5p	-0.242628501	0.302678802	0.0178432	0.940481544
mir-25-3p	mir-375	-0.055157923	0.817341362	0.207014486	0.381178156
mir-22-3p	mir-19b-2-3p	0.900421415	6.34E-08	0.836453609	4.32E-06
mir-22-3p	mir-19b-1-3p	0.900421415	6.34E-08	0.836453609	4.32E-06
let-7d-3p	mir-363-3p	-0.393408397	0.086146818	-0.592178439	0.005942336
let-7b-5p	mir-374b-5p	-0.172379302	0.467389355	-0.412726931	0.07052352
mir-151a-3p	mir-150-5p	0.132469311	0.577706128	-0.130372072	0.583796774
mir-26a-2-5p	mir-15b-5p	0.168635423	0.477260339	0.409157543	0.073236749
mir-26a-1-5p	mir-15b-5p	0.168635423	0.477260339	0.409157543	0.073236749
mir-29a-3p	mir-361-5p	0.012094873	0.95963737	0.26954467	0.250452105
mir-143-3p	mir-486-2-5p	-0.309915446	0.183596476	-0.52609268	0.017186331
let-7a-3-5p	mir-30c-1-5p	0.870300161	6.10E-07	0.789477244	3.49E-05
let-7a-3-5p	mir-30c-2-5p	0.870300161	6.10E-07	0.789477244	3.49E-05
let-7a-2-5p	mir-30c-1-5p	0.870300161	6.10E-07	0.789477244	3.49E-05
let-7a-2-5p	mir-30c-2-5p	0.870300161	6.10E-07	0.789477244	3.49E-05
let-7a-1-5p	mir-30c-1-5p	0.870300161	6.10E-07	0.789477244	3.49E-05
let-7a-1-5p	mir-30c-2-5p	0.870300161	6.10E-07	0.789477244	3.49E-05
mir-144-3p	mir-629-5p	0.907001305	3.51E-08	0.847206755	2.44E-06
mir-425-5p	mir-24-2-3p	-0.02750296	0.908367094	0.232338917	0.324268621
mir-425-5p	mir-24-1-3p	-0.02750296	0.908367094	0.232338917	0.324268621
mir-151a-5p	mir-375	0.040641261	0.864915892	-0.219769955	0.35184876
mir-182-5p	mir-378a-3p	0.623679736	0.003298706	0.435742872	0.054798932
mir-378a-3p	cpeptide	0.085095391	0.721314717	0.335645856	0.147962352
mir-181a-2-5p	mir-151a-5p	0.500183795	0.02470819	0.671381166	0.001190358
mir-151a-5p	mir-181a-1-5p	0.500183795	0.02470819	0.671381166	0.001190358
mir-92a-2-3p	mir-340-5p	-0.61945721	0.003582215	-0.756422658	0.000113723
mir-340-5p	mir-92a-1-3p	-0.61945721	0.003582215	-0.756422658	0.000113723
mir-144-3p	mir-223-3p	-0.084072327	0.724539525	-0.334541163	0.149384574
mir-151a-5p	mir-30e-5p	0.069012185	0.772501923	0.320938908	0.167682502
mir-106a-5p	mir-150-5p	0.142983443	0.547592675	0.386377427	0.092422884
mir-139-5p	mir-93-5p	0.048321333	0.839680209	-0.211867772	0.369861125
mir-148a-3p	mir-10a-5p	0.21381713	0.365369875	-0.046108243	0.846937783
mir-199a-1-5p	mir-363-3p	-0.409368183	0.073074501	-0.60315764	0.004873146
mir-199a-2-5p	mir-363-3p	-0.409368183	0.073074501	-0.60315764	0.004873146
mir-151a-5p	mir-484	0.051080207	0.830650278	0.304307963	0.192071238
mir-151a-5p	let-7g-5p	0.105383787	0.658359436	0.353003547	0.126843473
mir-532-5p	cpeptide	-0.053032336	0.82427312	0.206822092	0.381630747
mir-21-5p	mir-363-3p	-0.397995686	0.082225195	-0.594233044	0.005728805
mir-186-5p	mir-363-3p	0.899443716	6.90E-08	0.835622453	4.51E-06
mir-423-5p	mir-223-3p	0.26219327	0.264104769	0.006035703	0.979851941
mir-7-3-5p	mir-191-5p	-0.169931194	0.473832211	-0.408566088	0.073693762
mir-191-5p	mir-7-1-5p	-0.169931194	0.473832211	-0.408566088	0.073693762
mir-191-5p	mir-7-2-5p	-0.169931194	0.473832211	-0.408566088	0.073693762
mir-17-5p	let-7b-5p	0.736463397	0.000213228	0.591903499	0.0059714
mir-19b-2-3p	mir-23a-3p	-0.149605667	0.528998508	-0.390859493	0.088384589
mir-19b-1-3p	mir-23a-3p	-0.149605667	0.528998508	-0.390859493	0.088384589
mir-30c-1-5p	mir-484	0.103112249	0.665308102	0.350078697	0.130242445
mir-484	mir-30c-2-5p	0.103112249	0.665308102	0.350078697	0.130242445
mir-29a-3p	mir-192-5p	0.539944565	0.013993429	0.329370216	0.156168552
mir-21-5p	mir-378a-3p	-0.427576578	0.060037479	-0.192556254	0.416021254
mir-106a-5p	let-7g-5p	0.522387507	0.018132371	0.307722034	0.186880755
mir-28-5p	norepi	0.180462271	0.446436064	0.416950261	0.067411267
mir-199a-1-5p	let-7b-5p	-0.579554947	0.00740258	-0.727062441	0.0002814
let-7b-5p	mir-199a-2-5p	-0.579554947	0.00740258	-0.727062441	0.0002814

mir-222-3p	mir-144-3p	0.684967973	0.0008612	0.800166406	2.28E-05
mir-21-5p	let-7b-5p	-0.499154756	0.025053336	-0.668918922	0.001260099
mir-24-2-3p	copeptin	-0.049910329	0.834476918	-0.300894976	0.197355987
mir-24-1-3p	copeptin	-0.049910329	0.834476918	-0.300894976	0.197355987
mir-146b-5p	mir-199a-1-5p	0.557537629	0.010645543	0.71127508	0.000437735
mir-146b-5p	mir-199a-2-5p	0.557537629	0.010645543	0.71127508	0.000437735
mir-148b-3p	norepi	0.382843069	0.095701471	0.141968227	0.550469096
mir-221-3p	let-7d-3p	0.912468676	2.08E-08	0.856968111	1.40E-06
mir-30d-5p	mir-151a-5p	0.495362633	0.026358247	0.66592	0.001349655
mir-320a	mir-128-1-3p	0.247533161	0.292703189	-0.007298874	0.975636386
mir-320a	mir-128-2-3p	0.247533161	0.292703189	-0.007298874	0.975636386
mir-107	mir-486-2-5p	0.944059537	4.16E-10	0.907660426	3.30E-08
mir-17-5p	mir-30c-1-5p	-0.007934794	0.973514373	0.24654818	0.294690163
mir-17-5p	mir-30c-2-5p	-0.007934794	0.973514373	0.24654818	0.294690163
let-7a-3-5p	mir-148b-3p	0.28702972	0.219820201	0.504098542	0.023429371
let-7a-2-5p	mir-148b-3p	0.28702972	0.219820201	0.504098542	0.023429371
let-7a-1-5p	mir-148b-3p	0.28702972	0.219820201	0.504098542	0.023429371
mir-222-3p	mir-543	-0.112109646	0.637944303	-0.355641535	0.123832377
let-7i-5p	copeptin	-0.001321989	0.995586599	-0.254821902	0.278256059
mir-361-5p	mir-378a-3p	-0.514615437	0.020252025	-0.300281089	0.198316755
mir-223-3p	mir-543	0.533211182	0.015479766	0.323447249	0.164197987
mir-30d-5p	mir-23a-3p	0.525899652	0.017234621	0.687530408	0.000808684
mir-106a-5p	mir-199a-1-5p	-0.503478004	0.023628506	-0.671049252	0.001199565
mir-106a-5p	mir-199a-2-5p	-0.503478004	0.023628506	-0.671049252	0.001199565
mir-10a-5p	mir-99a-5p	0.526461577	0.017094347	0.687787903	0.00080356
mir-151a-5p	mir-99b-5p	0.372794177	0.105486983	0.132653953	0.577171218
mir-101-1-3p	let-7d-5p	-0.289470224	0.215749691	-0.505037431	0.023130606
let-7d-5p	mir-101-2-3p	-0.289470224	0.215749691	-0.505037431	0.023130606
mir-19b-2-3p	mir-27b-3p	-0.149426855	0.529496698	-0.387075857	0.091784871
mir-27b-3p	mir-19b-1-3p	-0.149426855	0.529496698	-0.387075857	0.091784871
mir-192-5p	mir-143-3p	-0.237332307	0.313679675	-0.461902478	0.04033592
mir-222-3p	cpeptide	-0.120030766	0.614219995	0.136272756	0.56673082
mir-340-5p	mir-15a-5p	-0.492482344	0.02738474	-0.662296229	0.001464954
mir-10b-5p	mir-423-3p	-0.352682635	0.127213292	-0.555224213	0.011044405
mir-320a	mir-378a-3p	0.766205393	8.18E-05	0.637520445	0.002496625
mir-106a-5p	mir-140-5p	0.413631953	0.069847705	0.181080586	0.444853634
mir-191-5p	mir-93-5p	-0.010171157	0.966053259	-0.260687978	0.266957009
mir-744-5p	let-7b-5p	-0.641476755	0.002299905	-0.76878071	7.48E-05
mir-340-5p	let-7g-5p	0.322420875	0.16561772	0.530604796	0.016088055
mir-191-5p	mir-375	-0.023715073	0.920946526	0.228725153	0.33206287
mir-361-5p	mir-486-2-5p	-0.570620293	0.00860391	-0.71871745	0.000356711
mir-17-5p	mir-16-2-5p	0.83615543	4.39E-06	0.740557655	0.000188285
mir-17-5p	mir-16-1-5p	0.83615543	4.39E-06	0.740557655	0.000188285
mir-192-5p	mir-148b-3p	0.311404909	0.181388647	0.06552801	0.783719162
mir-222-3p	mir-484	0.5820753	0.007089742	0.726795575	0.000283579
mir-25-3p	mir-143-3p	-0.270727058	0.248299099	-0.48837552	0.028902503
mir-7-3-5p	mir-199a-1-5p	-0.452058411	0.045382199	-0.631091477	0.002846142
mir-7-3-5p	mir-199a-2-5p	-0.452058411	0.045382199	-0.631091477	0.002846142
mir-199a-1-5p	mir-7-1-5p	-0.452058411	0.045382199	-0.631091477	0.002846142
mir-199a-1-5p	mir-7-2-5p	-0.452058411	0.045382199	-0.631091477	0.002846142
mir-7-1-5p	mir-199a-2-5p	-0.452058411	0.045382199	-0.631091477	0.002846142
mir-7-2-5p	mir-199a-2-5p	-0.452058411	0.045382199	-0.631091477	0.002846142
mir-425-5p	mir-378a-3p	0.47825744	0.032924478	0.650760145	0.001888573
mir-223-3p	mir-224-5p	0.439389209	0.05257557	0.621427875	0.003447485
mir-19b-2-3p	mir-584-5p	-0.238655058	0.310909917	-0.461377487	0.040593556
mir-584-5p	mir-19b-1-3p	-0.238655058	0.310909917	-0.461377487	0.040593556
mir-26a-2-5p	mir-425-5p	-0.119580312	0.615559573	0.134651814	0.571397065
mir-425-5p	mir-26a-1-5p	-0.119580312	0.615559573	0.134651814	0.571397065
mir-99a-5p	cpeptide	0.141734805	0.551131414	-0.112444939	0.636932966
mir-192-5p	mir-148a-3p	0.553250818	0.011394138	0.351924633	0.128089856
mir-106b-3p	mir-23a-3p	-0.410810688	0.071970604	-0.599156578	0.005242685
mir-191-5p	mir-23a-3p	0.681620119	0.000934131	0.795897234	2.71E-05
mir-182-5p	mir-99b-5p	-0.576916638	0.007742068	-0.722515636	0.000320543
mir-181b-2-5p	mir-148a-3p	-0.080229841	0.736690993	0.172784096	0.466328327

mir-181b-1-5p	mir-148a-3p	-0.080229841	0.736690993	0.172784096	0.466328327
mir-30d-5p	let-7b-5p	-0.046742249	0.844857394	-0.292786902	0.210297796
mir-374b-5p	mir-30a-5p	-0.010614839	0.96457332	0.239377754	0.309402888
mir-744-5p	mir-30a-5p	-0.030953044	0.896927661	0.220011083	0.351307249
mir-22-3p	mir-3615	0.652359607	0.001824347	0.481370013	0.03164331
mir-92a-2-3p	mir-532-5p	0.804202376	1.93E-05	0.694271728	0.000683279
mir-532-5p	mir-92a-1-3p	0.804202376	1.93E-05	0.694271728	0.000683279
mir-98-5p	mir-15b-5p	0.479756185	0.032302614	0.262386596	0.263739849
mir-27b-3p	mir-26b-5p	-0.309359454	0.184425262	-0.5180814	0.01928365
mir-361-5p	mir-20b-5p	-0.502067896	0.024086002	-0.667269189	0.001308724
let-7a-3-5p	mir-199a-1-5p	0.741227634	0.000184451	0.604081956	0.004790907
let-7a-3-5p	mir-199a-2-5p	0.741227634	0.000184451	0.604081956	0.004790907
mir-199a-1-5p	let-7a-2-5p	0.741227634	0.000184451	0.604081956	0.004790907
mir-199a-1-5p	let-7a-1-5p	0.741227634	0.000184451	0.604081956	0.004790907
let-7a-2-5p	mir-199a-2-5p	0.741227634	0.000184451	0.604081956	0.004790907
let-7a-1-5p	mir-199a-2-5p	0.741227634	0.000184451	0.604081956	0.004790907
mir-19b-2-3p	mir-224-5p	0.191437735	0.418786258	-0.059714119	0.802527657
mir-224-5p	mir-19b-1-3p	0.191437735	0.418786258	-0.059714119	0.802527657
mir-16-2-5p	mir-363-3p	0.929039786	3.34E-09	0.956651192	4.38E-11
mir-16-1-5p	mir-363-3p	0.929039786	3.34E-09	0.956651192	4.38E-11
mir-340-5p	mir-194-2-5p	-0.42012868	0.065137955	-0.192020714	0.417343891
mir-340-5p	mir-194-1-5p	-0.42012868	0.065137955	-0.192020714	0.417343891
mir-10b-5p	mir-128-1-3p	0.126050218	0.596433278	-0.125838157	0.597056235
mir-10b-5p	mir-128-2-3p	0.126050218	0.596433278	-0.125838157	0.597056235
mir-92a-2-3p	mir-20a-5p	0.464899827	0.038889151	0.245501375	0.296810884
mir-20a-5p	mir-92a-1-3p	0.464899827	0.038889151	0.245501375	0.296810884
mir-106a-5p	mir-15a-5p	0.822552406	8.53E-06	0.889051894	1.61E-07
mir-451a	mir-23a-3p	-0.330873717	0.15417444	-0.534526135	0.015179965
mir-125a-5p	mir-224-5p	0.329748301	0.15566542	0.533586563	0.015393701
mir-423-3p	mir-99b-5p	0.545819068	0.012792612	0.345277803	0.13596067
mir-1307-3p	mir-99a-5p	0.077285514	0.746043187	0.31825173	0.171471374
mir-451a	mir-221-3p	-0.475319996	0.034170401	-0.646425982	0.002072197
mir-30a-5p	let-7g-5p	-0.081374294	0.733065334	0.168936661	0.476462271
mir-222-3p	let-7g-5p	0.38790451	0.091032097	0.579292808	0.007435756
mir-29a-3p	mir-185-5p	0.473452341	0.034981497	0.256588441	0.274822597
mir-3615	mir-28-5p	-0.231138506	0.326845523	-0.45198968	0.045419061
mir-29a-3p	mir-10b-5p	0.624518116	0.003244696	0.446798322	0.048270556
mir-194-2-5p	mir-30d-5p	-0.253058531	0.281709841	-0.007016639	0.976578232
mir-30d-5p	mir-194-1-5p	-0.253058531	0.281709841	-0.007016639	0.976578232
mir-1307-3p	mir-374b-5p	0.215766519	0.360909834	0.438752503	0.052958756
mir-340-5p	mir-425-5p	-0.043378342	0.85590649	0.205037909	0.385842216
mir-223-3p	cpeptide	-0.060963486	0.798476633	-0.302625882	0.194663799
mir-140-5p	mir-151a-5p	0.281432805	0.22934435	0.49342453	0.027045553
mir-361-5p	mir-27a-3p	-0.139627501	0.557126874	0.110299597	0.643414509
mir-148b-3p	let-7g-5p	0.515039477	0.020131513	0.675312122	0.001085762
mir-29a-3p	mir-199a-1-5p	-0.119645079	0.615366894	0.129778931	0.585524273
mir-29a-3p	mir-199a-2-5p	-0.119645079	0.615366894	0.129778931	0.585524273
mir-19b-2-3p	mir-27a-3p	0.693831838	0.000690926	0.540326489	0.013912698
mir-19b-1-3p	mir-27a-3p	0.693831838	0.000690926	0.540326489	0.013912698
mir-101-1-3p	mir-425-5p	0.756971381	0.000111683	0.628265843	0.003012154
mir-425-5p	mir-101-2-3p	0.756971381	0.000111683	0.628265843	0.003012154
mir-26b-5p	mir-30c-1-5p	-0.365638889	0.11288329	-0.132112516	0.578740374
mir-26b-5p	mir-30c-2-5p	-0.365638889	0.11288329	-0.132112516	0.578740374
mir-423-3p	mir-93-5p	-0.279171684	0.23326694	-0.490888522	0.027966132
mir-532-5p	mir-125a-5p	-0.8056673	1.81E-05	-0.698481828	0.000613645
mir-221-3p	mir-93-5p	-0.296272008	0.204668016	-0.504653482	0.023252416
mir-199a-2-3p	mir-320a	-0.162837096	0.492751128	-0.392156845	0.087240315
mir-320a	mir-199a-1-3p	-0.162837096	0.492751128	-0.392156845	0.087240315
mir-320a	mir-199b-3p	-0.162837096	0.492751128	-0.392156845	0.087240315
mir-20a-5p	mir-99a-5p	0.306137257	0.189278221	0.06615908	0.781684326
mir-126-5p	mir-99a-5p	-0.03738153	0.875666543	0.209386487	0.375623007
mir-10b-5p	mir-221-3p	0.164512399	0.488250275	-0.083575666	0.726106669
mir-29a-3p	mir-363-3p	0.416575854	0.067682929	0.191480166	0.418681189
mir-191-5p	mir-10a-5p	0.247421069	0.292928893	0.463890561	0.039371739

mir-146b-5p	mir-486-2-5p	-0.555439894	0.011006714	-0.704261382	0.000527917
mir-17-5p	mir-144-3p	0.769086798	7.40E-05	0.646303477	0.002077596
mir-192-5p	mir-151a-3p	0.118873781	0.617663043	-0.128942561	0.587963841
mir-17-5p	mir-451a	0.729025979	0.000265803	0.590003201	0.00617549
mir-223-3p	mir-93-5p	-0.012229247	0.959189298	-0.255401356	0.277126902
mir-15a-5p	mir-27a-3p	0.352571371	0.127341691	0.118871732	0.617669148
mir-29a-3p	mir-191-5p	0.054287662	0.820177824	0.294165539	0.208058664
mir-106a-5p	mir-30d-5p	-0.017488726	0.941661995	-0.260148306	0.267984279
let-7a-3-5p	mir-363-3p	-0.348178929	0.132484452	-0.545405216	0.012874385
let-7a-2-5p	mir-363-3p	-0.348178929	0.132484452	-0.545405216	0.012874385
let-7a-1-5p	mir-363-3p	-0.348178929	0.132484452	-0.545405216	0.012874385
mir-125a-5p	mir-374b-5p	0.647993966	0.002004116	0.480460693	0.03201349
mir-744-5p	mir-106b-3p	-0.60450014	0.004754079	-0.739031715	0.000197272
mir-25-3p	mir-30d-5p	0.045008476	0.850548797	-0.200299202	0.39715276
mir-16-2-5p	mir-28-5p	-0.113671029	0.633240015	-0.347092815	0.133778421
mir-16-1-5p	mir-28-5p	-0.113671029	0.633240015	-0.347092815	0.133778421
mir-181b-2-5p	mir-423-3p	-0.142744415	0.548269304	0.103609465	0.663784839
mir-423-3p	mir-181b-1-5p	-0.142744415	0.548269304	0.103609465	0.663784839
mir-22-3p	mir-423-5p	0.756905381	0.000111927	0.629960777	0.002911634
mir-151a-3p	mir-107	-0.033917737	0.887113173	-0.274295634	0.24187302
mir-122-5p	mir-126-5p	-0.190145583	0.421992749	0.054987352	0.817897138
mir-423-3p	mir-194-2-5p	-0.592697164	0.005887819	-0.408908888	0.073428625
mir-423-3p	mir-194-1-5p	-0.592697164	0.005887819	-0.408908888	0.073428625
mir-16-2-5p	mir-223-3p	-0.099254451	0.677169317	-0.333737192	0.150425608
mir-223-3p	mir-16-1-5p	-0.099254451	0.677169317	-0.333737192	0.150425608
let-7f-2-5p	mir-92a-2-3p	-0.725941134	0.000290653	-0.823463709	8.17E-06
let-7f-2-5p	mir-92a-1-3p	-0.725941134	0.000290653	-0.823463709	8.17E-06
mir-92a-2-3p	let-7f-1-5p	-0.725941134	0.000290653	-0.823463709	8.17E-06
let-7f-1-5p	mir-92a-1-3p	-0.725941134	0.000290653	-0.823463709	8.17E-06
mir-194-2-5p	mir-148a-3p	0.471950994	0.035644325	0.259557599	0.269111535
mir-148a-3p	mir-194-1-5p	0.471950994	0.035644325	0.259557599	0.269111535
mir-125b-1-5p	mir-30a-5p	0.464032992	0.039303355	0.634742171	0.002642997
mir-30a-5p	mir-125b-2-5p	0.464032992	0.039303355	0.634742171	0.002642997
mir-150-5p	mir-363-3p	0.172836549	0.466190929	0.398098214	0.082139082
mir-423-3p	mir-425-5p	-0.167462337	0.480374549	0.077537398	0.745241757
mir-126-5p	copeptin	-0.141210485	0.552620435	-0.370402594	0.107918904
mir-122-5p	mir-28-3p	0.10155795	0.67007795	0.334905637	0.148914287
mir-125a-5p	let-7b-5p	-0.732447782	0.000240383	-0.59665553	0.005485189
mir-24-2-3p	let-7g-5p	0.084637887	0.722756262	0.319234854	0.170078459
let-7g-5p	mir-24-1-3p	0.084637887	0.722756262	0.319234854	0.170078459
mir-26a-2-5p	mir-199a-2-3p	0.932491114	2.16E-09	0.892095104	1.27E-07
mir-26a-2-5p	mir-199a-1-3p	0.932491114	2.16E-09	0.892095104	1.27E-07
mir-26a-2-5p	mir-199b-3p	0.932491114	2.16E-09	0.892095104	1.27E-07
mir-199a-2-3p	mir-26a-1-5p	0.932491114	2.16E-09	0.892095104	1.27E-07
mir-199a-1-3p	mir-26a-1-5p	0.932491114	2.16E-09	0.892095104	1.27E-07
mir-199b-3p	mir-26a-1-5p	0.932491114	2.16E-09	0.892095104	1.27E-07
mir-375	copeptin	-0.117078514	0.623020747	-0.347548873	0.133234002
mir-15a-5p	mir-378a-3p	0.639665687	0.002388287	0.472065948	0.035593231
mir-126-5p	let-7d-3p	0.540850918	0.013802458	0.691160385	0.00073894
mir-192-5p	mir-744-5p	-0.611132867	0.004200484	-0.434940875	0.055297413
mir-101-1-3p	mir-584-5p	-0.319481456	0.169730286	-0.519610734	0.018868279
mir-584-5p	mir-101-2-3p	-0.319481456	0.169730286	-0.519610734	0.018868279
mir-146a-5p	mir-139-5p	0.750801521	0.000136535	0.839463087	3.70E-06
mir-224-5p	mir-199a-2-3p	0.586242326	0.006596305	0.724181131	0.000305699
mir-224-5p	mir-199a-1-3p	0.586242326	0.006596305	0.724181131	0.000305699
mir-224-5p	mir-199b-3p	0.586242326	0.006596305	0.724181131	0.000305699
mir-98-5p	mir-27a-3p	0.052619613	0.825620522	0.288658323	0.217098314
mir-744-5p	mir-451a	-0.579089529	0.007461566	-0.718886179	0.000355034
mir-1307-3p	let-7d-3p	0.431659942	0.057372682	0.608253444	0.00443387
mir-106a-5p	mir-484	0.647935659	0.002006614	0.483691882	0.03071328
mir-27b-3p	mir-128-1-3p	0.611291404	0.004187936	0.741972054	0.00018027
mir-27b-3p	mir-128-2-3p	0.611291404	0.004187936	0.741972054	0.00018027
mir-151a-5p	mir-186-5p	-0.167283958	0.480848979	0.074771177	0.754056797
mir-16-2-5p	mir-27a-3p	0.649467614	0.001941848	0.485869502	0.029860629

mir-16-1-5p	mir-27a-3p	0.649467614	0.001941848	0.485869502	0.029860629
mir-223-3p	let-7g-5p	0.217672084	0.356580375	0.434104721	0.05582079
mir-181a-2-5p	mir-423-3p	0.594359405	0.005715881	0.729639895	0.000261081
mir-423-3p	mir-181a-1-5p	0.594359405	0.005715881	0.729639895	0.000261081
mir-98-5p	mir-20a-5p	-0.002271162	0.992417898	-0.240899715	0.306243611
mir-584-5p	mir-107	-0.181676983	0.443330073	-0.402846253	0.07822396
mir-10b-5p	mir-375	0.648410419	0.001986352	0.768148386	7.64E-05
mir-151a-3p	mir-99a-5p	0.280463368	0.231020842	0.486412361	0.029650992
mir-16-2-5p	mir-30c-1-5p	-0.42692775	0.060469386	-0.21008238	0.374001964
mir-16-2-5p	mir-30c-2-5p	-0.42692775	0.060469386	-0.21008238	0.374001964
mir-30c-1-5p	mir-16-1-5p	-0.42692775	0.060469386	-0.21008238	0.374001964
mir-16-1-5p	mir-30c-2-5p	-0.42692775	0.060469386	-0.21008238	0.374001964
let-7b-5p	let-7g-5p	0.492841131	0.027255184	0.288526214	0.217318277
mir-151a-3p	mir-378a-3p	0.118679932	0.618240668	-0.122633902	0.606501818
mir-146a-5p	mir-224-5p	0.561257086	0.010028821	0.704981203	0.00051799
mir-148a-3p	mir-484	0.004368056	0.985418117	0.241914142	0.304148763
mir-30e-5p	mir-185-5p	0.916146307	1.43E-08	0.867304579	7.41E-07
mir-19b-2-3p	copeptin	0.095671647	0.688251023	-0.145369988	0.540857735
mir-19b-1-3p	copeptin	0.095671647	0.688251023	-0.145369988	0.540857735
mir-30c-1-5p	mir-28-3p	0.467045168	0.037878586	0.634253799	0.002669451
mir-30c-2-5p	mir-28-3p	0.467045168	0.037878586	0.634253799	0.002669451
mir-144-3p	mir-21-5p	-0.306365888	0.188931074	-0.507046024	0.022501587
mir-30a-5p	norepi	-0.253503179	0.280836448	-0.01710275	0.942947483
mir-3615	mir-21-5p	-0.527018313	0.016956272	-0.67935053	0.000986483
let-7i-5p	mir-10b-5p	-0.256610991	0.274778942	-0.020640486	0.931170562
mir-361-5p	mir-425-5p	-0.24922339	0.28931274	-0.013080874	0.956349807
mir-222-3p	mir-486-2-5p	0.740981116	0.000185854	0.831831797	5.45E-06
mir-320a	mir-23a-3p	-0.189888013	0.422633481	-0.408279516	0.073915959
mir-151a-3p	copeptin	-0.078066253	0.743559898	-0.309059383	0.18487361
let-7a-3-5p	mir-106b-3p	-0.385826774	0.092928193	-0.570434128	0.008630535
let-7a-2-5p	mir-106b-3p	-0.385826774	0.092928193	-0.570434128	0.008630535
let-7a-1-5p	mir-106b-3p	-0.385826774	0.092928193	-0.570434128	0.008630535
mir-10b-5p	mir-224-5p	0.350523083	0.129721908	0.124425926	0.601211711
mir-543	mir-375	-0.056489837	0.813004464	0.182272501	0.441811479
mir-17-5p	mir-15b-5p	0.780976743	4.82E-05	0.858674015	1.26E-06
mir-144-3p	mir-340-5p	-0.138124991	0.561419286	-0.362054823	0.116725196
mir-361-5p	mir-221-3p	0.89249983	1.23E-07	0.932112062	2.27E-09
mir-101-1-3p	mir-224-5p	0.129212516	0.587175954	-0.109770693	0.645016281
mir-224-5p	mir-101-2-3p	0.129212516	0.587175954	-0.109770693	0.645016281
mir-223-3p	mir-320a	-0.099286486	0.677070517	-0.327264801	0.15899092
mir-25-3p	cpeptide	-0.17032763	0.472785867	0.067949354	0.775919193
mir-16-2-5p	mir-374b-5p	0.04343355	0.855724939	-0.194060284	0.412318833
mir-374b-5p	mir-16-1-5p	0.04343355	0.855724939	-0.194060284	0.412318833
mir-182-5p	cpeptide	-0.062869235	0.79230689	0.175212711	0.459988282
mir-151a-5p	mir-15b-5p	0.278273332	0.234837388	0.482142878	0.031331322
mir-224-5p	mir-486-2-5p	-0.004894593	0.98366059	-0.239882617	0.308352724
mir-199a-1-5p	mir-425-5p	-0.245108406	0.2976094	-0.010464766	0.965073888
mir-425-5p	mir-199a-2-5p	-0.245108406	0.2976094	-0.010464766	0.965073888
mir-29a-3p	mir-128-1-3p	0.23142294	0.326233838	0.442543092	0.050708603
mir-29a-3p	mir-128-2-3p	0.23142294	0.326233838	0.442543092	0.050708603
mir-99b-5p	mir-30e-5p	-0.436571581	0.05428745	-0.224714957	0.340840345
mir-486-2-5p	mir-99b-5p	-0.578979095	0.007475619	-0.398379918	0.081902825
mir-1307-3p	mir-30a-5p	0.319912922	0.169122283	0.092213239	0.699006327
mir-125a-5p	mir-107	-0.573682955	0.008175416	-0.391808042	0.087546883
mir-423-3p	mir-30c-1-5p	0.783788177	4.34E-05	0.67292555	0.001148296
mir-423-3p	mir-30c-2-5p	0.783788177	4.34E-05	0.67292555	0.001148296
mir-222-3p	mir-20b-5p	0.713645502	0.000410385	0.812241375	1.36E-05
mir-140-5p	mir-423-5p	0.740253439	0.000190048	0.611956322	0.004135642
mir-92a-2-3p	mir-320a	0.497339184	0.02567157	0.297518059	0.202679708
mir-320a	mir-92a-1-3p	0.497339184	0.02567157	0.297518059	0.202679708
mir-224-5p	mir-221-3p	0.548044226	0.012360101	0.693424309	0.000698075
mir-1307-3p	mir-484	-0.50637862	0.02270907	-0.308559081	0.18562277
mir-98-5p	copeptin	-0.306335864	0.188976638	-0.077504365	0.745346846
mir-26a-2-5p	mir-30a-5p	0.054950799	0.818016251	0.285523439	0.22235748

mir-30a-5p	mir-26a-1-5p	0.054950799	0.818016251	0.285523439	0.22235748
let-7a-3-5p	mir-486-2-5p	-0.305267183	0.190603243	-0.503487503	0.023625448
let-7a-2-5p	mir-486-2-5p	-0.305267183	0.190603243	-0.503487503	0.023625448
let-7a-1-5p	mir-486-2-5p	-0.305267183	0.190603243	-0.503487503	0.023625448
mir-103a-2-3p	mir-140-5p	0.769406132	7.32E-05	0.652849776	0.001805034
mir-140-5p	mir-103a-1-3p	0.769406132	7.32E-05	0.652849776	0.001805034
mir-143-3p	norepi	-0.377518117	0.100800471	-0.15739893	0.507499973
let-7f-2-5p	mir-199a-2-3p	0.85237679	1.83E-06	0.905716064	3.95E-08
let-7f-2-5p	mir-199a-1-3p	0.85237679	1.83E-06	0.905716064	3.95E-08
let-7f-2-5p	mir-199b-3p	0.85237679	1.83E-06	0.905716064	3.95E-08
mir-199a-2-3p	let-7f-1-5p	0.85237679	1.83E-06	0.905716064	3.95E-08
mir-199a-1-3p	let-7f-1-5p	0.85237679	1.83E-06	0.905716064	3.95E-08
let-7f-1-5p	mir-199b-3p	0.85237679	1.83E-06	0.905716064	3.95E-08
let-7i-5p	mir-532-5p	0.245431973	0.296951813	0.012647309	0.957795336
mir-26a-2-5p	let-7b-5p	-0.462898733	0.039850498	-0.628430361	0.003002272
let-7b-5p	mir-26a-1-5p	-0.462898733	0.039850498	-0.628430361	0.003002272
mir-98-5p	mir-186-5p	-0.019935492	0.933516423	-0.252120202	0.283558458
mir-140-5p	mir-451a	0.432030304	0.057135503	0.221247864	0.348537358
mir-340-5p	mir-10a-5p	0.191211066	0.41934779	0.406147386	0.07558486
mir-23a-3p	mir-363-3p	-0.367458877	0.110967607	-0.552911565	0.011455156
mir-223-3p	mir-15b-5p	0.38576548	0.092984565	0.567531385	0.009054451
mir-361-5p	mir-192-5p	-0.531141581	0.015961236	-0.340543443	0.141770824
mir-140-5p	mir-363-3p	0.470838863	0.036141591	0.267505617	0.254192902
mir-361-5p	mir-25-3p	-0.485840923	0.029871697	-0.645509194	0.002112891
let-7f-2-5p	let-7i-5p	0.533918442	0.01531793	0.681790098	0.000930307
let-7i-5p	let-7f-1-5p	0.533918442	0.01531793	0.681790098	0.000930307
mir-7-3-5p	mir-320a	0.91748287	1.24E-08	0.870734234	5.93E-07
mir-7-1-5p	mir-320a	0.91748287	1.24E-08	0.870734234	5.93E-07
mir-320a	mir-7-2-5p	0.91748287	1.24E-08	0.870734234	5.93E-07
mir-1307-3p	mir-486-2-5p	-0.691304619	0.000736277	-0.795836562	2.72E-05
mir-1307-3p	mir-199a-1-5p	0.504375054	0.023341066	0.659429721	0.001561919
mir-1307-3p	mir-199a-2-5p	0.504375054	0.023341066	0.659429721	0.001561919
mir-101-1-3p	mir-128-1-3p	0.194976715	0.41007167	-0.039007264	0.870302103
mir-101-1-3p	mir-128-2-3p	0.194976715	0.41007167	-0.039007264	0.870302103
mir-128-1-3p	mir-101-2-3p	0.194976715	0.41007167	-0.039007264	0.870302103
mir-128-2-3p	mir-101-2-3p	0.194976715	0.41007167	-0.039007264	0.870302103
mir-150-5p	norepi	-0.41477337	0.069002299	-0.202023389	0.39301643
mir-451a	cpeptide	-0.08677198	0.716039729	0.148329306	0.532559345
mir-375	mir-23a-3p	0.527530395	0.01683006	0.336698486	0.146615974
mir-27b-3p	mir-185-5p	-0.185351053	0.434004619	-0.400144954	0.080433985
mir-199a-1-5p	mir-30e-5p	-0.256213634	0.275548858	-0.025867375	0.913796405
mir-30e-5p	mir-199a-2-5p	-0.256213634	0.275548858	-0.025867375	0.913796405
mir-320a	mir-484	0.809202032	1.56E-05	0.710749262	0.000444008
mir-3615	let-7g-5p	0.385140011	0.093561238	0.168554916	0.477473739
mir-26b-5p	mir-15a-5p	0.886782542	1.91E-07	0.824599089	7.75E-06
mir-26b-5p	mir-374b-5p	0.095098113	0.690030741	-0.139235358	0.558245752
mir-106a-5p	let-7b-5p	0.924335662	5.85E-09	0.952072295	1.06E-10
mir-192-5p	mir-423-5p	0.613720213	0.00399953	0.445896652	0.048779506
mir-374b-5p	mir-23a-3p	0.563385801	0.009689133	0.702669395	0.000550448
norepi	cpeptide	-0.391831695	0.087526068	-0.177562376	0.453896572
let-7f-2-5p	mir-99b-5p	0.808621514	1.60E-05	0.71069181	0.000444698
mir-99b-5p	let-7f-1-5p	0.808621514	1.60E-05	0.71069181	0.000444698
mir-425-5p	mir-148a-3p	0.032007257	0.893436008	0.260027537	0.268214503
mir-744-5p	mir-425-5p	-0.357315524	0.121948256	-0.138808625	0.55946445
mir-126-3p	mir-128-1-3p	0.781174332	4.78E-05	0.857130212	1.39E-06
mir-126-3p	mir-128-2-3p	0.781174332	4.78E-05	0.857130212	1.39E-06
mir-16-2-5p	mir-181a-2-5p	0.089282098	0.708165406	-0.143421818	0.546352723
mir-16-2-5p	mir-181a-1-5p	0.089282098	0.708165406	-0.143421818	0.546352723
mir-181a-2-5p	mir-16-1-5p	0.089282098	0.708165406	-0.143421818	0.546352723
mir-16-1-5p	mir-181a-1-5p	0.089282098	0.708165406	-0.143421818	0.546352723
mir-103a-2-3p	norepi	0.586549834	0.006561038	0.412744292	0.070510511
mir-103a-1-3p	norepi	0.586549834	0.006561038	0.412744292	0.070510511
mir-20a-5p	copeptin	0.017053741	0.943110718	-0.212877062	0.367531859
mir-139-5p	mir-363-3p	-0.200750948	0.396066708	-0.410901196	0.07190176

mir-27b-3p	mir-425-5p	-0.209468902	0.37543082	0.020537588	0.931512919
mir-1307-3p	mir-363-3p	-0.704937742	0.000518585	-0.804082954	1.94E-05
mir-144-3p	mir-3615	0.871362748	5.69E-07	0.802550644	2.06E-05
mir-3615	mir-199a-1-5p	-0.59891082	0.005266115	-0.727979372	0.000274022
mir-3615	mir-199a-2-5p	-0.59891082	0.005266115	-0.727979372	0.000274022
mir-15a-5p	cpeptide	-0.057863999	0.808535491	0.173250882	0.465106324
mir-7-3-5p	mir-23a-3p	-0.314450842	0.176929878	-0.506801248	0.022577508
mir-7-1-5p	mir-23a-3p	-0.314450842	0.176929878	-0.506801248	0.022577508
mir-7-2-5p	mir-23a-3p	-0.314450842	0.176929878	-0.506801248	0.022577508
mir-22-3p	mir-99b-5p	-0.523236252	0.017912072	-0.334555693	0.149365806
mir-150-5p	cpeptide	0.37522895	0.10305219	0.160362347	0.499436737
let-7f-2-5p	mir-25-3p	-0.355026336	0.124529986	-0.53978911	0.014026396
mir-25-3p	let-7f-1-5p	-0.355026336	0.124529986	-0.53978911	0.014026396
let-7f-2-5p	mir-3615	-0.542587767	0.013442368	-0.686045094	0.000838784
mir-3615	let-7f-1-5p	-0.542587767	0.013442368	-0.686045094	0.000838784
mir-7-3-5p	mir-423-3p	-0.448017012	0.047589147	-0.613632276	0.004006227
mir-423-3p	mir-7-1-5p	-0.448017012	0.047589147	-0.613632276	0.004006227
mir-423-3p	mir-7-2-5p	-0.448017012	0.047589147	-0.613632276	0.004006227
mir-629-5p	let-7b-5p	0.937129404	1.16E-09	0.90175114	5.64E-08
mir-363-3p	cpeptide	-0.092666986	0.697592018	0.13851118	0.560314616
mir-199a-1-5p	mir-375	-0.07541262	0.752010076	-0.298515935	0.201096712
mir-375	mir-199a-2-5p	-0.07541262	0.752010076	-0.298515935	0.201096712
mir-378a-3p	mir-185-5p	0.768727623	7.49E-05	0.655515054	0.001702995
mir-19b-2-3p	mir-101-1-3p	0.928916335	3.39E-09	0.954724508	6.44E-11
mir-19b-2-3p	mir-101-2-3p	0.928916335	3.39E-09	0.954724508	6.44E-11
mir-101-1-3p	mir-19b-1-3p	0.928916335	3.39E-09	0.954724508	6.44E-11
mir-19b-1-3p	mir-101-2-3p	0.928916335	3.39E-09	0.954724508	6.44E-11
mir-194-2-5p	mir-139-5p	-0.277620359	0.235983161	-0.053066503	0.824161597
mir-194-1-5p	mir-139-5p	-0.277620359	0.235983161	-0.053066503	0.824161597
mir-24-2-3p	mir-30c-1-5p	0.786016955	3.99E-05	0.679935971	0.000972747
mir-24-2-3p	mir-30c-2-5p	0.786016955	3.99E-05	0.679935971	0.000972747
mir-30c-1-5p	mir-24-1-3p	0.786016955	3.99E-05	0.679935971	0.000972747
mir-24-1-3p	mir-30c-2-5p	0.786016955	3.99E-05	0.679935971	0.000972747
mir-3615	mir-30d-5p	-0.179714449	0.448353844	-0.391523425	0.087797625
mir-423-5p	let-7b-5p	0.662557095	0.001456387	0.512122528	0.020972171

Col 1 and 2. Col 3 and 4, magnitude and significance of correlation at 14degrees; col 5
and magnitude of correlations; col 9-11, local FDR estimates at 14 degrees, RT and

p (difference)	(r1-r2)	lfdr (in cond. 1)	lfdr (in cond. 2)	lfdr (difference)
0.000118006	-0.445717786	0.134257478	6.53E-09	0.423840643
0.000118006	-0.445717786	0.134257478	6.53E-09	0.423840643
0.000311075	-0.342237534	0.035148121	3.90E-09	0.741428628
0.000311075	-0.342237534	0.035148121	3.90E-09	0.741428628
0.000311075	-0.342237534	0.035148121	3.90E-09	0.741428628
0.000489904	-0.484369549	0.205025676	9.79E-08	0.741428628
0.00070924	0.140726698	2.11E-11	4.69E-05	0.741428628
0.000899487	-0.760668676	1	6.41E-05	0.741428628
0.001186775	-0.2060563	0.001207877	5.82E-10	0.741428628
0.001243181	-0.340391104	0.03951775	1.63E-08	0.741428628
0.001243181	-0.340391104	0.03951775	1.63E-08	0.741428628
0.001287518	-0.474253276	0.241457119	2.98E-07	0.741428628
0.001421453	-0.370820852	0.073031096	5.53E-08	0.741428628
0.00146212	-0.73400065	1	0.000115129	0.741428628
0.001552749	-0.359839449	0.043105813	5.53E-08	0.741428628
0.001723805	0.932136584	0.584854032	0.011906673	0.741428628
0.001723805	0.932136584	0.584854032	0.011906673	0.741428628
0.001887178	-0.418112268	0.146615922	2.98E-07	0.846566334
0.001887178	-0.418112268	0.146615922	2.98E-07	0.846566334
0.00283729	-0.445993259	0.205025676	8.55E-07	0.846566334
0.00283729	-0.445993259	0.205025676	8.55E-07	0.846566334
0.002852146	-0.486015965	0.307400178	3.06E-06	0.846566334
0.003422478	-0.436813783	0.19926681	8.55E-07	0.846566334
0.003601256	-0.276711722	0.018163994	1.63E-08	0.846566334
0.003601256	-0.276711722	0.018163994	1.63E-08	0.846566334
0.003742635	-0.292705999	0.025193624	4.93E-08	0.846566334
0.004167558	-0.90719948	0.412603306	0.10273905	0.846566334
0.004759743	-0.782552167	1	0.003736177	0.846566334
0.005229788	-0.88329352	0.095538718	0.397942841	0.846566334
0.005229788	-0.88329352	0.095538718	0.397942841	0.846566334
0.005313955	-0.888956996	0.307400178	0.145037597	0.846566334
0.005339237	-0.888841986	0.295435331	0.145037597	0.846566334
0.005339237	-0.888841986	0.295435331	0.145037597	0.846566334
0.005424989	-0.410498941	0.169239932	8.55E-07	0.846566334
0.005424989	-0.410498941	0.169239932	8.55E-07	0.846566334
0.005459295	-0.434063142	0.205025676	3.06E-06	0.846566334
0.005762655	0.097653448	1.75E-10	4.11E-06	0.846566334
0.005762655	0.097653448	1.75E-10	4.11E-06	0.846566334
0.00582768	-0.754089043	1	0.002721298	0.846566334
0.005934671	-0.371067933	0.095006685	5.53E-07	0.846566334
0.006240705	-0.85045786	0.584854032	0.049298372	0.846566334
0.006267425	0.711051115	1	0.001791344	0.846566334
0.006648292	0.598074371	0.737584497	0.000124759	0.846566334
0.007268796	-0.81893321	0.737584497	0.025431136	0.846566334
0.007268796	-0.81893321	0.737584497	0.025431136	0.846566334
0.007371932	-0.545393543	0.584854032	4.69E-05	0.846566334
0.007449132	0.527292497	0.439859057	4.69E-05	0.846566334
0.007449132	0.527292497	0.439859057	4.69E-05	0.846566334
0.007449132	0.527292497	0.439859057	4.69E-05	0.846566334
0.007463437	-0.847874581	0.439859057	0.085309726	0.846566334
0.007463437	-0.847874581	0.439859057	0.085309726	0.846566334
0.007673366	0.855456405	0.295435331	0.242167012	0.846566334
0.007762788	0.796044823	1	0.02341474	0.846566334
0.007762788	0.796044823	1	0.02341474	0.846566334
0.008234545	0.570442161	0.584854032	0.000115129	0.846566334
0.008234545	0.570442161	0.584854032	0.000115129	0.846566334
0.008234545	0.570442161	0.584854032	0.000115129	0.846566334
0.008234545	0.570442161	0.584854032	0.000115129	0.846566334
0.008431408	-0.815418662	0.737584497	0.042826979	0.846566334

0.008431408	-0.815418662	0.737584497	0.042826979	0.846566334
0.008431408	-0.815418662	0.737584497	0.042826979	0.846566334
0.008431408	-0.815418662	0.737584497	0.042826979	0.846566334
0.008431408	-0.815418662	0.737584497	0.042826979	0.846566334
0.008431408	-0.815418662	0.737584497	0.042826979	0.846566334
0.00858711	-0.824900677	0.584854032	0.060775899	0.846566334
0.00858711	-0.824900677	0.584854032	0.060775899	0.846566334
0.00858711	-0.824900677	0.584854032	0.060775899	0.846566334
0.008619669	-0.183019104	0.0008771	4.73E-09	0.846566334
0.008978131	-0.234015587	0.008580492	2.24E-08	0.846566334
0.008978131	-0.234015587	0.008580492	2.24E-08	0.846566334
0.009828255	-0.41874855	0.205025676	4.11E-06	0.846566334
0.009828255	-0.41874855	0.205025676	4.11E-06	0.846566334
0.009921286	0.278405684	9.36E-08	0.011906673	0.846566334
0.010009409	0.797531722	0.043105813	0.704162183	0.846566334
0.010033486	-0.352266952	0.075374611	8.55E-07	0.846566334
0.010496845	0.655505677	1	0.001791344	0.846566334
0.010615945	-0.654656587	1	0.001791344	0.846566334
0.010717826	-0.816407116	0.439859057	0.126792252	0.846566334
0.010807515	-0.81839852	0.439859057	0.145037597	0.846566334
0.010807515	-0.81839852	0.439859057	0.145037597	0.846566334
0.010920573	0.635308	1	0.001090387	0.846566334
0.011258021	-0.49970723	0.439859057	4.69E-05	0.846566334
0.011857537	-0.813232707	0.349433107	0.242167012	0.846566334
0.011857537	-0.813232707	0.349433107	0.242167012	0.846566334
0.012196624	-0.262945551	0.018163994	9.79E-08	0.846566334
0.012196624	-0.262945551	0.018163994	9.79E-08	0.846566334
0.012196624	-0.262945551	0.018163994	9.79E-08	0.846566334
0.012369	-0.382275377	0.146615922	3.66E-06	0.846566334
0.012706877	-0.803540434	0.205025676	0.397942841	0.846566334
0.012829923	0.779259297	0.075374611	0.704162183	0.846566334
0.012843621	0.454641905	0.412603306	4.35E-05	0.846566334
0.013019784	-0.803444612	0.307400178	0.272770942	0.846566334
0.013101687	-0.392500424	0.169239932	4.11E-06	0.846566334
0.013101687	-0.392500424	0.169239932	4.11E-06	0.846566334
0.013150672	-0.643590555	1	0.002093839	0.846566334
0.013155434	-0.772336511	0.737584497	0.053084396	0.846566334
0.013155434	-0.772336511	0.737584497	0.053084396	0.846566334
0.013324875	-0.79953261	0.439859057	0.164911511	0.846566334
0.013474815	0.285867041	4.10E-07	0.02341474	0.846566334
0.013533129	-0.776703107	0.737584497	0.061934943	0.846566334
0.01355276	-0.128064457	8.53E-05	5.82E-10	0.846566334
0.01355276	-0.128064457	8.53E-05	5.82E-10	0.846566334
0.013775665	-0.39295075	0.169239932	5.99E-06	0.846566334
0.013775665	-0.39295075	0.169239932	5.99E-06	0.846566334
0.013775665	-0.39295075	0.169239932	5.99E-06	0.846566334
0.013775665	-0.39295075	0.169239932	5.99E-06	0.846566334
0.013915868	0.584484868	0.881481882	0.000596242	0.846566334
0.014203875	-0.514630052	0.439859057	0.000124759	0.846566334
0.014203875	-0.514630052	0.439859057	0.000124759	0.846566334
0.014255918	0.509165931	0.439859057	0.000115129	0.846566334
0.014255918	0.509165931	0.439859057	0.000115129	0.846566334
0.014255918	0.509165931	0.439859057	0.000115129	0.846566334
0.014261904	-0.305870095	0.03951775	8.55E-07	0.846566334
0.014261904	-0.305870095	0.03951775	8.55E-07	0.846566334
0.014261904	-0.305870095	0.03951775	8.55E-07	0.846566334
0.014355842	-0.404362879	0.19926681	9.47E-06	0.868819888
0.014841561	-0.404145982	0.205025676	9.47E-06	0.868819888
0.015024935	0.739575436	1	0.031639242	0.868819888
0.015251514	-0.768749812	0.691111318	0.085309726	0.868819888
0.015254782	-0.321612999	0.043105813	8.55E-07	0.868819888
0.015539775	0.769506385	0.104319013	0.704162183	0.868819888
0.015697335	-0.387764887	0.169239932	9.47E-06	0.868819888
0.015913062	-0.782729171	0.412603306	0.242167012	0.868819888

0.016364676	-0.772525511	0.584854032	0.145037597	0.868819888
0.016466585	0.170992008	7.89E-09	0.000596242	0.868819888
0.016628853	-0.569866037	0.737584497	0.000997588	0.868819888
0.016628853	-0.569866037	0.737584497	0.000997588	0.868819888
0.016720575	0.724975607	1	0.031639242	0.868819888
0.016720575	0.724975607	1	0.031639242	0.868819888
0.01689805	0.775311757	0.307400178	0.397942841	0.868819888
0.016978524	-0.772242921	0.439859057	0.145037597	0.868819888
0.016978524	-0.772242921	0.439859057	0.145037597	0.868819888
0.01700799	0.766271552	0.584854032	0.126792252	0.868819888
0.017287876	0.31579422	0.043105813	8.55E-07	0.868819888
0.017287876	0.31579422	0.043105813	8.55E-07	0.868819888
0.017400436	-0.262173682	0.024201521	2.98E-07	0.868819888
0.017400436	-0.262173682	0.024201521	2.98E-07	0.868819888
0.017400436	-0.262173682	0.024201521	2.98E-07	0.868819888
0.017465743	-0.582604857	0.989933981	0.001090387	0.868819888
0.017532251	-0.401966421	0.205025676	1.04E-05	0.868819888
0.018484948	0.559386805	0.737584497	0.000997588	0.868819888
0.018728452	0.279678391	1.09E-06	0.02341474	0.868819888
0.018983194	0.698356747	1	0.025431136	0.868819888
0.018983194	0.698356747	1	0.025431136	0.868819888
0.019171356	-0.335563212	0.075374611	3.16E-06	0.868819888
0.019488065	0.350486672	0.095538718	4.11E-06	0.868819888
0.019488732	0.434667065	6.77E-05	0.242167012	0.868819888
0.019488732	0.434667065	6.77E-05	0.242167012	0.868819888
0.019771113	0.755914214	0.439859057	0.164911511	0.868819888
0.019850037	0.758955305	0.412603306	0.305102445	0.868819888
0.019966005	0.279317251	0.034012327	5.53E-07	0.868819888
0.019966005	0.279317251	0.034012327	5.53E-07	0.868819888
0.020227496	-0.676579209	1	0.017403168	0.868819888
0.020432166	-0.428802398	0.307400178	4.69E-05	0.868819888
0.020511833	0.250450718	3.42E-07	0.009270366	0.868819888
0.020511833	0.250450718	3.42E-07	0.009270366	0.868819888
0.020511833	0.250450718	3.42E-07	0.009270366	0.868819888
0.020820864	-0.714254735	0.073031096	1	0.868819888
0.020867837	-0.322449243	0.070946263	3.06E-06	0.868819888
0.021335678	-0.213444988	0.004526275	9.27E-08	0.868819888
0.021704517	0.733539686	0.737584497	0.126792252	0.868819888
0.022412281	0.628688531	0.016023223	1	0.868819888
0.022412281	0.628688531	0.016023223	1	0.868819888
0.022412281	0.628688531	0.016023223	1	0.868819888
0.022412281	0.628688531	0.016023223	1	0.868819888
0.022493006	-0.250515427	0.018163994	2.98E-07	0.868819888
0.022561691	0.743295861	0.307400178	0.416730107	0.868819888
0.023012791	0.549791162	0.737584497	0.001090387	0.868819888
0.02356695	-0.326103482	0.075374611	3.66E-06	0.868819888
0.02356695	-0.326103482	0.075374611	3.66E-06	0.868819888
0.023934986	-0.153020295	0.000852046	6.53E-09	0.868819888
0.024266901	0.73241342	0.584854032	0.164911511	0.868819888
0.024266901	0.73241342	0.584854032	0.164911511	0.868819888
0.024266901	0.73241342	0.584854032	0.164911511	0.868819888
0.024266901	0.73241342	0.584854032	0.164911511	0.868819888
0.024302879	-0.364457705	0.169239932	1.04E-05	0.868819888
0.024670484	-0.726230574	0.584854032	0.145037597	0.868819888
0.024670484	-0.726230574	0.584854032	0.145037597	0.868819888
0.024670484	-0.726230574	0.584854032	0.145037597	0.868819888
0.024670484	-0.726230574	0.584854032	0.145037597	0.868819888
0.024777578	0.423223868	0.307400178	6.41E-05	0.868819888
0.025281808	0.299729601	4.58E-06	0.031639242	0.868819888
0.025526216	-0.479058618	0.439859057	0.0002997	0.868819888
0.025597818	-0.353471761	0.104319013	9.47E-06	0.868819888
0.025597818	-0.353471761	0.104319013	9.47E-06	0.868819888
0.025620355	-0.72514545	0.584854032	0.164911511	0.868819888
0.025620355	-0.72514545	0.584854032	0.164911511	0.868819888

0.026498871	-0.69174954	1	0.060775899	0.868819888
0.026498871	-0.69174954	1	0.060775899	0.868819888
0.026678768	-0.649751936	1	0.02341474	0.868819888
0.027059731	0.551690889	0.881481882	0.002093839	0.868819888
0.027108172	-0.719813757	0.307400178	0.489952655	0.868819888
0.027325748	-0.692646068	1	0.066020955	0.868819888
0.027631791	0.691910507	1	0.066020955	0.868819888
0.027857194	0.551413059	0.881481882	0.002093839	0.868819888
0.027899185	0.713381949	0.205025676	0.601859652	0.868819888
0.028395531	0.435891431	0.000249829	0.272770942	0.868819888
0.028395531	0.435891431	0.000249829	0.272770942	0.868819888
0.028420321	-0.473961327	0.439859057	0.000324671	0.868819888
0.029290471	-0.15402175	0.0008771	1.22E-08	0.868819888
0.029290471	-0.15402175	0.0008771	1.22E-08	0.868819888
0.029290471	-0.15402175	0.0008771	1.22E-08	0.868819888
0.029353519	-0.368385176	0.169239932	2.13E-05	0.868819888
0.029373099	0.67807681	0.087427713	1	0.868819888
0.029394403	-0.348047255	0.104319013	1.04E-05	0.868819888
0.029394403	-0.348047255	0.104319013	1.04E-05	0.868819888
0.029394403	-0.348047255	0.104319013	1.04E-05	0.868819888
0.030113741	-0.692297427	0.881481882	0.126792252	0.868819888
0.030224006	0.206760978	1.10E-07	0.002721298	0.868819888
0.0304345	0.166944235	4.36E-08	0.000596242	0.868819888
0.030779528	0.223125812	4.10E-07	0.005827837	0.868819888
0.030779528	0.223125812	4.10E-07	0.005827837	0.868819888
0.030840459	-0.667255698	0.075374611	1	0.868819888
0.030857552	0.707929234	0.439859057	0.305102445	0.868819888
0.030857552	0.707929234	0.439859057	0.305102445	0.868819888
0.031033376	-0.331595241	0.095006685	9.47E-06	0.868819888
0.031452019	0.378263996	6.77E-05	0.145037597	0.868819888
0.031452019	0.378263996	6.77E-05	0.145037597	0.868819888
0.031825134	0.618514525	1	0.02341474	0.868819888
0.031825134	0.618514525	1	0.02341474	0.868819888
0.031834368	0.683161903	0.989933981	0.102782106	0.868819888
0.031834368	0.683161903	0.989933981	0.102782106	0.868819888
0.03192897	-0.634850895	1	0.025431136	0.868819888
0.032506356	0.493893107	0.584854032	0.000997588	0.868819888
0.032622944	-0.556043101	1	0.003736177	0.868819888
0.032687661	0.701425605	0.439859057	0.397942841	0.868819888
0.032835749	-0.262996409	0.030989574	8.55E-07	0.868819888
0.033447175	-0.609427534	1	0.017403168	0.868819888
0.033447175	-0.609427534	1	0.017403168	0.868819888
0.033539367	-0.554994829	1	0.004171959	0.868819888
0.033539367	-0.554994829	1	0.004171959	0.868819888
0.033553912	-0.695880627	0.584854032	0.242167012	0.868819888
0.033553912	-0.695880627	0.584854032	0.242167012	0.868819888
0.033600083	-0.689004	0.737584497	0.145037597	0.868819888
0.033600083	-0.689004	0.737584497	0.145037597	0.868819888
0.033866546	-0.327656175	0.095006685	9.47E-06	0.868819888
0.034235252	0.119771076	6.38E-09	4.69E-05	0.868819888
0.034235252	0.119771076	6.38E-09	4.69E-05	0.868819888
0.034392602	0.350311356	0.134257478	1.80E-05	0.868819888
0.034392602	0.350311356	0.134257478	1.80E-05	0.868819888
0.034392602	0.350311356	0.134257478	1.80E-05	0.868819888
0.034429364	-0.690347624	0.584854032	0.242167012	0.868819888
0.034429364	-0.690347624	0.584854032	0.242167012	0.868819888
0.034429364	-0.690347624	0.584854032	0.242167012	0.868819888
0.034429364	-0.690347624	0.584854032	0.242167012	0.868819888
0.034751265	-0.472952081	0.439859057	0.000596242	0.868819888
0.035139419	0.507829538	0.691111318	0.001791344	0.868819888
0.035207852	-0.386082187	0.205025676	6.41E-05	0.868819888
0.035305135	-0.332644422	0.095538718	1.04E-05	0.868819888
0.035305135	-0.332644422	0.095538718	1.04E-05	0.868819888
0.035558686	-0.683995217	0.584854032	0.164911511	0.868819888

0.035558686	-0.683995217	0.584854032	0.164911511	0.868819888
0.035558686	-0.683995217	0.584854032	0.164911511	0.868819888
0.035628767	-0.258605546	0.030989574	8.55E-07	0.868819888
0.036885412	-0.469530369	0.439859057	0.000997588	0.868819888
0.036885412	-0.469530369	0.439859057	0.000997588	0.868819888
0.037255424	0.68435322	0.493333254	0.305102445	0.868819888
0.037255424	0.68435322	0.493333254	0.305102445	0.868819888
0.037557097	0.322209841	4.14E-05	0.060775899	0.868819888
0.037706077	-0.655147697	1	0.085309726	0.868819888
0.038005628	-0.392511544	0.295435331	0.000115129	0.868819888
0.038005628	-0.392511544	0.295435331	0.000115129	0.868819888
0.038005628	-0.392511544	0.295435331	0.000115129	0.868819888
0.038201912	-0.643627384	1	0.060775899	0.868819888
0.038340182	0.658014636	1	0.102782106	0.868819888
0.038766783	-0.27167813	0.034012327	3.06E-06	0.868819888
0.038880977	-0.287643572	0.043105813	4.11E-06	0.868819888
0.038880977	-0.287643572	0.043105813	4.11E-06	0.868819888
0.038918715	-0.197478824	0.004526275	2.98E-07	0.868819888
0.038962697	-0.679006436	0.412603306	0.489952655	0.868819888
0.03899777	0.177441322	9.36E-08	0.001090387	0.868819888
0.039475457	-0.241076361	0.018394332	8.55E-07	0.868819888
0.039640129	-0.236031612	0.018163994	8.55E-07	0.868819888
0.040251447	0.433001862	0.439859057	0.000324671	0.868819888
0.040264031	0.309850229	0.075374611	9.47E-06	0.868819888
0.040264031	0.309850229	0.075374611	9.47E-06	0.868819888
0.040341406	-0.419005665	0.412603306	0.0002997	0.868819888
0.041065756	0.293210081	0.043105813	5.99E-06	0.868819888
0.041065756	0.293210081	0.043105813	5.99E-06	0.868819888
0.041065756	0.293210081	0.043105813	5.99E-06	0.868819888
0.041065756	0.293210081	0.043105813	5.99E-06	0.868819888
0.041065756	0.293210081	0.043105813	5.99E-06	0.868819888
0.041065756	0.293210081	0.043105813	5.99E-06	0.868819888
0.041238056	-0.332348014	0.095538718	1.80E-05	0.868819888
0.041238056	-0.332348014	0.095538718	1.80E-05	0.868819888
0.041238056	-0.332348014	0.095538718	1.80E-05	0.868819888
0.041238056	-0.332348014	0.095538718	1.80E-05	0.868819888
0.041344769	0.29714526	0.043105813	9.47E-06	0.868819888
0.041961106	-0.668616483	0.584854032	0.242167012	0.868819888
0.041961106	-0.668616483	0.584854032	0.242167012	0.868819888
0.041961106	-0.668616483	0.584854032	0.242167012	0.868819888
0.041974662	-0.441231319	0.439859057	0.000596242	0.868819888
0.042208691	-0.402762595	0.34036444	0.000163543	0.868819888
0.042506986	0.668631282	0.439859057	0.489952655	0.868819888
0.04268729	-0.401539341	0.307400178	0.000163543	0.868819888
0.043143063	0.629771326	1	0.066020955	0.868819888
0.043446012	0.376686879	0.205025676	0.000115129	0.868819888
0.043466303	-0.661515883	0.584854032	0.242167012	0.868819888
0.043466303	-0.661515883	0.584854032	0.242167012	0.868819888
0.043466303	-0.661515883	0.584854032	0.242167012	0.868819888
0.043466303	-0.661515883	0.584854032	0.242167012	0.868819888
0.043466303	-0.661515883	0.584854032	0.242167012	0.868819888
0.043466303	-0.661515883	0.584854032	0.242167012	0.868819888
0.043950487	-0.104094432	6.19E-05	3.90E-09	0.868819888
0.043976613	-0.637868453	1	0.10273905	0.868819888
0.043976613	-0.637868453	1	0.10273905	0.868819888
0.04459961	0.651450825	0.205025676	0.850173656	0.868819888
0.044657359	0.202652696	4.10E-07	0.003736177	0.868819888
0.044761404	0.246895303	4.58E-06	0.011906673	0.868819888
0.044881068	0.6333657	1	0.085309726	0.868819888
0.044881068	0.6333657	1	0.085309726	0.868819888
0.044927344	0.324350084	0.095538718	1.93E-05	0.868819888
0.044957942	0.495302907	0.737584497	0.002420613	0.868819888
0.045094187	0.515320951	0.008580492	0.850173656	0.868819888
0.045671272	-0.270610845	0.035148121	4.11E-06	0.868819888

0.045671272	-0.270610845	0.035148121	4.11E-06	0.868819888
0.045671272	-0.270610845	0.035148121	4.11E-06	0.868819888
0.045772919	-0.232096965	0.018163994	8.55E-07	0.868819888
0.045901209	0.644735529	0.88322726	0.145037597	0.868819888
0.045901209	0.644735529	0.88322726	0.145037597	0.868819888
0.045901209	0.644735529	0.88322726	0.145037597	0.868819888
0.046304539	-0.652541189	0.691111318	0.242167012	0.868819888
0.046345686	-0.65649619	0.584854032	0.364167203	0.868819888
0.046345686	-0.65649619	0.584854032	0.364167203	0.868819888
0.046462452	0.110923125	6.38E-09	4.69E-05	0.868819888
0.047056504	0.650100959	0.737584497	0.242167012	0.868819888
0.047056504	0.650100959	0.737584497	0.242167012	0.868819888
0.047166339	0.637643765	0.19926681	1	0.868819888
0.047166339	0.637643765	0.19926681	1	0.868819888
0.047379208	-0.651003994	0.584854032	0.242167012	0.868819888
0.047379208	-0.651003994	0.584854032	0.242167012	0.868819888
0.047452137	-0.64522408	0.737584497	0.164911511	0.868819888
0.047452137	-0.64522408	0.737584497	0.164911511	0.868819888
0.047452137	-0.64522408	0.737584497	0.164911511	0.868819888
0.047457691	-0.390616454	0.307400178	0.000163543	0.868819888
0.047457691	-0.390616454	0.307400178	0.000163543	0.868819888
0.047869093	-0.422517875	0.412603306	0.000596242	0.868819888
0.047972549	-0.17058436	0.001207877	9.79E-08	0.868819888
0.047972549	-0.17058436	0.001207877	9.79E-08	0.868819888
0.048246925	-0.652701162	0.439859057	0.416730107	0.868819888
0.048530004	-0.263805932	0.034012327	4.11E-06	0.868819888
0.048530004	-0.263805932	0.034012327	4.11E-06	0.868819888
0.048530004	-0.263805932	0.034012327	4.11E-06	0.868819888
0.048530004	-0.263805932	0.034012327	4.11E-06	0.868819888
0.048763522	-0.160912145	0.0008771	9.27E-08	0.868819888
0.04881438	0.149655671	4.36E-08	0.000324671	0.868819888
0.048914045	0.203656268	1.09E-06	0.003736177	0.868819888
0.04915092	0.650263664	0.439859057	0.489952655	0.868819888
0.04915092	0.650263664	0.439859057	0.489952655	0.868819888
0.049203234	-0.646925254	0.584854032	0.242167012	0.868819888
0.049499334	-0.288326583	0.043105813	9.47E-06	0.868819888
0.049499334	-0.288326583	0.043105813	9.47E-06	0.868819888
0.049512676	-0.339535173	0.169239932	4.69E-05	0.868819888
0.049589561	0.646688831	0.412603306	0.601859652	0.868819888
0.049658303	0.608995188	0.095006685	1	0.868819888
0.050250147	-0.515957636	0.989933981	0.006372161	0.868819888
0.050385612	0.148366319	4.36E-08	0.000324671	0.868819888
0.050385612	0.148366319	4.36E-08	0.000324671	0.868819888
0.05089417	0.602680367	1	0.060775899	0.868819888
0.05089417	0.602680367	1	0.060775899	0.868819888
0.050966006	0.645333221	0.439859057	0.416730107	0.868819888
0.05096929	-0.284550733	0.043105813	9.47E-06	0.868819888
0.05096929	-0.284550733	0.043105813	9.47E-06	0.868819888
0.051075	-0.324289834	0.095538718	4.69E-05	0.868819888
0.051075	-0.324289834	0.095538718	4.69E-05	0.868819888
0.051255161	0.327375184	6.77E-05	0.085309726	0.868819888
0.05128188	-0.235720754	0.018394332	3.06E-06	0.868819888
0.05153766	0.596444139	0.075374611	1	0.868819888
0.051637895	-0.605890828	1	0.06824239	0.868819888
0.051640883	-0.265625486	0.035148121	4.11E-06	0.868819888
0.051910169	0.633837562	0.737584497	0.242167012	0.868819888
0.052395061	0.339982423	0.169239932	6.41E-05	0.868819888
0.052395061	0.339982423	0.169239932	6.41E-05	0.868819888
0.053132926	-0.569445098	0.043105813	1	0.868819888
0.053132926	-0.569445098	0.043105813	1	0.868819888
0.053229818	0.597927564	1	0.061934943	0.868819888
0.053267566	0.552204995	1	0.02341474	0.868819888
0.053317073	-0.624398371	0.989933981	0.145037597	0.868819888
0.053480627	0.636542542	0.412603306	0.645935818	0.868819888

0.053736921	-0.482476518	0.737584497	0.003736177	0.868819888
0.053869407	0.44590714	0.002297238	0.489952655	0.868819888
0.054119612	0.632722489	0.412603306	0.704162183	0.868819888
0.054188959	0.465312308	0.004273917	0.601859652	0.868819888
0.054249272	0.449220509	0.584854032	0.001791344	0.868819888
0.054323672	0.377941221	0.307400178	0.00028972	0.868819888
0.054323672	0.377941221	0.307400178	0.00028972	0.868819888
0.054325493	-0.630139271	0.307400178	0.704162183	0.868819888
0.054429794	0.18521625	4.10E-07	0.002093839	0.868819888
0.054540437	-0.629806048	0.737584497	0.242167012	0.868819888
0.054650065	0.487866956	0.737584497	0.004171959	0.868819888
0.054650065	0.487866956	0.737584497	0.004171959	0.868819888
0.054650065	0.487866956	0.737584497	0.004171959	0.868819888
0.054650065	0.487866956	0.737584497	0.004171959	0.868819888
0.054913604	0.592641155	1	0.061934943	0.868819888
0.055276188	-0.428371735	0.439859057	0.001090387	0.868819888
0.055276188	-0.428371735	0.439859057	0.001090387	0.868819888
0.055439557	0.516731662	1	0.009270366	0.868819888
0.055587919	-0.399043868	0.412603306	0.000348916	0.868819888
0.055587919	-0.399043868	0.412603306	0.000348916	0.868819888
0.055685098	0.624177527	0.307400178	0.850173656	0.868819888
0.055738027	-0.108393462	6.77E-05	6.53E-09	0.868819888
0.055738027	-0.108393462	6.77E-05	6.53E-09	0.868819888
0.055738027	-0.108393462	6.77E-05	6.53E-09	0.868819888
0.055822453	-0.619614223	0.989933981	0.145037597	0.868819888
0.055971107	-0.601226829	1	0.085309726	0.868819888
0.056111134	0.488263683	0.737584497	0.005827837	0.868819888
0.056365111	0.618125509	0.989933981	0.145037597	0.868819888
0.056673054	0.233891053	4.58E-06	0.011906673	0.868819888
0.056684522	-0.303648242	0.087427713	4.35E-05	0.868819888
0.056794339	-0.620149119	0.881481882	0.164911511	0.868819888
0.057106081	-0.609018053	1	0.145037597	0.868819888
0.057220212	-0.594179883	1	0.085309726	0.868819888
0.057272227	-0.589676353	1	0.066020955	0.868819888
0.057351843	-0.371988431	0.295435331	0.00028972	0.868819888
0.057351843	-0.371988431	0.295435331	0.00028972	0.868819888
0.057861821	0.587216636	1	0.066020955	0.868819888
0.057897116	0.628460535	0.439859057	0.489952655	0.868819888
0.057963977	0.120417448	2.61E-08	0.000115129	0.868819888
0.058112627	-0.597625974	1	0.10273905	0.868819888
0.058187631	-0.569801882	0.066393258	1	0.868819888
0.058490233	0.283070147	0.043105813	1.29E-05	0.868819888
0.058711434	0.391336794	0.0008771	0.242167012	0.868819888
0.058908579	-0.611266028	0.205025676	1	0.868819888
0.058934164	0.613563877	0.989933981	0.164911511	0.868819888
0.058978645	-0.277497775	0.043105813	1.04E-05	0.868819888
0.059203379	-0.159840643	0.0008771	9.79E-08	0.868819888
0.059203379	-0.159840643	0.0008771	9.79E-08	0.868819888
0.059203379	-0.159840643	0.0008771	9.79E-08	0.868819888
0.059203379	-0.159840643	0.0008771	9.79E-08	0.868819888
0.059203379	-0.159840643	0.0008771	9.79E-08	0.868819888
0.059203379	-0.159840643	0.0008771	9.79E-08	0.868819888
0.059425011	-0.192487118	0.004526275	5.53E-07	0.868819888
0.059425011	-0.192487118	0.004526275	5.53E-07	0.868819888
0.059927036	0.458234546	0.584854032	0.002420613	0.868819888
0.060008595	0.621739207	0.584854032	0.364167203	0.868819888
0.060549324	0.394588259	0.0008771	0.242167012	0.868819888
0.060601032	0.57520636	1	0.060775899	0.868819888
0.061267787	-0.604910397	1	0.145037597	0.868819888
0.061267787	-0.604910397	1	0.145037597	0.868819888
0.061267787	-0.604910397	1	0.145037597	0.868819888
0.061267787	-0.604910397	1	0.145037597	0.868819888
0.061624101	0.391703781	0.0008771	0.242167012	0.868819888
0.061720378	-0.619744858	0.439859057	0.489952655	0.868819888

0.061720378	-0.619744858	0.439859057	0.489952655	0.868819888
0.061726191	0.465910417	0.584854032	0.003736177	0.868819888
0.06173499	0.61089373	0.307400178	0.850173656	0.868819888
0.06173499	0.61089373	0.307400178	0.850173656	0.868819888
0.06173499	0.61089373	0.307400178	0.850173656	0.868819888
0.061743225	-0.304758006	0.095006685	4.69E-05	0.868819888
0.062058289	0.412592088	0.001207877	0.397942841	0.868819888
0.062095467	0.462909319	0.584854032	0.003736177	0.868819888
0.062095467	0.462909319	0.584854032	0.003736177	0.868819888
0.062184142	-0.249433743	0.034012327	4.11E-06	0.868819888
0.062239067	-0.478163019	0.737584497	0.005827837	0.868819888
0.06284475	-0.597605995	1	0.145037597	0.868819888
0.06284475	-0.597605995	1	0.145037597	0.868819888
0.06284475	-0.597605995	1	0.145037597	0.868819888
0.06284475	-0.597605995	1	0.145037597	0.868819888
0.062915849	0.593456484	0.192696374	1	0.868819888
0.063213243	0.238943103	7.65E-06	0.011906673	0.868819888
0.063213243	0.238943103	7.65E-06	0.011906673	0.868819888
0.063213243	0.238943103	7.65E-06	0.011906673	0.868819888
0.063213243	0.238943103	7.65E-06	0.011906673	0.868819888
0.063213243	0.238943103	7.65E-06	0.011906673	0.868819888
0.063213243	0.238943103	7.65E-06	0.011906673	0.868819888
0.063310165	0.551103621	1	0.041232736	0.868819888
0.063453712	0.162432884	1.48E-07	0.001090387	0.868819888
0.063521962	-0.610117978	0.737584497	0.242167012	0.868819888
0.063521962	-0.610117978	0.737584497	0.242167012	0.868819888
0.063663475	-0.606005071	0.881481882	0.242167012	0.868819888
0.063772826	0.405434392	0.412603306	0.000997588	0.868819888
0.064301731	0.262768445	1.56E-05	0.025431136	0.868819888
0.065205346	0.60356382	0.307400178	0.850173656	0.868819888
0.065288392	-0.373679914	0.307400178	0.000324671	0.868819888
0.065288392	-0.373679914	0.307400178	0.000324671	0.868819888
0.065288392	-0.373679914	0.307400178	0.000324671	0.868819888
0.065349745	0.387866765	0.412603306	0.000596242	0.868819888
0.065356833	0.297680262	0.075374611	4.69E-05	0.868819888
0.06557857	0.152793998	9.36E-08	0.000596242	0.868819888
0.065648402	0.074568079	1.44E-09	3.06E-06	0.868819888
0.065872461	0.610308505	0.439859057	0.489952655	0.868819888
0.065894107	0.572613629	1	0.085309726	0.868819888
0.066353709	0.13202655	4.36E-08	0.000163543	0.868819888
0.066660401	-0.241010743	0.030989574	4.11E-06	0.868819888
0.066680438	-0.569171936	0.095538718	1	0.868819888
0.066680438	-0.569171936	0.095538718	1	0.868819888
0.066706471	0.488690915	0.881481882	0.009270366	0.868819888
0.066706471	0.488690915	0.881481882	0.009270366	0.868819888
0.066706471	0.488690915	0.881481882	0.009270366	0.868819888
0.066720474	-0.328140404	0.169239932	0.000115129	0.868819888
0.066720474	-0.328140404	0.169239932	0.000115129	0.868819888
0.066785354	-0.168919049	0.001207877	2.98E-07	0.868819888
0.067336864	0.588596637	1	0.145037597	0.868819888
0.067336864	0.588596637	1	0.145037597	0.868819888
0.067572032	0.215936146	4.58E-06	0.006372161	0.868819888
0.067862879	0.337945912	0.000261772	0.145037597	0.868819888
0.067958901	0.304564648	8.53E-05	0.066020955	0.868819888
0.068337154	0.578881093	1	0.126792252	0.868819888
0.068337154	0.578881093	1	0.126792252	0.868819888
0.06863908	-0.475671988	0.737584497	0.006372161	0.868819888
0.06863908	-0.475671988	0.737584497	0.006372161	0.868819888
0.068680905	0.084025399	1.89E-09	5.99E-06	0.868819888
0.068767619	-0.237183428	0.030989574	4.11E-06	0.868819888
0.068767619	-0.237183428	0.030989574	4.11E-06	0.868819888
0.068910302	-0.56945379	1	0.085309726	0.868819888
0.069005473	0.359352686	0.295435331	0.0002997	0.868819888
0.069822126	-0.297090675	0.087427713	4.69E-05	0.868819888

0.069984357	0.60205269	0.439859057	0.489952655	0.868819888
0.07043966	0.433013303	0.004526275	0.489952655	0.868819888
0.070474578	0.596560079	0.412603306	0.704162183	0.868819888
0.07057942	-0.503421662	0.030989574	1	0.868819888
0.071052264	0.440114349	0.584854032	0.003736177	0.868819888
0.071313415	0.324960788	0.000245628	0.126792252	0.868819888
0.0713598	-0.599036315	0.439859057	0.601859652	0.868819888
0.0713598	-0.599036315	0.439859057	0.601859652	0.868819888
0.071762168	0.541175236	0.073031096	1	0.868819888
0.071886553	0.598138039	0.439859057	0.489952655	0.868819888
0.071886553	0.598138039	0.439859057	0.489952655	0.868819888
0.072061608	0.389944562	0.412603306	0.000997588	0.868819888
0.072061608	0.389944562	0.412603306	0.000997588	0.868819888
0.072133118	0.485959608	0.989933981	0.011906673	0.868819888
0.072841624	-0.490405525	1	0.011906673	0.868819888
0.07291559	0.542403141	1	0.060775899	0.868819888
0.072974566	-0.596248557	0.584854032	0.489952655	0.868819888
0.073262314	-0.581494306	1	0.164911511	0.868819888
0.073262314	-0.581494306	1	0.164911511	0.868819888
0.073447721	0.431006938	0.004526275	0.489952655	0.868819888
0.073657787	-0.594137123	0.439859057	0.645935818	0.868819888
0.073740139	-0.335924918	0.19926681	0.000163543	0.868819888
0.073967507	-0.374981686	0.412603306	0.000596242	0.868819888
0.073967507	-0.374981686	0.412603306	0.000596242	0.868819888
0.074099449	-0.169768725	0.002297238	2.98E-07	0.868819888
0.074099449	-0.169768725	0.002297238	2.98E-07	0.868819888
0.074099449	-0.169768725	0.002297238	2.98E-07	0.868819888
0.074157442	-0.332435906	0.192696374	0.000140627	0.868819888
0.074157442	-0.332435906	0.192696374	0.000140627	0.868819888
0.074209952	0.396400065	0.412603306	0.001090387	0.868819888
0.074280418	-0.584165113	0.989933981	0.242167012	0.868819888
0.074280418	-0.584165113	0.989933981	0.242167012	0.868819888
0.074416941	0.582273524	1	0.242167012	0.868819888
0.074416941	0.582273524	1	0.242167012	0.868819888
0.074539902	0.484071699	0.989933981	0.011906673	0.868819888
0.074556541	0.591491462	0.439859057	0.704162183	0.868819888
0.07463541	0.582186859	0.307400178	1	0.868819888
0.07463541	0.582186859	0.307400178	1	0.868819888
0.074672918	-0.53647707	0.075374611	1	0.868819888
0.074798662	0.249038135	1.56E-05	0.025431136	0.868819888
0.074813412	0.411231931	0.003169634	0.416730107	0.868819888
0.074869493	-0.592087233	0.439859057	0.601859652	0.868819888
0.074869493	-0.592087233	0.439859057	0.601859652	0.868819888
0.074957864	-0.586286346	0.881481882	0.242167012	0.868819888
0.074980115	-0.33442211	0.19926681	0.000163543	0.868819888
0.075289882	0.442399514	0.008580492	0.645935818	0.868819888
0.075289882	0.442399514	0.008580492	0.645935818	0.868819888
0.075526082	0.548918517	0.095538718	1	0.868819888
0.075734632	0.421833406	0.439859057	0.002420613	0.868819888
0.075734632	0.421833406	0.439859057	0.002420613	0.868819888
0.075734632	0.421833406	0.439859057	0.002420613	0.868819888
0.075806344	-0.116005948	0.000155445	1.63E-08	0.868819888
0.075907206	0.570309387	1	0.145037597	0.868819888
0.076147794	-0.218826486	0.018163994	3.16E-06	0.868819888
0.076147794	-0.218826486	0.018163994	3.16E-06	0.868819888
0.076199425	0.078701657	1.89E-09	4.11E-06	0.868819888
0.07644672	-0.129916842	0.000295301	5.53E-08	0.868819888
0.076481816	-0.416347367	0.439859057	0.002420613	0.868819888
0.076481816	-0.416347367	0.439859057	0.002420613	0.868819888
0.076481816	-0.416347367	0.439859057	0.002420613	0.868819888
0.076538646	0.349014935	0.205025676	0.000324671	0.868819888
0.076627384	0.525526249	0.043105813	1	0.868819888
0.076855523	0.094093996	7.89E-09	1.65E-05	0.868819888
0.076855523	0.094093996	7.89E-09	1.65E-05	0.868819888

0.076863507	-0.505980457	1	0.025431136	0.868819888
0.076863507	-0.505980457	1	0.025431136	0.868819888
0.077211061	-0.558319959	0.169239932	1	0.868819888
0.077570452	-0.434265791	0.584854032	0.003736177	0.868819888
0.077631774	-0.586350833	0.584854032	0.416730107	0.868819888
0.077631774	-0.586350833	0.584854032	0.416730107	0.868819888
0.077750264	-0.321709397	0.169239932	0.000124759	0.868819888
0.077761044	0.587085587	0.439859057	0.489952655	0.868819888
0.077761044	0.587085587	0.439859057	0.489952655	0.868819888
0.077773438	0.470508201	0.881481882	0.009270366	0.868819888
0.077913992	-0.581666282	0.412603306	0.850173656	0.868819888
0.078358914	-0.584663347	0.584854032	0.416730107	0.868819888
0.078358914	-0.584663347	0.584854032	0.416730107	0.868819888
0.079258086	-0.583869043	0.584854032	0.489952655	0.868819888
0.079519839	0.465068847	0.018163994	0.850173656	0.868819888
0.079598211	-0.309464475	0.104319013	0.000115129	0.868819888
0.079598211	-0.309464475	0.104319013	0.000115129	0.868819888
0.079604768	0.414220145	0.439859057	0.002420613	0.868819888
0.079728398	-0.323845526	0.169239932	0.000140627	0.868819888
0.079728398	-0.323845526	0.169239932	0.000140627	0.868819888
0.080167883	-0.441309955	0.584854032	0.006372161	0.868819888
0.080413327	-0.160198926	0.001207877	2.98E-07	0.868819888
0.080666516	-0.579541379	0.737584497	0.397942841	0.868819888
0.080666516	-0.579541379	0.737584497	0.397942841	0.868819888
0.080672689	-0.56834098	1	0.242167012	0.868819888
0.08090072	0.242311417	1.56E-05	0.02341474	0.868819888
0.081764151	0.488774855	1	0.02341474	0.868819888
0.081764151	0.488774855	1	0.02341474	0.868819888
0.081764151	0.488774855	1	0.02341474	0.868819888
0.081901038	0.106170519	2.61E-08	4.69E-05	0.868819888
0.081938072	-0.546848196	1	0.126792252	0.868819888
0.081938072	-0.546848196	1	0.126792252	0.868819888
0.0819404	-0.238010962	0.034012327	9.47E-06	0.868819888
0.0819404	-0.238010962	0.034012327	9.47E-06	0.868819888
0.082155755	-0.526034521	1	0.060775899	0.868819888
0.082155755	-0.526034521	1	0.060775899	0.868819888
0.082187989	0.555711171	0.19926681	1	0.868819888
0.082404777	-0.173490862	0.003169634	5.53E-07	0.868819888
0.082404777	-0.173490862	0.003169634	5.53E-07	0.868819888
0.082462457	-0.506148722	1	0.031639242	0.868819888
0.082462457	-0.506148722	1	0.031639242	0.868819888
0.082482944	-0.565656359	1	0.242167012	0.868819888
0.082867497	-0.569449192	0.412603306	1	0.868819888
0.083096325	0.52139636	1	0.060775899	0.868819888
0.083609873	-0.553545277	0.19926681	1	0.868819888
0.083753427	-0.445331969	0.737584497	0.006372161	0.868819888
0.083762364	-0.573717537	0.737584497	0.397942841	0.868819888
0.083762364	-0.573717537	0.737584497	0.397942841	0.868819888
0.084018859	0.317880048	0.169239932	0.000140627	0.868819888
0.084181788	0.55232822	1	0.145037597	0.868819888
0.084181788	0.55232822	1	0.145037597	0.868819888
0.084181788	0.55232822	1	0.145037597	0.868819888
0.08428092	0.383583192	0.001611045	0.364167203	0.868819888
0.085033652	-0.56752115	0.88322726	0.242167012	0.868819888
0.085196137	-0.486781192	0.034012327	1	0.868819888
0.085196137	-0.486781192	0.034012327	1	0.868819888
0.085568198	0.382499253	0.412603306	0.001791344	0.868819888
0.085568198	0.382499253	0.412603306	0.001791344	0.868819888
0.085568198	0.382499253	0.412603306	0.001791344	0.868819888
0.085655447	0.557049419	1	0.164911511	0.868819888
0.086021842	0.246175745	6.19E-05	0.025431136	0.868819888
0.086362666	0.530444119	1	0.085309726	0.868819888
0.086535691	0.282211108	0.075374611	6.41E-05	0.868819888
0.086917695	0.386924223	0.003169634	0.397942841	0.868819888

0.086999287	-0.566231209	0.412603306	0.850173656	0.868819888
0.086999287	-0.566231209	0.412603306	0.850173656	0.868819888
0.087087352	-0.494008976	1	0.031639242	0.868819888
0.087087352	-0.494008976	1	0.031639242	0.868819888
0.087101892	0.402746236	0.439859057	0.002420613	0.868819888
0.087154737	0.3786977	0.412603306	0.001791344	0.868819888
0.087194149	0.561510302	1	0.242167012	0.868819888
0.087194149	0.561510302	1	0.242167012	0.868819888
0.087240551	0.551781478	1	0.145037597	0.868819888
0.087495995	-0.55396336	1	0.164911511	0.868819888
0.087594089	0.385462296	0.003169634	0.397942841	0.868819888
0.087643623	0.533083961	0.134257478	1	0.868819888
0.087899103	-0.544407074	0.19926681	1	0.868819888
0.088200506	-0.128414945	0.000467492	9.27E-08	0.868819888
0.088241625	0.49803776	1	0.034534533	0.868819888
0.088241625	0.49803776	1	0.034534533	0.868819888
0.088374724	-0.142622345	0.0008771	1.54E-07	0.868819888
0.088577667	0.362853879	0.001207877	0.242167012	0.868819888
0.088577667	0.362853879	0.001207877	0.242167012	0.868819888
0.088577667	0.362853879	0.001207877	0.242167012	0.868819888
0.088588566	-0.562499338	0.412603306	1	0.868819888
0.088661837	0.566330843	0.439859057	0.704162183	0.868819888
0.088661837	0.566330843	0.439859057	0.704162183	0.868819888
0.088661837	0.566330843	0.439859057	0.704162183	0.868819888
0.088677111	0.360781932	0.34036444	0.000997588	0.868819888
0.088677111	0.360781932	0.34036444	0.000997588	0.868819888
0.088884914	0.539310924	0.192696374	1	0.868819888
0.088884914	0.539310924	0.192696374	1	0.868819888
0.088915007	0.560245092	0.989933981	0.242167012	0.868819888
0.089264242	0.172907372	1.23E-06	0.002093839	0.868819888
0.089366877	0.416163369	0.584854032	0.004171959	0.868819888
0.089397225	-0.56231508	0.881481882	0.364167203	0.868819888
0.089397225	-0.56231508	0.881481882	0.364167203	0.868819888
0.089445835	0.364020003	0.001207877	0.242167012	0.868819888
0.089522049	0.271372526	8.53E-05	0.052574033	0.868819888
0.089522049	0.271372526	8.53E-05	0.052574033	0.868819888
0.089675279	-0.16717177	0.003169634	5.53E-07	0.868819888
0.089930624	-0.323892229	0.19926681	0.0002997	0.868819888
0.089967927	-0.379660354	0.412603306	0.001791344	0.868819888
0.090151695	0.421959748	0.584854032	0.005827837	0.868819888
0.090232211	0.373717173	0.412603306	0.001791344	0.868819888
0.090365399	0.451102269	0.737584497	0.011906673	0.868819888
0.090640379	0.488550043	1	0.031639242	0.868819888
0.090640379	0.488550043	1	0.031639242	0.868819888
0.090715767	0.346035634	0.0008771	0.217058541	0.868819888
0.090765261	0.563954565	0.584854032	0.489952655	0.868819888
0.090765261	0.563954565	0.584854032	0.489952655	0.868819888
0.090765261	0.563954565	0.584854032	0.489952655	0.868819888
0.090765261	0.563954565	0.584854032	0.489952655	0.868819888
0.090765261	0.563954565	0.584854032	0.489952655	0.868819888
0.090765261	0.563954565	0.584854032	0.489952655	0.868819888
0.090767153	-0.454479676	0.881481882	0.011906673	0.868819888
0.090805759	0.522638704	0.099208829	1	0.868819888
0.091143802	0.389335042	0.439859057	0.002420613	0.868819888
0.091170334	0.264099061	6.77E-05	0.042826979	0.868819888
0.091223582	-0.198399082	0.010835964	3.06E-06	0.868819888
0.091355927	0.554736564	1	0.242167012	0.868819888
0.091404778	0.561161759	0.439859057	0.704162183	0.868819888
0.091664898	-0.5628616	0.584854032	0.601859652	0.868819888
0.091664898	-0.5628616	0.584854032	0.601859652	0.868819888
0.091788944	-0.558683754	0.439859057	0.850173656	0.868819888
0.09182446	-0.345688388	0.295435331	0.000596242	0.868819888
0.09182446	-0.345688388	0.295435331	0.000596242	0.868819888
0.091897776	0.320443739	0.000467492	0.129712192	0.868819888

0.091982052	-0.491849532	1	0.041232736	0.868819888
0.092247349	-0.265952643	0.043105813	4.69E-05	0.868819888
0.092289639	0.384636911	0.439859057	0.002093839	0.868819888
0.09249034	-0.552555082	1	0.242167012	0.868819888
0.092661956	-0.561144947	0.584854032	0.489952655	0.868819888
0.092752629	0.440748234	0.737584497	0.009270366	0.868819888
0.093009828	0.077070954	1.89E-09	4.11E-06	0.868819888
0.093009828	0.077070954	1.89E-09	4.11E-06	0.868819888
0.093189434	0.316080553	0.000467492	0.126792252	0.868819888
0.093306212	-0.327570503	0.205025676	0.000324671	0.868819888
0.093387695	0.428148085	0.584854032	0.006372161	0.868819888
0.093751323	0.24200771	6.19E-05	0.025431136	0.868819888
0.093955942	0.344760216	0.0008771	0.217058541	0.868819888
0.094124236	0.541083833	1	0.164911511	0.868819888
0.09430147	-0.326582167	0.205025676	0.000324671	0.868819888
0.094796976	0.198190058	4.58E-06	0.006372161	0.868819888
0.094796976	0.198190058	4.58E-06	0.006372161	0.868819888
0.094806491	-0.261493353	0.043105813	4.69E-05	0.868819888
0.094806491	-0.261493353	0.043105813	4.69E-05	0.868819888
0.094920959	-0.233958381	0.034012327	1.04E-05	0.868819888
0.094920959	-0.233958381	0.034012327	1.04E-05	0.868819888
0.095057711	-0.55060632	0.989933981	0.305102445	0.868819888
0.095108422	0.42891868	0.584854032	0.009270366	0.868819888
0.095153063	0.359538463	0.001207877	0.242167012	0.868819888
0.095191338	-0.545602076	0.307400178	1	0.868819888
0.095191338	-0.545602076	0.307400178	1	0.868819888
0.095191338	-0.545602076	0.307400178	1	0.868819888
0.095222331	0.492495878	0.066393258	1	0.868819888
0.095222331	0.492495878	0.066393258	1	0.868819888
0.095223976	-0.473535803	1	0.025431136	0.878100647
0.095773791	0.1077713	2.78E-08	6.41E-05	0.878100647
0.095773791	0.1077713	2.78E-08	6.41E-05	0.878100647
0.095911972	0.261694085	6.77E-05	0.042826979	0.878100647
0.095996965	0.552084815	0.881481882	0.397942841	0.878100647
0.096136998	0.28329293	0.000189446	0.061934943	0.878100647
0.09622951	-0.478863135	1	0.031639242	0.878100647
0.096667538	0.36989121	0.002810027	0.364167203	0.878100647
0.096795076	0.54362627	0.34036444	1	0.878100647
0.096926788	0.531691176	1	0.145037597	0.878100647
0.096926788	0.531691176	1	0.145037597	0.878100647
0.097383677	0.548965438	0.412603306	1	0.878100647
0.097383677	0.548965438	0.412603306	1	0.878100647
0.097383677	0.548965438	0.412603306	1	0.878100647
0.097403795	-0.505609598	1	0.066020955	0.878100647
0.097574441	-0.46955062	1	0.025431136	0.878100647
0.097724817	-0.471558759	1	0.029310175	0.878100647
0.097732025	-0.352487946	0.307400178	0.001090387	0.878100647
0.09799022	0.524515267	1	0.129712192	0.878100647
0.098047848	0.20764343	1.22E-05	0.009270366	0.878100647
0.098139059	-0.533184869	0.208605117	1	0.878100647
0.098360134	0.394807483	0.008580492	0.489952655	0.878100647
0.098504975	-0.543978023	1	0.242167012	0.878100647
0.099243308	0.250601963	6.77E-05	0.031639242	0.878100647
0.099311229	-0.506695045	0.095538718	1	0.878100647
0.099694494	0.53104262	1	0.164911511	0.878100647
0.099694494	0.53104262	1	0.164911511	0.878100647
0.099982431	0.425585498	0.584854032	0.009270366	0.878100647
0.099982431	0.425585498	0.584854032	0.009270366	0.878100647
0.100235365	-0.30254194	0.169239932	0.00028972	0.878100647
0.100256464	0.548862716	0.584854032	0.704162183	0.878100647
0.100294799	-0.269932649	0.075374611	7.48E-05	0.878100647
0.100507223	-0.541150421	0.412603306	1	0.878100647
0.100507223	-0.541150421	0.412603306	1	0.878100647
0.100507223	-0.541150421	0.412603306	1	0.878100647

0.100538475	-0.377912435	0.439859057	0.002420613	0.878100647
0.100572667	0.145363588	4.10E-07	0.000997588	0.878100647
0.100642711	0.392229965	0.439859057	0.003736177	0.878100647
0.100874463	-0.15343112	0.001207877	5.53E-07	0.878100647
0.101182173	0.492445183	0.075374611	1	0.878100647
0.101182173	0.492445183	0.075374611	1	0.878100647
0.102334357	0.510978634	1	0.126792252	0.878100647
0.102615573	0.061889783	1.44E-09	8.55E-07	0.878100647
0.102780131	-0.211092361	0.018394332	9.47E-06	0.878100647
0.102880146	-0.26947496	0.075374611	7.48E-05	0.878100647
0.102880146	-0.26947496	0.075374611	7.48E-05	0.878100647
0.103119873	-0.119444249	0.000261772	9.27E-08	0.878100647
0.103244854	0.257140751	0.043105813	4.69E-05	0.878100647
0.103979711	0.33629436	0.001207877	0.164911511	0.878100647
0.104446683	-0.522461901	1	0.164911511	0.878100647
0.104446683	-0.522461901	1	0.164911511	0.878100647
0.104446683	-0.522461901	1	0.164911511	0.878100647
0.104446683	-0.522461901	1	0.164911511	0.878100647
0.104862505	0.140254062	3.42E-07	0.000596242	0.878100647
0.104959776	0.128350334	1.10E-07	0.0002997	0.878100647
0.105216686	-0.257953478	0.043105813	4.69E-05	0.878100647
0.105441355	0.143146731	4.10E-07	0.000596242	0.878100647
0.105583268	0.175631996	3.89E-06	0.002420613	0.878100647
0.105583268	0.175631996	3.89E-06	0.002420613	0.878100647
0.105583268	0.175631996	3.89E-06	0.002420613	0.878100647
0.105583268	0.175631996	3.89E-06	0.002420613	0.878100647
0.105583268	0.175631996	3.89E-06	0.002420613	0.878100647
0.105583268	0.175631996	3.89E-06	0.002420613	0.878100647
0.105672061	-0.476688778	1	0.052574033	0.878100647
0.105672061	-0.476688778	1	0.052574033	0.878100647
0.105679173	-0.190146168	0.009746439	3.16E-06	0.878100647
0.105730155	0.158513322	1.09E-06	0.001791344	0.878100647
0.105741573	-0.220139424	0.026049691	1.04E-05	0.878100647
0.105741573	-0.220139424	0.026049691	1.04E-05	0.878100647
0.105904287	0.395298389	0.009746439	0.489952655	0.878100647
0.106002925	0.423604201	0.691111318	0.011906673	0.878100647
0.10614822	0.538711685	0.737584497	0.489952655	0.878100647
0.106848858	-0.280601846	0.095006685	0.000124759	0.878100647
0.106848858	-0.280601846	0.095006685	0.000124759	0.878100647
0.107149645	-0.538253771	0.737584497	0.489952655	0.878100647
0.107377609	0.499158099	1	0.10273905	0.878100647
0.107423863	0.535054223	0.439859057	0.850173656	0.878100647
0.107423863	0.535054223	0.439859057	0.850173656	0.878100647
0.107504029	0.512314033	0.19926681	1	0.878100647
0.107749162	0.326807493	0.205025676	0.000997588	0.878100647
0.108451166	0.529823292	1	0.364167203	0.878100647
0.108562008	-0.340304212	0.307400178	0.001090387	0.878100647
0.108562008	-0.340304212	0.307400178	0.001090387	0.878100647
0.109450341	0.496953282	1	0.10273905	0.878100647
0.109802404	-0.501848174	1	0.126792252	0.878100647
0.109926277	0.533092146	0.439859057	0.704162183	0.878100647
0.109926277	0.533092146	0.439859057	0.704162183	0.878100647
0.109962242	-0.279014844	0.095538718	0.000124759	0.878100647
0.110079224	-0.494112935	1	0.085309726	0.878100647
0.110280093	-0.533837665	0.737584497	0.489952655	0.878100647
0.110280093	-0.533837665	0.737584497	0.489952655	0.878100647
0.110280093	-0.533837665	0.737584497	0.489952655	0.878100647
0.110280093	-0.533837665	0.737584497	0.489952655	0.878100647
0.110690635	-0.418878809	0.691111318	0.011906673	0.878100647
0.110998463	-0.353181644	0.412603306	0.002093839	0.878100647
0.110998463	-0.353181644	0.412603306	0.002093839	0.878100647
0.111149492	0.527726898	1	0.364167203	0.878100647
0.111149492	0.527726898	1	0.364167203	0.878100647
0.111204897	-0.346119793	0.349433107	0.001791344	0.878100647

0.111522512	-0.220424751	0.030989574	1.65E-05	0.878100647
0.111770815	0.056162689	1.44E-09	5.53E-07	0.878100647
0.111837414	-0.531577408	0.584854032	0.704162183	0.878100647
0.111932056	0.436209736	0.881481882	0.02341474	0.878100647
0.112077817	-0.482948395	1	0.06824239	0.878100647
0.112077817	-0.482948395	1	0.06824239	0.878100647
0.112106003	0.398625099	0.584854032	0.006372161	0.878100647
0.112106003	0.398625099	0.584854032	0.006372161	0.878100647
0.112106003	0.398625099	0.584854032	0.006372161	0.878100647
0.112567321	-0.254475646	0.043105813	6.41E-05	0.878100647
0.112567321	-0.254475646	0.043105813	6.41E-05	0.878100647
0.113042473	0.509094351	0.205025676	1	0.878100647
0.113202839	-0.527401755	0.881481882	0.416730107	0.878100647
0.11320726	0.524888583	1	0.397942841	0.878100647
0.113318572	-0.528810369	0.737584497	0.489952655	0.878100647
0.113318572	-0.528810369	0.737584497	0.489952655	0.878100647
0.113547248	-0.494144514	1	0.126792252	0.878100647
0.113548527	0.516590152	0.307400178	1	0.878100647
0.113838544	0.510123621	0.295435331	1	0.878100647
0.113941631	-0.495606207	0.169239932	1	0.878100647
0.11413141	-0.506289331	1	0.145037597	0.878100647
0.11413141	-0.506289331	1	0.145037597	0.878100647
0.114256263	-0.262935515	0.075374611	0.000115129	0.878100647
0.114332986	0.386389558	0.439859057	0.005827837	0.878100647
0.114370497	-0.269748644	0.075374611	0.000124759	0.878100647
0.114723477	0.380416914	0.439859057	0.004171959	0.878100647
0.114723477	0.380416914	0.439859057	0.004171959	0.878100647
0.114757991	-0.226905621	0.034012327	1.80E-05	0.878100647
0.114757991	-0.226905621	0.034012327	1.80E-05	0.878100647
0.114934257	0.116188636	9.36E-08	0.000124759	0.878100647
0.115155477	-0.206583635	0.018394332	9.47E-06	0.878100647
0.115155477	-0.206583635	0.018394332	9.47E-06	0.878100647
0.115271668	0.488632228	1	0.102782106	0.878100647
0.11536966	-0.305777343	0.192696374	0.000348916	0.878100647
0.115383067	-0.512782175	1	0.242167012	0.878100647
0.115387649	0.077615747	7.89E-09	5.99E-06	0.878100647
0.11543151	0.465987479	1	0.053084396	0.878100647
0.11543151	0.465987479	1	0.053084396	0.878100647
0.115449014	-0.153057416	0.001207877	8.55E-07	0.878100647
0.115449014	-0.153057416	0.001207877	8.55E-07	0.878100647
0.11586462	-0.524838214	0.439859057	0.850173656	0.878100647
0.116846569	0.211030004	6.10E-05	0.011906673	0.878100647
0.117325172	0.253430473	0.000155445	0.042826979	0.878100647
0.117325172	0.253430473	0.000155445	0.042826979	0.878100647
0.117666078	-0.292391588	0.169239932	0.0002997	0.878100647
0.118210574	0.405390217	0.584854032	0.009270366	0.878100647
0.118210574	0.405390217	0.584854032	0.009270366	0.878100647
0.118559517	-0.522823748	0.584854032	0.704162183	0.878100647
0.118664272	-0.389432803	0.584854032	0.006372161	0.878100647
0.119592856	0.511177417	1	0.242167012	0.878100647
0.120132987	-0.474494134	1	0.085309726	0.878100647
0.120268706	0.131487648	0.0008771	2.98E-07	0.878100647
0.120268706	0.131487648	0.0008771	2.98E-07	0.878100647
0.120355546	0.430237924	0.881481882	0.025431136	0.878100647
0.120476273	0.190621212	1.22E-05	0.006372161	0.878100647
0.120504152	-0.520311555	0.584854032	0.645935818	0.878100647
0.120504152	-0.520311555	0.584854032	0.645935818	0.878100647
0.120643852	0.320378209	0.208605117	0.001090387	0.878100647
0.120643852	0.320378209	0.208605117	0.001090387	0.878100647
0.120691056	-0.506590403	1	0.242167012	0.878100647
0.120691056	-0.506590403	1	0.242167012	0.878100647
0.121182083	0.485329113	1	0.126792252	0.878100647
0.12120706	-0.517637617	0.737584497	0.489952655	0.878100647
0.12120706	-0.517637617	0.737584497	0.489952655	0.878100647

0.121618682	0.153141466	0.001207877	8.55E-07	0.878100647
0.121618682	0.153141466	0.001207877	8.55E-07	0.878100647
0.121618682	0.153141466	0.001207877	8.55E-07	0.878100647
0.121618682	0.153141466	0.001207877	8.55E-07	0.878100647
0.121628197	0.461157576	0.075374611	1	0.878100647
0.121695412	0.49437427	0.205025676	1	0.878100647
0.121724249	-0.233945413	0.035148121	4.69E-05	0.878100647
0.12173261	0.516121974	0.439859057	0.850173656	0.878100647
0.121745071	0.411892631	0.737584497	0.017403168	0.878100647
0.121932197	0.475540935	1	0.085309726	0.878100647
0.121969633	-0.136782908	0.0008771	2.98E-07	0.878100647
0.122128842	-0.325444263	0.295435331	0.001090387	0.878100647
0.122128842	-0.325444263	0.295435331	0.001090387	0.878100647
0.122242981	-0.47291578	1	0.085309726	0.878100647
0.122242981	-0.47291578	1	0.085309726	0.878100647
0.122362545	0.450223818	0.066393258	1	0.878100647
0.122462411	-0.449987972	1	0.049298372	0.878100647
0.122462411	-0.449987972	1	0.049298372	0.878100647
0.122665709	-0.509665403	0.412603306	1	0.878100647
0.12284578	0.471122814	1	0.085309726	0.878100647
0.122902782	0.32435286	0.001207877	0.164911511	0.878100647
0.123307684	0.163647891	3.89E-06	0.002093839	0.878100647
0.123510445	0.510956151	0.439859057	1	0.878100647
0.123713778	-0.474368059	1	0.085309726	0.878100647
0.123967272	-0.494361377	1	0.164911511	0.878100647
0.123985246	-0.320800784	0.295435331	0.001090387	0.878100647
0.124027635	0.484166954	0.192696374	1	0.878100647
0.124067005	0.500422437	1	0.242167012	0.878100647
0.124067005	0.500422437	1	0.242167012	0.878100647
0.12474547	-0.514385268	0.737584497	0.489952655	0.878100647
0.124782775	0.505834228	1	0.305102445	0.878100647
0.124937744	0.312361677	0.001207877	0.145037597	0.878100647
0.124969956	-0.384215899	0.584854032	0.006372161	0.878100647
0.12554089	0.511459105	0.881481882	0.489952655	0.878100647
0.125700898	-0.272022617	0.095538718	0.000163543	0.878100647
0.125700898	-0.272022617	0.095538718	0.000163543	0.878100647
0.126025509	-0.152069863	0.001207877	8.55E-07	0.878100647
0.126337978	-0.509994381	0.439859057	1	0.878100647
0.126448335	-0.133310117	0.0008771	2.98E-07	0.878100647
0.126448335	-0.133310117	0.0008771	2.98E-07	0.878100647
0.126532883	0.393924461	0.584854032	0.009270366	0.878100647
0.126532883	0.393924461	0.584854032	0.009270366	0.878100647
0.126532883	0.393924461	0.584854032	0.009270366	0.878100647
0.12653641	-0.426721989	0.881481882	0.025431136	0.878100647
0.126814824	0.507880074	0.989933981	0.416730107	0.878100647
0.126814824	0.507880074	0.989933981	0.416730107	0.878100647
0.126831234	0.453564375	1	0.060775899	0.878100647
0.127070969	0.504236263	1	0.364167203	0.878100647
0.127128688	-0.287835861	0.169239932	0.000324671	0.878100647
0.127213927	0.169065597	4.58E-06	0.002420613	0.878100647
0.127248557	-0.261947907	0.075374611	0.000124759	0.878100647
0.127248557	-0.261947907	0.075374611	0.000124759	0.878100647
0.127248557	-0.261947907	0.075374611	0.000124759	0.878100647
0.127343338	0.390036327	0.584854032	0.009270366	0.878100647
0.12734378	0.49892606	1	0.242167012	0.878100647
0.127494512	-0.167230052	0.004526275	3.06E-06	0.878100647
0.127749285	0.499343008	1	0.242167012	0.878100647
0.127749285	0.499343008	1	0.242167012	0.878100647
0.127970376	-0.230532726	0.035148121	4.69E-05	0.878100647
0.127970376	-0.230532726	0.035148121	4.69E-05	0.878100647
0.127970376	-0.230532726	0.035148121	4.69E-05	0.878100647
0.127970376	-0.230532726	0.035148121	4.69E-05	0.878100647
0.128081002	0.504673547	0.439859057	1	0.878100647
0.128129253	0.479962685	1	0.145037597	0.878100647

0.128149603	0.499460186	1	0.242167012	0.878100647
0.128149603	0.499460186	1	0.242167012	0.878100647
0.128269716	-0.066853033	4.58E-06	3.90E-09	0.878100647
0.128269716	-0.066853033	4.58E-06	3.90E-09	0.878100647
0.128269716	-0.066853033	4.58E-06	3.90E-09	0.878100647
0.128487881	0.494036502	1	0.242167012	0.878100647
0.128791666	-0.189433698	0.016023223	9.47E-06	0.878100647
0.128791666	-0.189433698	0.016023223	9.47E-06	0.878100647
0.129276261	-0.508763072	0.691111318	0.601859652	0.878100647
0.129637284	0.384556735	0.018163994	0.489952655	0.878100647
0.129637284	0.384556735	0.018163994	0.489952655	0.878100647
0.129789531	0.174273615	5.98E-06	0.003736177	0.878100647
0.129867106	-0.24347147	0.043105813	7.48E-05	0.878100647
0.129933743	-0.441957554	1	0.052574033	0.878100647
0.129955719	-0.318662303	0.295435331	0.001090387	0.878100647
0.129955719	-0.318662303	0.295435331	0.001090387	0.878100647
0.129982261	-0.153493384	0.002297238	8.55E-07	0.878100647
0.129982261	-0.153493384	0.002297238	8.55E-07	0.878100647
0.129982261	-0.153493384	0.002297238	8.55E-07	0.878100647
0.130090593	-0.230615059	0.035148121	4.69E-05	0.878100647
0.130150847	0.1589408	3.58E-06	0.002093839	0.878100647
0.130153234	-0.461165726	1	0.085309726	0.878100647
0.1302457	0.502317242	1	0.397942841	0.878100647
0.1302457	0.502317242	1	0.397942841	0.878100647
0.13025497	0.271690084	0.095538718	0.0002997	0.878100647
0.130328928	0.504939846	0.88322726	0.489952655	0.878100647
0.130354676	-0.326418058	0.307400178	0.001791344	0.878100647
0.130432808	-0.497231128	1	0.242167012	0.878100647
0.130432808	-0.497231128	1	0.242167012	0.878100647
0.130456269	0.345111522	0.412603306	0.002420613	0.878100647
0.130483814	0.20971326	6.19E-05	0.011906673	0.878100647
0.130658498	-0.44970941	0.075374611	1	0.878100647
0.13087018	-0.160983297	0.004273917	3.06E-06	0.878100647
0.130976697	0.188644655	1.22E-05	0.006372161	0.878100647
0.131139852	-0.213831155	0.030989574	2.13E-05	0.878100647
0.131657746	-0.24587755	0.043105813	0.000115129	0.878100647
0.131657746	-0.24587755	0.043105813	0.000115129	0.878100647
0.131680136	-0.431217135	1	0.031639242	0.878100647
0.131758881	-0.321341395	0.307400178	0.001791344	0.878100647
0.131860007	-0.259721417	0.075374611	0.000124759	0.878100647
0.131887097	0.449341034	1	0.060775899	0.878100647
0.132033807	-0.190005159	0.016023223	9.47E-06	0.878100647
0.132072774	0.182518007	1.22E-05	0.005827837	0.878100647
0.132254564	-0.477218506	1	0.145037597	0.878100647
0.132254564	-0.477218506	1	0.145037597	0.878100647
0.132449915	-0.402632905	0.737584497	0.02341474	0.878100647
0.132500832	-0.122138454	0.000467492	2.98E-07	0.878100647
0.132500832	-0.122138454	0.000467492	2.98E-07	0.878100647
0.132500832	-0.122138454	0.000467492	2.98E-07	0.878100647
0.132877057	-0.361094511	0.439859057	0.005827837	0.878100647
0.133235104	0.492507871	1	0.242167012	0.878100647
0.133417733	0.439019278	0.073031096	1	0.878100647
0.133536217	-0.182462719	0.009746439	5.99E-06	0.878100647
0.133536217	-0.182462719	0.009746439	5.99E-06	0.878100647
0.13420444	-0.369438202	0.439859057	0.006372161	0.878100647
0.13420444	-0.369438202	0.439859057	0.006372161	0.878100647
0.135272239	0.406215517	0.737584497	0.02341474	0.878100647
0.135452132	0.438053872	1	0.053084396	0.878100647
0.135629284	0.35757948	0.009746439	0.416730107	0.878100647
0.135711304	-0.370754714	0.439859057	0.006372161	0.878100647
0.135741086	0.291721567	0.169239932	0.000596242	0.878100647
0.136805527	0.403375489	0.737584497	0.02341474	0.878100647
0.136989506	0.498061607	0.881481882	0.489952655	0.878100647
0.137358963	-0.465608794	1	0.126792252	0.878100647

0.137358963	-0.465608794	1	0.126792252	0.878100647
0.137591449	0.41691653	0.043105813	1	0.878100647
0.137741675	0.321917158	0.307400178	0.002093839	0.878100647
0.137741675	0.321917158	0.307400178	0.002093839	0.878100647
0.13781434	0.121651004	3.18E-07	0.0002997	0.878100647
0.13781434	0.121651004	3.18E-07	0.0002997	0.878100647
0.13781434	0.121651004	3.18E-07	0.0002997	0.878100647
0.137919568	-0.455468059	0.134257478	1	0.878100647
0.137919568	-0.455468059	0.134257478	1	0.878100647
0.137992642	-0.392440419	0.584854032	0.017403168	0.878100647
0.13854297	0.156169843	4.58E-06	0.002093839	0.878100647
0.138662738	0.484397466	0.412603306	1	0.878100647
0.138784814	-0.277922942	0.134257478	0.000324671	0.878100647
0.138891743	-0.134050818	0.0008771	5.53E-07	0.878100647
0.139278202	-0.478967538	0.307400178	1	0.878100647
0.139283287	0.407229892	0.737584497	0.025431136	0.878100647
0.139359254	-0.477772798	1	0.242167012	0.878100647
0.139480241	-0.371829414	0.493333254	0.009270366	0.878100647
0.139480241	-0.371829414	0.493333254	0.009270366	0.878100647
0.139653052	-0.48958886	0.439859057	1	0.878100647
0.140201173	-0.366589037	0.439859057	0.006372161	0.878100647
0.140450348	0.346522758	0.412603306	0.003736177	0.878100647
0.140450348	0.346522758	0.412603306	0.003736177	0.878100647
0.140450348	0.346522758	0.412603306	0.003736177	0.878100647
0.140478365	0.452315366	0.134257478	1	0.878100647
0.140508486	-0.483303328	1	0.242167012	0.878100647
0.140650244	0.466291786	1	0.145037597	0.878100647
0.140832051	0.233067135	0.000155445	0.031639242	0.878100647
0.141103718	0.410357422	0.881481882	0.029310175	0.878100647
0.141193304	-0.420271281	0.989933981	0.041232736	0.878100647
0.141231876	-0.469625476	1	0.145037597	0.878100647
0.141363634	-0.473888214	1	0.217058541	0.878100647
0.141363634	-0.473888214	1	0.217058541	0.878100647
0.141363634	-0.473888214	1	0.217058541	0.878100647
0.141456334	-0.226057797	0.035148121	4.69E-05	0.878100647
0.141482294	0.47951939	1	0.242167012	0.878100647
0.141759823	-0.488290993	1	0.416730107	0.878100647
0.142424331	0.388475872	0.584854032	0.017403168	0.878100647
0.142652083	-0.492493921	0.584854032	0.704162183	0.878100647
0.143146294	0.491911434	0.584854032	0.704162183	0.878100647
0.143399102	0.087197354	2.78E-08	1.80E-05	0.878100647
0.143399102	0.087197354	2.78E-08	1.80E-05	0.878100647
0.143494215	-0.439408975	1	0.066020955	0.878100647
0.143494215	-0.439408975	1	0.066020955	0.878100647
0.143876431	-0.286413728	0.169239932	0.000596242	0.878100647
0.143897905	-0.487949043	0.989933981	0.489952655	0.878100647
0.143897905	-0.487949043	0.989933981	0.489952655	0.878100647
0.144198612	-0.481034882	1	0.364167203	0.878100647
0.144433235	-0.362410527	0.439859057	0.006372161	0.878100647
0.144503858	-0.257897768	0.087427713	0.00028972	0.878100647
0.144525678	0.459493329	0.19926681	1	0.878100647
0.144525678	0.459493329	0.19926681	1	0.878100647
0.144945321	-0.145700945	0.001207877	8.55E-07	0.878100647
0.144945321	-0.145700945	0.001207877	8.55E-07	0.878100647
0.145144879	0.143558463	0.001207877	8.55E-07	0.878100647
0.145213047	0.38841747	0.584854032	0.02341474	0.878100647
0.145337591	-0.11275984	0.000261772	2.98E-07	0.878100647
0.145337591	-0.11275984	0.000261772	2.98E-07	0.878100647
0.145537654	-0.289489495	0.192696374	0.000997588	0.878100647
0.145537654	-0.289489495	0.192696374	0.000997588	0.878100647
0.145678679	0.405077796	0.03951775	0.850173656	0.878100647
0.145696598	-0.487657954	0.584854032	0.850173656	0.878100647
0.14582844	-0.31569066	0.307400178	0.002093839	0.878100647
0.145868659	0.129955167	1.09E-06	0.000596242	0.878100647

0.146279271	-0.286430749	0.169239932	0.000997588	0.878100647
0.146367277	0.463281146	1	0.145037597	0.878100647
0.146711196	-0.449206733	1	0.126792252	0.878100647
0.14674078	-0.485285018	0.989933981	0.489952655	0.878100647
0.146931595	0.486938869	0.584854032	0.704162183	0.878100647
0.146947389	-0.421530211	1	0.053084396	0.878100647
0.14764918	0.394531313	0.737584497	0.025431136	0.878100647
0.147885134	0.458942801	0.205025676	1	0.878100647
0.147919863	0.431225799	1	0.061934943	0.878100647
0.148200545	0.235528422	0.043105813	0.000115129	0.878100647
0.148200545	0.235528422	0.043105813	0.000115129	0.878100647
0.148670983	0.476334118	0.412603306	1	0.878100647
0.148670983	0.476334118	0.412603306	1	0.878100647
0.148856587	0.141910475	1.84E-06	0.001090387	0.878100647
0.148856587	0.141910475	1.84E-06	0.001090387	0.878100647
0.149220233	-0.221958237	0.035148121	6.41E-05	0.878100647
0.149385534	-0.182340808	0.010835964	9.47E-06	0.878100647
0.149385534	-0.182340808	0.010835964	9.47E-06	0.878100647
0.149385534	-0.182340808	0.010835964	9.47E-06	0.878100647
0.149593921	0.271820776	0.104319013	0.000348916	0.878100647
0.149593921	0.271820776	0.104319013	0.000348916	0.878100647
0.149593921	0.271820776	0.104319013	0.000348916	0.878100647
0.149593921	0.271820776	0.104319013	0.000348916	0.878100647
0.14966333	0.256209514	0.000467492	0.060775899	0.878100647
0.149687945	-0.474158615	1	0.364167203	0.878100647
0.149723612	-0.183091111	0.010835964	9.47E-06	0.878100647
0.149875791	-0.35346651	0.439859057	0.006372161	0.878100647
0.149875791	-0.35346651	0.439859057	0.006372161	0.878100647
0.149875791	-0.35346651	0.439859057	0.006372161	0.878100647
0.149881035	0.327164168	0.006343149	0.242167012	0.878100647
0.149881035	0.327164168	0.006343149	0.242167012	0.878100647
0.150337718	-0.463353675	1	0.242167012	0.878100647
0.15047952	-0.325518749	0.412603306	0.002721298	0.878100647
0.151591878	-0.39065206	0.737584497	0.025431136	0.878100647
0.151591878	-0.39065206	0.737584497	0.025431136	0.878100647
0.151606064	-0.474061331	1	0.364167203	0.878100647
0.151606064	-0.474061331	1	0.364167203	0.878100647
0.151915595	0.439675468	1	0.10273905	0.878100647
0.151915595	0.439675468	1	0.10273905	0.878100647
0.152016334	0.22211717	0.000155445	0.025431136	0.878100647
0.152016334	0.22211717	0.000155445	0.025431136	0.878100647
0.152280952	0.47041093	1	0.305102445	0.878100647
0.152280952	0.47041093	1	0.305102445	0.878100647
0.152529218	0.436293752	0.104319013	1	0.878100647
0.152549595	0.435219319	1	0.085309726	0.878100647
0.152610357	0.090949973	4.36E-08	4.69E-05	0.878100647
0.152610357	0.090949973	4.36E-08	4.69E-05	0.878100647
0.152900555	-0.317350419	0.307400178	0.002420613	0.878100647
0.153000545	-0.294840227	0.205025676	0.001090387	0.878100647
0.153244006	0.463426484	1	0.242167012	0.878100647
0.153631589	-0.073490385	1.22E-05	9.50E-09	0.878100647
0.153631589	-0.073490385	1.22E-05	9.50E-09	0.878100647
0.153631589	-0.073490385	1.22E-05	9.50E-09	0.878100647
0.154006985	-0.288640162	0.19926681	0.001090387	0.878100647
0.154050059	-0.472850666	1	0.397942841	0.878100647
0.154066453	-0.35964879	0.439859057	0.009270366	0.878100647
0.154086432	-0.478480085	0.881481882	0.489952655	0.878100647
0.154157517	-0.397947844	0.737584497	0.031639242	0.878100647
0.15460564	-0.478284451	0.737584497	0.601859652	0.878100647
0.154676613	-0.186414331	0.016023223	1.29E-05	0.878100647
0.154676613	-0.186414331	0.016023223	1.29E-05	0.878100647
0.154676613	-0.186414331	0.016023223	1.29E-05	0.878100647
0.154676613	-0.186414331	0.016023223	1.29E-05	0.878100647
0.154714858	-0.348220743	0.439859057	0.006372161	0.878100647

0.155224186	-0.226210574	0.043105813	0.000115129	0.878100647
0.155681889	0.264056347	0.095538718	0.000324671	0.878100647
0.155681889	0.264056347	0.095538718	0.000324671	0.878100647
0.155692425	0.468448761	0.412603306	1	0.878100647
0.155899028	0.302571545	0.295435331	0.001791344	0.878100647
0.155902028	0.477312667	0.691111318	0.704162183	0.878100647
0.156078624	-0.366500989	0.584854032	0.011906673	0.878100647
0.156104936	0.299747159	0.002297238	0.145037597	0.878100647
0.156104936	0.299747159	0.002297238	0.145037597	0.878100647
0.156225071	0.417828976	0.075374611	1	0.878100647
0.156225071	0.417828976	0.075374611	1	0.878100647
0.156743077	-0.132004602	0.0008771	8.55E-07	0.878100647
0.156996255	-0.412304634	1	0.053084396	0.878100647
0.157260941	-0.239366286	0.066393258	0.000124759	0.878100647
0.157281028	-0.313816614	0.307400178	0.002420613	0.878100647
0.157281028	-0.313816614	0.307400178	0.002420613	0.878100647
0.157341199	-0.179819905	0.010835964	9.47E-06	0.878100647
0.157341199	-0.179819905	0.010835964	9.47E-06	0.878100647
0.157341199	-0.179819905	0.010835964	9.47E-06	0.878100647
0.157341199	-0.179819905	0.010835964	9.47E-06	0.878100647
0.157341199	-0.179819905	0.010835964	9.47E-06	0.878100647
0.157341199	-0.179819905	0.010835964	9.47E-06	0.878100647
0.157725943	-0.186851033	0.018163994	1.65E-05	0.878100647
0.157744966	0.404696057	0.043105813	1	0.878100647
0.157744966	0.404696057	0.043105813	1	0.878100647
0.15781594	0.440076806	1	0.126792252	0.878100647
0.157909667	0.400862363	0.881481882	0.041232736	0.878100647
0.158010515	0.25004573	0.000467492	0.060775899	0.878100647
0.158280964	0.111460257	3.18E-07	0.000140627	0.878100647
0.158558553	-0.421902514	1	0.066020955	0.878100647
0.158558553	-0.421902514	1	0.066020955	0.878100647
0.158833494	-0.155455895	0.004273917	3.16E-06	0.878100647
0.158833494	-0.155455895	0.004273917	3.16E-06	0.878100647
0.159609956	0.394135271	0.814576593	0.031639242	0.878100647
0.159961603	-0.464090401	1	0.364167203	0.878100647
0.159961603	-0.464090401	1	0.364167203	0.878100647
0.159961603	-0.464090401	1	0.364167203	0.878100647
0.160108809	-0.291777107	0.205025676	0.001791344	0.878100647
0.160402075	-0.275631782	0.169239932	0.000997588	0.878100647
0.160826495	-0.274854612	0.169239932	0.000997588	0.878100647
0.160834469	0.359594958	0.584854032	0.011906673	0.878100647
0.160834469	0.359594958	0.584854032	0.011906673	0.878100647
0.160834469	0.359594958	0.584854032	0.011906673	0.878100647
0.161529852	0.461292656	1	0.364167203	0.878100647
0.161529852	0.461292656	1	0.364167203	0.878100647
0.161553992	0.454224956	1	0.242167012	0.878100647
0.16171111	0.37574008	0.034012327	0.704162183	0.878100647
0.16204023	-0.425257788	1	0.085309726	0.878100647
0.162391314	-0.251461896	0.087427713	0.000324671	0.878100647
0.162787239	0.463916945	1	0.416730107	0.878100647
0.162988933	-0.181180212	0.016023223	1.04E-05	0.878100647
0.162988933	-0.181180212	0.016023223	1.04E-05	0.878100647
0.163085955	0.45802001	0.412603306	1	0.878100647
0.163085955	0.45802001	0.412603306	1	0.878100647
0.163235939	-0.403823876	1	0.053084396	0.878100647
0.163483998	-0.246979695	0.075374611	0.0002997	0.878100647
0.163761703	-0.425614814	1	0.10273905	0.878100647
0.164128892	0.468135779	0.584854032	0.704162183	0.878100647
0.164128892	0.468135779	0.584854032	0.704162183	0.878100647
0.164128892	0.468135779	0.584854032	0.704162183	0.878100647
0.164128892	0.468135779	0.584854032	0.704162183	0.878100647
0.164161112	0.452787223	1	0.242167012	0.878100647
0.164161112	0.452787223	1	0.242167012	0.878100647
0.164612927	-0.139502224	0.001207877	3.06E-06	0.878100647

0.164690527	-0.335817151	0.439859057	0.006372161	0.878100647
0.164767246	0.038923762	1.97E-10	9.27E-08	0.878100647
0.164767246	0.038923762	1.97E-10	9.27E-08	0.878100647
0.164999683	0.467105657	0.584854032	0.850173656	0.878100647
0.165208295	0.377149398	0.691111318	0.025431136	0.878100647
0.165208295	0.377149398	0.691111318	0.025431136	0.878100647
0.165208295	0.377149398	0.691111318	0.025431136	0.878100647
0.165393524	-0.418524616	1	0.085309726	0.878100647
0.165405637	-0.243349025	0.075374611	0.0002997	0.878100647
0.165405637	-0.243349025	0.075374611	0.0002997	0.878100647
0.165434818	-0.447685479	0.307400178	1	0.878100647
0.165434818	-0.447685479	0.307400178	1	0.878100647
0.165539991	0.216123749	0.035148121	0.000108916	0.878100647
0.1658963	-0.150121591	0.003169634	3.16E-06	0.878100647
0.165954023	0.435906466	1	0.145037597	0.878100647
0.165996054	-0.220735723	0.03951775	0.000115129	0.878100647
0.165996054	-0.220735723	0.03951775	0.000115129	0.878100647
0.166498603	-0.459160033	1	0.416730107	0.878100647
0.166543215	0.426162236	0.169239932	1	0.878100647
0.166543215	0.426162236	0.169239932	1	0.878100647
0.166903621	0.307788804	0.307400178	0.002420613	0.878100647
0.167016195	-0.461868552	0.439859057	1	0.878100647
0.167016195	-0.461868552	0.439859057	1	0.878100647
0.167019848	0.462678971	0.439859057	1	0.878100647
0.167019848	0.462678971	0.439859057	1	0.878100647
0.167019848	0.462678971	0.439859057	1	0.878100647
0.167019848	0.462678971	0.439859057	1	0.878100647
0.167049611	0.461890954	1	0.489952655	0.878100647
0.167140093	0.218126381	0.000189446	0.025431136	0.878100647
0.167140093	0.218126381	0.000189446	0.025431136	0.878100647
0.167379226	0.432264637	1	0.145037597	0.878100647
0.167470704	-0.294686785	0.208605117	0.002093839	0.878100647
0.167696353	-0.231869611	0.043105813	0.000140627	0.878100647
0.167696353	-0.231869611	0.043105813	0.000140627	0.878100647
0.1677801	0.462794347	0.584854032	1	0.878100647
0.168118946	0.463467471	0.584854032	0.850173656	0.878100647
0.168141953	0.1729209	1.56E-05	0.005827837	0.878100647
0.168465437	-0.186856949	0.018163994	1.80E-05	0.878100647
0.168532131	0.253467323	0.0008771	0.066020955	0.878100647
0.168532131	0.253467323	0.0008771	0.066020955	0.878100647
0.168532131	0.253467323	0.0008771	0.066020955	0.878100647
0.168566337	0.430541434	1	0.145037597	0.878100647
0.168688258	0.171654402	1.56E-05	0.005827837	0.878100647
0.1687414	0.108299897	3.18E-07	0.000124759	0.878100647
0.169102078	0.198020952	6.77E-05	0.011906673	0.878100647
0.169119543	-0.456206803	1	0.416730107	0.878100647
0.169119543	-0.456206803	1	0.416730107	0.878100647
0.169235012	-0.288537202	0.205025676	0.001791344	0.878100647
0.169451569	-0.101512943	0.000189446	1.54E-07	0.878100647
0.169451569	-0.101512943	0.000189446	1.54E-07	0.878100647
0.169719902	0.402106724	0.075374611	1	0.878100647
0.169719902	0.402106724	0.075374611	1	0.878100647
0.170392025	-0.443942103	1	0.242167012	0.878100647
0.170729172	-0.110854596	0.000295301	2.98E-07	0.878100647
0.17097323	-0.369230653	0.584854032	0.025431136	0.878100647
0.17097323	-0.369230653	0.584854032	0.025431136	0.878100647
0.171350405	-0.054046525	1.23E-06	3.90E-09	0.878100647
0.171386586	0.442084619	1	0.242167012	0.878100647
0.171386586	0.442084619	1	0.242167012	0.878100647
0.171406614	0.35713544	0.584854032	0.017403168	0.878100647
0.171406614	0.35713544	0.584854032	0.017403168	0.878100647
0.171406614	0.35713544	0.584854032	0.017403168	0.878100647
0.171498436	-0.433388461	1	0.145037597	0.878100647
0.171544723	0.142375168	4.58E-06	0.001791344	0.878100647

0.171544723	0.142375168	4.58E-06	0.001791344	0.878100647
0.171768506	0.415679815	1	0.085309726	0.878100647
0.171768506	0.415679815	1	0.085309726	0.878100647
0.171876422	0.35375717	0.026049691	0.489952655	0.878100647
0.172219316	0.162640706	1.22E-05	0.003736177	0.878100647
0.172219316	0.162640706	1.22E-05	0.003736177	0.878100647
0.172245712	-0.138626969	0.001207877	3.06E-06	0.878100647
0.172245712	-0.138626969	0.001207877	3.06E-06	0.878100647
0.172279027	0.127663446	1.23E-06	0.000596242	0.878100647
0.172291794	0.454595651	1	0.416730107	0.878100647
0.172337254	-0.20958587	0.034012327	7.48E-05	0.878100647
0.172337254	-0.20958587	0.034012327	7.48E-05	0.878100647
0.172550655	-0.130295962	0.0008771	8.55E-07	0.878100647
0.172828753	-0.125232964	0.0008771	8.55E-07	0.878100647
0.173053272	-0.453762136	1	0.416730107	0.878100647
0.173160852	0.318237749	0.412603306	0.005827837	0.878100647
0.173567273	0.192042139	0.024201521	4.69E-05	0.878100647
0.173567273	0.192042139	0.024201521	4.69E-05	0.878100647
0.173567273	0.192042139	0.024201521	4.69E-05	0.878100647
0.173567273	0.192042139	0.024201521	4.69E-05	0.878100647
0.173567273	0.192042139	0.024201521	4.69E-05	0.878100647
0.173567273	0.192042139	0.024201521	4.69E-05	0.878100647
0.17376799	-0.20044944	0.030989574	4.69E-05	0.878100647
0.173798051	0.429095061	1	0.145037597	0.878100647
0.173855701	-0.209806099	0.034012327	7.48E-05	0.878100647
0.174072316	-0.102995143	0.000245628	2.98E-07	0.878100647
0.174246171	-0.321715171	0.412603306	0.006372161	0.878100647
0.174246171	-0.321715171	0.412603306	0.006372161	0.878100647
0.174311944	-0.457317416	0.881481882	0.645935818	0.878100647
0.174311944	-0.457317416	0.881481882	0.645935818	0.878100647
0.174474478	-0.457380783	0.737584497	0.704162183	0.878100647
0.174567897	-0.444081739	1	0.242167012	0.878100647
0.174648746	0.32397689	0.010835964	0.364167203	0.878100647
0.175037274	0.448547143	0.439859057	1	0.878100647
0.175037274	0.448547143	0.439859057	1	0.878100647
0.175690497	0.290139482	0.205025676	0.002093839	0.878100647
0.175997407	-0.159185216	0.007135475	9.47E-06	0.878100647
0.176022561	-0.434086877	0.307400178	1	0.878100647
0.176039015	0.375793193	0.737584497	0.031639242	0.878100647
0.17609871	0.339071207	0.439859057	0.009270366	0.878100647
0.176268756	-0.450856431	0.439859057	1	0.878100647
0.176514495	-0.453604838	1	0.489952655	0.878100647
0.17657033	0.398158636	1	0.060775899	0.878100647
0.177009421	-0.351908555	0.584854032	0.02341474	0.878100647
0.177009421	-0.351908555	0.584854032	0.02341474	0.878100647
0.177109319	-0.306828051	0.34036444	0.003736177	0.878100647
0.177344999	-0.451352126	1	0.489952655	0.878100647
0.177371486	-0.453073637	0.989933981	0.489952655	0.878100647
0.177574102	-0.130360414	0.0008771	8.55E-07	0.878100647
0.177574102	-0.130360414	0.0008771	8.55E-07	0.878100647
0.177671197	0.436134904	0.307400178	1	0.878100647
0.1785023	0.315024564	0.009746439	0.272770942	0.878100647
0.178508863	-0.453261827	0.584854032	0.850173656	0.878100647
0.178516017	0.066405404	7.89E-09	3.66E-06	0.878100647
0.178516017	0.066405404	7.89E-09	3.66E-06	0.878100647
0.178516017	0.066405404	7.89E-09	3.66E-06	0.878100647
0.178516017	0.066405404	7.89E-09	3.66E-06	0.878100647
0.178516017	0.066405404	7.89E-09	3.66E-06	0.878100647
0.178516017	0.066405404	7.89E-09	3.66E-06	0.878100647
0.178646702	-0.238568023	0.075374611	0.0002997	0.878100647
0.178646702	-0.238568023	0.075374611	0.0002997	0.878100647
0.178908095	-0.098830467	0.000189446	1.54E-07	0.878100647
0.178924273	-0.444190523	1	0.397942841	0.878100647
0.179101976	-0.326689037	0.439859057	0.006372161	0.878100647

0.179101976	-0.326689037	0.439859057	0.006372161	0.878100647
0.179769557	-0.249361153	0.095538718	0.000596242	0.878100647
0.17983301	0.433634279	0.307400178	1	0.878100647
0.179992471	-0.320245793	0.412603306	0.006372161	0.878100647
0.179992471	-0.320245793	0.412603306	0.006372161	0.878100647
0.180137509	-0.304144411	0.307400178	0.003736177	0.878100647
0.180231941	0.445748261	1	0.416730107	0.878100647
0.180231941	0.445748261	1	0.416730107	0.878100647
0.18042142	-0.164911007	0.008580492	9.47E-06	0.878100647
0.180674417	-0.205047371	0.034012327	7.48E-05	0.878100647
0.180783209	0.311605673	0.412603306	0.005827837	0.878100647
0.180783209	0.311605673	0.412603306	0.005827837	0.878100647
0.180801445	-0.44972491	0.989933981	0.489952655	0.878100647
0.180810012	-0.425994202	0.273258425	1	0.878100647
0.181143933	0.300566113	0.307400178	0.003736177	0.878100647
0.181164643	0.282292849	0.003169634	0.145037597	0.878100647
0.181269859	-0.328445976	0.439859057	0.009270366	0.878100647
0.181269859	-0.328445976	0.439859057	0.009270366	0.878100647
0.181269859	-0.328445976	0.439859057	0.009270366	0.878100647
0.18172276	0.302425065	0.307400178	0.003736177	0.878100647
0.182156225	0.210788427	0.000228282	0.025431136	0.878100647
0.18222284	0.353193285	0.584854032	0.02341474	0.878100647
0.182474154	0.431687455	1	0.242167012	0.878100647
0.182474154	0.431687455	1	0.242167012	0.878100647
0.182474154	0.431687455	1	0.242167012	0.878100647
0.182852357	-0.417103897	1	0.145037597	0.878100647
0.183128559	0.434615566	1	0.242167012	0.878100647
0.183128559	0.434615566	1	0.242167012	0.878100647
0.183416738	0.446571426	0.584854032	1	0.878100647
0.183523299	-0.448208004	0.584854032	0.850173656	0.878100647
0.183802505	0.428984733	1	0.242167012	0.878100647
0.183946264	0.299081084	0.307400178	0.003736177	0.878100647
0.1840634	0.307161219	0.00974133	0.242167012	0.878100647
0.1844145	-0.445888026	1	0.489952655	0.878100647
0.184642825	-0.447318736	0.737584497	0.704162183	0.878100647
0.184642825	-0.447318736	0.737584497	0.704162183	0.878100647
0.184649279	0.233135717	0.075374611	0.0002997	0.878100647
0.184649279	0.233135717	0.075374611	0.0002997	0.878100647
0.1847319	-0.186610433	0.018394332	4.69E-05	0.878100647
0.185027348	-0.443412915	1	0.489952655	0.878100647
0.18506653	0.359749151	0.584854032	0.025431136	0.878100647
0.18506653	0.359749151	0.584854032	0.025431136	0.878100647
0.18506653	0.359749151	0.584854032	0.025431136	0.878100647
0.185194126	0.430793886	1	0.242167012	0.878100647
0.185822295	0.414085341	0.19926681	1	0.878100647
0.18613263	0.276303539	0.19926681	0.001791344	0.878100647
0.186620586	-0.402023608	1	0.102782106	0.878100647
0.186620586	-0.402023608	1	0.102782106	0.878100647
0.186730164	0.427371248	0.307400178	1	0.878100647
0.187009345	0.401129738	0.134257478	1	0.878100647
0.187009345	0.401129738	0.134257478	1	0.878100647
0.187546727	-0.431744194	0.412603306	1	0.878100647
0.187713094	0.301956933	0.008580492	0.242167012	0.878100647
0.188142499	-0.1584617	0.008580492	9.47E-06	0.878100647
0.18843233	0.283399501	0.205025676	0.002420613	0.878100647
0.188467751	0.425396508	0.307400178	1	0.878100647
0.188834099	-0.291150414	0.307400178	0.003736177	0.878100647
0.188885556	-0.436992802	1	0.416730107	0.878100647
0.189184347	-0.432574746	1	0.364167203	0.878100647
0.189184347	-0.432574746	1	0.364167203	0.878100647
0.189208906	0.437149347	1	0.416730107	0.878100647
0.189208906	0.437149347	1	0.416730107	0.878100647
0.189305198	0.440508598	0.584854032	1	0.878100647
0.189410344	-0.337597913	0.439859057	0.017403168	0.878100647

0.189686258	-0.42195175	1	0.242167012	0.878100647
0.189686258	-0.42195175	1	0.242167012	0.878100647
0.189686258	-0.42195175	1	0.242167012	0.878100647
0.189999109	-0.30599236	0.009746439	0.242167012	0.878100647
0.189999109	-0.30599236	0.009746439	0.242167012	0.878100647
0.190031112	0.20283628	0.000189446	0.02341474	0.878100647
0.190268038	-0.248041345	0.095538718	0.000596242	0.878100647
0.190268038	-0.248041345	0.095538718	0.000596242	0.878100647
0.190468537	0.410551053	0.205025676	1	0.878100647
0.190468537	0.410551053	0.205025676	1	0.878100647
0.190468537	0.410551053	0.205025676	1	0.878100647
0.190628048	0.148509741	0.004273917	4.11E-06	0.878100647
0.190628048	0.148509741	0.004273917	4.11E-06	0.878100647
0.190688262	0.048147327	1.75E-09	2.98E-07	0.878100647
0.190693406	0.42806406	1	0.272770942	0.878100647
0.190709876	0.384680223	1	0.061934943	0.878100647
0.190757605	-0.423765329	1	0.242167012	0.878100647
0.190892223	-0.44119404	0.737584497	0.704162183	0.878100647
0.191183794	-0.426320603	1	0.242167012	0.878100647
0.191183794	-0.426320603	1	0.242167012	0.878100647
0.192001309	0.109698437	1.09E-06	0.00028972	0.878100647
0.192005828	0.13262842	4.58E-06	0.001090387	0.878100647
0.192234303	0.372246586	0.881481882	0.052574033	0.878100647
0.192248197	0.437724004	1	0.489952655	0.878100647
0.192248197	0.437724004	1	0.489952655	0.878100647
0.192249137	-0.187297225	0.025193624	4.69E-05	0.878100647
0.192249137	-0.187297225	0.025193624	4.69E-05	0.878100647
0.192249137	-0.187297225	0.025193624	4.69E-05	0.878100647
0.192249137	-0.187297225	0.025193624	4.69E-05	0.878100647
0.192614019	0.388891456	0.095538718	1	0.878100647
0.192924137	0.329561747	0.439859057	0.011906673	0.878100647
0.192998916	0.347599319	0.584854032	0.025431136	0.878100647
0.193103838	0.067305944	2.61E-08	4.11E-06	0.878100647
0.193103838	0.067305944	2.61E-08	4.11E-06	0.878100647
0.193235031	0.279570635	0.004273917	0.145037597	0.878100647
0.193262041	-0.427223345	1	0.364167203	0.878100647
0.193262041	-0.427223345	1	0.364167203	0.878100647
0.193449094	0.434262014	1	0.489952655	0.878100647
0.193502514	0.185464373	6.77E-05	0.011906673	0.878100647
0.193502514	0.185464373	6.77E-05	0.011906673	0.878100647
0.193572977	-0.414051525	1	0.164911511	0.878100647
0.193572977	-0.414051525	1	0.164911511	0.878100647
0.193572977	-0.414051525	1	0.164911511	0.878100647
0.193704166	-0.413837159	0.295435331	1	0.878100647
0.193916199	0.420630023	0.34036444	1	0.878100647
0.194230414	0.426359935	0.412603306	1	0.878100647
0.194230414	0.426359935	0.412603306	1	0.878100647
0.194234434	0.40967907	1	0.145037597	0.878100647
0.194234434	0.40967907	1	0.145037597	0.878100647
0.194259002	0.252201765	0.104319013	0.000997588	0.878100647
0.194292133	0.191552042	8.53E-05	0.017403168	0.878100647
0.194743341	0.352181607	0.03951775	0.645935818	0.878100647
0.194743341	0.352181607	0.03951775	0.645935818	0.878100647
0.194744319	0.417479409	1	0.242167012	0.878100647
0.19485988	0.401381014	1	0.129712192	0.878100647
0.194989289	-0.02405564	7.89E-09	1.09E-10	0.878100647
0.194989289	-0.02405564	7.89E-09	1.09E-10	0.878100647
0.194989289	-0.02405564	7.89E-09	1.09E-10	0.878100647
0.195086915	0.21632926	0.000295301	0.031639242	0.878100647
0.195096641	-0.371617947	0.075374611	0.850173656	0.878100647
0.195115911	0.145332649	1.22E-05	0.002093839	0.878100647
0.195196703	0.362970705	0.043105813	0.704162183	0.878100647
0.195258324	0.313244279	0.016023223	0.364167203	0.878100647
0.195686549	0.300935951	0.412603306	0.005827837	0.878100647

0.195822724	0.121137597	1.84E-06	0.000596242	0.878100647
0.19596098	0.416528992	0.307400178	1	0.878100647
0.196013776	-0.427375646	1	0.397942841	0.878100647
0.196013776	-0.427375646	1	0.397942841	0.878100647
0.196013776	-0.427375646	1	0.397942841	0.878100647
0.196013776	-0.427375646	1	0.397942841	0.878100647
0.196072715	-0.137399254	0.001611045	3.16E-06	0.878100647
0.196072715	-0.137399254	0.001611045	3.16E-06	0.878100647
0.196165014	0.405973318	0.205025676	1	0.878100647
0.196165014	0.405973318	0.205025676	1	0.878100647
0.196238878	-0.431532289	1	0.489952655	0.878100647
0.196238878	-0.431532289	1	0.489952655	0.878100647
0.19628914	-0.276216691	0.205025676	0.002420613	0.878100647
0.196302468	0.405090312	1	0.145037597	0.878100647
0.196302468	0.405090312	1	0.145037597	0.878100647
0.196302596	0.278765389	0.205025676	0.002420613	0.878100647
0.196690159	-0.426070848	1	0.397942841	0.878100647
0.196862939	-0.340800998	0.584854032	0.02341474	0.878100647
0.197081333	0.43280478	0.584854032	1	0.878100647
0.197143104	0.245390629	0.0008771	0.085309726	0.878100647
0.197912509	-0.421662897	1	0.305102445	0.878100647
0.197912509	-0.421662897	1	0.305102445	0.878100647
0.198785617	0.421999305	0.412603306	1	0.878100647
0.198982316	0.432978875	0.88322726	0.704162183	0.878100647
0.199067071	-0.389126872	1	0.10273905	0.878100647
0.19949157	0.432754436	0.881481882	0.704162183	0.878100647
0.199801073	0.145149533	1.22E-05	0.002093839	0.878100647
0.199867505	0.16861171	0.010835964	1.80E-05	0.878100647
0.200093019	0.204420457	0.000249829	0.025431136	0.878100647
0.200106396	-0.317951217	0.439859057	0.009270366	0.878100647
0.200106396	-0.317951217	0.439859057	0.009270366	0.878100647
0.200285962	-0.425804542	1	0.416730107	0.878100647
0.200589439	-0.376150396	1	0.061934943	0.878100647
0.201217686	0.315821579	0.439859057	0.009270366	0.878100647
0.201217686	0.315821579	0.439859057	0.009270366	0.878100647
0.201801745	0.430703747	0.881481882	0.704162183	0.878100647
0.201899607	0.430496685	0.881481882	0.704162183	0.878100647
0.202269496	0.398020612	1	0.145037597	0.878100647
0.202401209	0.190401122	0.000155445	0.017403168	0.878100647
0.202450301	-0.40301501	0.205025676	1	0.878100647
0.202450301	-0.40301501	0.205025676	1	0.878100647
0.202450301	-0.40301501	0.205025676	1	0.878100647
0.202450301	-0.40301501	0.205025676	1	0.878100647
0.202518402	0.217204302	0.000467492	0.041232736	0.878100647
0.202687199	0.393464599	1	0.126792252	0.878100647
0.202687199	0.393464599	1	0.126792252	0.878100647
0.202687199	0.393464599	1	0.126792252	0.878100647
0.202820613	-0.429583296	0.881481882	0.704162183	0.878100647
0.203346642	0.419645858	0.439859057	1	0.878100647
0.203346642	0.419645858	0.439859057	1	0.878100647
0.203487011	0.158167283	6.19E-05	0.004171959	0.878100647
0.203503979	0.063259071	2.61E-08	3.66E-06	0.878100647
0.204356436	-0.415691972	1	0.364167203	0.878100647
0.204404476	-0.424363746	1	0.489952655	0.878100647
0.204416645	0.227732237	0.0008771	0.053084396	0.878100647
0.204586098	-0.180957121	0.018394332	4.69E-05	0.878100647
0.204586098	-0.180957121	0.018394332	4.69E-05	0.878100647
0.204586098	-0.180957121	0.018394332	4.69E-05	0.878100647
0.204586098	-0.180957121	0.018394332	4.69E-05	0.878100647
0.204586098	-0.180957121	0.018394332	4.69E-05	0.878100647
0.204586098	-0.180957121	0.018394332	4.69E-05	0.878100647
0.205044623	0.293893794	0.349433107	0.005827837	0.878100647
0.205044623	0.293893794	0.349433107	0.005827837	0.878100647
0.205044623	0.293893794	0.349433107	0.005827837	0.878100647

0.205859286	0.045865978	1.75E-09	2.98E-07	0.878100647
0.205859286	0.045865978	1.75E-09	2.98E-07	0.878100647
0.205866553	0.420564353	1	0.416730107	0.878100647
0.206018326	-0.395424245	1	0.145037597	0.878100647
0.206018326	-0.395424245	1	0.145037597	0.878100647
0.206129552	-0.247538564	0.104319013	0.001090387	0.878100647
0.206140147	-0.243933255	0.095538718	0.000997588	0.878100647
0.206192055	0.399438649	0.205025676	1	0.878100647
0.206246933	-0.414957402	1	0.364167203	0.878100647
0.206246933	-0.414957402	1	0.364167203	0.878100647
0.206630939	0.395684633	1	0.145037597	0.878100647
0.20664414	-0.328120524	0.439859057	0.02341474	0.878100647
0.20664414	-0.328120524	0.439859057	0.02341474	0.878100647
0.206967864	-0.206979076	0.043105813	0.000163543	0.878100647
0.206967864	-0.206979076	0.043105813	0.000163543	0.878100647
0.206967864	-0.206979076	0.043105813	0.000163543	0.878100647
0.206967864	-0.206979076	0.043105813	0.000163543	0.878100647
0.207043088	0.23270245	0.0008771	0.060775899	0.878100647
0.207043088	0.23270245	0.0008771	0.060775899	0.878100647
0.207372326	-0.425669079	0.881481882	0.704162183	0.878100647
0.207586622	0.326481118	0.439859057	0.02341474	0.878100647
0.207638728	0.394821007	0.205025676	1	0.878100647
0.207638728	0.394821007	0.205025676	1	0.878100647
0.207718936	-0.376268396	1	0.085309726	0.878100647
0.207718936	-0.376268396	1	0.085309726	0.878100647
0.20811335	-0.334081423	0.034012327	0.489952655	0.878100647
0.20811335	-0.334081423	0.034012327	0.489952655	0.878100647
0.208423233	0.367555079	0.989933981	0.061934943	0.878100647
0.208489938	0.291289263	0.010835964	0.242167012	0.878100647
0.208583206	0.424545069	0.881481882	0.704162183	0.878100647
0.208601382	-0.417217129	1	0.416730107	0.878100647
0.208601382	-0.417217129	1	0.416730107	0.878100647
0.208640644	-0.282831737	0.307400178	0.003736177	0.878100647
0.20878876	0.11429449	1.23E-06	0.000324671	0.878100647
0.208853746	0.394220058	0.205025676	1	0.878100647
0.209158881	0.240791771	0.095538718	0.000997588	0.878100647
0.209261458	-0.419041627	1	0.489952655	0.878100647
0.209533755	-0.420648898	1	0.489952655	0.878100647
0.210032778	0.301602292	0.412603306	0.009270366	0.878100647
0.210032778	0.301602292	0.412603306	0.009270366	0.878100647
0.210172428	-0.222691536	0.073031096	0.000324671	0.878100647
0.210172428	-0.222691536	0.073031096	0.000324671	0.878100647
0.210731382	0.345781801	0.584854032	0.031639242	0.878100647
0.210731382	0.345781801	0.584854032	0.031639242	0.878100647
0.210731382	0.345781801	0.584854032	0.031639242	0.878100647
0.210731382	0.345781801	0.584854032	0.031639242	0.878100647
0.210731382	0.345781801	0.584854032	0.031639242	0.878100647
0.210731382	0.345781801	0.584854032	0.031639242	0.878100647
0.210731382	0.345781801	0.584854032	0.031639242	0.878100647
0.210731382	0.345781801	0.584854032	0.031639242	0.878100647
0.210731382	0.345781801	0.584854032	0.031639242	0.878100647
0.211023623	0.422542663	0.737584497	0.850173656	0.878100647
0.211470277	-0.387191619	1	0.145037597	0.878100647
0.211911137	-0.404072613	1	0.242167012	0.878100647
0.211911137	-0.404072613	1	0.242167012	0.878100647
0.212006851	-0.315050362	0.439859057	0.011906673	0.878100647
0.212631754	-0.213466662	0.043105813	0.0002997	0.878100647
0.212631754	-0.213466662	0.043105813	0.0002997	0.878100647
0.212698229	0.244356322	0.104319013	0.001090387	0.878100647
0.213017647	-0.196471836	0.034012327	0.000124759	0.878100647
0.213342405	0.359100304	0.881481882	0.060775899	0.878100647
0.213342733	-0.390504052	1	0.145037597	0.878100647
0.213342733	-0.390504052	1	0.145037597	0.878100647
0.214043237	-0.127740658	0.001207877	3.06E-06	0.878100647

0.214043237	-0.127740658	0.001207877	3.06E-06	0.878100647
0.214043237	-0.127740658	0.001207877	3.06E-06	0.878100647
0.214043237	-0.127740658	0.001207877	3.06E-06	0.878100647
0.214210761	0.403810249	1	0.242167012	0.878100647
0.214390457	-0.276231715	0.295435331	0.003736177	0.878100647
0.214857427	-0.4191009	0.783270054	0.704162183	0.878100647
0.214857427	-0.4191009	0.783270054	0.704162183	0.878100647
0.214867712	-0.066526487	1.22E-05	1.63E-08	0.878100647
0.214969083	-0.124978676	0.001207877	3.06E-06	0.878100647
0.214996085	0.054708586	7.89E-09	8.55E-07	0.878100647
0.215315279	0.409695311	0.439859057	1	0.878100647
0.215373148	-0.391283578	1	0.164911511	0.878100647
0.215579402	0.415304963	0.584854032	1	0.878100647
0.215579402	0.415304963	0.584854032	1	0.878100647
0.215668803	0.225727289	0.0008771	0.060775899	0.878100647
0.215668803	0.225727289	0.0008771	0.060775899	0.878100647
0.216272345	0.349835598	0.737584497	0.052574033	0.878100647
0.216272345	0.349835598	0.737584497	0.052574033	0.878100647
0.216272345	0.349835598	0.737584497	0.052574033	0.878100647
0.216342394	0.417808962	0.737584497	0.704162183	0.878100647
0.216342394	0.417808962	0.737584497	0.704162183	0.878100647
0.216561388	-0.412260539	0.439859057	1	0.878100647
0.216764873	-0.417234311	0.881481882	0.704162183	0.878100647
0.216769342	-0.412591736	1	0.489952655	0.878100647
0.21685745	-0.381199551	1	0.145037597	0.878100647
0.217331402	-0.32201727	0.034012327	0.489952655	0.878100647
0.217331402	-0.32201727	0.034012327	0.489952655	0.878100647
0.217377821	0.416173564	0.989933981	0.704162183	0.878100647
0.217742979	-0.407673752	1	0.397942841	0.878100647
0.218076067	0.399838187	1	0.242167012	0.878100647
0.218076067	0.399838187	1	0.242167012	0.878100647
0.218109954	0.333777103	0.584854032	0.029310175	0.878100647
0.218118584	0.165880912	6.77E-05	0.006372161	0.878100647
0.218118584	0.165880912	6.77E-05	0.006372161	0.878100647
0.218245397	0.395395993	0.307400178	1	0.878100647
0.218245397	0.395395993	0.307400178	1	0.878100647
0.21835685	0.396279914	0.307400178	1	0.878100647
0.218439281	0.343391529	0.043105813	0.704162183	0.878100647
0.218439281	0.343391529	0.043105813	0.704162183	0.878100647
0.218439281	0.343391529	0.043105813	0.704162183	0.878100647
0.218439281	0.343391529	0.043105813	0.704162183	0.878100647
0.218480063	-0.260616021	0.19926681	0.002420613	0.878100647
0.218651296	-0.181659158	0.025193624	7.48E-05	0.878100647
0.218699771	0.410347438	1	0.489952655	0.878100647
0.218699771	0.410347438	1	0.489952655	0.878100647
0.218977447	0.410139684	0.439859057	1	0.878100647
0.219121872	0.344302945	0.058883576	0.704162183	0.878100647
0.219130779	0.140950375	1.22E-05	0.002093839	0.878100647
0.219287066	-0.408158452	1	0.416730107	0.878100647
0.219331641	0.392901649	1	0.242167012	0.878100647
0.21968206	-0.159708886	0.009746439	1.80E-05	0.878100647
0.21968206	-0.159708886	0.009746439	1.80E-05	0.878100647
0.219930674	-0.390930568	1	0.242167012	0.878100647
0.219965339	0.339340334	0.584854032	0.040084197	0.878100647
0.220650888	0.384358546	0.205025676	1	0.878100647
0.220709684	-0.152105274	0.008580492	1.29E-05	0.878100647
0.220709684	-0.152105274	0.008580492	1.29E-05	0.878100647
0.220741795	0.401043929	0.412603306	1	0.878100647
0.22076985	0.063224909	2.61E-08	4.11E-06	0.878100647
0.221206233	0.244762042	0.001611045	0.10273905	0.878100647
0.221293935	0.255092697	0.192696374	0.002093839	0.878100647
0.221339692	0.344114686	0.737584497	0.052574033	0.878100647
0.221466155	-0.375827783	1	0.126792252	0.878100647
0.221466155	-0.375827783	1	0.126792252	0.878100647

0.221492138	-0.400312929	1	0.364167203	0.878100647
0.22172575	-0.310177302	0.439859057	0.017403168	0.878100647
0.221856581	0.363696794	1	0.085309726	0.878100647
0.221973104	-0.410318339	0.584854032	1	0.878100647
0.221996793	0.182820151	0.000155445	0.017403168	0.878100647
0.221996793	0.182820151	0.000155445	0.017403168	0.878100647
0.221996793	0.182820151	0.000155445	0.017403168	0.878100647
0.221996793	0.182820151	0.000155445	0.017403168	0.878100647
0.222257197	0.347983182	0.737584497	0.053084396	0.878100647
0.222257197	0.347983182	0.737584497	0.053084396	0.878100647
0.222257197	0.347983182	0.737584497	0.053084396	0.878100647
0.222400405	0.153405029	6.19E-05	0.003736177	0.878100647
0.222400405	0.153405029	6.19E-05	0.003736177	0.878100647
0.222636655	0.364902757	0.104319013	1	0.878100647
0.222636655	0.364902757	0.104319013	1	0.878100647
0.22296802	-0.39614472	1	0.242167012	0.878100647
0.223779955	0.365957275	0.134257478	1	0.878100647
0.223871171	0.394519492	1	0.242167012	0.878100647
0.22433272	0.113059692	3.58E-06	0.000324671	0.878100647
0.224342011	-0.275720379	0.307400178	0.005827837	0.878100647
0.224574028	0.321002802	0.034012327	0.489952655	0.878100647
0.224621053	-0.100623799	0.000261772	5.53E-07	0.878100647
0.224710902	-0.384856451	1	0.164911511	0.878100647
0.224793753	-0.166610535	0.016023223	4.69E-05	0.878100647
0.225464489	0.196193541	0.000261772	0.025431136	0.878100647
0.225464489	0.196193541	0.000261772	0.025431136	0.878100647
0.225464489	0.196193541	0.000261772	0.025431136	0.878100647
0.22565802	0.10700237	1.23E-06	0.0002997	0.878100647
0.225785014	-0.403424861	1	0.489952655	0.878100647
0.225999418	0.384878311	1	0.217058541	0.878100647
0.226050869	0.285980343	0.016023223	0.242167012	0.878100647
0.226340693	0.165778637	6.77E-05	0.006372161	0.878100647
0.226340693	0.165778637	6.77E-05	0.006372161	0.878100647
0.226344572	0.283476315	0.349433107	0.006372161	0.878100647
0.226458872	-0.3019471	0.439859057	0.011906673	0.878100647
0.226458872	-0.3019471	0.439859057	0.011906673	0.878100647
0.226516973	0.163833081	0.016023223	4.69E-05	0.878100647
0.226723794	-0.360536355	1	0.085309726	0.878100647
0.226723794	-0.360536355	1	0.085309726	0.878100647
0.226741226	0.364527045	0.169239932	1	0.878100647
0.226742778	0.384448122	1	0.242167012	0.878100647
0.226742778	0.384448122	1	0.242167012	0.878100647
0.226898614	0.17021915	8.53E-05	0.009270366	0.878100647
0.226901822	-0.371794595	1	0.129712192	0.878100647
0.226901822	-0.371794595	1	0.129712192	0.878100647
0.227056641	-0.404591888	0.584854032	1	0.878100647
0.227056641	-0.404591888	0.584854032	1	0.878100647
0.227222374	-0.384969501	1	0.242167012	0.878100647
0.227919034	0.230386917	0.001207877	0.066020955	0.878100647
0.228214514	-0.056432244	4.58E-06	6.53E-09	0.878100647
0.228461077	0.284053033	0.016023223	0.242167012	0.878100647
0.228830955	0.406427855	0.691111318	1	0.878100647
0.228974374	-0.392069674	1	0.305102445	0.878100647
0.228974374	-0.392069674	1	0.305102445	0.878100647
0.229012396	0.218566913	0.0008771	0.053084396	0.878100647
0.229123698	-0.400705242	0.439859057	1	0.878100647
0.229266206	-0.37219082	1	0.145037597	0.878100647
0.229343201	0.326806795	0.043105813	0.601859652	0.878100647
0.229343201	0.326806795	0.043105813	0.601859652	0.878100647
0.229343201	0.326806795	0.043105813	0.601859652	0.878100647
0.229343201	0.326806795	0.043105813	0.601859652	0.878100647
0.229343201	0.326806795	0.043105813	0.601859652	0.878100647
0.229343201	0.326806795	0.043105813	0.601859652	0.878100647
0.229455607	-0.084554526	6.77E-05	1.54E-07	0.878100647

0.230987984	0.356210222	0.104319013	1	0.878100647
0.230987984	0.356210222	0.104319013	1	0.878100647
0.231263672	-0.120056035	0.0008771	3.06E-06	0.878100647
0.231263672	-0.120056035	0.0008771	3.06E-06	0.878100647
0.231530285	-0.398965346	1	0.489952655	0.878100647
0.231670563	0.154181873	6.19E-05	0.005827837	0.878100647
0.231670563	0.154181873	6.19E-05	0.005827837	0.878100647
0.231758068	-0.18979152	0.034012327	0.000124759	0.878100647
0.231758068	-0.18979152	0.034012327	0.000124759	0.878100647
0.231758068	-0.18979152	0.034012327	0.000124759	0.878100647
0.231758068	-0.18979152	0.034012327	0.000124759	0.878100647
0.231987829	0.400281226	0.584854032	1	0.878100647
0.231987829	0.400281226	0.584854032	1	0.878100647
0.232139942	0.401742286	1	0.489952655	0.878100647
0.232139942	0.401742286	1	0.489952655	0.878100647
0.232215363	-0.138981355	0.004273917	9.47E-06	0.878100647
0.232255991	-0.257712507	0.205025676	0.002721298	0.878100647
0.232255991	-0.257712507	0.205025676	0.002721298	0.878100647
0.232436349	0.383844937	1	0.242167012	0.878100647
0.232630992	-0.099293939	0.000261772	5.53E-07	0.878100647
0.232803659	0.368456869	1	0.145037597	0.878100647
0.23295861	0.280479306	0.349433107	0.006372161	0.878100647
0.23300361	-0.302512818	0.439859057	0.017403168	0.878100647
0.23300361	-0.302512818	0.439859057	0.017403168	0.878100647
0.233091532	-0.324913507	0.584854032	0.031639242	0.878100647
0.233486803	-0.263662525	0.205025676	0.003736177	0.878100647
0.233486803	-0.263662525	0.205025676	0.003736177	0.878100647
0.233501064	0.289824826	0.412603306	0.009270366	0.878100647
0.233617846	0.03948389	1.75E-09	1.54E-07	0.878100647
0.233617846	0.03948389	1.75E-09	1.54E-07	0.878100647
0.233881296	0.394510445	0.439859057	1	0.878100647
0.233881296	0.394510445	0.439859057	1	0.878100647
0.233881296	0.394510445	0.439859057	1	0.878100647
0.233881296	0.394510445	0.439859057	1	0.878100647
0.233909644	0.237102185	0.001611045	0.085309726	0.878100647
0.233909644	0.237102185	0.001611045	0.085309726	0.878100647
0.234118196	0.240887741	0.003169634	0.102782106	0.878100647
0.234274052	0.397815013	1	0.489952655	0.878100647
0.234528033	-0.40039347	1	0.601859652	0.878100647
0.234612914	-0.379241974	0.307400178	1	0.878100647
0.234612914	-0.379241974	0.307400178	1	0.878100647
0.234672405	0.184580271	0.034012327	0.000124759	0.878100647
0.235266351	-0.2664446	0.295435331	0.004171959	0.878100647
0.235266351	-0.2664446	0.295435331	0.004171959	0.878100647
0.235503015	0.117502115	4.58E-06	0.000596242	0.878100647
0.235503015	0.117502115	4.58E-06	0.000596242	0.878100647
0.235758184	-0.401162393	0.814576593	0.850173656	0.878100647
0.235758184	-0.401162393	0.814576593	0.850173656	0.878100647
0.23595476	0.289440445	0.412603306	0.009270366	0.878100647
0.236097999	-0.236975401	0.134257478	0.001791344	0.878100647
0.236327306	-0.39108527	1	0.397942841	0.878100647
0.236897417	0.244132231	0.00373662	0.126792252	0.878100647
0.237082443	0.07345376	9.36E-08	1.65E-05	0.878100647
0.237082443	0.07345376	9.36E-08	1.65E-05	0.878100647
0.237232385	0.396842843	1	0.489952655	0.878100647
0.237232385	0.396842843	1	0.489952655	0.878100647
0.237424146	-0.143385596	0.004526275	1.04E-05	0.878100647
0.237624026	0.364867721	1	0.145037597	0.878100647
0.237714896	-0.126244064	0.001207877	4.11E-06	0.878100647
0.237714896	-0.126244064	0.001207877	4.11E-06	0.878100647
0.237872482	0.096412131	1.09E-06	0.000124759	0.878100647
0.237872482	0.096412131	1.09E-06	0.000124759	0.878100647
0.237872482	0.096412131	1.09E-06	0.000124759	0.878100647
0.238600318	-0.328201707	0.584854032	0.045751002	0.878100647

0.238643554	-0.187908047	0.034012327	0.000124759	0.878100647
0.238643554	-0.187908047	0.034012327	0.000124759	0.878100647
0.238661065	0.38145429	0.412603306	1	0.878100647
0.238763706	-0.323126348	0.584854032	0.031639242	0.878100647
0.238962871	0.379544583	0.349433107	1	0.878100647
0.239002939	-0.173124182	0.024201521	7.48E-05	0.878100647
0.23902552	-0.227881395	0.095538718	0.001090387	0.878100647
0.23904756	-0.397721048	1	0.704162183	0.878100647
0.239326111	0.033790234	1.44E-09	5.53E-08	0.878100647
0.239326111	0.033790234	1.44E-09	5.53E-08	0.878100647
0.239326111	0.033790234	1.44E-09	5.53E-08	0.878100647
0.239326111	0.033790234	1.44E-09	5.53E-08	0.878100647
0.239333751	0.10912631	3.58E-06	0.000324671	0.878100647
0.239544922	0.288106834	0.412603306	0.011906673	0.878100647
0.239794197	0.397800713	0.737584497	0.850173656	0.878100647
0.239889177	0.37845368	1	0.242167012	0.878100647
0.239933416	0.176487809	0.000189446	0.011906673	0.878100647
0.240026643	-0.389068645	0.439859057	1	0.878100647
0.240132297	0.362383198	0.19926681	1	0.878100647
0.24013824	0.313630638	0.03951775	0.489952655	0.878100647
0.240293033	-0.202753195	0.043105813	0.000324671	0.878100647
0.240293033	-0.202753195	0.043105813	0.000324671	0.878100647
0.240293033	-0.202753195	0.043105813	0.000324671	0.878100647
0.240293033	-0.202753195	0.043105813	0.000324671	0.878100647
0.240734556	0.270020967	0.307400178	0.006372161	0.878100647
0.240734556	0.270020967	0.307400178	0.006372161	0.878100647
0.241346574	0.202160374	0.0008771	0.031639242	0.878100647
0.241538288	-0.378318274	1	0.242167012	0.878100647
0.241538288	-0.378318274	1	0.242167012	0.878100647
0.24161012	0.394885193	1	0.645935818	0.878100647
0.241682643	0.350767885	1	0.10273905	0.878100647
0.241891011	0.348806722	1	0.085309726	0.878100647
0.241891011	0.348806722	1	0.085309726	0.878100647
0.242384383	-0.394133384	1	0.645935818	0.878100647
0.242384383	-0.394133384	1	0.645935818	0.878100647
0.242853866	0.167191762	8.53E-05	0.009270366	0.878100647
0.243062623	-0.393146025	1	0.601859652	0.878100647
0.243062623	-0.393146025	1	0.601859652	0.878100647
0.243062623	-0.393146025	1	0.601859652	0.878100647
0.243092463	0.377150109	1	0.242167012	0.878100647
0.243312791	0.389412671	0.439859057	1	0.878100647
0.243374809	0.335662463	0.737584497	0.060775899	0.878100647
0.243374809	0.335662463	0.737584497	0.060775899	0.878100647
0.243374809	0.335662463	0.737584497	0.060775899	0.878100647
0.243985058	-0.384217001	1	0.397942841	0.878100647
0.244396885	-0.202509556	0.043105813	0.000324671	0.878100647
0.244735134	-0.393424737	0.989933981	0.704162183	0.878100647
0.244795231	0.228751438	0.095538718	0.001791344	0.878100647
0.244923079	0.183128313	0.000261772	0.02341474	0.878100647
0.244923079	0.183128313	0.000261772	0.02341474	0.878100647
0.245360559	0.337292891	0.095006685	0.850173656	0.878100647
0.245360559	0.337292891	0.095006685	0.850173656	0.878100647
0.246002679	0.391316584	0.584854032	1	0.878100647
0.24647212	-0.235374714	0.134257478	0.002093839	0.878100647
0.246495578	0.206142875	0.0008771	0.042826979	0.878100647
0.246588375	0.379389789	0.412603306	1	0.878100647
0.246599	0.332723612	0.087427713	0.704162183	0.878100647
0.246669981	0.338908706	0.881481882	0.085309726	0.878100647
0.246945694	0.391971447	0.881481882	0.850173656	0.878100647
0.247018239	0.391789488	0.737584497	1	0.878100647
0.247019719	0.128384269	0.002297238	5.99E-06	0.878100647
0.247198156	0.31704461	0.043105813	0.601859652	0.878100647
0.247297494	-0.171004226	0.024201521	0.000115129	0.878100647
0.247332891	0.185099424	0.000261772	0.02341474	0.878100647

0.247332891	0.185099424	0.000261772	0.02341474	0.878100647
0.247395382	-0.314211603	0.584854032	0.031639242	0.878100647
0.247480782	0.272185373	0.34036444	0.006372161	0.878100647
0.247688998	0.343246733	0.989933981	0.085309726	0.878100647
0.247709099	0.384165522	0.439859057	1	0.878100647
0.247839236	0.264447328	0.010835964	0.242167012	0.878100647
0.24805702	0.267254621	0.307400178	0.006372161	0.878100647
0.24805702	0.267254621	0.307400178	0.006372161	0.878100647
0.248097702	0.312058205	0.584854032	0.031639242	0.878100647
0.248097702	0.312058205	0.584854032	0.031639242	0.878100647
0.248321088	0.172684779	0.000228282	0.011906673	0.878100647
0.248321088	0.172684779	0.000228282	0.011906673	0.878100647
0.248967818	0.320398035	0.584854032	0.045751002	0.878100647
0.249385838	0.382628998	0.439859057	1	0.878100647
0.249385838	0.382628998	0.439859057	1	0.878100647
0.24955985	-0.37686889	1	0.364167203	0.878100647
0.249859719	-0.303918842	0.439859057	0.025431136	0.878100647
0.249859719	-0.303918842	0.439859057	0.025431136	0.878100647
0.249859719	-0.303918842	0.439859057	0.025431136	0.878100647
0.249859719	-0.303918842	0.439859057	0.025431136	0.878100647
0.249923057	0.360974185	1	0.164911511	0.878100647
0.249988558	-0.085943589	0.000153763	2.98E-07	0.878100647
0.250094557	0.10425847	2.89E-06	0.0002997	0.878100647
0.250430365	-0.376157483	0.412603306	1	0.878100647
0.250504777	-0.236493196	0.169239932	0.002093839	0.878100647
0.250504777	-0.236493196	0.169239932	0.002093839	0.878100647
0.251119287	-0.341469999	0.989933981	0.085309726	0.878100647
0.251197218	0.385395389	1	0.489952655	0.878100647
0.251202426	0.267736953	0.307400178	0.006372161	0.878100647
0.25153129	0.114550382	0.0008771	3.06E-06	0.878100647
0.25153129	0.114550382	0.0008771	3.06E-06	0.878100647
0.251541352	0.263302826	0.010835964	0.242167012	0.878100647
0.251606047	0.380063991	1	0.416730107	0.878100647
0.251652978	0.380950695	0.439859057	1	0.878100647
0.252029149	0.305544574	0.584854032	0.029310175	0.878100647
0.252443871	-0.124356127	0.001207877	5.99E-06	0.878100647
0.252443871	-0.124356127	0.001207877	5.99E-06	0.878100647
0.252630181	0.089478249	1.09E-06	0.000115129	0.878100647
0.252864038	-0.176178953	0.030989574	0.000124759	0.878100647
0.252956828	0.293065786	0.439859057	0.02341474	0.878100647
0.253000969	-0.38664164	1	0.704162183	0.878100647
0.253026964	-0.374594436	1	0.364167203	0.878100647
0.253295231	0.386589196	1	0.704162183	0.878100647
0.25341489	0.321762902	0.691111318	0.053084396	0.878100647
0.253737058	-0.229149868	0.104319013	0.001791344	0.878100647
0.253737058	-0.229149868	0.104319013	0.001791344	0.878100647
0.253776199	0.330033561	0.737584497	0.061934943	0.878100647
0.253776199	0.330033561	0.737584497	0.061934943	0.878100647
0.253910658	0.338800812	0.120559922	1	0.878100647
0.254015335	-0.384003193	0.584854032	1	0.878100647
0.254058877	-0.35967091	1	0.164911511	0.878100647
0.254173505	0.364845946	1	0.242167012	0.878100647
0.254667089	0.287653717	0.030989574	0.397942841	0.878100647
0.254919055	0.257918558	0.010835964	0.164911511	0.878100647
0.255310857	0.38374287	1	0.704162183	0.878100647
0.255521606	0.07861662	3.18E-07	4.69E-05	0.878100647
0.255958066	0.37570412	0.439859057	1	0.878100647
0.256124458	0.305737969	0.584854032	0.031639242	0.878100647
0.256175699	0.348272839	1	0.145037597	0.878100647
0.256175699	0.348272839	1	0.145037597	0.878100647
0.256175699	0.348272839	1	0.145037597	0.878100647
0.256242085	0.214192459	0.001207877	0.060775899	0.878100647
0.256242085	0.214192459	0.001207877	0.060775899	0.878100647
0.256242085	0.214192459	0.001207877	0.060775899	0.878100647

0.256328952	0.332678453	0.881481882	0.085309726	0.878100647
0.256496065	0.38007698	1	0.489952655	0.878100647
0.25674464	0.238993794	0.004526275	0.126792252	0.878100647
0.256853977	0.320831034	0.075374611	0.704162183	0.878100647
0.256853977	0.320831034	0.075374611	0.704162183	0.878100647
0.256853977	0.320831034	0.075374611	0.704162183	0.878100647
0.256853977	0.320831034	0.075374611	0.704162183	0.878100647
0.256985083	0.37643065	0.439859057	1	0.878100647
0.256985083	0.37643065	0.439859057	1	0.878100647
0.257694327	-0.311701565	0.584854032	0.042826979	0.878100647
0.257694327	-0.311701565	0.584854032	0.042826979	0.878100647
0.257694327	-0.311701565	0.584854032	0.042826979	0.878100647
0.258026916	-0.17076726	0.025193624	0.000115129	0.878100647
0.258532149	0.315912208	0.584854032	0.052574033	0.878100647
0.258541972	-0.142185698	0.008580492	1.80E-05	0.878100647
0.258541972	-0.142185698	0.008580492	1.80E-05	0.878100647
0.258669718	0.319602049	0.691111318	0.060775899	0.878100647
0.258731571	-0.101003817	0.000467492	8.55E-07	0.878100647
0.258844076	0.379797348	0.584854032	1	0.878100647
0.259423223	-0.306822224	0.584854032	0.031639242	0.878100647
0.259509955	0.217024158	0.095006685	0.001090387	0.878100647
0.259509955	0.217024158	0.095006685	0.001090387	0.878100647
0.259509955	0.217024158	0.095006685	0.001090387	0.878100647
0.259588975	0.338991119	0.169239932	1	0.878100647
0.259650068	0.38170028	0.737584497	1	0.878100647
0.259650068	0.38170028	0.737584497	1	0.878100647
0.259650068	0.38170028	0.737584497	1	0.878100647
0.260728417	0.376296737	1	0.489952655	0.878100647
0.260802656	-0.148976473	0.009746439	4.69E-05	0.878100647
0.261115894	0.327040049	0.737584497	0.06824239	0.878100647
0.261115894	0.327040049	0.737584497	0.06824239	0.878100647
0.261115894	0.327040049	0.737584497	0.06824239	0.878100647
0.261115894	0.327040049	0.737584497	0.06824239	0.878100647
0.261115894	0.327040049	0.737584497	0.06824239	0.878100647
0.261115894	0.327040049	0.737584497	0.06824239	0.878100647
0.261327231	0.17094504	0.000249829	0.011906673	0.878100647
0.261336069	-0.380384922	0.989933981	0.704162183	0.878100647
0.261588539	0.210935879	0.001207877	0.060775899	0.878100647
0.261588539	0.210935879	0.001207877	0.060775899	0.878100647
0.261642049	0.353230809	1	0.164911511	0.878100647
0.26173675	0.210571532	0.075374611	0.001090387	0.878100647
0.262305768	0.232046199	0.169239932	0.002420613	0.878100647
0.262551193	-0.234563445	0.169239932	0.002420613	0.878100647
0.262551193	-0.234563445	0.169239932	0.002420613	0.878100647
0.262788978	-0.152864574	6.77E-05	0.006372161	0.878100647
0.263066874	-0.101301723	0.000467492	8.55E-07	0.878100647
0.263066874	-0.101301723	0.000467492	8.55E-07	0.878100647
0.263066874	-0.101301723	0.000467492	8.55E-07	0.878100647
0.263066874	-0.101301723	0.000467492	8.55E-07	0.878100647
0.263250598	-0.222811828	0.095538718	0.001791344	0.878100647
0.26339159	-0.330041334	0.104319013	1	0.878100647
0.263467191	-0.143363249	0.008580492	4.35E-05	0.878100647
0.263467191	-0.143363249	0.008580492	4.35E-05	0.878100647
0.263467191	-0.143363249	0.008580492	4.35E-05	0.878100647
0.263721505	0.378590634	0.989933981	0.850173656	0.878100647
0.263721505	0.378590634	0.989933981	0.850173656	0.878100647
0.263747789	0.373578053	1	0.489952655	0.878100647
0.263747789	0.373578053	1	0.489952655	0.878100647
0.263958007	-0.372720734	1	0.489952655	0.878100647
0.264149264	0.243092851	0.19926681	0.003736177	0.878100647
0.264149264	0.243092851	0.19926681	0.003736177	0.878100647
0.264610318	-0.367295888	0.439859057	1	0.878100647
0.264662319	-0.265009918	0.34036444	0.009270366	0.878100647
0.264662319	-0.265009918	0.34036444	0.009270366	0.878100647

0.264715587	-0.296951509	0.439859057	0.029310175	0.878100647
0.264736038	0.127053085	1.56E-05	0.001791344	0.878100647
0.264854673	-0.356261185	1	0.242167012	0.878100647
0.264901189	0.293623147	0.439859057	0.025431136	0.878100647
0.2649235	-0.372451943	0.493333254	1	0.878100647
0.265147111	-0.329955351	0.120559922	1	0.878100647
0.265263616	0.138071352	0.004526275	1.65E-05	0.878100647
0.26534533	-0.368273287	0.439859057	1	0.878100647
0.265625146	-0.305382511	0.584854032	0.042826979	0.878100647
0.265657485	0.316451002	0.075374611	0.704162183	0.878100647
0.265741505	-0.376630763	1	0.704162183	0.878100647
0.265929512	0.336590507	1	0.126792252	0.878100647
0.265929512	0.336590507	1	0.126792252	0.878100647
0.266516266	0.329446042	0.134257478	1	0.878100647
0.266516266	0.329446042	0.134257478	1	0.878100647
0.266599991	0.200143508	0.0008771	0.045751002	0.878100647
0.266599991	0.200143508	0.0008771	0.045751002	0.878100647
0.26660091	-0.133751743	0.004273917	1.65E-05	0.878100647
0.26660091	-0.133751743	0.004273917	1.65E-05	0.878100647
0.26661086	0.285724344	0.439859057	0.02341474	0.878100647
0.26661086	0.285724344	0.439859057	0.02341474	0.878100647
0.26661086	0.285724344	0.439859057	0.02341474	0.878100647
0.266652969	-0.31868511	0.737584497	0.061934943	0.878100647
0.266652969	-0.31868511	0.737584497	0.061934943	0.878100647
0.266652969	-0.31868511	0.737584497	0.061934943	0.878100647
0.266685164	0.139849098	6.19E-05	0.003736177	0.878100647
0.266833997	-0.160324611	0.018163994	0.000108916	0.878100647
0.26692462	0.355096234	0.307400178	1	0.878100647
0.266977502	0.235625467	0.004526275	0.126792252	0.878100647
0.266977502	0.235625467	0.004526275	0.126792252	0.878100647
0.267178457	-0.349329406	1	0.164911511	0.878100647
0.267178457	-0.349329406	1	0.164911511	0.878100647
0.267441332	0.253020648	0.295435331	0.006372161	0.878100647
0.267643815	-0.368065817	1	0.489952655	0.878100647
0.267662274	0.369504962	0.439859057	1	0.878100647
0.267662274	0.369504962	0.439859057	1	0.878100647
0.267847236	-0.249051471	0.205025676	0.005827837	0.878100647
0.267914242	0.32692125	0.881481882	0.085309726	0.878100647
0.268284884	0.361680687	0.412603306	1	0.878100647
0.268507389	0.371069964	1	0.489952655	0.878100647
0.268534821	-0.375006749	0.881481882	1	0.878100647
0.268544806	-0.373759163	1	0.704162183	0.878100647
0.268595875	-0.098461427	0.000467492	8.55E-07	0.878100647
0.268653947	0.291777236	0.439859057	0.025431136	0.878100647
0.268689914	0.342440313	0.205025676	1	0.878100647
0.269386518	-0.371486553	1	0.601859652	0.878100647
0.269429035	0.344099293	1	0.145037597	0.878100647
0.269429035	0.344099293	1	0.145037597	0.878100647
0.269429035	0.344099293	1	0.145037597	0.878100647
0.269429035	0.344099293	1	0.145037597	0.878100647
0.269429035	0.344099293	1	0.145037597	0.878100647
0.269429035	0.344099293	1	0.145037597	0.878100647
0.269523706	-0.109103617	0.0008771	3.06E-06	0.878100647
0.269523706	-0.109103617	0.0008771	3.06E-06	0.878100647
0.269637393	0.370419815	0.584854032	1	0.878100647
0.269637393	0.370419815	0.584854032	1	0.878100647
0.269868678	0.373523259	1	0.704162183	0.878100647
0.269868678	0.373523259	1	0.704162183	0.878100647
0.269992692	0.345405926	1	0.164911511	0.878100647
0.27005528	-0.185328349	0.035148121	0.000324671	0.878100647
0.270131587	-0.34417921	1	0.145037597	0.878100647
0.270479132	-0.357514673	1	0.305102445	0.878100647
0.270479132	-0.357514673	1	0.305102445	0.878100647
0.270563584	-0.34012244	1	0.145037597	0.878100647

0.270937674	0.066493653	9.36E-08	9.47E-06	0.878100647
0.271451429	0.293022993	0.043105813	0.489952655	0.878100647
0.271474512	-0.129219863	0.00373662	1.04E-05	0.878100647
0.271533506	0.276992675	0.412603306	0.02341474	0.878100647
0.271533506	0.276992675	0.412603306	0.02341474	0.878100647
0.271626214	0.05405925	2.61E-08	3.06E-06	0.878100647
0.271626214	0.05405925	2.61E-08	3.06E-06	0.878100647
0.271626214	0.05405925	2.61E-08	3.06E-06	0.878100647
0.271754365	0.132938869	0.004273917	1.65E-05	0.8914411
0.271754365	0.132938869	0.004273917	1.65E-05	0.8914411
0.271901808	0.31224506	0.691111318	0.060775899	0.8914411
0.272301508	-0.350854879	0.307400178	1	0.8914411
0.272383512	0.309186011	0.584854032	0.060775899	0.8914411
0.272584772	-0.300239263	0.584854032	0.042826979	0.8914411
0.272732535	0.371558969	1	0.704162183	0.8914411
0.273366531	-0.237551733	0.19926681	0.003736177	0.8914411
0.273560155	0.295236732	0.043105813	0.489952655	0.8914411
0.273625793	-0.281660628	0.439859057	0.02341474	0.8914411
0.273627173	0.368413216	1	0.601859652	0.8914411
0.273627173	0.368413216	1	0.601859652	0.8914411
0.273954994	-0.369871772	1	0.704162183	0.8914411
0.274506068	0.247802581	0.010835964	0.164911511	0.8914411
0.274750073	0.215935732	0.095538718	0.001791344	0.8914411
0.274750073	0.215935732	0.095538718	0.001791344	0.8914411
0.274889604	0.23331005	0.192696374	0.003736177	0.8914411
0.275251132	-0.289624381	0.439859057	0.029310175	0.8914411
0.275373419	0.166472374	0.000261772	0.011906673	0.8914411
0.275408335	0.165962172	0.000249829	0.011906673	0.8914411
0.276126427	0.17630592	0.000467492	0.02341474	0.8914411
0.276126427	0.17630592	0.000467492	0.02341474	0.8914411
0.276671937	0.084479086	1.09E-06	7.48E-05	0.8914411
0.276895662	0.218182863	0.095538718	0.002093839	0.8914411
0.276895662	0.218182863	0.095538718	0.002093839	0.8914411
0.276895662	0.218182863	0.095538718	0.002093839	0.8914411
0.277025808	0.27740878	0.034012327	0.397942841	0.8914411
0.277203188	-0.161361884	0.018394332	0.000115129	0.8914411
0.277203188	-0.161361884	0.018394332	0.000115129	0.8914411
0.277525356	0.236854954	0.19926681	0.004171959	0.8914411
0.27840638	0.263460881	0.024201521	0.242167012	0.8914411
0.278552453	-0.152015879	0.016023223	6.41E-05	0.8914411
0.27883096	-0.361743043	1	0.489952655	0.8914411
0.27885399	0.307804261	0.691111318	0.060775899	0.8914411
0.279236467	-0.206635094	0.075374611	0.001090387	0.8914411
0.279524245	0.074242128	3.18E-07	4.69E-05	0.8914411
0.279813246	-0.223576059	0.169239932	0.002420613	0.8914411
0.279813246	-0.223576059	0.169239932	0.002420613	0.8914411
0.279813246	-0.223576059	0.169239932	0.002420613	0.8914411
0.280005894	-0.126238258	0.003169634	1.04E-05	0.8914411
0.280129649	-0.35338217	0.439859057	1	0.8914411
0.28029383	0.145664557	6.77E-05	0.005827837	0.8914411
0.28029383	0.145664557	6.77E-05	0.005827837	0.8914411
0.280691329	0.064274123	9.36E-08	9.47E-06	0.8914411
0.280691329	0.064274123	9.36E-08	9.47E-06	0.8914411
0.28077481	-0.117968026	0.001207877	9.47E-06	0.8914411
0.28077481	-0.117968026	0.001207877	9.47E-06	0.8914411
0.2813078	-0.362899918	0.584854032	1	0.8914411
0.281342048	-0.36521071	0.737584497	1	0.8914411
0.281367452	-0.305244791	0.584854032	0.060775899	0.8914411
0.281524695	0.249028399	0.295435331	0.006372161	0.8914411
0.281524695	0.249028399	0.295435331	0.006372161	0.8914411
0.281907087	-0.356236133	0.439859057	1	0.8914411
0.281907087	-0.356236133	0.439859057	1	0.8914411
0.282033408	-0.3516396	1	0.364167203	0.8914411
0.282072235	0.096663052	3.58E-06	0.0002997	0.8914411

0.282092308	-0.364176058	0.737584497	1	0.8914411
0.282281809	-0.357227698	1	0.489952655	0.8914411
0.282614565	0.35908576	1	0.489952655	0.8914411
0.282720736	-0.221809655	0.134257478	0.002420613	0.8914411
0.282720736	-0.221809655	0.134257478	0.002420613	0.8914411
0.282805532	0.265206863	0.030989574	0.305102445	0.8914411
0.282805532	0.265206863	0.030989574	0.305102445	0.8914411
0.282904692	-0.29306578	0.584854032	0.042826979	0.8914411
0.283081145	0.362473506	0.584854032	1	0.8914411
0.2831026	-0.123784585	0.003169634	9.47E-06	0.8914411
0.283691395	0.325557915	0.192696374	1	0.8914411
0.283691395	0.325557915	0.192696374	1	0.8914411
0.283939883	-0.276507022	0.439859057	0.025431136	0.8914411
0.283939883	-0.276507022	0.439859057	0.025431136	0.8914411
0.284037161	0.30605552	0.095006685	0.704162183	0.8914411
0.284037161	0.30605552	0.095006685	0.704162183	0.8914411
0.284078361	-0.106747978	0.0008771	3.47E-06	0.8914411
0.28416987	-0.359350575	0.584854032	1	0.8914411
0.284172874	-0.34551581	1	0.242167012	0.8914411
0.284231683	-0.356289792	1	0.489952655	0.8914411
0.284231683	-0.356289792	1	0.489952655	0.8914411
0.284291267	-0.363175681	0.989933981	1	0.8914411
0.28434903	-0.362727505	1	0.704162183	0.8914411
0.284610052	0.362921306	0.881481882	1	0.8914411
0.284752188	-0.217005831	0.104319013	0.002093839	0.8914411
0.285061414	0.174867258	0.000467492	0.02341474	0.8914411
0.285210602	0.313533672	0.737584497	0.085309726	0.8914411
0.285644707	-0.307918668	0.737584497	0.066020955	0.8914411
0.285878447	0.286356174	0.439859057	0.031639242	0.8914411
0.285878447	0.286356174	0.439859057	0.031639242	0.8914411
0.285878776	0.35477983	1	0.489952655	0.8914411
0.286015971	0.289708133	0.584854032	0.041232736	0.8914411
0.286015971	0.289708133	0.584854032	0.041232736	0.8914411
0.286068746	0.275916678	0.439859057	0.025431136	0.8914411
0.286068746	0.275916678	0.439859057	0.025431136	0.8914411
0.286137466	0.243396601	0.273258425	0.006372161	0.8914411
0.286807757	0.297280679	0.075374611	0.601859652	0.8914411
0.28703494	0.332081581	1	0.164911511	0.8914411
0.287204973	-0.069250883	6.19E-05	9.79E-08	0.8914411
0.287390731	0.261626378	0.412603306	0.011906673	0.8914411
0.287581193	0.349427709	0.439859057	1	0.8914411
0.288240822	0.140756243	0.00974133	4.69E-05	0.8914411
0.288242242	0.314808366	0.134257478	0.850173656	0.8914411
0.288280937	0.358860801	1	0.704162183	0.8914411
0.288280937	0.358860801	1	0.704162183	0.8914411
0.288280937	0.358860801	1	0.704162183	0.8914411
0.288280937	0.358860801	1	0.704162183	0.8914411
0.288280937	0.358860801	1	0.704162183	0.8914411
0.288280937	0.358860801	1	0.704162183	0.8914411
0.288385842	0.151590133	0.000155445	0.006372161	0.8914411
0.288385842	0.151590133	0.000155445	0.006372161	0.8914411
0.288420992	0.329155818	1	0.145037597	0.8914411
0.28861013	-0.236445444	0.205025676	0.005827837	0.8914411
0.28861013	-0.236445444	0.205025676	0.005827837	0.8914411
0.288800465	0.188178978	0.043105813	0.000596242	0.8914411
0.288800465	0.188178978	0.043105813	0.000596242	0.8914411
0.288903549	0.145204707	8.53E-05	0.006372161	0.8914411
0.290191154	0.357913103	0.737584497	1	0.8914411
0.290285009	-0.331350705	1	0.164911511	0.8914411
0.290435369	0.342618652	0.412603306	1	0.8914411
0.290529001	0.142925284	8.53E-05	0.005827837	0.8914411
0.290529001	0.142925284	8.53E-05	0.005827837	0.8914411
0.290540493	0.058040131	4.36E-08	4.11E-06	0.8914411
0.290540493	0.058040131	4.36E-08	4.11E-06	0.8914411

0.290643218	0.119412609	1.56E-05	0.001791344	0.8914411
0.290662713	-0.220752309	0.169239932	0.002420613	0.8914411
0.291141267	0.207499282	0.003169634	0.066020955	0.8914411
0.291147232	0.265971707	0.034012327	0.364167203	0.8914411
0.291147232	0.265971707	0.034012327	0.364167203	0.8914411
0.292339597	-0.35478432	1	0.645935818	0.8914411
0.29249162	0.313797543	0.881481882	0.126792252	0.8914411
0.292750808	0.326683057	0.205025676	1	0.8914411
0.292750808	0.326683057	0.205025676	1	0.8914411
0.292956599	0.262051298	0.412603306	0.02341474	0.8914411
0.293147283	0.193872178	0.001207877	0.052574033	0.8914411
0.293267013	-0.178258159	0.035148121	0.000324671	0.8914411
0.293663452	-0.182084411	0.043105813	0.000596242	0.8914411
0.293663452	-0.182084411	0.043105813	0.000596242	0.8914411
0.293722717	-0.237671412	0.205025676	0.006372161	0.8914411
0.293968851	0.296523652	0.075374611	0.704162183	0.8914411
0.294387817	0.353625914	1	0.704162183	0.8914411
0.294408259	-0.320294959	1	0.145037597	0.8914411
0.294442438	0.345170523	0.439859057	1	0.8914411
0.294722965	0.181303591	0.0008771	0.029310175	0.8914411
0.295198606	0.202607583	0.087427713	0.001791344	0.8914411
0.295198606	0.202607583	0.087427713	0.001791344	0.8914411
0.295227302	-0.325587274	1	0.164911511	0.8914411
0.295227302	-0.325587274	1	0.164911511	0.8914411
0.295291202	0.347204986	1	0.489952655	0.8914411
0.29555025	0.340849411	1	0.364167203	0.8914411
0.295988553	0.285018181	0.584854032	0.042826979	0.8914411
0.295988553	0.285018181	0.584854032	0.042826979	0.8914411
0.296064418	-0.261490861	0.412603306	0.02341474	0.8914411
0.29624054	-0.142274074	0.009746439	4.69E-05	0.8914411
0.296258245	0.352748787	0.684045313	1	0.8914411
0.296266866	0.312924248	0.169239932	1	0.8914411
0.296530238	0.197347362	0.075374611	0.001090387	0.8914411
0.296604407	-0.352754671	1	0.704162183	0.8914411
0.296604407	-0.352754671	1	0.704162183	0.8914411
0.296834424	0.319277015	0.19926681	1	0.8914411
0.296873202	0.077396492	4.10E-07	4.69E-05	0.8914411
0.296952007	0.323834922	0.205025676	1	0.8914411
0.297128477	-0.352122931	0.691111318	1	0.8914411
0.29724537	-0.348244705	0.584854032	1	0.8914411
0.29724537	-0.348244705	0.584854032	1	0.8914411
0.297438737	-0.161506566	0.025193624	0.000163543	0.8914411
0.297586923	-0.353357382	1	0.850173656	0.8914411
0.297586923	-0.353357382	1	0.850173656	0.8914411
0.297591335	-0.34704382	0.439859057	1	0.8914411
0.297591335	-0.34704382	0.439859057	1	0.8914411
0.297697035	-0.353433703	0.88322726	1	0.8914411
0.297697035	-0.353433703	0.88322726	1	0.8914411
0.298406678	0.304661327	0.104319013	0.704162183	0.8914411
0.298406678	0.304661327	0.104319013	0.704162183	0.8914411
0.298440113	-0.262279117	0.412603306	0.02341474	0.8914411
0.29894914	-0.340948901	1	0.397942841	0.8914411
0.29894914	-0.340948901	1	0.397942841	0.8914411
0.299041879	0.224133964	0.192696374	0.003736177	0.8914411
0.299041879	0.224133964	0.192696374	0.003736177	0.8914411
0.299302341	-0.288803543	0.075374611	0.601859652	0.8914411
0.299413568	0.291661781	0.584854032	0.060775899	0.8914411
0.299514764	0.151616067	0.000245628	0.009270366	0.8914411
0.299514764	0.151616067	0.000245628	0.009270366	0.8914411
0.2996003	-0.265530063	0.439859057	0.025431136	0.8914411
0.2996003	-0.265530063	0.439859057	0.025431136	0.8914411
0.300428896	0.347229864	1	0.489952655	0.8914411
0.300602189	0.055828787	4.36E-08	3.66E-06	0.8914411
0.300602189	0.055828787	4.36E-08	3.66E-06	0.8914411

0.300624355	0.328489303	1	0.242167012	0.8914411
0.300777425	-0.343366107	1	0.489952655	0.8914411
0.301016871	0.294019023	0.087427713	0.704162183	0.8914411
0.301016871	0.294019023	0.087427713	0.704162183	0.8914411
0.301016871	0.294019023	0.087427713	0.704162183	0.8914411
0.301251402	0.346196534	0.584854032	1	0.8914411
0.301251402	0.346196534	0.584854032	1	0.8914411
0.301292496	0.171155709	0.0008771	0.025431136	0.8914411
0.301299669	0.25989608	0.412603306	0.02341474	0.8914411
0.30163586	0.341801363	1	0.416730107	0.8914411
0.30163586	0.341801363	1	0.416730107	0.8914411
0.301744612	-0.345672092	1	0.489952655	0.8914411
0.301744612	-0.345672092	1	0.489952655	0.8914411
0.302069845	0.210317608	0.104319013	0.002420613	0.8914411
0.302480671	-0.347217129	1	0.645935818	0.8914411
0.302567266	0.287232782	0.584854032	0.060775899	0.8914411
0.302567266	0.287232782	0.584854032	0.060775899	0.8914411
0.302567266	0.287232782	0.584854032	0.060775899	0.8914411
0.302567266	0.287232782	0.584854032	0.060775899	0.8914411
0.302567266	0.287232782	0.584854032	0.060775899	0.8914411
0.302567266	0.287232782	0.584854032	0.060775899	0.8914411
0.302988421	0.343485525	1	0.489952655	0.8914411
0.303001368	0.169067106	0.034012327	0.000324671	0.8914411
0.303001368	0.169067106	0.034012327	0.000324671	0.8914411
0.30303855	0.159302764	0.000261772	0.011906673	0.8914411
0.30303855	0.159302764	0.000261772	0.011906673	0.8914411
0.303094717	0.346683554	1	0.645935818	0.8914411
0.303470701	0.280429229	0.584854032	0.042826979	0.8914411
0.304037515	-0.198935115	0.087427713	0.001791344	0.8914411
0.304196164	-0.277314548	0.043105813	0.489952655	0.8914411
0.304333965	-0.241280271	0.307400178	0.009270366	0.8914411
0.304681567	-0.326696058	1	0.242167012	0.8914411
0.304709009	-0.326617232	1	0.242167012	0.8914411
0.304713777	-0.090214457	0.000261772	8.55E-07	0.8914411
0.305009086	0.098410698	4.58E-06	0.000324671	0.8914411
0.305064753	-0.237482105	0.295435331	0.009270366	0.8914411
0.305075326	0.323844875	0.307400178	1	0.8914411
0.30513768	0.325514786	1	0.242167012	0.8914411
0.305270321	-0.346902269	0.737584497	1	0.8914411
0.305270321	-0.346902269	0.737584497	1	0.8914411
0.305322877	-0.054760156	5.98E-06	4.93E-08	0.8914411
0.305322877	-0.054760156	5.98E-06	4.93E-08	0.8914411
0.305350006	0.086872337	1.23E-06	0.000124759	0.8914411
0.305350006	0.086872337	1.23E-06	0.000124759	0.8914411
0.305415097	0.344862611	1	0.601859652	0.8914411
0.30552695	0.20177949	0.095006685	0.002093839	0.8914411
0.305724002	0.293235453	0.095538718	0.704162183	0.8914411
0.305769626	0.347541948	0.881481882	1	0.8914411
0.305801865	0.265969059	0.439859057	0.025431136	0.8914411
0.305828884	0.301099853	0.737584497	0.085309726	0.8914411
0.305928863	-0.20886246	0.104319013	0.002420613	0.8914411
0.306299843	-0.342116698	0.584854032	1	0.8914411
0.306321574	0.346858637	1	0.850173656	0.8914411
0.306321574	0.346858637	1	0.850173656	0.8914411
0.306341023	0.274365236	0.439859057	0.031639242	0.8914411
0.306341023	0.274365236	0.439859057	0.031639242	0.8914411
0.306681416	0.2575059	0.412603306	0.02341474	0.8914411
0.306842898	0.292904224	0.691111318	0.066020955	0.8914411
0.306842898	0.292904224	0.691111318	0.066020955	0.8914411
0.306842898	0.292904224	0.691111318	0.066020955	0.8914411
0.307203346	0.151688108	0.000249829	0.009270366	0.8914411
0.307203346	0.151688108	0.000249829	0.009270366	0.8914411
0.307310105	0.344293957	1	0.704162183	0.8914411
0.307461864	0.332759199	0.412603306	1	0.8914411

0.307461864	0.332759199	0.412603306	1	0.8914411
0.307461864	0.332759199	0.412603306	1	0.8914411
0.307461864	0.332759199	0.412603306	1	0.8914411
0.307595323	0.286963657	0.584854032	0.060775899	0.8914411
0.307595323	0.286963657	0.584854032	0.060775899	0.8914411
0.308082005	0.253146091	0.030989574	0.242167012	0.8914411
0.308117993	-0.296217226	0.737584497	0.085309726	0.8914411
0.308133351	0.097997989	4.58E-06	0.000324671	0.8914411
0.308347657	-0.248328125	0.412603306	0.011906673	0.8914411
0.308520895	-0.335392117	1	0.416730107	0.8914411
0.308852385	0.324047778	0.34036444	1	0.8914411
0.308859437	-0.320682882	0.307400178	1	0.8914411
0.308859437	-0.320682882	0.307400178	1	0.8914411
0.309011348	0.209412439	0.104319013	0.002420613	0.8914411
0.309100067	-0.298702018	0.737584497	0.085309726	0.8914411
0.309100067	-0.298702018	0.737584497	0.085309726	0.8914411
0.309117091	-0.058261219	1.22E-05	5.53E-08	0.8914411
0.309117091	-0.058261219	1.22E-05	5.53E-08	0.8914411
0.309135415	0.28155748	0.075374611	0.489952655	0.8914411
0.309530802	0.066859156	1.48E-07	1.65E-05	0.8914411
0.309530802	0.066859156	1.48E-07	1.65E-05	0.8914411
0.309703628	0.344830179	0.881481882	1	0.8914411
0.310080038	0.317086788	0.295435331	1	0.8914411
0.31028157	-0.335411384	1	0.416730107	0.8914411
0.310357785	-0.341148915	1	0.601859652	0.8914411
0.310620352	0.251613164	0.030989574	0.242167012	0.8914411
0.310620352	0.251613164	0.030989574	0.242167012	0.8914411
0.310620352	0.251613164	0.030989574	0.242167012	0.8914411
0.310677349	0.158233629	0.025193624	0.00028972	0.8914411
0.310812494	0.049340629	2.61E-08	3.06E-06	0.8914411
0.310812494	0.049340629	2.61E-08	3.06E-06	0.8914411
0.311293775	-0.326114183	1	0.242167012	0.8914411
0.311401392	-0.333418854	1	0.416730107	0.8914411
0.311596278	-0.27529341	0.493333254	0.045751002	0.8914411
0.311600331	-0.343275821	0.737584497	1	0.8914411
0.311675393	0.12470581	6.77E-05	0.002420613	0.8914411
0.311675393	0.12470581	6.77E-05	0.002420613	0.8914411
0.311675393	0.12470581	6.77E-05	0.002420613	0.8914411
0.311675393	0.12470581	6.77E-05	0.002420613	0.8914411
0.311920071	-0.136117574	0.00974133	4.69E-05	0.8914411
0.311945005	-0.337999885	1	0.489952655	0.8914411
0.312041118	-0.343129731	0.881481882	1	0.8914411
0.312090309	0.126457096	6.77E-05	0.002420613	0.8914411
0.312358996	0.330208088	0.439859057	1	0.8914411
0.312373299	-0.173779016	0.035148121	0.000596242	0.8914411
0.312373299	-0.173779016	0.035148121	0.000596242	0.8914411
0.312639266	-0.258477428	0.412603306	0.025431136	0.8914411
0.312639266	-0.258477428	0.412603306	0.025431136	0.8914411
0.312731555	0.334231907	1	0.489952655	0.8914411
0.31292158	0.326928501	0.412603306	1	0.8914411
0.31292158	0.326928501	0.412603306	1	0.8914411
0.313176884	0.333549217	1	0.416730107	0.8914411
0.313192598	0.336490487	1	0.489952655	0.8914411
0.314038045	-0.341203903	1	0.704162183	0.8914411
0.314207702	-0.295419602	0.134257478	0.704162183	0.8914411
0.314220559	0.069691153	3.18E-07	4.35E-05	0.8914411
0.314220559	0.069691153	3.18E-07	4.35E-05	0.8914411
0.314432045	0.21670373	0.169239932	0.004171959	0.8914411
0.314778054	-0.305317832	0.989933981	0.145037597	0.8914411
0.315308087	-0.208716701	0.004526275	0.085309726	0.8914411
0.315378934	0.295036902	0.134257478	0.704162183	0.8914411
0.315387073	0.302718466	0.88322726	0.129712192	0.8914411
0.315387073	0.302718466	0.88322726	0.129712192	0.8914411
0.31567258	-0.324934427	1	0.364167203	0.8914411

0.31567258	-0.324934427	1	0.364167203	0.8914411
0.315722826	-0.339935481	1	0.704162183	0.8914411
0.315722826	-0.339935481	1	0.704162183	0.8914411
0.315785011	-0.113971509	0.001207877	1.04E-05	0.8914411
0.315785011	-0.113971509	0.001207877	1.04E-05	0.8914411
0.316007485	-0.295918065	0.169239932	0.704162183	0.8914411
0.316007485	-0.295918065	0.169239932	0.704162183	0.8914411
0.316053888	0.337901857	1	0.645935818	0.8914411
0.316053888	0.337901857	1	0.645935818	0.8914411
0.316130816	0.088023645	3.58E-06	0.000140627	0.8914411
0.316421756	0.316684709	0.307400178	1	0.8914411
0.316421756	0.316684709	0.307400178	1	0.8914411
0.316421756	0.316684709	0.307400178	1	0.8914411
0.316491696	0.324216211	0.412603306	1	0.8914411
0.316610938	0.331942742	0.439859057	1	0.8914411
0.316610938	0.331942742	0.439859057	1	0.8914411
0.316819622	0.297717964	0.881481882	0.126792252	0.8914411
0.316835151	-0.168576091	0.0008771	0.025431136	0.8914411
0.316906354	0.339196149	0.737584497	1	0.8914411
0.316922837	-0.13224401	0.008580492	4.69E-05	0.8914411
0.317024907	0.081054973	1.23E-06	0.000115129	0.8914411
0.317230142	0.27543469	0.584854032	0.053084396	0.8914411
0.317234226	-0.284538246	0.095006685	0.645935818	0.940898014
0.317234226	-0.284538246	0.095006685	0.645935818	0.940898014
0.317234226	-0.284538246	0.095006685	0.645935818	0.940898014
0.317234226	-0.284538246	0.095006685	0.645935818	0.940898014
0.317758941	-0.323071493	0.412603306	1	0.940898014
0.317758941	-0.323071493	0.412603306	1	0.940898014
0.317905476	0.240322177	0.025193624	0.242167012	0.940898014
0.317905476	0.240322177	0.025193624	0.242167012	0.940898014
0.318833309	0.298979659	0.169239932	1	0.940898014
0.319325418	0.338170305	1	1	0.940898014
0.319325418	0.338170305	1	1	0.940898014
0.31935715	-0.055886752	1.22E-05	5.53E-08	0.940898014
0.319512966	-0.323599431	0.412603306	1	0.940898014
0.319686583	-0.335511604	0.584854032	1	0.940898014
0.319686583	-0.335511604	0.584854032	1	0.940898014
0.319893362	0.334276086	1	0.601859652	0.940898014
0.319893362	0.334276086	1	0.601859652	0.940898014
0.319954392	-0.327816952	0.439859057	1	0.940898014
0.319954392	-0.327816952	0.439859057	1	0.940898014
0.319954392	-0.327816952	0.439859057	1	0.940898014
0.319954392	-0.327816952	0.439859057	1	0.940898014
0.320176856	0.193284041	0.075374611	0.001791344	0.940898014
0.320176856	0.193284041	0.075374611	0.001791344	0.940898014
0.320176856	0.193284041	0.075374611	0.001791344	0.940898014
0.320234418	-0.320288059	1	0.305102445	0.946960882
0.320852871	0.275509442	0.584854032	0.060775899	0.946960882
0.320930359	0.149068486	0.000261772	0.009270366	0.946960882
0.321929398	0.33648511	0.989933981	1	0.946960882
0.322233326	0.306050842	1	0.145037597	0.946960882
0.322740721	0.262754664	0.043105813	0.416730107	0.946960882
0.322810155	-0.090070671	0.000467492	3.06E-06	0.946960882
0.322931273	-0.318273872	1	0.272770942	0.946960882
0.32300063	0.2235804	0.205025676	0.006372161	0.946960882
0.32300063	0.2235804	0.205025676	0.006372161	0.946960882
0.323129487	0.199443604	0.004273917	0.085309726	0.946960882
0.323129487	0.199443604	0.004273917	0.085309726	0.946960882
0.323518332	0.22735004	0.205025676	0.009270366	0.946960882
0.323518332	0.22735004	0.205025676	0.009270366	0.946960882
0.324040522	0.329874344	1	0.489952655	0.946960882
0.324040522	0.329874344	1	0.489952655	0.946960882
0.324469357	-0.314428653	0.412603306	1	0.946960882
0.324817248	0.152421329	0.000295301	0.011906673	0.946960882

0.325106522	0.089084754	4.58E-06	0.00028972	0.946960882
0.325746853	-0.283277508	0.691111318	0.085309726	0.946960882
0.325746853	-0.283277508	0.691111318	0.085309726	0.946960882
0.325807107	-0.333747582	0.881481882	1	0.946960882
0.325878545	0.321607655	1	0.397942841	0.946960882
0.325878545	0.321607655	1	0.397942841	0.946960882
0.325950932	0.329260975	0.584854032	1	0.946960882
0.326082256	0.32464285	1	0.489952655	0.946960882
0.326685156	0.035548516	6.38E-09	2.98E-07	0.946960882
0.326685156	0.035548516	6.38E-09	2.98E-07	0.946960882
0.326695857	0.333254879	0.989933981	1	0.946960882
0.326726389	0.32697729	1	0.489952655	0.946960882
0.326726389	0.32697729	1	0.489952655	0.946960882
0.32678957	-0.158830144	0.030989574	0.0002997	0.946960882
0.32678957	-0.158830144	0.030989574	0.0002997	0.946960882
0.32710361	0.183357168	0.001207877	0.052574033	0.946960882
0.327126618	-0.324702305	0.439859057	1	0.946960882
0.327175962	-0.330326471	1	0.704162183	0.946960882
0.327175962	-0.330326471	1	0.704162183	0.946960882
0.327357775	0.15516635	0.000467492	0.011906673	0.946960882
0.327634932	0.261570808	0.043105813	0.416730107	0.946960882
0.327650732	-0.094975508	0.0008771	3.66E-06	0.946960882
0.327650732	-0.094975508	0.0008771	3.66E-06	0.946960882
0.327719681	-0.277217285	0.584854032	0.061934943	0.946960882
0.328063254	-0.331649088	1	0.704162183	0.946960882
0.328131913	0.09478584	5.98E-06	0.000324671	0.946960882
0.328131913	0.09478584	5.98E-06	0.000324671	0.946960882
0.328131913	0.09478584	5.98E-06	0.000324671	0.946960882
0.32878622	-0.130241074	0.008580492	4.69E-05	0.946960882
0.328830605	0.132215908	8.53E-05	0.004171959	0.946960882
0.328830605	0.132215908	8.53E-05	0.004171959	0.946960882
0.328830605	0.132215908	8.53E-05	0.004171959	0.946960882
0.328830605	0.132215908	8.53E-05	0.004171959	0.946960882
0.328857485	-0.120015303	0.004273917	4.35E-05	0.946960882
0.329230899	0.275193724	0.087427713	0.601859652	0.946960882
0.329230899	0.275193724	0.087427713	0.601859652	0.946960882
0.329286335	-0.116828047	0.003169634	1.80E-05	0.946960882
0.329286335	-0.116828047	0.003169634	1.80E-05	0.946960882
0.329286335	-0.116828047	0.003169634	1.80E-05	0.946960882
0.330229354	0.307644289	1	0.242167012	0.946960882
0.330233268	0.179074658	0.043105813	0.001090387	0.946960882
0.330351925	-0.146769938	0.018394332	0.000124759	0.946960882
0.330351925	-0.146769938	0.018394332	0.000124759	0.946960882
0.330351925	-0.146769938	0.018394332	0.000124759	0.946960882
0.330351925	-0.146769938	0.018394332	0.000124759	0.946960882
0.330483493	0.312603431	0.412603306	1	0.946960882
0.330483493	0.312603431	0.412603306	1	0.946960882
0.330483493	0.312603431	0.412603306	1	0.946960882
0.330483493	0.312603431	0.412603306	1	0.946960882
0.330483493	0.312603431	0.412603306	1	0.946960882
0.330483493	0.312603431	0.412603306	1	0.946960882
0.330682989	0.325878788	1	0.489952655	0.946960882
0.330843572	-0.144509582	0.018163994	0.000124759	0.946960882
0.330843572	-0.144509582	0.018163994	0.000124759	0.946960882
0.33100205	0.257066833	0.439859057	0.031639242	0.946960882
0.331119972	0.32666939	0.584854032	1	0.946960882
0.331119972	0.32666939	0.584854032	1	0.946960882
0.33193196	0.28612122	0.169239932	0.704162183	0.946960882
0.33193196	0.28612122	0.169239932	0.704162183	0.946960882
0.332227651	-0.291075856	0.881481882	0.126792252	0.946960882
0.332276409	0.32656561	1	0.645935818	0.946960882
0.332276409	0.32656561	1	0.645935818	0.946960882
0.332687211	0.106967489	1.56E-05	0.001090387	0.946960882
0.332772903	0.293404617	0.989933981	0.145037597	0.946960882

0.332777325	0.298655209	1	0.145037597	0.946960882
0.332777325	0.298655209	1	0.145037597	0.946960882
0.332777325	0.298655209	1	0.145037597	0.946960882
0.332851431	0.081441538	1.84E-06	0.000115129	0.946960882
0.3328595	0.327045364	0.691111318	1	0.946960882
0.33286151	0.224846894	0.018163994	0.164911511	0.946960882
0.332887663	0.327233255	1	0.704162183	0.950058752
0.333244341	0.262073277	0.439859057	0.045751002	0.950058752
0.333244341	0.262073277	0.439859057	0.045751002	0.950058752
0.333438631	-0.113175939	0.002297238	1.65E-05	0.950058752
0.333660835	0.185756892	0.003169634	0.060775899	0.950058752
0.334151837	-0.322998646	1	0.489952655	0.950058752
0.334151837	-0.322998646	1	0.489952655	0.950058752
0.334151837	-0.322998646	1	0.489952655	0.950058752
0.334207705	0.258541972	0.439859057	0.041232736	0.950058752
0.334361703	0.087373895	4.58E-06	0.000163543	0.950058752
0.334423113	0.164756022	0.035148121	0.000596242	0.950058752
0.334532957	-0.327738635	0.881481882	1	0.950058752
0.335054913	-0.234961807	0.34036444	0.017403168	0.950058752
0.335054913	-0.234961807	0.34036444	0.017403168	0.950058752
0.335187968	-0.327358085	1	0.850173656	0.950058752
0.335187968	-0.327358085	1	0.850173656	0.950058752
0.335247646	0.252995853	0.043105813	0.416730107	0.950058752
0.335456557	-0.271702608	0.584854032	0.061934943	0.950058752
0.335537751	-0.326331558	1	0.704162183	0.950058752
0.335877456	0.322898737	0.584854032	1	0.950058752
0.335924967	-0.252440594	0.439859057	0.031639242	0.950058752
0.335924967	-0.252440594	0.439859057	0.031639242	0.950058752
0.336061322	0.324214877	1	0.704162183	0.953968118
0.336426014	0.160265218	0.0008771	0.02341474	0.953968118
0.337036331	-0.32561267	0.737584497	1	0.953968118
0.337708391	0.055432743	9.36E-08	5.99E-06	0.953968118
0.338117492	-0.324926444	0.737584497	1	0.953968118
0.338117492	-0.324926444	0.737584497	1	0.953968118
0.33814132	0.137678096	0.000245628	0.006372161	0.953968118
0.338493141	-0.271199543	0.584854032	0.066020955	0.953968118
0.338982606	0.319946556	1	0.489952655	0.953968118
0.338982606	0.319946556	1	0.489952655	0.953968118
0.339552279	0.281080651	0.737584497	0.126792252	0.953968118
0.339571017	0.159382407	0.034012327	0.000348916	0.953968118
0.340119261	-0.323966783	0.881481882	1	0.953968118
0.340408795	-0.323009119	0.737584497	1	0.953968118
0.340712437	-0.27323164	0.095538718	0.704162183	0.953968118
0.340884435	0.251358007	0.439859057	0.031639242	0.953968118
0.340884435	0.251358007	0.439859057	0.031639242	0.953968118
0.341052749	-0.316630961	1	0.489952655	0.953968118
0.341235427	-0.20437715	0.169239932	0.005827837	0.953968118
0.341238361	0.321229348	0.691111318	1	0.953968118
0.341707607	0.305334812	1	0.242167012	0.953968118
0.34174159	-0.307473847	1	0.364167203	0.953968118
0.34174159	-0.307473847	1	0.364167203	0.953968118
0.342062911	0.286999449	0.19926681	1	0.953968118
0.342403236	-0.176774835	0.043105813	0.001791344	0.953968118
0.342403236	-0.176774835	0.043105813	0.001791344	0.953968118
0.342403236	-0.176774835	0.043105813	0.001791344	0.953968118
0.342403236	-0.176774835	0.043105813	0.001791344	0.953968118
0.342527424	0.196744208	0.104319013	0.003736177	0.953968118
0.34303406	-0.322254665	1	1	0.953968118
0.343098242	-0.300388849	1	0.242167012	0.953968118
0.343634637	-0.12700292	0.008580492	6.41E-05	0.953968118
0.343634637	-0.12700292	0.008580492	6.41E-05	0.953968118
0.34428273	0.312677591	0.439859057	1	0.953968118
0.344311891	0.258976412	0.439859057	0.053084396	0.953968118
0.344528956	-0.171395563	0.043105813	0.001090387	0.953968118

0.344731378	0.270367908	0.584854032	0.085309726	0.953968118
0.34506302	-0.227412621	0.307400178	0.011906673	0.953968118
0.346136437	0.219352169	0.208605117	0.009270366	0.953968118
0.346524953	0.312845566	1	0.489952655	0.953968118
0.346524953	0.312845566	1	0.489952655	0.953968118
0.346689848	-0.287336957	0.989933981	0.145037597	0.953968118
0.346754378	0.309119783	0.439859057	1	0.953968118
0.346812208	-0.225061308	0.025193624	0.242167012	0.953968118
0.346878694	0.191678469	0.095538718	0.002420613	0.953968118
0.346895633	-0.313454633	1	0.489952655	0.953968118
0.346948312	0.317778981	0.737584497	1	0.953968118
0.346963046	0.208183416	0.192696374	0.006372161	0.953968118
0.347079845	0.03887099	7.89E-09	5.53E-07	0.953968118
0.347129349	-0.311550059	1	0.489952655	0.953968118
0.347264098	-0.163518437	0.035148121	0.000997588	0.953968118
0.347270624	0.315676266	1	0.601859652	0.953968118
0.347270624	0.315676266	1	0.601859652	0.953968118
0.347270624	0.315676266	1	0.601859652	0.953968118
0.347772626	-0.107214053	0.001207877	1.65E-05	0.953968118
0.347772626	-0.107214053	0.001207877	1.65E-05	0.953968118
0.347772626	-0.107214053	0.001207877	1.65E-05	0.953968118
0.347848535	0.265658662	0.584854032	0.066020955	0.953968118
0.347940853	0.188091833	0.004273917	0.061934943	0.953968118
0.348082761	-0.318437973	0.737584497	1	0.953968118
0.348088932	0.262718234	0.584854032	0.060775899	0.953968118
0.348475056	0.16548438	0.001207877	0.029310175	0.953968118
0.348475056	0.16548438	0.001207877	0.029310175	0.953968118
0.34862051	-0.214022029	0.205025676	0.009270366	0.953968118
0.34862051	-0.214022029	0.205025676	0.009270366	0.953968118
0.348837661	0.209829462	0.19926681	0.006372161	0.953968118
0.348837661	0.209829462	0.19926681	0.006372161	0.953968118
0.348837661	0.209829462	0.19926681	0.006372161	0.953968118
0.348948821	-0.315152412	1	0.645935818	0.953968118
0.349247482	-0.31685293	1	0.704162183	0.953968118
0.349332744	-0.243720776	0.439859057	0.031639242	0.953968118
0.349641113	0.278455439	0.737584497	0.126792252	0.953968118
0.350026152	-0.210770507	0.19926681	0.006372161	0.953968118
0.350380339	0.313005275	1	0.601859652	0.953968118
0.350836731	-0.274359151	0.737584497	0.126792252	0.953968118
0.350847448	-0.296778194	1	0.242167012	0.953968118
0.350943253	0.295164274	0.34036444	1	0.953968118
0.350943253	0.295164274	0.34036444	1	0.953968118
0.350968259	0.276608859	0.169239932	0.704162183	0.953968118
0.350968259	0.276608859	0.169239932	0.704162183	0.953968118
0.351406472	0.110382028	6.19E-05	0.001791344	0.953968118
0.351413594	0.284320003	0.989933981	0.145037597	0.953968118
0.35201121	-0.301782918	1	0.364167203	0.953968118
0.35201121	-0.301782918	1	0.364167203	0.953968118
0.352077482	-0.311558103	0.584854032	1	0.953968118
0.352077482	-0.311558103	0.584854032	1	0.953968118
0.352176069	0.058552788	1.10E-07	9.47E-06	0.953968118
0.352365002	0.305279335	0.439859057	1	0.953968118
0.352365002	0.305279335	0.439859057	1	0.953968118
0.352365002	0.305279335	0.439859057	1	0.953968118
0.352365002	0.305279335	0.439859057	1	0.953968118
0.352459192	0.312150424	0.584854032	1	0.953968118
0.352459192	0.312150424	0.584854032	1	0.953968118
0.352532677	0.17708733	0.002810027	0.052574033	0.953968118
0.35258432	-0.240845769	0.412603306	0.029310175	0.953968118
0.352711228	0.165814833	0.043105813	0.000997588	0.953968118
0.353026827	0.107311427	6.19E-05	0.001791344	0.953968118
0.353062641	-0.168140125	0.043105813	0.001090387	0.953968118
0.353062641	-0.168140125	0.043105813	0.001090387	0.953968118
0.353114077	0.310024409	0.584854032	1	0.953968118

0.353559125	0.304880407	0.439859057	1	0.953968118
0.353559125	0.304880407	0.439859057	1	0.953968118
0.3541334	0.15556134	0.0008771	0.02341474	0.953968118
0.3541334	0.15556134	0.0008771	0.02341474	0.953968118
0.354364412	0.29056776	0.307400178	1	0.953968118
0.354364412	0.29056776	0.307400178	1	0.953968118
0.354388137	-0.087455725	0.000467492	3.16E-06	0.953968118
0.354388137	-0.087455725	0.000467492	3.16E-06	0.953968118
0.354583214	0.247600017	0.043105813	0.416730107	0.953968118
0.354588071	0.213269597	0.205025676	0.009270366	0.953968118
0.354723989	0.30101416	0.439859057	1	0.953968118
0.355068263	0.298388012	1	0.364167203	0.953968118
0.355198539	-0.113914881	0.004273917	4.69E-05	0.953968118
0.355198539	-0.113914881	0.004273917	4.69E-05	0.953968118
0.355198539	-0.113914881	0.004273917	4.69E-05	0.953968118
0.355198539	-0.113914881	0.004273917	4.69E-05	0.953968118
0.355214768	-0.312282467	1	0.704162183	0.953968118
0.355214768	-0.312282467	1	0.704162183	0.953968118
0.355214768	-0.312282467	1	0.704162183	0.953968118
0.355377202	-0.311713771	1	0.704162183	0.953968118
0.355416178	0.261421905	0.584854032	0.066020955	0.953968118
0.355760556	0.313418437	0.737584497	1	0.953968118
0.356265535	0.234215567	0.412603306	0.025431136	0.953968118
0.356543875	0.151506965	0.030989574	0.000324671	0.953968118
0.356543875	0.151506965	0.030989574	0.000324671	0.953968118
0.356543875	0.151506965	0.030989574	0.000324671	0.953968118
0.356543875	0.151506965	0.030989574	0.000324671	0.953968118
0.356600328	0.17328856	0.002297238	0.045751002	0.953968118
0.35669101	0.240013098	0.439859057	0.031639242	0.953968118
0.35669101	0.240013098	0.439859057	0.031639242	0.953968118
0.356708001	-0.132342186	0.010835964	0.000115129	0.953968118
0.356863627	-0.193623585	0.104319013	0.003736177	0.953968118
0.356863627	-0.193623585	0.104319013	0.003736177	0.953968118
0.357564654	-0.299448095	1	0.397942841	0.953968118
0.357888103	0.057329199	1.10E-07	9.47E-06	0.953968118
0.357888103	0.057329199	1.10E-07	9.47E-06	0.953968118
0.357888103	0.057329199	1.10E-07	9.47E-06	0.953968118
0.3579249	-0.295424528	1	0.305102445	0.953968118
0.3579249	-0.295424528	1	0.305102445	0.953968118
0.35809033	0.230248065	0.412603306	0.025431136	0.953968118
0.358269978	-0.106610759	0.001207877	1.65E-05	0.953968118
0.358321147	-0.047339496	4.58E-06	4.93E-08	0.953968118
0.358321147	-0.047339496	4.58E-06	4.93E-08	0.953968118
0.358352863	0.308240363	1	0.601859652	0.953968118
0.358429056	-0.310710803	1	0.704162183	0.953968118
0.358429056	-0.310710803	1	0.704162183	0.953968118
0.358838955	-0.311953718	1	1	0.953968118
0.359429673	0.170930939	0.001611045	0.042826979	0.953968118
0.359755283	0.210407417	0.205025676	0.009270366	0.953968118
0.359892921	0.112581407	0.00373662	4.69E-05	0.953968118
0.359892921	0.112581407	0.00373662	4.69E-05	0.953968118
0.36006664	-0.053709173	1.22E-05	9.27E-08	0.953968118
0.36006664	-0.053709173	1.22E-05	9.27E-08	0.953968118
0.360073016	0.24216094	0.439859057	0.040084197	0.953968118
0.360623867	-0.273733749	0.192696374	0.850173656	0.953968118
0.360623867	-0.273733749	0.192696374	0.850173656	0.953968118
0.361466329	-0.303368755	1	0.489952655	0.953968118
0.36170075	0.287454878	0.307400178	1	0.953968118
0.36170075	0.287454878	0.307400178	1	0.953968118
0.361915897	0.310057452	1	1	0.953968118
0.361942131	0.24528142	0.439859057	0.052574033	0.953968118
0.361957491	0.192378274	0.008580492	0.085309726	0.953968118
0.362103096	-0.079150789	0.000249829	3.06E-06	0.953968118
0.362103096	-0.079150789	0.000249829	3.06E-06	0.953968118

0.362103096	-0.079150789	0.000249829	3.06E-06	0.953968118
0.362360568	-0.135064371	0.016023223	0.000124759	0.953968118
0.362632665	0.296422775	0.439859057	1	0.953968118
0.362632665	0.296422775	0.439859057	1	0.953968118
0.362898941	0.12012841	0.008580492	6.41E-05	0.953968118
0.363011661	0.30769485	1	0.704162183	0.953968118
0.363049645	-0.308285777	0.737584497	1	0.953968118
0.363130007	0.036973793	7.89E-09	2.98E-07	0.953968118
0.363130007	0.036973793	7.89E-09	2.98E-07	0.953968118
0.363393558	0.306237339	0.584854032	1	0.953968118
0.363393558	0.306237339	0.584854032	1	0.953968118
0.363428551	0.203398324	0.19926681	0.006372161	0.953968118
0.363428551	0.203398324	0.19926681	0.006372161	0.953968118
0.363531241	-0.20575893	0.19926681	0.009270366	0.953968118
0.363607601	-0.053011445	1.22E-05	9.27E-08	0.953968118
0.363607601	-0.053011445	1.22E-05	9.27E-08	0.953968118
0.363607601	-0.053011445	1.22E-05	9.27E-08	0.953968118
0.36365438	0.137696735	0.018163994	0.00028972	0.953968118
0.363863716	0.091138491	1.22E-05	0.000324671	0.953968118
0.363863716	0.091138491	1.22E-05	0.000324671	0.953968118
0.36455541	0.270149104	0.737584497	0.126792252	0.953968118
0.364704226	0.25614735	0.095538718	0.489952655	0.953968118
0.364704226	0.25614735	0.095538718	0.489952655	0.953968118
0.3647153	0.163942664	0.043105813	0.001090387	0.953968118
0.364832129	-0.304889526	0.584854032	1	0.953968118
0.364889531	-0.308260123	1	1	0.953968118
0.364895085	0.291325094	1	0.364167203	0.953968118
0.364973408	-0.055545336	1.56E-05	9.79E-08	0.953968118
0.364973408	-0.055545336	1.56E-05	9.79E-08	0.953968118
0.364973408	-0.055545336	1.56E-05	9.79E-08	0.953968118
0.365174461	-0.052801482	1.22E-05	9.27E-08	0.953968118
0.365294549	0.072735267	1.23E-06	7.48E-05	0.953968118
0.365294549	0.072735267	1.23E-06	7.48E-05	0.953968118
0.365294549	0.072735267	1.23E-06	7.48E-05	0.953968118
0.36533801	-0.101760442	0.001207877	1.04E-05	0.953968118
0.36533801	-0.101760442	0.001207877	1.04E-05	0.953968118
0.36533801	-0.101760442	0.001207877	1.04E-05	0.953968118
0.366104612	-0.154169976	0.034012327	0.000596242	0.953968118
0.366104612	-0.154169976	0.034012327	0.000596242	0.953968118
0.366115166	0.294454579	1	0.397942841	0.953968118
0.366171193	0.254672054	0.095538718	0.489952655	0.953968118
0.366171193	0.254672054	0.095538718	0.489952655	0.953968118
0.366216617	0.230786035	0.035148121	0.305102445	0.953968118
0.366433798	-0.252061593	0.584854032	0.060775899	0.953968118
0.366463684	-0.301242422	0.584854032	1	0.953968118
0.366566411	0.230959107	0.412603306	0.029310175	0.953968118
0.366751485	0.299327351	0.439859057	1	0.953968118
0.366751485	0.299327351	0.439859057	1	0.953968118
0.366891968	-0.240891156	0.043105813	0.416730107	0.953968118
0.367128446	0.296849762	0.439859057	1	0.953968118
0.367178727	-0.306684465	0.989933981	1	0.953968118
0.367379212	0.304114962	1	0.704162183	0.953968118
0.367379212	0.304114962	1	0.704162183	0.953968118
0.367723812	-0.300335128	1	0.489952655	0.953968118
0.367741787	0.243512525	0.439859057	0.052574033	0.953968118
0.368163605	-0.304790774	1	0.704162183	0.953968118
0.368163605	-0.304790774	1	0.704162183	0.953968118
0.368597422	0.304449137	0.737584497	1	0.953968118
0.368597422	0.304449137	0.737584497	1	0.953968118
0.368621971	0.250903109	0.087427713	0.489952655	0.953968118
0.368621971	0.250903109	0.087427713	0.489952655	0.953968118
0.368690406	0.202497731	0.19926681	0.009270366	0.953968118
0.368755134	0.240383887	0.043105813	0.416730107	1
0.368808428	-0.292041992	1	0.397942841	1

0.368861628	0.25547834	0.584854032	0.085309726	1
0.369155625	0.303590279	1	0.704162183	1
0.369155625	0.303590279	1	0.704162183	1
0.369233675	-0.305568978	1	1	1
0.369644054	0.119048977	8.53E-05	0.003736177	1
0.369644054	0.119048977	8.53E-05	0.003736177	1
0.369808498	-0.256261852	0.584854032	0.085309726	1
0.36996169	0.253546491	0.095538718	0.489952655	1
0.370231126	-0.288601415	0.412603306	1	1
0.370231126	-0.288601415	0.412603306	1	1
0.370433109	0.271975419	0.881481882	0.145037597	1
0.370433109	0.271975419	0.881481882	0.145037597	1
0.370433109	0.271975419	0.881481882	0.145037597	1
0.370446681	0.28946429	0.412603306	1	1
0.370488004	0.275386729	0.989933981	0.164911511	1
0.37060726	-0.060765837	6.19E-05	2.98E-07	1
0.370742925	-0.295005768	1	0.489952655	1
0.371269552	0.29840408	1	0.489952655	1
0.371269552	0.29840408	1	0.489952655	1
0.371801356	0.119854419	0.000155445	0.003736177	1
0.371872083	-0.270267155	0.881481882	0.145037597	1
0.372219998	-0.301589573	1	0.704162183	1
0.372247898	-0.293988379	0.439859057	1	1
0.372247898	-0.293988379	0.439859057	1	1
0.372450386	0.227569829	0.412603306	0.025431136	1
0.37292271	0.239170982	0.439859057	0.052574033	1
0.373058716	-0.121353555	0.008580492	0.000115129	1
0.373119731	0.290805474	1	0.416730107	1
0.373249129	0.302835473	1	1	1
0.373254884	0.258588156	0.134257478	0.704162183	1
0.373771211	0.250317268	0.584854032	0.066020955	1
0.373771211	0.250317268	0.584854032	0.066020955	1
0.374168902	0.301614889	1	0.850173656	1
0.374743872	-0.298983592	0.584854032	1	1
0.374743872	-0.298983592	0.584854032	1	1
0.374806923	0.020910534	3.99E-10	6.53E-09	1
0.375257717	0.300752598	0.737584497	1	1
0.375570549	-0.116011109	0.004526275	4.69E-05	1
0.375570549	-0.116011109	0.004526275	4.69E-05	1
0.375570549	-0.116011109	0.004526275	4.69E-05	1
0.375570549	-0.116011109	0.004526275	4.69E-05	1
0.375570549	-0.116011109	0.004526275	4.69E-05	1
0.375570549	-0.116011109	0.004526275	4.69E-05	1
0.375634102	0.299866603	0.737584497	1	1
0.375758976	0.301012696	1	0.850173656	1
0.375939267	0.266748264	0.783270054	0.145037597	1
0.376012606	-0.27875253	0.307400178	1	1
0.376033365	0.158588598	0.001207877	0.029310175	1
0.376033365	0.158588598	0.001207877	0.029310175	1
0.376062589	0.239623963	0.073031096	0.416730107	1
0.376157128	0.225601016	0.412603306	0.025431136	1
0.376157128	0.225601016	0.412603306	0.025431136	1
0.376500596	-0.263439112	0.737584497	0.129712192	1
0.376503734	0.289876032	0.439859057	1	1
0.376503734	0.289876032	0.439859057	1	1
0.376758685	0.299942939	0.737584497	1	1
0.37682564	-0.163058066	0.043105813	0.001791344	1
0.377463007	0.299216364	0.737584497	1	1
0.377941369	-0.25922499	0.169239932	0.704162183	1
0.377941369	-0.25922499	0.169239932	0.704162183	1
0.377976438	0.179374887	0.007135475	0.066020955	1
0.378233458	0.176695933	0.004526275	0.060775899	1
0.378744233	0.156051647	0.001207877	0.025431136	1
0.379490428	-0.12298713	0.009746439	0.000115129	1

0.379490428	-0.12298713	0.009746439	0.000115129	1
0.379494348	0.060797458	4.10E-07	1.65E-05	1
0.379494348	0.060797458	4.10E-07	1.65E-05	1
0.379494348	0.060797458	4.10E-07	1.65E-05	1
0.379769098	-0.266433263	0.205025676	0.850173656	1
0.379769098	-0.266433263	0.205025676	0.850173656	1
0.379769098	-0.266433263	0.205025676	0.850173656	1
0.379769098	-0.266433263	0.205025676	0.850173656	1
0.379769098	-0.266433263	0.205025676	0.850173656	1
0.379769098	-0.266433263	0.205025676	0.850173656	1
0.380351332	0.298644142	1	1	1
0.380351332	0.298644142	1	1	1
0.380658087	-0.22820628	0.412603306	0.031639242	1
0.380927586	-0.183795796	0.095538718	0.004171959	1
0.381204796	-0.133692015	0.018163994	0.00028972	1
0.381204796	-0.133692015	0.018163994	0.00028972	1
0.381232767	0.138796382	0.0008771	0.011906673	1
0.381280819	-0.296279861	1	0.704162183	1
0.381379471	-0.292074157	1	0.489952655	1
0.381603576	-0.137002724	0.018394332	0.0002997	1
0.381603576	-0.137002724	0.018394332	0.0002997	1
0.381626944	0.193681999	0.016023223	0.126792252	1
0.382163829	0.159241411	0.043105813	0.001695222	1
0.382163829	0.159241411	0.043105813	0.001695222	1
0.382164053	-0.296994501	1	0.850173656	1
0.382403441	0.277075375	1	0.242167012	1
0.382484902	-0.278913627	0.412603306	1	1
0.382484902	-0.278913627	0.412603306	1	1
0.382484902	-0.278913627	0.412603306	1	1
0.382484902	-0.278913627	0.412603306	1	1
0.382751026	-0.23228845	0.043105813	0.397942841	1
0.382814081	0.257769951	0.169239932	0.704162183	1
0.382814081	0.257769951	0.169239932	0.704162183	1
0.382833274	0.043237226	4.36E-08	3.06E-06	1
0.383135594	-0.242597291	0.584854032	0.060775899	1
0.383208795	-0.213506046	0.307400178	0.02341474	1
0.383226708	0.056776639	3.18E-07	1.04E-05	1
0.383226708	0.056776639	3.18E-07	1.04E-05	1
0.383351217	-0.041239393	1.23E-06	1.63E-08	1
0.383464878	-0.227811833	0.412603306	0.031639242	1
0.383705043	0.253514666	0.584854032	0.102782106	1
0.383797331	0.296448665	1	1	1
0.383797331	0.296448665	1	1	1
0.384568924	-0.209481909	0.295435331	0.02341474	1
0.384638141	0.293421139	0.691111318	1	1
0.385032784	0.23108808	0.043105813	0.397942841	1
0.385032784	0.23108808	0.043105813	0.397942841	1
0.385075028	0.255020242	0.169239932	0.704162183	1
0.385175334	-0.107222513	0.003169634	4.69E-05	1
0.385582862	-0.147583109	0.034012327	0.000596242	1
0.385981778	0.253557942	0.169239932	0.704162183	1
0.385981778	0.253557942	0.169239932	0.704162183	1
0.386069558	0.184767128	0.009746439	0.085309726	1
0.386070849	0.264881483	0.881481882	0.145037597	1
0.386110189	0.290866076	1	0.645935818	1
0.386377161	0.071223829	1.84E-06	7.48E-05	1
0.386913563	0.054521331	1.97E-07	9.47E-06	1
0.386913563	0.054521331	1.97E-07	9.47E-06	1
0.387076923	-0.287951917	0.584854032	1	1
0.38722304	-0.294338646	1	1	1
0.387858192	-0.245292504	0.584854032	0.085309726	1
0.387999059	0.216876173	0.035148121	0.242167012	1
0.388222881	-0.180182503	0.095538718	0.004171959	1
0.388375625	0.096950875	6.19E-05	0.000997588	1

0.388375625	0.096950875	6.19E-05	0.000997588	1
0.388375625	0.096950875	6.19E-05	0.000997588	1
0.388458055	0.288442725	0.584854032	1	1
0.388458055	0.288442725	0.584854032	1	1
0.388762256	0.249406766	0.584854032	0.10273905	1
0.388832299	-0.223450338	0.412603306	0.031639242	1
0.389626465	0.08099203	5.98E-06	0.00028972	1
0.389664474	0.291866296	0.737584497	1	1
0.389941457	0.045538172	9.36E-08	3.06E-06	1
0.390274664	-0.25226629	0.691111318	0.126792252	1
0.390340371	0.262102331	0.881481882	0.145037597	1
0.390585107	0.22464045	0.412603306	0.031639242	1
0.391124089	0.285345451	0.584854032	1	1
0.391124089	0.285345451	0.584854032	1	1
0.391153143	0.281679847	0.439859057	1	1
0.391732188	-0.284935944	0.584854032	1	1
0.392042	-0.127736109	0.016023223	0.000163543	1
0.392042	-0.127736109	0.016023223	0.000163543	1
0.392042	-0.127736109	0.016023223	0.000163543	1
0.392042	-0.127736109	0.016023223	0.000163543	1
0.392302591	-0.064401341	6.77E-05	8.55E-07	1
0.392450891	0.245421349	0.104319013	0.601859652	1
0.392450891	0.245421349	0.104319013	0.601859652	1
0.392474321	-0.24373854	0.099208829	0.489952655	1
0.392474321	-0.24373854	0.099208829	0.489952655	1
0.39301299	0.220908311	0.412603306	0.031639242	1
0.39301299	0.220908311	0.412603306	0.031639242	1
0.393674064	-0.253629304	0.737584497	0.145037597	1
0.393674064	-0.253629304	0.737584497	0.145037597	1
0.393830081	-0.290132951	1	1	1
0.394688272	-0.281317227	1	0.489952655	1
0.394758462	0.276094204	1	0.397942841	1
0.394759535	-0.201796939	0.241457119	0.011906673	1
0.394759535	-0.201796939	0.241457119	0.011906673	1
0.394872432	-0.287630739	1	0.704162183	1
0.394872432	-0.287630739	1	0.704162183	1
0.394872432	-0.287630739	1	0.704162183	1
0.395129983	0.275158981	1	0.397942841	1
0.395129983	0.275158981	1	0.397942841	1
0.395547835	-0.170546823	0.075374611	0.003736177	1
0.395547835	-0.170546823	0.075374611	0.003736177	1
0.395572967	0.260564826	0.88322726	0.164911511	1
0.396299044	0.216923951	0.412603306	0.029310175	1
0.396326051	0.288651125	1	1	1
0.396527878	0.144971151	0.0008771	0.02341474	1
0.396527878	0.144971151	0.0008771	0.02341474	1
0.396594838	0.26054894	0.295435331	1	1
0.396766904	-0.219589322	0.412603306	0.031639242	1
0.397072979	0.235996421	0.439859057	0.061934943	1
0.397072979	0.235996421	0.439859057	0.061934943	1
0.397130663	-0.281095226	0.584854032	1	1
0.397312775	0.167891552	0.075374611	0.002420613	1
0.39746555	0.141412077	0.030989574	0.000596242	1
0.39747559	-0.283530953	0.584854032	1	1
0.39747559	-0.283530953	0.584854032	1	1
0.398107235	0.233219448	0.087427713	0.489952655	1
0.398107235	0.233219448	0.087427713	0.489952655	1
0.398107235	0.233219448	0.087427713	0.489952655	1
0.398126848	0.170300868	0.087427713	0.003736177	1
0.398285413	0.214181113	0.03951775	0.242167012	1
0.398387717	0.234128099	0.439859057	0.061934943	1
0.398548103	-0.095265217	0.001207877	1.65E-05	1
0.399235667	0.160432951	0.003169634	0.042826979	1
0.400164433	0.196695118	0.025193624	0.145037597	1

0.400299399	-0.245623791	0.584854032	0.126792252	1
0.400326354	0.28325882	1	0.704162183	1
0.400326354	0.28325882	1	0.704162183	1
0.40034961	-0.27802019	0.439859057	1	1
0.40034961	-0.27802019	0.439859057	1	1
0.40034961	-0.27802019	0.439859057	1	1
0.40034961	-0.27802019	0.439859057	1	1
0.400514375	-0.170215039	0.087427713	0.003736177	1
0.400514375	-0.170215039	0.087427713	0.003736177	1
0.400752788	0.261782734	1	0.242167012	1
0.400760605	0.249560585	0.192696374	0.704162183	1
0.401633446	0.138128562	0.0008771	0.017403168	1
0.401633446	0.138128562	0.0008771	0.017403168	1
0.401705596	0.235648287	0.584854032	0.085309726	1
0.401705596	0.235648287	0.584854032	0.085309726	1
0.401705596	0.235648287	0.584854032	0.085309726	1
0.402470164	0.277991506	1	0.489952655	1
0.402496473	0.225816098	0.439859057	0.053084396	1
0.402963655	-0.229877916	0.439859057	0.060775899	1
0.403039964	0.124422435	0.000295301	0.006372161	1
0.403230004	0.066946417	1.23E-06	4.69E-05	1
0.403523375	-0.252651389	0.205025676	0.850173656	1
0.403523375	-0.252651389	0.205025676	0.850173656	1
0.404074638	0.283762593	0.88322726	1	1
0.404074638	0.283762593	0.88322726	1	1
0.404245823	-0.179358428	0.120559922	0.006372161	1
0.404539018	0.274452591	0.439859057	1	1
0.404564933	0.282425528	0.737584497	1	1
0.404888899	0.248166772	0.737584497	0.145037597	1
0.404888899	0.248166772	0.737584497	0.145037597	1
0.405075926	-0.174025444	0.095538718	0.005827837	1
0.405075926	-0.174025444	0.095538718	0.005827837	1
0.405230067	0.179019053	0.010835964	0.085309726	1
0.405230067	0.179019053	0.010835964	0.085309726	1
0.405247558	-0.261238725	1	0.242167012	1
0.405247558	-0.261238725	1	0.242167012	1
0.405247558	-0.261238725	1	0.242167012	1
0.405305471	0.055109452	3.18E-07	1.04E-05	1
0.405391123	0.283383447	1	1	1
0.405517316	-0.172573097	0.008580492	0.066020955	1
0.405577248	-0.142220542	0.034012327	0.000997588	1
0.405577248	-0.142220542	0.034012327	0.000997588	1
0.405616015	-0.277975525	1	0.601859652	1
0.405888714	-0.282856813	1	1	1
0.405888714	-0.282856813	1	1	1
0.405888714	-0.282856813	1	1	1
0.406277929	0.150516237	0.001207877	0.029310175	1
0.406277929	0.150516237	0.001207877	0.029310175	1
0.406277929	0.150516237	0.001207877	0.029310175	1
0.406465809	-0.086108672	0.0008771	9.47E-06	1
0.406466366	-0.282453026	1	1	1
0.406486789	0.226927992	0.439859057	0.060775899	1
0.406486789	0.226927992	0.439859057	0.060775899	1
0.406486789	0.226927992	0.439859057	0.060775899	1
0.406561049	0.228102402	0.439859057	0.060775899	1
0.406561049	0.228102402	0.439859057	0.060775899	1
0.406588633	-0.25875349	1	0.242167012	1
0.40704082	-0.240079891	0.584854032	0.102782106	1
0.407155544	-0.238935607	0.584854032	0.10273905	1
0.407508231	0.218685173	0.043105813	0.397942841	1
0.407508231	0.218685173	0.043105813	0.397942841	1
0.407508231	0.218685173	0.043105813	0.397942841	1
0.407508231	0.218685173	0.043105813	0.397942841	1
0.407556251	-0.251669532	0.881481882	0.145037597	1

0.407788813	-0.208850194	0.035148121	0.242167012	1
0.408284101	-0.081252667	0.0008771	5.99E-06	1
0.408770275	0.23766574	0.134257478	0.601859652	1
0.408905844	0.281250196	1	1	1
0.409012395	0.13363785	0.0008771	0.011906673	1
0.409763064	-0.201604947	0.307400178	0.02341474	1
0.409926545	-0.22559533	0.075374611	0.416730107	1
0.410010172	0.200047213	0.295435331	0.02341474	1
0.410010172	0.200047213	0.295435331	0.02341474	1
0.411038421	-0.276080807	1	0.645935818	1
0.411038421	-0.276080807	1	0.645935818	1
0.411057071	-0.067925926	0.000155445	8.55E-07	1
0.411057071	-0.067925926	0.000155445	8.55E-07	1
0.411400329	0.027685873	2.10E-09	9.79E-08	1
0.411482497	0.088368254	4.14E-05	0.000596242	1
0.411673087	0.190079021	0.024201521	0.145037597	1
0.411706521	-0.036715932	1.09E-06	1.63E-08	1
0.411921582	0.258440448	1	0.242167012	1
0.41205504	0.232458062	0.099208829	0.489952655	1
0.41205504	0.232458062	0.099208829	0.489952655	1
0.41205504	0.232458062	0.099208829	0.489952655	1
0.412229747	0.27453618	0.584854032	1	1
0.412429673	0.225185834	0.087427713	0.489952655	1
0.412429673	0.225185834	0.087427713	0.489952655	1
0.412706751	0.269050434	1	0.489952655	1
0.412713157	-0.278037864	0.881481882	1	1
0.412847546	0.172943138	0.009746439	0.085309726	1
0.412847546	0.172943138	0.009746439	0.085309726	1
0.41292789	-0.271732065	1	0.489952655	1
0.41292789	-0.271732065	1	0.489952655	1
0.412968142	-0.144869232	0.035148121	0.001090387	1
0.412968142	-0.144869232	0.035148121	0.001090387	1
0.413142016	-0.152249913	0.043105813	0.001791344	1
0.413379674	-0.270940984	1	0.489952655	1
0.413745786	0.267543694	1	0.416730107	1
0.413745786	0.267543694	1	0.416730107	1
0.413745786	0.267543694	1	0.416730107	1
0.41404584	0.054844656	4.10E-07	1.04E-05	1
0.414053386	0.2232733	0.439859057	0.060775899	1
0.414053386	0.2232733	0.439859057	0.060775899	1
0.414059744	-0.029766072	1.10E-07	6.53E-09	1
0.414059744	-0.029766072	1.10E-07	6.53E-09	1
0.414173139	0.203050304	0.035148121	0.242167012	1
0.415164446	0.264259587	1	0.397942841	1
0.415219892	0.133524996	0.0008771	0.011906673	1
0.415249607	0.21278127	0.043105813	0.364167203	1
0.415398307	0.269566142	1	0.489952655	1
0.415692378	0.262090826	1	0.364167203	1
0.415993394	0.277191781	1	1	1
0.416039458	-0.277003077	1	1	1
0.41604458	-0.249314321	0.295435331	0.850173656	1
0.41604458	-0.249314321	0.295435331	0.850173656	1
0.41604458	-0.249314321	0.295435331	0.850173656	1
0.41609146	0.256282557	1	0.242167012	1
0.416096067	0.266409751	0.439859057	1	1
0.416096067	0.266409751	0.439859057	1	1
0.416185161	-0.1628545	0.075374611	0.003736177	1
0.41631033	-0.276591667	0.989933981	1	1
0.416544779	0.104230001	8.53E-05	0.002420613	1
0.416589327	-0.272161517	0.584854032	1	1
0.416589327	-0.272161517	0.584854032	1	1
0.417110723	0.051924652	3.18E-07	9.47E-06	1
0.417110723	0.051924652	3.18E-07	9.47E-06	1
0.417110723	0.051924652	3.18E-07	9.47E-06	1

0.417321341	0.247815845	0.881481882	0.164911511	1
0.417416353	0.244821486	0.737584497	0.145037597	1
0.417416353	0.244821486	0.737584497	0.145037597	1
0.417416353	0.244821486	0.737584497	0.145037597	1
0.417563495	0.110507891	0.000228282	0.003736177	1
0.417783359	0.239406178	0.691111318	0.129712192	1
0.418282798	0.188704933	0.205025676	0.011906673	1
0.418305973	0.21054972	0.412603306	0.034534533	1
0.418703365	0.052224992	3.18E-07	9.47E-06	1
0.418752309	-0.274360238	0.737584497	1	1
0.418894888	-0.241667988	0.737584497	0.145037597	1
0.418894888	-0.241667988	0.737584497	0.145037597	1
0.419180027	0.247806846	0.295435331	0.850173656	1
0.419200261	-0.273174282	1	0.704162183	1
0.419226996	-0.099298854	0.002810027	4.69E-05	1
0.419226996	-0.099298854	0.002810027	4.69E-05	1
0.419226996	-0.099298854	0.002810027	4.69E-05	1
0.419451628	-0.179392152	0.169239932	0.009270366	1
0.419922571	0.222574708	0.095006685	0.489952655	1
0.420069031	0.160082789	0.004526275	0.053084396	1
0.420177025	0.235688046	0.584854032	0.126792252	1
0.420597874	0.274469093	1	1	1
0.420597874	0.274469093	1	1	1
0.420597874	0.274469093	1	1	1
0.420675829	-0.265059964	0.439859057	1	1
0.420675829	-0.265059964	0.439859057	1	1
0.4211838	0.239126132	0.737584497	0.145037597	1
0.421301209	0.272993616	1	0.850173656	1
0.421301209	0.272993616	1	0.850173656	1
0.421427505	0.243602816	0.205025676	0.850173656	1
0.421659282	0.26517555	0.439859057	1	1
0.421776092	0.200974815	0.307400178	0.025431136	1
0.421868018	-0.186864723	0.025193624	0.145037597	1
0.421913332	0.256851175	1	0.364167203	1
0.42302435	-0.210664274	0.412603306	0.042826979	1
0.423262429	-0.266998002	0.584854032	1	1
0.423262429	-0.266998002	0.584854032	1	1
0.423298209	-0.27243993	1	1	1
0.423298209	-0.27243993	1	1	1
0.423368553	0.254412778	1	0.242167012	1
0.423745247	0.141230322	0.001207877	0.025431136	1
0.424367338	0.271822563	1	1	1
0.424367338	0.271822563	1	1	1
0.424367338	0.271822563	1	1	1
0.424367338	0.271822563	1	1	1
0.424367338	0.271822563	1	1	1
0.424367338	0.271822563	1	1	1
0.424511143	0.165619035	0.095006685	0.004171959	1
0.424638956	-0.26303092	0.439859057	1	1
0.425019292	0.234349662	0.584854032	0.126792252	1
0.425406093	0.200067975	0.035148121	0.242167012	1
0.425406093	0.200067975	0.035148121	0.242167012	1
0.425764342	-0.111484187	0.008580492	0.000124759	1
0.425764342	-0.111484187	0.008580492	0.000124759	1
0.426212993	-0.165781597	0.095538718	0.005827837	1
0.426213153	0.254526898	1	0.364167203	1
0.426324411	0.194818954	0.034012327	0.242167012	1
0.426477336	-0.171387972	0.104319013	0.006372161	1
0.426477336	-0.171387972	0.104319013	0.006372161	1
0.426477336	-0.171387972	0.104319013	0.006372161	1
0.426552976	-0.238954081	0.737584497	0.145037597	1
0.426552976	-0.238954081	0.737584497	0.145037597	1
0.427101209	-0.269494616	1	0.850173656	1
0.427101209	-0.269494616	1	0.850173656	1

0.42733026	0.069337041	4.58E-06	0.000115129	1
0.427485562	-0.158405212	0.075374611	0.003736177	1
0.427840193	0.229122095	0.584854032	0.126792252	1
0.428164326	-0.270090399	1	1	1
0.428164326	-0.270090399	1	1	1
0.428208392	0.1345862	0.0008771	0.02341474	1
0.428208392	0.1345862	0.0008771	0.02341474	1
0.428208392	0.1345862	0.0008771	0.02341474	1
0.428756416	0.141824245	0.001207877	0.025431136	1
0.428759834	-0.266797478	0.737584497	1	1
0.428780626	-0.239125558	0.737584497	0.145037597	1
0.428798465	-0.238042697	0.205025676	0.704162183	1
0.428798465	-0.238042697	0.205025676	0.704162183	1
0.428798465	-0.238042697	0.205025676	0.704162183	1
0.428798465	-0.238042697	0.205025676	0.704162183	1
0.429090214	0.037702549	2.78E-08	8.55E-07	1
0.429090214	0.037702549	2.78E-08	8.55E-07	1
0.429438654	-0.135555616	0.034012327	0.000997588	1
0.429438654	-0.135555616	0.034012327	0.000997588	1
0.429471135	-0.099320752	0.003169634	4.69E-05	1
0.429674181	0.25558007	1	0.397942841	1
0.429674181	0.25558007	1	0.397942841	1
0.429675554	-0.082522553	0.0008771	9.47E-06	1
0.429839195	0.235416597	0.19926681	0.704162183	1
0.429842598	0.146763905	0.003169634	0.031639242	1
0.429917555	-0.231517899	0.584854032	0.126792252	1
0.429917555	-0.231517899	0.584854032	0.126792252	1
0.430209695	0.262078929	0.584854032	1	1
0.430327275	-0.268813988	1	1	1
0.430377757	-0.063230701	8.53E-05	8.55E-07	1
0.430460029	-0.228594256	0.584854032	0.126792252	1
0.43114848	0.262145879	1	0.489952655	1
0.431218556	0.17769669	0.169239932	0.009270366	1
0.431742758	0.233143746	0.691111318	0.145037597	1
0.432091422	-0.074982422	0.000322414	4.11E-06	1
0.432091422	-0.074982422	0.000322414	4.11E-06	1
0.432277133	0.211458643	0.075374611	0.397942841	1
0.432501087	-0.15142196	0.043105813	0.002420613	1
0.432716726	0.149779621	0.004273917	0.041232736	1
0.432738078	-0.233180789	0.691111318	0.145037597	1
0.433184429	0.210308605	0.439859057	0.053084396	1
0.433184429	0.210308605	0.439859057	0.053084396	1
0.433184429	0.210308605	0.439859057	0.053084396	1
0.433694417	-0.116823039	0.010835964	0.0002997	1
0.434037983	0.264733553	0.737584497	1	1
0.43454152	-0.256886084	0.439859057	1	1
0.43454152	-0.256886084	0.439859057	1	1
0.434801906	0.263976732	1	0.704162183	1
0.434801906	0.263976732	1	0.704162183	1
0.434922652	-0.253300895	1	0.416730107	1
0.4350992	-0.178127991	0.192696374	0.011906673	1
0.43544731	-0.265534877	1	1	1
0.435459591	0.197095091	0.043105813	0.242167012	1
0.435591788	0.125990936	0.024201521	0.000596242	1
0.435591788	0.125990936	0.024201521	0.000596242	1
0.435797753	0.187884475	0.034012327	0.164911511	1
0.435989268	-0.252867881	1	0.416730107	1
0.435989268	-0.252867881	1	0.416730107	1
0.436435691	0.263036029	1	0.704162183	1
0.436873998	0.264440999	1	1	1
0.43694547	0.025969404	2.10E-09	9.27E-08	1
0.437254788	0.263986859	1	0.850173656	1
0.4375687	0.231962865	0.737584497	0.145037597	1
0.4375687	0.231962865	0.737584497	0.145037597	1

0.4375687	0.231962865	0.737584497	0.145037597	1
0.437708432	0.166881719	0.104319013	0.006372161	1
0.437836097	0.061638557	1.84E-06	4.69E-05	1
0.437995797	-0.086497962	0.0008771	1.65E-05	1
0.437995797	-0.086497962	0.0008771	1.65E-05	1
0.438325264	-0.26426186	1	1	1
0.438444643	0.260804445	0.691111318	1	1
0.438718496	0.044557346	9.36E-08	3.66E-06	1
0.438932371	0.189110288	0.034012327	0.164911511	1
0.439035095	-0.223724478	0.584854032	0.126792252	1
0.439050747	-0.260471702	0.691111318	1	1
0.439302791	-0.26217241	1	0.850173656	1
0.439422945	0.063967806	3.58E-06	6.41E-05	1
0.439422945	0.063967806	3.58E-06	6.41E-05	1
0.439433754	0.198770042	0.412603306	0.031639242	1
0.440145533	0.240347629	0.989933981	0.242167012	1
0.440845271	0.262841383	1	1	1
0.4409034	-0.240522121	0.989933981	0.242167012	1
0.4409034	-0.240522121	0.989933981	0.242167012	1
0.441006862	-0.257449797	1	0.601859652	1
0.441010448	0.216177234	0.439859057	0.085309726	1
0.441014024	0.080822917	1.56E-05	0.000348916	1
0.441014024	0.080822917	1.56E-05	0.000348916	1
0.441014024	0.080822917	1.56E-05	0.000348916	1
0.441014024	0.080822917	1.56E-05	0.000348916	1
0.441014024	0.080822917	1.56E-05	0.000348916	1
0.441014024	0.080822917	1.56E-05	0.000348916	1
0.441177161	0.05979455	1.23E-06	4.69E-05	1
0.441191797	-0.259841877	1	0.704162183	1
0.441191797	-0.259841877	1	0.704162183	1
0.441351407	0.260411216	1	0.704162183	1
0.441415359	0.187936864	0.034012327	0.164911511	1
0.441701338	-0.250550465	1	0.416730107	1
0.442001514	-0.171197371	0.169239932	0.009270366	1
0.442001514	-0.171197371	0.169239932	0.009270366	1
0.442046857	0.136965448	0.034012327	0.001695222	1
0.442046857	0.136965448	0.034012327	0.001695222	1
0.442071701	0.250468836	1	0.416730107	1
0.442228119	-0.251926724	1	0.489952655	1
0.442239685	-0.243393985	1	0.242167012	1
0.44237762	0.260189105	1	0.704162183	1
0.442682791	0.259925373	0.737584497	1	1
0.442795762	0.193789457	0.307400178	0.031639242	1
0.442795762	0.193789457	0.307400178	0.031639242	1
0.44298295	-0.253227756	1	0.489952655	1
0.443054891	-0.24761976	1	0.397942841	1
0.443337339	-0.259854428	1	0.850173656	1
0.443357898	0.196237358	0.412603306	0.031639242	1
0.443542689	0.063821264	4.58E-06	6.41E-05	1
0.444213348	0.256157567	0.584854032	1	1
0.444444939	0.238634894	0.989933981	0.242167012	1
0.444444939	0.238634894	0.989933981	0.242167012	1
0.444444939	0.238634894	0.989933981	0.242167012	1
0.444752783	0.144559898	0.004273917	0.031639242	1
0.444819953	0.241253826	1	0.242167012	1
0.444819953	0.241253826	1	0.242167012	1
0.444861041	-0.246966448	1	0.397942841	1
0.444861041	-0.246966448	1	0.397942841	1
0.445030846	0.210574349	0.095006685	0.416730107	1
0.44506365	-0.235020324	0.307400178	0.850173656	1
0.445665748	0.214665473	0.099208829	0.489952655	1
0.445763389	-0.236487989	0.88322726	0.242167012	1
0.447259918	0.147507494	0.043105813	0.002420613	1
0.447259918	0.147507494	0.043105813	0.002420613	1

0.447284065	-0.115198433	0.016023223	0.0002997	1
0.447382879	0.169764166	0.169239932	0.009270366	1
0.447474717	0.250984647	1	0.489952655	1
0.447474717	0.250984647	1	0.489952655	1
0.447552692	-0.153737451	0.075374611	0.003736177	1
0.447552692	-0.153737451	0.075374611	0.003736177	1
0.447646462	0.240874841	0.412603306	1	1
0.447982509	0.055500566	1.09E-06	1.93E-05	1
0.448028133	-0.170557367	0.169239932	0.009270366	1
0.448293237	0.254832035	0.584854032	1	1
0.448293237	0.254832035	0.584854032	1	1
0.448362086	0.036399111	4.36E-08	8.55E-07	1
0.449014331	-0.254482974	1	0.704162183	1
0.449014331	-0.254482974	1	0.704162183	1
0.449380481	-0.217068823	0.584854032	0.102782106	1
0.449380481	-0.217068823	0.584854032	0.102782106	1
0.449380481	-0.217068823	0.584854032	0.102782106	1
0.44965151	0.243531889	1	0.397942841	1
0.449762839	0.253499913	1	0.704162183	1
0.449905676	-0.214334347	0.120559922	0.489952655	1
0.449958464	0.209763932	0.095538718	0.489952655	1
0.450539327	-0.161630756	0.095538718	0.006372161	1
0.450717371	0.167571248	0.169239932	0.009270366	1
0.450717371	0.167571248	0.169239932	0.009270366	1
0.451044489	-0.161326327	0.095538718	0.006372161	1
0.451542568	0.240140224	0.412603306	1	1
0.45183431	0.215567206	0.584854032	0.102782106	1
0.45183431	0.215567206	0.584854032	0.102782106	1
0.452282626	0.237649002	1	0.242167012	1
0.452282626	0.237649002	1	0.242167012	1
0.452317766	0.224570172	0.737584497	0.145037597	1
0.452394436	-0.256303522	1	1	1
0.452705438	0.169813885	0.169239932	0.011906673	1
0.452990837	0.202541578	0.439859057	0.060775899	1
0.453536086	0.128684948	0.001207877	0.02341474	1
0.453891702	0.232551367	0.307400178	1	1
0.454261441	0.250516821	1	0.645935818	1
0.454286683	0.127303956	0.030989574	0.000997588	1
0.454322652	-0.208183921	0.439859057	0.085309726	1
0.454457579	-0.252440226	1	0.704162183	1
0.454490151	0.148097157	0.073031096	0.003736177	1
0.454494545	0.095597775	8.53E-05	0.002093839	1
0.454494545	0.095597775	8.53E-05	0.002093839	1
0.454606996	0.245876899	0.439859057	1	1
0.454911231	-0.144720275	0.043105813	0.002420613	1
0.454962002	0.217648462	0.584854032	0.126792252	1
0.45554813	0.179033066	0.205025676	0.02341474	1
0.45554813	0.179033066	0.205025676	0.02341474	1
0.45554813	0.179033066	0.205025676	0.02341474	1
0.45554813	0.179033066	0.205025676	0.02341474	1
0.45554813	0.179033066	0.205025676	0.02341474	1
0.45554813	0.179033066	0.205025676	0.02341474	1
0.455636204	-0.172502704	0.19926681	0.011906673	1
0.455705455	-0.182038666	0.295435331	0.025431136	1
0.455955343	0.222722428	0.691111318	0.145037597	1
0.455955343	0.222722428	0.691111318	0.145037597	1
0.456100921	-0.254232127	1	1	1
0.456100921	-0.254232127	1	1	1
0.45612176	0.254179744	1	1	1
0.456479108	0.201326185	0.075374611	0.397942841	1
0.456780593	0.18834589	0.307400178	0.031639242	1
0.456886252	-0.114277114	0.016023223	0.000324671	1
0.457106495	0.145598998	0.066393258	0.003668854	1
0.457321471	-0.253013937	1	1	1

0.457321471	-0.253013937	1	1	1
0.457501929	0.246044652	1	0.489952655	1
0.457689499	-0.249992592	1	0.704162183	1
0.4578634	-0.250964127	1	0.704162183	1
0.457865424	0.170989594	0.025193624	0.129712192	1
0.458552992	0.109930648	0.000467492	0.006372161	1
0.458552992	0.109930648	0.000467492	0.006372161	1
0.45898186	0.217369589	0.192696374	0.645935818	1
0.459195883	0.208721946	0.439859057	0.085309726	1
0.459442905	0.165201293	0.169239932	0.009270366	1
0.459599517	0.137145678	0.003169634	0.029310175	1
0.459599517	0.137145678	0.003169634	0.029310175	1
0.459599517	0.137145678	0.003169634	0.029310175	1
0.459599517	0.137145678	0.003169634	0.029310175	1
0.459599517	0.137145678	0.003169634	0.029310175	1
0.459599517	0.137145678	0.003169634	0.029310175	1
0.459660046	0.251151855	0.881481882	1	1
0.459660046	0.251151855	0.881481882	1	1
0.459816359	-0.027611406	1.10E-07	6.53E-09	1
0.459816359	-0.027611406	1.10E-07	6.53E-09	1
0.46001506	-0.228107966	0.307400178	0.850173656	1
0.46001506	-0.228107966	0.307400178	0.850173656	1
0.460335354	0.251888375	1	1	1
0.460335354	0.251888375	1	1	1
0.460882713	0.219398452	0.205025676	0.704162183	1
0.460882713	0.219398452	0.205025676	0.704162183	1
0.461235801	-0.066499488	0.000249829	3.66E-06	1
0.461366174	0.203652417	0.439859057	0.06824239	1
0.461457055	-0.203838262	0.439859057	0.06824239	1
0.461928633	0.200541265	0.087427713	0.416730107	1
0.462059463	-0.240966216	1	0.489952655	1
0.462142584	0.171105986	0.19926681	0.017403168	1
0.462297613	-0.250310955	1	1	1
0.462403889	-0.191388298	0.412603306	0.042826979	1
0.462419547	0.216863899	0.19926681	0.645935818	1
0.462866712	0.220851174	0.737584497	0.145037597	1
0.463081369	0.177719794	0.034012327	0.164911511	1
0.463124238	-0.246041892	0.584854032	1	1
0.463124238	-0.246041892	0.584854032	1	1
0.46345831	-0.222985984	0.737584497	0.164911511	1
0.46360361	-0.248416251	1	0.850173656	1
0.463644514	0.241662395	1	0.489952655	1
0.463696207	-0.211991725	0.584854032	0.126792252	1
0.463773766	-0.249927098	1	1	1
0.464512719	-0.160272645	0.120559922	0.009270366	1
0.464770261	-0.24942401	1	1	1
0.464770261	-0.24942401	1	1	1
0.464851246	0.153505348	0.010835964	0.061934943	1
0.464851246	0.153505348	0.010835964	0.061934943	1
0.465123036	0.128705538	0.001207877	0.02341474	1
0.465123036	0.128705538	0.001207877	0.02341474	1
0.46520768	-0.233526373	0.412603306	1	1
0.46520768	-0.233526373	0.412603306	1	1
0.465285374	0.211716838	0.584854032	0.126792252	1
0.465676985	-0.107185472	0.000467492	0.005827837	1
0.465899438	0.208381474	0.584854032	0.102782106	1
0.465998662	0.229319749	1	0.242167012	1
0.465998662	0.229319749	1	0.242167012	1
0.465998662	0.229319749	1	0.242167012	1
0.466043002	0.239978177	0.439859057	1	1
0.466209213	-0.246768017	1	0.704162183	1
0.466452534	0.248088065	1	1	1
0.466673284	0.225095688	0.307400178	0.850173656	1
0.466807187	-0.216469492	0.584854032	0.145037597	1

0.466989544	0.148821487	0.075374611	0.005827837	1
0.467573838	0.122783322	0.001207877	0.017403168	1
0.467683382	0.247816342	1	1	1
0.467913206	0.139022778	0.004526275	0.031639242	1
0.467951681	0.243172109	1	0.645935818	1
0.467973309	0.23369964	0.439859057	1	1
0.468261937	-0.239877877	1	0.489952655	1
0.468266012	0.24265958	1	0.645935818	1
0.4688553	0.197226288	0.439859057	0.060775899	1
0.4688553	0.197226288	0.439859057	0.060775899	1
0.4688553	0.197226288	0.439859057	0.060775899	1
0.469209204	0.167533273	0.026049691	0.129712192	1
0.469416764	0.134531574	0.043105813	0.002093839	1
0.469507602	0.245307678	1	0.850173656	1
0.469710068	0.233421786	1	0.397942841	1
0.469710068	0.233421786	1	0.397942841	1
0.470178651	-0.24635388	1	1	1
0.470178651	-0.24635388	1	1	1
0.470412472	0.126944605	0.001207877	0.02341474	1
0.4704229	0.240377897	1	0.601859652	1
0.470496827	-0.245132936	0.881481882	1	1
0.470528767	-0.183788276	0.043105813	0.242167012	1
0.470528767	-0.183788276	0.043105813	0.242167012	1
0.470649968	0.234482741	1	0.416730107	1
0.470649968	0.234482741	1	0.416730107	1
0.470748203	0.097522575	0.004526275	0.000115129	1
0.470748203	0.097522575	0.004526275	0.000115129	1
0.470748203	0.097522575	0.004526275	0.000115129	1
0.470748203	0.097522575	0.004526275	0.000115129	1
0.471551018	0.212393395	0.19926681	0.645935818	1
0.471551018	0.212393395	0.19926681	0.645935818	1
0.471665609	-0.17070918	0.205025676	0.02341474	1
0.471665609	-0.17070918	0.205025676	0.02341474	1
0.471812445	-0.225261665	0.989933981	0.242167012	1
0.47190201	-0.244999735	0.989933981	1	1
0.471935818	0.229192108	1	0.364167203	1
0.472465711	-0.233347687	1	0.416730107	1
0.473156068	-0.135792252	0.004273917	0.031639242	1
0.473328997	-0.234596967	1	0.489952655	1
0.473328997	-0.234596967	1	0.489952655	1
0.474942184	0.04039601	9.36E-08	3.06E-06	1
0.474942184	0.04039601	9.36E-08	3.06E-06	1
0.474942184	0.04039601	9.36E-08	3.06E-06	1
0.474942184	0.04039601	9.36E-08	3.06E-06	1
0.474942184	0.04039601	9.36E-08	3.06E-06	1
0.474942184	0.04039601	9.36E-08	3.06E-06	1
0.474983321	0.230470359	1	0.397942841	1
0.475258984	0.167599739	0.030989574	0.145037597	1
0.475377726	-0.150309467	0.095006685	0.006372161	1
0.475491676	-0.176191992	0.035148121	0.164911511	1
0.475521313	0.200129277	0.439859057	0.085309726	1
0.475521313	0.200129277	0.439859057	0.085309726	1
0.475835068	-0.088661566	0.002297238	4.69E-05	1
0.476020357	-0.137938805	0.043105813	0.002420613	1
0.476020357	-0.137938805	0.043105813	0.002420613	1
0.476020357	-0.137938805	0.043105813	0.002420613	1
0.476068103	-0.23603871	1	0.489952655	1
0.47641006	0.13979665	0.043105813	0.003736177	1
0.476472832	-0.176593502	0.307400178	0.029310175	1
0.476959871	0.164243776	0.026049691	0.126792252	1
0.477082481	-0.13068065	0.035148121	0.002093839	1
0.477082481	-0.13068065	0.035148121	0.002093839	1
0.477244839	-0.242055135	0.989933981	1	1
0.477324107	0.163598112	0.026049691	0.126792252	1

0.477324107	0.163598112	0.026049691	0.126792252	1
0.477334653	-0.216432637	0.737584497	0.217058541	1
0.477615583	-0.13528049	0.043105813	0.002420613	1
0.477615583	-0.13528049	0.043105813	0.002420613	1
0.477830916	0.238628553	1	0.704162183	1
0.478070177	0.22116927	0.88322726	0.242167012	1
0.47821736	-0.119737967	0.026049691	0.000997588	1
0.478360523	-0.205948994	0.584854032	0.126792252	1
0.478883579	-0.216845371	0.307400178	0.704162183	1
0.478883579	-0.216845371	0.307400178	0.704162183	1
0.478883579	-0.216845371	0.307400178	0.704162183	1
0.478883579	-0.216845371	0.307400178	0.704162183	1
0.478934138	0.204314917	0.169239932	0.489952655	1
0.479571615	0.241313834	1	1	1
0.479621452	-0.143724117	0.075374611	0.004171959	1
0.479676717	-0.237546086	1	0.704162183	1
0.479743188	0.048841728	1.09E-06	1.04E-05	1
0.479795997	0.241041635	1	1	1
0.479795997	0.241041635	1	1	1
0.480078415	-0.167208632	0.205025676	0.02341474	1
0.480078415	-0.167208632	0.205025676	0.02341474	1
0.48008018	0.200680136	0.439859057	0.102782106	1
0.480382833	-0.236400429	0.584854032	1	1
0.480653997	0.152332218	0.095538718	0.009270366	1
0.480772709	-0.235970505	0.584854032	1	1
0.481372913	-0.236142515	0.584854032	1	1
0.481556334	-0.090850681	0.003169634	0.000108916	1
0.481698193	0.218391503	0.881481882	0.242167012	1
0.481778368	0.23099313	1	0.489952655	1
0.481780456	0.184607354	0.412603306	0.052574033	1
0.481780456	0.184607354	0.412603306	0.052574033	1
0.481780456	0.184607354	0.412603306	0.052574033	1
0.482348495	0.226097157	0.439859057	1	1
0.482500437	-0.238762338	1	1	1
0.483368419	-0.077697272	0.0008771	1.80E-05	1
0.483677749	0.223929832	1	0.364167203	1
0.483787907	-0.039612232	4.58E-06	9.79E-08	1
0.483825897	0.238983209	1	1	1
0.483825897	0.238983209	1	1	1
0.483837748	0.227978315	1	0.416730107	1
0.483999653	-0.238276984	0.989933981	1	1
0.484080638	0.237493834	1	0.850173656	1
0.484080638	0.237493834	1	0.850173656	1
0.484120864	-0.238081946	1	1	1
0.484173322	-0.203869546	0.584854032	0.126792252	1
0.484552204	0.234988024	1	0.704162183	1
0.48458539	-0.23464364	0.647493563	1	1
0.48458539	-0.23464364	0.647493563	1	1
0.484658796	-0.211120152	0.737584497	0.164911511	1
0.484658796	-0.211120152	0.737584497	0.164911511	1
0.485252213	-0.211856624	0.295435331	0.704162183	1
0.48555675	-0.180599177	0.043105813	0.242167012	1
0.485793454	0.227699682	0.439859057	1	1
0.485817168	-0.181874914	0.066393258	0.242167012	1
0.485842438	0.110862626	0.0008771	0.009270366	1
0.485842438	0.110862626	0.0008771	0.009270366	1
0.48591529	-0.098595872	0.008580492	0.000163543	1
0.485934474	0.128297117	0.003169634	0.025431136	1
0.485983061	0.199821125	0.169239932	0.489952655	1
0.485983061	0.199821125	0.169239932	0.489952655	1
0.486038239	-0.145380083	0.087427713	0.006372161	1
0.486119611	-0.197819539	0.146615922	0.489952655	1
0.486226855	-0.228831499	0.439859057	1	1
0.486514111	-0.23057264	1	0.489952655	1

0.486514111	-0.23057264	1	0.489952655	1
0.48656918	0.198220319	0.439859057	0.102782106	1
0.48656918	0.198220319	0.439859057	0.102782106	1
0.48656918	0.198220319	0.439859057	0.102782106	1
0.486620045	0.116556356	0.001207877	0.011906673	1
0.486620045	0.116556356	0.001207877	0.011906673	1
0.486944534	-0.220119187	0.412603306	1	1
0.487078583	-0.053339274	6.77E-05	8.55E-07	1
0.487078583	-0.053339274	6.77E-05	8.55E-07	1
0.487078583	-0.053339274	6.77E-05	8.55E-07	1
0.487078583	-0.053339274	6.77E-05	8.55E-07	1
0.487078583	-0.053339274	6.77E-05	8.55E-07	1
0.487078583	-0.053339274	6.77E-05	8.55E-07	1
0.487941655	0.232784664	0.584854032	1	1
0.48807817	0.165531628	0.205025676	0.02341474	1
0.48807817	0.165531628	0.205025676	0.02341474	1
0.488235508	0.232184709	1	0.704162183	1
0.488812177	0.21078244	0.295435331	0.704162183	1
0.488862393	-0.21493632	0.881481882	0.242167012	1
0.489433503	0.185452688	0.412603306	0.060775899	1
0.48947916	-0.181765905	0.412603306	0.053084396	1
0.489536534	-0.190598137	0.095538718	0.416730107	1
0.48963645	0.203333247	0.19926681	0.601859652	1
0.489659727	0.159668271	0.192696374	0.017403168	1
0.489876567	-0.147871657	0.095538718	0.006372161	1
0.489876567	-0.147871657	0.095538718	0.006372161	1
0.490000957	0.046748636	1.09E-06	9.47E-06	1
0.490000957	0.046748636	1.09E-06	9.47E-06	1
0.490000957	0.046748636	1.09E-06	9.47E-06	1
0.490131114	0.104531943	0.010835964	0.000324671	1
0.490232396	-0.155054667	0.169239932	0.011906673	1
0.490232396	-0.155054667	0.169239932	0.011906673	1
0.490442822	0.233983979	0.881481882	1	1
0.490442822	0.233983979	0.881481882	1	1
0.490442822	0.233983979	0.881481882	1	1
0.490442822	0.233983979	0.881481882	1	1
0.490462752	-0.212749981	0.307400178	0.850173656	1
0.490644546	-0.235101286	1	1	1
0.490772177	0.190831909	0.095538718	0.416730107	1
0.490863689	0.214793902	0.881481882	0.242167012	1
0.491091928	-0.230346258	0.584854032	1	1
0.491091928	-0.230346258	0.584854032	1	1
0.49138931	0.09845277	0.000295301	0.003736177	1
0.491599744	0.216585095	0.412603306	1	1
0.492084092	0.062183453	1.22E-05	0.000115129	1
0.492279273	0.234333471	1	1	1
0.492408127	-0.027736633	3.18E-07	9.50E-09	1
0.492704182	0.167823561	0.035148121	0.164911511	1
0.493672768	-0.139283594	0.075374611	0.005827837	1
0.49418232	-0.21426932	0.412603306	1	1
0.494263853	0.097929705	0.000467492	0.003736177	1
0.494263853	0.097929705	0.000467492	0.003736177	1
0.494878287	-0.22802028	1	0.645935818	1
0.494924747	-0.218506898	0.439859057	1	1
0.495005674	-0.07595588	0.0008771	1.93E-05	1
0.495005674	-0.07595588	0.0008771	1.93E-05	1
0.495213282	0.232703917	1	1	1
0.495213282	0.232703917	1	1	1
0.495213282	0.232703917	1	1	1
0.495213282	0.232703917	1	1	1
0.496066292	0.173805542	0.043105813	0.242167012	1
0.496066292	0.173805542	0.043105813	0.242167012	1
0.496503775	0.229930803	1	0.704162183	1
0.496611512	0.210150248	0.881481882	0.242167012	1

0.496655457	-0.23000649	0.737584497	1	1
0.496926652	0.099145212	0.00974133	0.0002997	1
0.496990323	0.068812104	1.56E-05	0.0002997	1
0.497003194	0.129068552	0.043105813	0.002420613	1
0.497003194	0.129068552	0.043105813	0.002420613	1
0.497045794	-0.231114881	1	1	1
0.497056707	0.192350406	0.439859057	0.102782106	1
0.497056707	0.192350406	0.439859057	0.102782106	1
0.497056707	0.192350406	0.439859057	0.102782106	1
0.497246421	-0.18868056	0.095538718	0.416730107	1
0.497430782	0.214866603	0.412603306	1	1
0.497545222	0.184762774	0.439859057	0.066020955	1
0.497545222	0.184762774	0.439859057	0.066020955	1
0.497627767	0.143457327	0.095006685	0.006372161	1
0.497627767	0.143457327	0.095006685	0.006372161	1
0.497849183	0.165615265	0.205025676	0.025431136	1
0.497849183	0.165615265	0.205025676	0.025431136	1
0.497849183	0.165615265	0.205025676	0.025431136	1
0.498000035	0.035378264	9.36E-08	3.06E-06	1
0.498162109	-0.231178166	1	1	1
0.498175854	0.223103315	1	0.489952655	1
0.498175854	0.223103315	1	0.489952655	1
0.498235472	0.113212568	0.001207877	0.011906673	1
0.498438848	-0.025808173	1.10E-07	6.53E-09	1
0.498438848	-0.025808173	1.10E-07	6.53E-09	1
0.498438848	-0.025808173	1.10E-07	6.53E-09	1
0.498438848	-0.025808173	1.10E-07	6.53E-09	1
0.498819713	-0.224553856	0.584854032	1	1
0.498819713	-0.224553856	0.584854032	1	1
0.498925511	0.106080984	0.0008771	0.006372161	1
0.498925511	0.106080984	0.0008771	0.006372161	1
0.498925511	0.106080984	0.0008771	0.006372161	1
0.498925511	0.106080984	0.0008771	0.006372161	1
0.498958608	0.211808976	0.88322726	0.242167012	1
0.49924584	0.150434567	0.018394332	0.085309726	1
